# Cation-tuned acidic electrified interface for hydrogen peroxide electrosynthesis with industrial-level current densities in natural seawater

**DOI:** 10.1038/s41467-026-72026-2

**Published:** 2026-04-20

**Authors:** Peike Cao, Xuanchen Liu, Yanming Liu, Zihao Zhao, Shuo Chen, Hongtao Yu, Jingguang G. Chen, Xie Quan

**Affiliations:** 1 Key Laboratory of Industrial Ecology and Environmental Engineering (Ministry of Education, China), School of Environmental Science and Technology, Dalian University of Technology, Dalian, PR China; 2 Department of Chemical Engineering, Columbia University, New York, NY USA

**Keywords:** Electrocatalysis, Electrocatalysis, Electrocatalysis

## Abstract

Electrocatalytic oxygen reduction reaction in seawater represents a sustainable approach for hydrogen peroxide (H_2_O_2_) production, yet industrial-level current densities trigger severe cathodic alkalization and scaling issues, while aggressive acidification of the reaction system compromises catalytic efficiency. Here we show a cationic modification strategy that dynamically modulates the acidic electrified interface to promote both the formation and desorption of the key *OOH intermediate for H_2_O_2_ synthesis. Enabled by this strategy, the cationic-modified catalysts achieve >90% efficiency at 500 mA cm^-2^ in natural seawater, and even reach 1.125 A cm^-2^ in high-salinity electrolytes, with a competitive estimated cost of $0.64 per kilogram of H_2_O_2_. Ab initio molecular dynamics simulations reveal that the introduced cationic modifications effectively counteract O–O bond cleavage induced by both the inherent strong binding of catalytic sites and the potential-induced over-binding effect under highly negative potentials, and thus facilitate *OOH desorption for H_2_O_2_ formation. This work highlights dynamic interfacial intermediate stabilization as a strategy that complements conventional static binding-energy tuning, enabling high-current-density H_2_O_2_ electrosynthesis in seawater.

## Abstract (web summary)

Hydrogen peroxide electrosynthesis from seawater offers a sustainable alternative to the energy-intensive anthraquinone process. Here, the authors report that a cationic modification strategy tunes the electrified interface to achieve ampere-level current density with high efficiency in seawater.

## Introduction

Hydrogen peroxide (H_2_O_2_) is an essential and environmentally benign oxidant, widely employed in disinfection, bleaching, and water treatment, with water and oxygen as its only decomposition products. Current commercial H_2_O_2_ production relies almost entirely on the anthraquinone process, which suffers from high energy demand, hazardous waste discharge, and safety risks stemming from hydrogen use and the instability of concentrated H_2_O_2_ solutions during storage and transport^1,2^. These drawbacks have motivated the pursuit of more sustainable, decentralized, and in-situ synthesis methods. The electrochemical synthesis of H_2_O_2_ via the two-electron oxygen reduction reaction (2e^–^ ORR) presents a promising sustainable alternative, which operates under ambient conditions using only water and O_2_ as feedstocks^3,4^, thereby reducing reliance on fossil-derived inputs and precious metal catalysts.

The alkaline 2e^–^ ORR has achieved promising performance for H_2_O_2_ production, with current densities of 500–1000 mA cm^-2^ and Faradaic efficiencies exceeding 80%^5–10^. However, its practical adoption is hindered by both the inherent decomposition of H_2_O_2_ under alkaline conditions^11,12^, and the reliance on concentrated alkaline electrolytes, which are costly and difficult to recycle. Acidic media offer enhanced H_2_O_2_ stability and higher oxidative capacity, which is beneficial for downstream applications such as water treatment, and lithium-ion battery recycling^13,14^. Nevertheless, acidic 2e^–^ ORR systems generally exhibit significantly inferior activity and selectivity compared to alkaline systems, and are further plagued by its reliance on precious metal catalysts (e.g., PdAu alloys) or complex synthesis of catalysts such as precisely engineered coordination of cobalt single-atom^15–20^. Recent studies indicate that introducing alkali metal cations into acidic electrolytes can markedly enhance both current density and selectivity for H_2_O_2_ production^21–24^. However, the promoting effect of alkali metal cations can be severely compromised under demanding conditions such as high current densities and low pH, as it hinges on a synergy with the catalytic material and other reaction parameters (e.g., applied potential and electrolyte composition). Thus, these systems still fail to reach current densities on the order of A cm^-2^ in acidic systems. Moreover, the need for continuous metal-salt addition raises operating costs and complicates waste management.

The utilization of natural seawater or low-value, high-salinity industrial wastewater as electrolytes offers an attractive route to minimize consumption of both fresh water and salt feedstocks. Seawater, rich in Na^+^, Cl^−^, and SO_4_^2−^, has already been validated as a viable electrolyte in water electrolysis for green hydrogen production^25^. Similarly, industrial high-salinity wastewater, typically with salinity exceeding 3.5 wt%, could serve as a low-cost feedstock. Moreover, direct H_2_O_2_ electrosynthesis in such media provides a practical in-situ strategy for the remediation of contaminated seawater and industrial wastewater^26^. However, 2e^–^ ORR in natural seawater remains relatively underexplored and is currently constrained by low current densities ( < 100 mA cm^−2^) compared to pure electrolytes^27–30^. The precipitation of Ca^2+^ and Mg^2+^ ions is the central issue, which is triggered by H^+^ consumption and a consequent local pH rise during ORR. The precipitation intensifies with increasing current density, causing performance degradation, as the insulating deposits block active sites and impede mass transport^25,31^.

Sufficient acidification of the electrolyte can maintain high interfacial H^+^ concentration and prevent precipitation at high current densities, however, it accelerates the competing 4e^−^ ORR pathway by influencing the adsorption of the key *OOH intermediate, and even causes hydrogen evolution reaction^32–34^. In the 2e^−^ ORR, *OOH is formed through the hydrogenation of *O_2_ and subsequently desorbs to yield H_2_O_2_. The binding strength of *OOH on the active sites serves as the primary descriptor within the established volcano relationship, determining the overall activity and H_2_O_2_ selectivity^16,35,36^. However, the collective influence of the electrified cathode interface that consists of the charged catalyst surface, electrolyte, cations, and H^+^ under the acidic conditions formed at highly negative potentials on the 2e^−^ ORR remains inadequately understood^37,38^. This knowledge gap highlights the critical need to engineer the entire acidic electrified interface, rather than solely the catalyst material, to simultaneously suppress precipitation and achieve high 2e^−^ ORR selectivity in acidic media. Compounding this challenge, the intrinsic heterogeneity of catalytic sites causes wide deviations of *OOH binding energies from the theoretical optimum^39–41^. It is further exacerbated under industrial-current-density operation, where applied potentials perturb adsorption behavior and impair selectivity^42^.

In this work, we report a cationic modification strategy that dynamically tunes the acidic electrified interface at the cathode to promote *OOH formation and facilitate its desorption for selective 2e^−^ ORR. This approach works effectively with various carbon-based active sites, regardless of their intrinsic strong or weak *OOH binding affinity, and thereby extends the operational window for selective H_2_O_2_ production beyond the narrow optimal range defined by the conventional volcano plot relationship. We validated this strategy using cationic-modified carbon nanotube catalysts, which enable selective H_2_O_2_ synthesis in natural seawater and high-salinity water, achieving a Faradaic efficiency of over 90% across a current density range of 25–500 mA cm^−2^. In contrast, the unmodified catalysts only exhibit a Faradaic efficiency of 44–78% at 25–225 mA cm^−2^, Correspondingly, the modified catalysts achieve a fourfold enhancement in H_2_O_2_ productivity. The modified catalyst  achieves an industrially relevant current density of 1.125 A cm^−2^ for acidic H_2_O_2_ electrosynthesis, with an estimated production cost of $0.64 per kg of H_2_O_2_ that is competitive with the commercial manufacturing method. We further employed ab initio molecular dynamics (AIMD) simulations to elucidate the reaction pathway of the 2e^−^ ORR and the role of cationic modification at the electrified acidic interface. Our simulation results reveal that while *OOH formation is favorable under both proton-rich and proton-deficient conditions, the electrified interface directly induces subsequent O–O bond cleavage. The introduced cationic modifiers play a critical role in mediating the efficient desorption of *OOH into the solution phase as H_2_O_2_/HO_2_^−^, thus mitigating the adverse effects of both the intrinsic strong and weak *OOH binding on unmodified catalysts and the potential-induced over-binding at the electrified interface.

## Results

### Structural characterization of cationic-modified carbon catalysts

**Fig. 1:**
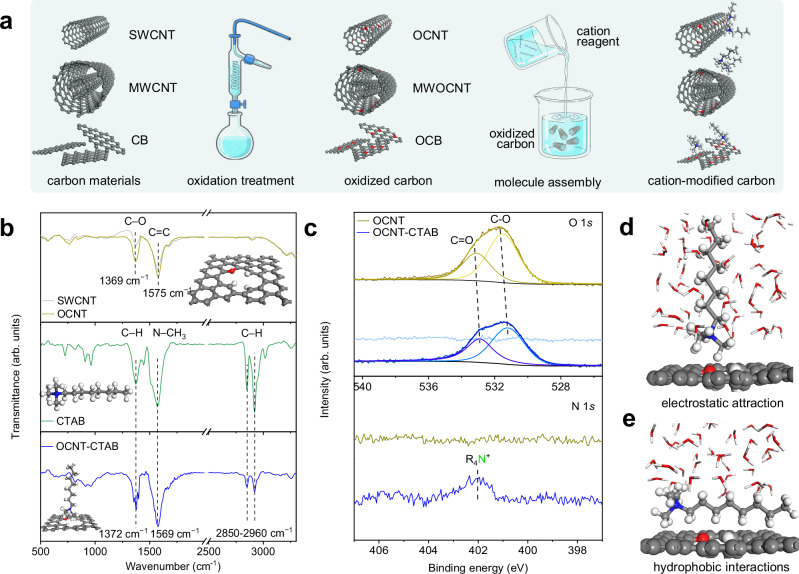
Characterization of cationic modified carbon catalysts. **a** Schematic illustration of the preparation process for cationic-modified carbon catalysts. **b** FT-IR spectra of SWCNT, OCNT, CTAB, and OCNT-CTAB showing characteristic functional groups and the insets depict proposed molecular models of OCNT and OCNT-CTAB. **c** High-resolution XPS spectra of O 1 *s* and N 1 *s* of OCNT and OCNT-CTAB. **d**, **e** Schematic illustration of electrostatic and hydrophobic interactions between a CTAB molecule and the OCNT surface in a water environment. (White, gray, red, and blue spheres represent H, C, O, and N atoms, respectively. Source data for Fig. 1 are provided as a Source Data file).

Among various H_2_O_2_-selective catalysts, carbon-based materials have emerged as promising catalysts for the 2e^–^ ORR due to their inherent selectivity and wide operational potential window^43–45^. We investigated commercially available single-walled carbon nanotubes (SWCNTs), multi-walled carbon nanotubes (MWCNTs), and carbon black as precursor catalysts for acidic H_2_O_2_ production. Prior to cationic functionalization, the carbon materials underwent controlled oxidation to generate oxygen-containing functional groups, causing negatively charged supports (designated as OCNT when derived from SWCNTs). A simple impregnation method was used to assemble cationic agents onto the oxidized carbon support, forming cationic-modified catalysts. This approach was versatile with diverse cationic compounds, including quaternary ammonium bromide surfactants (OTAB, DTAB, CTAB, STAB) and cationic ionomers (Sus, Fum, and QAPPT), as illustrated in Fig. 1a.

Scanning electron microscopy (SEM) images (Supplementary Fig. 1) revealed that pristine SWCNTs exhibited significant aggregation due to strong π–π interactions. This stacking was notably weakened after oxidation, which improved CNT dispersibility and facilitated effective interfacial assembly with CTAB. X-ray photoelectron spectra (XPS) and Raman spectra analyses confirmed increased oxygen content in OCNTs prepared at higher oxidation temperatures (Supplementary Fig. 2). Pristine SWCNTs displayed a sharp G band and a weak D band, indicating a low defect density. In contrast, oxidized OCNTs showed elevated I_D_/I_G_ ratios, suggesting that oxygen-containing functional groups would be preferentially incorporated at defect sites^46^, as schematically represented in Supplementary Fig. 3. Fourier-transform infrared (FTIR) spectra identified characteristic C–O stretching vibrations (1050–1300 cm^−1^) in OCNTs (Fig. 1b). CTAB, a quaternary ammonium compound, exhibited distinct vibrational modes including C–H stretching (2850–2960 cm^−1^), C–H bending (1370–1385 cm^−1^), and N–CH_3_ bending (~1500 cm^−1^). The presence of these features in the OCNT-CTAB composite confirmed successful CTAB immobilization. XPS analysis further validated the modification: OCNT-CTAB samples showed a distinct N 1 *s* peak at 402 eV, corresponding to quaternary N^+^ species, which was absent in unmodified OCNTs (Fig. 1c, Supplementary Figs. 4–5). High-resolution O 1 *s* spectra revealed a shift of the C–O peak to lower binding energy with reduced intensity after CTAB modification, indicating electronic interaction between OCNT and CTAB. The assembly of OCNT-CTAB can be driven in principle by electrostatic attraction between the positively charged −N^+^(CH_3_)_3_ groups and the negatively charged OCNT surface, with additional contributions from hydrophobic interactions. When electrostatic forces dominate, cationic headgroups adsorb onto the OCNT surface, orienting the alkyl chains outward to form a hydrophobic interface (Fig. 1d). Conversely, if hydrophobic interactions prevail, alkyl chains anchor to the surface, exposing ammonium groups and creating a hydrophilic interface (Fig. 1e). Contact angle measurements of the electrode surface confirmed increased surface hydrophobicity after CTAB modification (Supplementary Fig. 6), supporting the predominance of electrostatic-driven assembly. The simple and reproducible preparation of the cationic-modified catalysts offers a promising route for scalable electrocatalytic H_2_O_2_ synthesis.

### Cation-tuned H_2_O_2_ electrosynthesis in acidic electrolytes

**Fig. 2:**
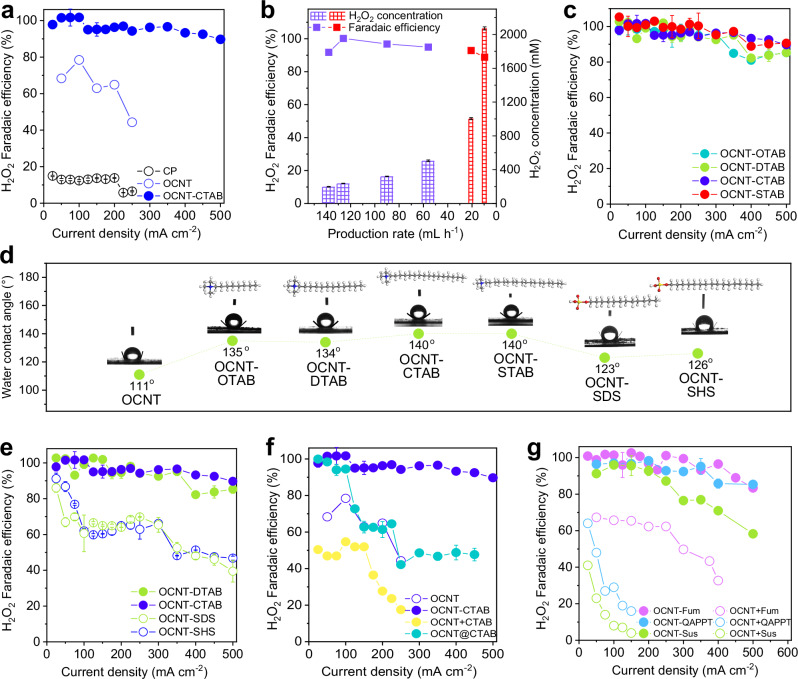
Performance evaluation of cationic-modified OCNT for acidic H_2_O_2_ electrosynthesis in high-salinity electrolytes. **a**, **b** H_2_O_2_ Faradaic efficiencies achieved on the bare CP substrate, OCNT, and OCNT-CTAB catalysts, and the H_2_O_2_ output concentrations at different catholyte flow rates under 300 mA cm^−2^ (red markers) or 400 mA cm^−2^ (purple markers). **c** Effect evaluation of cationic modifications on H_2_O_2_ Faradaic efficiencies. **d** Water contact angles on the surfaces of ionic-modified OCNT and unmodified OCNT and the insets show the molecular structures of the ionic modifiers. **e**–**g** Effects of anionic modifications, CTAB modification methods, and cationic ionomers (Sus, Fum, and QAPPT) on H_2_O_2_ Faradaic efficiencies. (Experimental parameters: two-compartment electrolytic cell with a cathode area of 4 cm^2^, 0.3 M K_2_SO_4_ in 0.1 M H_2_SO_4_ as the catholyte, 0.5 M H_2_SO_4_ as the anolyte, O_2_ flow rate of 30 mL min^−1^. Reported Faradaic efficiencies represent the average of triplicate measurements, with error bars indicating standard deviations. Two independent water contact angle measurements were performed for each sample, and the average value was reported. Source data for Fig. 2 are provided as a Source Data file).

We evaluated the H_2_O_2_ electrosynthesis performance of cationic-modified carbon catalysts using a custom two-cell flow electrolyzer. The catholyte consisted of an acidified high-salinity solution (0.3 M K_2_SO_4_ in 0.1 M H_2_SO_4_) to maintain an acidic interface at the cathode during 2e^–^ ORR, while the anolyte was a pure H_2_SO_4_ solution that enabled proton supply from the anode to the cathode compartment (Supplementary Fig. 7). The effects of CNT oxidation and cationic modification were investigated. As shown in Supplementary Fig. 8, pristine SWCNT achieved the Faradaic efficiency from 41.4 ± 2.8% to 88.4 ± 0.2% at 25–500 mA cm^−2^, while oxygen-doped OCNTs (e.g., OCNT-140-c) exhibited lower performance (18–52% at 25–175 mA cm^−2^), with efficiency declining as oxygen content increased. Oxygen doping has been reported to enhance ORR activity by creating active sites^47,48^; the observed performance loss in our case likely results from a concurrent decrease in surface hydrophobicity (Supplementary Fig. 9). This reduced hydrophobicity impairs O_2_ mass transfer and leads to electrolyte flooding^44,49^, thereby outweighing the benefit from the additional active sites. Notably, cationic modification substantially enhanced catalytic performance, as evidenced by the OCNT-140-c-CTAB catalyst’s Faradaic efficiencies of 99.5 ± 1.7% at 25 mA cm^−2^ and 72.6 ± 1.6% at 500 mA cm^−2^, compared to the lower efficiencies observed for the unmodified OCNT-140-c (Supplementary Fig.10). Further improvement was observed with the OCNT-CTAB catalyst, which exhibited a lower oxygen content and achieved over 90% Faradaic efficiency at higher currents (250–500 mA cm^−2^). In contrast, the unmodified OCNT catalyst displayed efficiencies ranging from 63 to 78% at lower currents (50–200 mA cm^−2^), with a notable drop to 44% at 250 mA cm^−2^ (Fig. 2a). This decrease in efficiency was primarily attributed to a shift toward a 4e⁻ ORR pathway. Hydrogen production efficiency for the OCNT catalyst remained below 2% up to 350 mA cm^−2^ but increased sharply to 16% when the current was raised to 400 and 500 mA cm^−2^ (Supplementary Fig. 11). The OCNT-CTAB catalyst maintained H_2_ production below 3% across the entire current range (50–500 mA cm^−2^), demonstrating its consistently high selectivity for H_2_O_2_ even at elevated current densities.

The OCNT-CTAB catalyst achieved an effective current density of 448.6 ± 10.0 mA cm^−2^ for acidic H_2_O_2_ production at a cell voltage of 5.2 V, representing a fourfold increase in H_2_O_2_ production rate compared to both SWCNT and OCNT (Supplementary Fig. 12). In contrast, the bare gas diffusion electrode exhibited negligible activity, confirming its minimal catalytic contribution. Importantly, the catholyte maintained an acidic pH throughout the electrolysis process (Supplementary Fig. 13), which is crucial for preventing the decomposition of the produced H_2_O_2_. The OCNT-CTAB catalyst also demonstrated over 90% Faradaic efficiency across current densities of 50–500 mA cm^−2^ in acidic catholytes with pH values of 0.8, 1.0, and 1.5 (Supplementary Fig. 14), highlighting its activity in acidic conditions. Furthermore, as shown in Fig. 2b, lowering the catholyte flow rate from 138 to 10 mL h^−1^ resulted in acidic H_2_O_2_ solutions with tunable concentrations ranging from 199 ± 3 mM to 2072 ± 18 mM, all while maintaining Faradaic efficiencies greater than 90%. This performance is in stark contrast to alkaline systems, where H_2_O_2_ concentrations typically remain below 3 wt% due to accelerated decomposition^11^.

To identify the active sites in cationic-modified carbon catalysts, we investigated the individual roles of defects and oxygen functional groups. Removing oxygen groups from OCNT while preserving the defect structure (OCNT-H-CTAB) resulted in only a minor decrease in Faradaic efficiency (83.9 ± 0.5% at 500 mA cm^−2^, Supplementary Fig. 15). This suggests that both defect sites and oxygen-containing groups contribute to the enhanced electrocatalytic activity observed with CTAB modification. Similar trends were also observed in MWCNT and carbon black catalysts (Supplementary Figs. 16–19), further supporting this conclusion. To understand the origin of the promotional effect of CTAB, we tested cationic modifiers with identical quaternary ammonium head groups but varying alkyl chain lengths (OTAB, DTAB, CTAB, STAB) and compared them with anionic surfactants (SDS, SHS). As shown in Fig. 2c, all cationic-modified OCNTs maintained Faradaic efficiencies >90% at 25–250 mA cm^−2^. Notably, CTAB and STAB preserved high Faradaic efficiency even at higher currents (250–500 mA cm^−2^), whereas OTAB and DTAB exhibited a slight decline (~80%). This decline correlated with their reduced capacity to enhance surface hydrophobicity (Fig. 2d). In contrast, anionic-modified OCNTs showed no significant improvement in H_2_O_2_ electrosynthesis, despite their increased hydrophobicity (Fig. 2e). This indicates that the performance enhancement strongly depends on the cationic nature of the modifier, rather than merely from improved hydrophobicity alone. To further test this hypothesis, we physically coated CTAB onto the OCNT electrode, forming a hydrophobic OCNT@CTAB interface (Supplementary Fig. 20). While this configuration enhanced performance in the low-current region, it yielded only minimal improvements at high currents compared to the unmodified OCNT electrode (Fig. 2f and Supplementary Fig. 21). Given that the electrode maintained its hydrophobicity during prolonged electrolysis, we can rule out CTAB detachment as a failure mechanism. This result confirms that hydrophobicity alone provides limited performance enhancement at high current densities, which was further confirmed by experiments with a hydrophobic dodecanethiol coating (Supplementary Fig. 22). Furthermore, we prepared an OCNT-MTAB electrode by modifying OCNT with the short-chain cationic surfactant tetramethylammonium bromide (MTAB), which exhibited hydrophilicity comparable to that of the OCNT (Supplementary Figs. 23–24). Although its Faradaic efficiency for H_2_O_2_ electrosynthesis was lower than that of OCNT-CTAB, especially at high current densities (Supplementary Fig. 24a), the MTAB-modified catalyst still showed substantial improvement over unmodified OCNT. This demonstrates that while cationic modification can enhance performance, strong surface hydrophobicity is crucial for achieving high Faradaic efficiency at high current densities.

The roles of electronic and mass-transfer effects induced by the cationic modifier were evaluated. Electrochemically active surface area measurements revealed no significant contribution of the cationic modifier to the electronic effects responsible for activity enhancement (Supplementary Figs. 25–27). Electrochemical impedance spectroscopy further confirmed that electron-transfer kinetics remained largely unchanged, and all cationic modifiers induced comparable mass-transport enhancements, suggesting that mass transport was not the primary factor in the observed performance improvements (Supplementary Fig. 28). To further investigate the importance of molecular-level assembly of OCNT and CTAB, we compared the OCNT-CTAB composite with physically mixed OCNT + CTAB samples, where CTAB was directly blended into the catalyst ink. The physically mixed OCNT+CTAB catalyst exhibited substantial performance degradation, underperforming even the unmodified OCNT. Although cationic compounds might block active sites, we minimized this risk by applying the cationic additive at one-tenth of the typical Nafion loading, following the non-blocking principle used in binder optimization (Supplementary Fig. 21b). Although the cationic modifier was applied across a wide range of concentrations, it still failed to outperform the OCNT, confirming the importance of  CTAB modification method. These findings collectively demonstrate that electrostatic modification of OCNTs with cationic groups, rather than a physical mixture, is crucial for achieving industrially relevant current densities in acidic H_2_O_2_ electrosynthesis. Furthermore, when cationic ionomers (such as Sus, Fum, and QAPPT) were chemically grafted onto OCNT, they also enhanced performance (Fig. 2g, Supplementary Fig. 29), highlighting the broad applicability of the cationic-functionalization strategy across various cationic agents and carbon supports. Although surfactants like CTAB have been reported to suppress hydrogen evolution and enhance selectivity in electrocatalytic CO_2_^50^ reduction and 2e⁻ ORR^51–54^, their direct addition to the electrolyte increases operational costs and only provides modest improvements in Faradaic efficiency and current density for acidic H_2_O_2_ synthesis^54^.

### Mechanism of cation-tuned acidic electrified interface

**Fig. 3:**
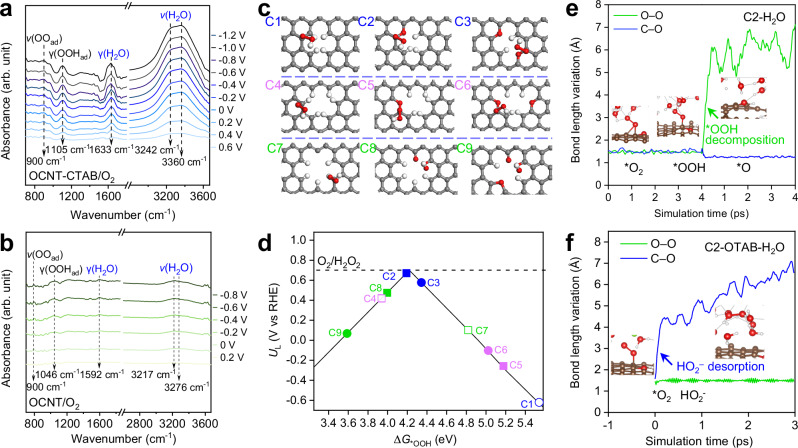
Mechanistic study on the promoting effect of cationic modifications for 2e^–^ ORR in acidic electrolytes. **a**, **b** In situ ATR-SEIRAS measurements on OCNT-CTAB and OCNT catalysts at gradually negatively shifted potentials in O_2_ saturated electrolyte. **c** Various types of carbon sites models on the OCNT catalyst for *OOH adsorption (white, gray, and red spheres represent H, C and O atoms, respectively). **d** *OOH binding energies at the evaluated carbon sites of the OCNT catalyst that was plotted on a volcano-type curve. **e**, **f** Bond length variation of O–O in *O_2_/*OOH intermediates and C–O between the O in *O_2_/*OOH and the C2 site of OCNT or OCNT-CTAB catalyst models during AIMD simulations, and the insets show snapshots of *O_2_ adsorption, *OOH cleavage or HO_2_^–^ desorption. (Source data for Fig. 3 are provided as a Source Data file and Supplementary data 1).

The electrochemical synthesis of H_2_O_2_ occurs at the electrified interface, a dynamic microenvironment formed by the charged catalyst surface, adsorbed reaction intermediates, and the surrounding electrolyte composition. To investigate the interactions within this interface, we conducted ¹H nuclear magnetic resonance (NMR) spectroscopy and in situ attenuated total reflectance surface-enhanced infrared absorption spectroscopy (ATR-SEIRAS) measurements on OCNT-CTAB. As shown in Supplementary Fig. 30, the ¹H chemical shift of water moved downfield upon CTAB addition, indicating deshielding of the water protons^51^. This effect can be attributed to hydrogen bonding between water molecules and the electronegative nitrogen atoms in the CTAB molecule, with the effect becoming more pronounced as the CTAB concentration increased. In situ ATR-SEIRAS further revealed cationic modification-dependent interfacial water restructuring during H_2_O_2_ electrosynthesis. As illustrated in Fig. 3a, characteristic vibrational bands were identified: *O_2_ species (600–900 cm^−1^), *OOH intermediates (1050–1100 cm^−1^), H–O–H bending of interfacial water (around 1600 cm^−1^), and O–H stretching of water molecules (3200–3400 cm^−1^)^55^. In the OCNT-CTAB/O_2_ system, these water-related peaks intensified under cathodic working potentials compared to an O_2_-excluded condition (Supplementary Fig. 31), implying the active involvement of interfacial water in proton transfer during H_2_O_2_ formation. In contrast, the unmodified OCNT exhibited minimal spectral changes (Fig. 3b), indicating poor water activation and limited ORR activity. These observations are linked to quaternary ammonium-induced water polarization, where the cationic headgroup generates a strong electric field that aligns adjacent water molecules via electrostatic interactions, thereby facilitating efficient proton relay.

To gain mechanistic insight into how cationic modification promotes H_2_O_2_ electrosynthesis at acidic electrified interface, we performed density functional theory (DFT) calculations to evaluate *OOH binding on the unmodified carbon catalysts and carried out AIMD simulations to trace the 2e^–^ ORR pathway to H_2_O_2_ at the catalyst/H_2_O/H_3_O^+^ interface. Given the established importance of oxygen doping and defects in H_2_O_2_ synthesis, we modeled carbon catalysts functionalized with C–O–C, C = O, and vacancy motifs (Fig. 3c). As shown in Fig. 3d single carbon vacancy (C1) exhibited weak *OOH binding, while oxygen doping (C2, C3) strengthened *OOH adsorption relative to the volcano peak. In multi-vacancy structures, oxygen doping modulated *OOH binding in contrasting ways: for C7-type carbon, binding strengthened at sites C8 and C9, whereas for C4-type carbon, it weakened at C5 and C6. Due to active site heterogeneity, this disparity in *OOH binding energies negatively impacted H_2_O_2_ selectivity, which could be especially critical at high current densities, where strong *OOH binding favors the competing O–O bond cleavage pathway.

We further employed AIMD simulations to study the 2e⁻ ORR pathway toward H_2_O_2_, specifically the sequential hydrogenation of *O_2_ to *OOH and *OOH to *H_2_O_2_ at cationic-modified carbon interfaces in H_2_O/H_3_O⁺ electrolyte. To more accurately simulate the 2e⁻ ORR under practical conditions, we introduced H_3_O^+^ ions into the water layer (pH 0) above the catalyst slab (Supplementary Fig. 32). During the hydrogenation of *O_2_ at the bare C2 site, both the O–O and C–O bond lengths remained nearly unchanged, indicating that O–O bond scission did not occur and *OOH was successfully formed and stably adsorbed (Fig. 3e, Supplementary Fig. 33). However, during the hydrogenation of *OOH, the O–O bond length increased abruptly from 1.5 Å to over 5 Å, signaling O–O bond cleavage, while the C–O bond shortened slightly from 1.56 Å to 1.23 Å. These results suggest that, at the C2- H_2_O interface, *OOH formation was favorable, but the acidic solvated environment led to its rapid decomposition, differing from the moderate *OOH binding observed under vacuum. In contrast, on the cationic-modified C2 site (C2-OTAB), the O–O bond of *O_2_ remained unchanged while the C–O bond elongated sharply, indicating that *O_2_ underwent hydrogenation and was converted into *H_2_O^–^ (Fig. 3f, Supplementary Fig. 34). This suggests that the cationic modification counteracts the overstabilization of *OOH in the electrified acidic interface, weakens its binding, and favors H_2_O_2_ formation. Cationic modifiers primarily function by electrostatically regulating the surface potential. To further explore their effect, we examined how they modulate H⁺ aggregation and the resulting hydrogenation behavior at the catalytic interface^56–58^. Previous studies have suggested that during high-current-density electrolysis in acidic conditions, rapid H⁺ consumption creates a proton-deficient interfacial region that can enhance H_2_O_2_ efficiency^22,59,60^. Our investigations revealed that cationic modification repelled H^+^ ions across different carbon sites (C2, C3, C8), as shown in Supplementary Figs. 35–37. At the bare C2 site, the proximity of H_3_O^+^ to the interface promoted *OOH decomposition, while repelling H⁺ from the interface prevented *OOH cleavage but did not efficiently promote desorption (Supplementary Figs. 33, 38). In contrast, the cationic modifier effectively repelled H_3_O^+^, shielding *O_2_/*OOH and suppressing their direct hydrogenation. This promoted an alternative proton-transfer pathway from H_2_O (Supplementary Figs. 34 and 39). Charge analysis revealed electron transfer from the carbon site to the bonding O atom in *OOH, accompanied by O–O bond elongation on the cationic-modified surface (Supplementary Figs. 40–41). The resulting weaker interaction between *OOH and the electrified catalytic surface promoted desorption over O–O cleavage.

We further verified this mechanism at the C8 surface, where cationic modification alleviated the excessively strong *OOH binding, reducing O–O bond cleavage and promoting HO_2_^−^ formation (Supplementary Figs. 42–43). Similar behavior was observed at C3 sites, where despite inherently weak *OOH binding, the electrified acidic interface over-stabilized *OOH adsorption. Cationic modifiers counteracted this effect by enhancing *OOH desorption, preventing it from becoming trapped on the catalytic site (Supplementary Figs. 44–45). However, for C9 sites with intrinsically strong *OOH binding, cationic modification had limited impact in altering the reaction pathway of 4e⁻ ORR (Supplementary Figs. 46–47). Ultimately, this cationic modification strategy effectively modulates the acidic electrified interface at negative potentials, promoting *OOH formation and desorption, thus enhancing selectivity for 2e⁻ ORR. While the model systems in DFT and AIMD simulations remain simplified, more accurate predictions of the electrocatalytic environment under operating conditions may be achieved through multiscale modeling approaches^61^.

### Assessment of H_2_O_2_ electrosynthesis using natural seawater

**Fig. 4:**
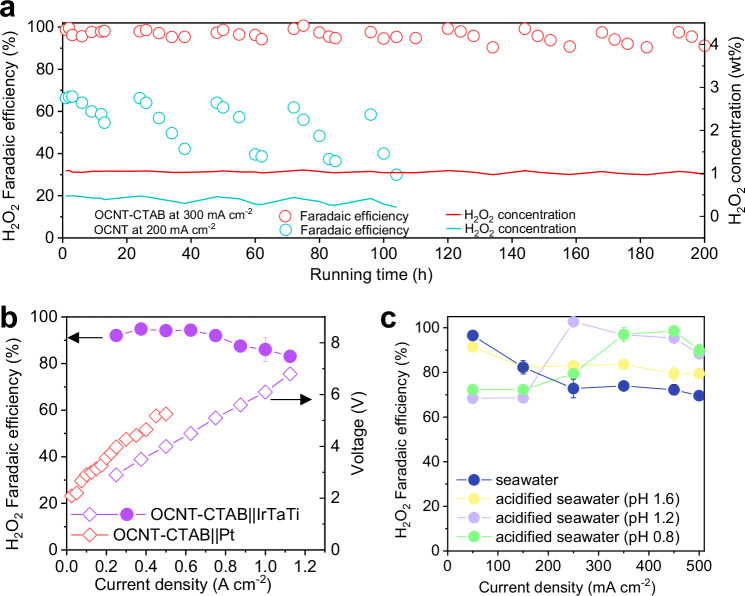
Performance assessment of acidic H_2_O_2_ electrosynthesis in high-salinity water and natural seawater. **a** Stability test of the OCNT-CTAB catalyst at 300 mA cm^−2^ and the unmodified OCNT catalyst at 200 mA cm^−2^. **b** H_2_O_2_ Faradaic efficiencies and current density-voltage plots achieved using a commercial IrTaTi anode compared to a Pt anode, both with an OCNT-CTAB cathode. **c** Performance evaluation of H_2_O_2_ electrosynthesis using acidified seawater as the catholyte. (Reported Faradaic efficiencies represent the average of triplicate measurements, with error bars indicating standard deviations. The cell voltage was recorded as the average of two or three measurements. Source data for Fig. 4 are provided as a Source Data file).

We further evaluated the practical potential of acidic H_2_O_2_ electrosynthesis through stability testing and techno-economic analysis. The OCNT-CTAB electrode exhibited operational stability, maintaining over 90% Faradaic efficiency during a 200-h continuous run at 300 mA cm^−2^ while consistently producing a 1 wt% H_2_O_2_ solution (Fig. 4a, Supplementary Fig. 48). Electrode stability was assessed by monitoring the effluent total organic carbon (TOC) and analyzing surface properties. The effluent TOC remained at approximately 0.5 mg L^−1^ over a subsequent 20-h period, a value consistent with the fresh electrolyte (Supplementary Fig. 49). Post-reaction characterization, including contact angle (wettability) and XPS (surface composition) measurements, revealed only a slight reduction in hydrophobicity, and the CTAB modifier remained effectively loaded with minimal leaching (Supplementary Figs. 50–52). When operated at a higher current of 400 mA cm^−2^, the system continuously generated ∼3 wt% H_2_O_2_ for over 60 h (Supplementary Fig. 53), confirming its durability under industrially relevant conditions. In contrast, the unmodified hydrophilic OCNT electrode experienced severe flooding at 200 mA cm^−2^, resulting in irreversible performance degradation. We also demonstrated efficient H_2_O_2_ production in acidified Na_2_SO_4_ catholyte, achieving >90% Faradaic efficiency at 300 mA cm^−2^ (Supplementary Fig. 54). However, when scaling to currents beyond 500 mA cm^−2^ with a Pt anode, significant anodic overpotential led to substantial Joule heating, increasing the catholyte temperature and causing partial H_2_O_2_ decomposition and catalyst detachment. This limitation was effectively addressed by replacing the Pt anode with a highly active commercial IrTaTi electrode, which has a lower overpotential (Supplementary Figs. 55–57). This change dramatically reduced the cell voltage, enabling stable operation at currents up to 1.125 A cm^−2^ while maintaining Faradaic efficiency above 80% (Fig. 4b)

We next explored the use of natural seawater as a sustainable electrolyte to reduce reliance on external salt inputs and freshwater consumption for H_2_O_2_ electrosynthesis. We investigated the impact of Cl⁻ ions on the electrolysis system and demonstrated that an acidification strategy could effectively address the key operational challenge of Ca^2+^/Mg^2+^ precipitation. To assess the influence of Cl⁻ on the ORR activity and 2e⁻ selectivity, we performed CV and rotating ring-disk electrode (RRDE) measurements. CV results (Supplementary Figs. 58–59 and Table 1) showed a positive shift in the ORR peak potential upon the addition of Cl⁻ to the K_2_SO_4_ electrolyte, indicating faster reaction kinetics in the presence of Cl⁻. Similarly, RRDE measurements revealed that the OCNT-CTAB catalyst exhibited an overpotential of 0.2 V for the 2e⁻ ORR (Supplementary Fig. 60), which remained virtually unchanged with or without Cl⁻ in the electrolyte. However, both the current density and H_2_O_2_ selectivity were significantly enhanced in the presence of Cl⁻, a trend observed for all tested catalysts. To further investigate whether Cl⁻ influences the 2e⁻ ORR process by directly affecting the catalytic activity or by altering the double-layer properties, we performed DFT calculations to examine the adsorption behavior of Cl⁻ on the active sites. As shown in Supplementary Figs. 61–62, Cl⁻ exhibited weak adsorption on the various carbon sites evaluated, making it unlikely to compete with the *OOH intermediate for adsorption. Therefore, we infer that the introduction of Cl⁻ does not significantly alter the electronic structure or the nature of the key active sites. Instead, it likely modulates the local environment or double-layer structure at the catalyst surface, thereby enhancing H_2_O_2_ electrosynthesis.

We next demonstrated highly efficient H_2_O_2_ electrosynthesis in an acidic high-salinity electrolyte containing Ca^2+^ and Mg^2+^ at concentrations representative of natural seawater, as well as in simulated seawater (0.5 M NaCl) acidified with concentrated HCl solution (Supplementary Figs. 63–64). Under these conditions, Faradaic efficiencies of 90–100% were achieved across 50–500 mA cm^−2^, with no observable precipitate formation after electrolysis. To further validate this approach, we used natural seawater collected from the Yellow Sea (Dalian, China). The concentrations of Na^+^, K^+^, Mg^2+^, and Ca^2+^ in the seawater sample were determined to be 9854, 411, 1303 and 555 mg L^−1^, respectively, as presented in Supplementary Fig. 65 and Tables 2–3. When unmodified seawater was used as the catholyte, Faradaic efficiency progressively declined with increasing current, accompanied by the formation of a dense layer of Ca^2+^/Mg^2+^ precipitates on the electrode surface (Fig. 4c, Supplementary Figs. 66–67). In contrast, acidification to pH 0.8–1.6 effectively suppressed scaling, allowing high Faradaic efficiency for H_2_O_2_ production even under high-current operation. Consequently, the electrode maintained a clean surface and stable performance during a 40-hour test in acidified seawater (Supplementary Figs. 68–70). Notably, our system achieved a current density of 500 mA cm^−2^ with >90% Faradaic efficiency in natural seawater, and further delivers an industrial-level current density of 1.125 A cm^−2^ in acidic electrolytes with competitive performance for acidic H_2_O_2_ synthesis (Supplementary Tables 4–5).

We evaluated and compared the operating costs for H_2_O_2_ electrosynthesis in various high-salinity electrolyte (under similar operating conditions) and in seawater (Supplementary Fig. 71). The analysis shows an operating cost range from $0.80 to $4.78 per kg H_2_O_2_, while acidified K_2_SO_4_ electrolyte consumption accounts for up to 49–85% of the total expense (Supplementary Fig. 72). When using the commercial IrTaTi anode to replace the Pt, the operating cost was reduced primarily due to lower electricity consumption (Supplementary Fig. 73). Substitution with less expensive Na_2_SO_4_ reduced the electrolyte cost contribution to 16% of the total, achieving an operating cost of $0.68/kg H_2_O_2_ (Supplementary Fig. 74). When natural seawater was used to completely replace the expensive artificially prepared electrolyte, an attractive operating cost of $0.77/kg H_2_O_2_ was achieved. A techno-economic assessment further considering capital expenditures highlights the economic promise of this approach for both acidified K_2_SO_4_- and seawater-based systems, with production costs of $0.64 and $0.80 per kg H_2_O_2_, respectively, compared to the commercial market price of H_2_O_2_ ($1.14/kg). Notably, acidic H_2_O_2_ remains stable up to 95 °C and is feasible for interim storage, unlike alkaline H_2_O_2_ that decomposes easily (Supplementary Fig. 75). Collectively, our results demonstrate both the technical feasibility and economic competitiveness of this approach for potential industrial-scale H_2_O_2_ production.

## Discussion

In summary, we have developed a cation-tuned electrified interface strategy that enables efficient H_2_O_2_ electrosynthesis at industrial-scale current densities using natural seawater or high-salinity water as electrolyte, while effectively suppressing cathodic scaling. This approach features several key advances: (1) This strategy allows carbon-based catalysts to achieve industrial-level current densities of 500–1125 mA cm^−2^ in acidic electrolytes and 500 mA cm^−2^ with a Faradaic efficiency of over 90% in seawater. By replacing conventional electrolytes with seawater or high-salinity water, this strategy presents a potentially sustainable alternative for H_2_O_2_ electrosynthesis. (2) Theoretical investigations reveal that cationic modifiers establish a favorable interfacial environment, which facilitates *OOH desorption for both intrinsically strong and weak *OOH-binding catalytic sites. This provides a feasible approach to overcome the limitations arising from active site heterogeneity and acidic electrified interfaces, and may expand the range of catalysts applicable for selective H_2_O_2_ generation. (3) Long-term durability tests verify stable operation of the catalytic system, with a Faradaic efficiency of over 90% retained for 200 h at 300 mA cm^−2^. Techno-economic analysis indicates that the production costs ($0.80/kg H_2_O_2_ for seawater and $0.64/kg H_2_O_2_ for K_2_SO_4_ electrolyte) are competitive relative to the commercial market price of H_2_O_2_ ($1.14/kg). Fundamentally, this work provides a perspective for electrocatalytic H_2_O_2_ synthesis that emphasizes dynamic intermediate stabilization at the electrified interface, in addition to conventional strategies focused on tuning the static binding energies of catalytic sites. This strategy offers insights that extend beyond typical volcano-plot relationships and provides a viable route toward high-current-density H_2_O_2_ electrosynthesis systems.

## Methods

### Chemicals and materials

Octyl trimethyl ammonium bromide (OTAB, 98%, Aladdin), dodecyl trimethyl ammonium bromide (DTAB, 99%, Maclin), cetyl trimethyl ammonium bromide (CTAB, 99%, Maclin), stearyl trimethyl ammonium bromide (STAB, 98%, Maclin), tetramethylammonium bromide (MTAB, 98%, Aladdin), sodium dodecyl sulfate (SDS, 99%, Maclin), and sodium hexadecyl sulfate (SHS, 95%, Maclin), K_2_SO_4_ (99%, Maclin) Na_2_SO_4_ (96%, Aladdin), isopropanol (99.5%, Maclin). Commercial single-wall carbon nanotube (SWCNT, 99%) and multi-wall carbon nanotube (MWCNT, 99%) powders were purchased from Shenzhen Nanotech Port Co., Ltd. (Shenzhen, China). The SWCNTs and MWCNTs had average diameters of approximately 10 nm and 50 nm, respectively. Commercial carbon black (CB, VXC 72) was obtained from Cabot Corporation. The Nafion solution (5 wt%) used for catalyst ink preparation was purchased from DuPont Co., Ltd. Teflon-treated hydrophobic carbon fiber papers (YLS-30T), used as the substrate for gas diffusion electrodes (GDEs), were manufactured by Toray Industries, Inc. (Japan). The proton exchange membrane used is Nafion 117 (DuPont) with a thickness of 183 μm. Sustainion imidazolium-functionalized polymers (Sus, 5 wt%) and Fumion FAA-3 (Fum, 10 wt%) were acquired from SCI Materials Hub, China. Quaternary ammonia poly(N-methyl-piperidine-co-p-terphenyl (QAPPT) was provided by the Yangtze River Delta Research Institute (Huzhou), University of Electronic Science and Technology of China. The Pt electrode (99.99%) was purchased from Tianjin Aida Hengsheng Technology Development Co., Ltd. IrTaTi oxide electrode with 0.1 mm thickness was purchased from Suzhou Shuerta Industrial Science & Technology Co., Ltd.

### Cationic-modified catalyst preparation

The cationic-modified catalysts were synthesized by integrating cationic compounds with various carbon supports, including SWCNTs, MWCNTs, and CB, through optimized modification processes. Prior to cationic integration, controlled oxidation of carbon materials was performed to introduce oxygen-containing functional groups via concentrated nitric acid treatment under reflux conditions. In a representative procedure, 1 g of SWCNT powder was uniformly dispersed in 150 mL of 27 wt% nitric acid solution under constant magnetic stirring. The oxidation process was conducted at 140 °C for 5 hours under reflux, followed by thorough deionized water washing and vacuum drying at 60 °C. Following the established procedure for SWCNT oxidation, additional supports including MWCNTs and CB were similarly processed to obtain MWOCNT and OCB. To enhance the oxidation degree and oxygen content of the SWCNT matrix, two key parameters were systematically optimized: (1) increasing the treatment temperature from 110 °C to 180 °C to obtain OCNT-110, OCNT-140 and OCNT-180, and (2) increasing the nitric acid concentration to 67 wt% to obtain OCNT-140-c. The cationic-modified catalysts were fabricated via an ultrasonic-assisted assembly strategy, employing oxidized carbon supports and diverse ionic modifiers. In a standardized protocol, 100 mg of oxidized carbon material and 0.03 mmol cationic agents were individually dispersed in ultrapure water, followed by controlled integration under ultrasonication at 25 °C to achieve molecular-level assembly. Subsequent post-treatments included filtration, washing with deionized water, and vacuum drying. This universal methodology enabled versatile ionic functionalization across: (1) multiple families of cationic surfactants—quaternary ammonium bromides (MTAB, OTAB, DTAB, CTAB, STAB), anionic sulfonic derivatives (SDS, SHS), cationic ionomers (Sus, Fum, QAPPT); and (2) varied carbon matrices (OCNT, MWOCNT, OCB).

### Catalyst characterization

Morphological features of catalysts were analyzed using field-emission scanning electron microscopy (FE-SEM, Hitachi S-4800). Crystalline phase identification was performed via X-ray diffraction (XRD, Bruker D8 Advance) with Cu Kα radiation. Surface chemical states were probed by X-ray photoelectron spectroscopy (XPS, Thermo ESCALAB250Xi) using monochromatic Al Kα excitation, with spectra deconvoluted employing Shirley backgrounds and Gaussian-Lorentzian peak fitting. Functional group analysis combined Fourier-transform infrared spectroscopy (FTIR) and XPS spectra. The defects were characterized by Raman D/G band intensity ratios (I_D_/I_G_). Electrode surface wettability was quantified via contact angle measurements using 5 μL deionized water droplets under ambient conditions.

### Electrode preparation

The catalytic ink formulation and electrode fabrication were carried out following a standardized protocol to ensure interfacial homogeneity and reproducibility. Specifically, 20 mg of catalyst powder was processed into catalytic ink through 60-minute ultrasonic dispersion in a solvent mixture composed of deionized water (1.6 mL), isopropanol (IPA, 0.4 mL), and a 5 wt% Nafion ionomer solution (100 μL), maintaining an optimized volume ratio of 8:2:0.5 (H_2_O:IPA:Nafion). The resulting homogenized ink (400 µL) was then precisely coated onto hydrophobic carbon fiber paper to fabricate gas diffusion electrodes (GDE) for electrolyzer testing. The loadings of the catalyst and Nafion were calculated to be 0.5 mg cm^−2^ and 120 µg cm^−2^, respectively. OCNT@CTAB and OCNT + CTAB electrodes were prepared for the comparative experiment. OCNT@CTAB electrode was achieved by coating a CTAB layer with loadings of 14, 28 and 55 µg cm^−2^ onto the surface of the OCNT catalytic layer. The OCNT + CTAB electrode was prepared by adding CTAB into the OCNT catalyst ink to achieve a final loading of 14, 28 and 55 µg cm^−2^. This loading is consistent with the amount used in the preparation of OCNT-CTAB.

### Electrochemical measurements

Electrochemical measurements were performed in a three-electrode system (single cell), which consisted of a working electrode, Hg/Hg_2_SO_4_ reference electrode (in saturated K_2_SO_4_), and Pt counter electrode (2 × 2 cm^2^). The working electrode was prepared by drop-casting the catalyst ink onto a glassy carbon electrode (3 mm diameter, geometric area of 0.07065 cm^2^) to form a catalyst loading of approximately 0.5 mg cm^−2^. Acidified 0.3 M K_2_SO_4_ was prepared by dissolving 52.28 g of K_2_SO_4_ in 0.1 M H_2_SO_4_ under ultrasonication. The pH of the as-prepared solution was measured to be 1.4 ± 0.1. The prepared electrolyte was stored in a sealed glass bottle at room temperature and used within three days. Cyclic voltammetry (CV) and linear sweep voltammetry (LSV) measurements were performed on a CHI760E electrochemical workstation. The recorded currents were normalized to the geometric area of the working electrode, and no *iR* correction was applied for potential. The reference electrode was calibrated against reversible hydrogen electrode (RHE) before testing. Potentials measured against the Hg/Hg_2_SO_4_ reference electrode were normalized to the RHE scale using the following equation:

1 $${E}_{{\rm{RHE}}}={E}_{{{\rm{Hg}}/{\rm{Hg}}}_{2}{{\rm{SO}}}_{4}}+0.615+0.059\times {\rm{pH}}$$

The electrochemically active surface area (ECSA) of the catalysts was evaluated by CV in the potential range of 0.80 to 0.92 V vs. RHE at scan rates of 20, 40, 60, 80, and 100 mV s^−1^. The test was performed in Ar-saturated electrolyte (0.3 M K_2_SO_4_ in 0.1 M H_2_SO_4_, 40 mL) to remove dissolved oxygen. In the non-Faradaic region, the current is primarily attributed to interfacial charging and discharging, following the relationship *I*=*C*_dl_×*v*, where *C*_dl_ is the double-layer capacitance. By measuring the capacitive currents at different scan rates, *C*_dl_ was determined. Assuming a specific capacitance (*C*_s_) per unit surface area of the catalyst, the ECSA was derived as ECSA= *C*_dl_/*C*_s_. Thus, the value of *C*_dl_ can be used to evaluate the ECSA. Electrochemical impedance spectroscopy (EIS) was conducted at open-circuit voltage in 0.3 M K_2_SO_4_ with 0.1 M H_2_SO_4_ (40 mL). The measurements were performed over a frequency range from 1 MHz to 0.1 Hz using a PARSTAT 2263 electrochemical workstation.

To assess the Cl⁻ effect on the ORR, CV curves were collected from 0 to 1.3 V vs. RHE at 50 mV s^−1^ in electrolytes containing varied Cl⁻ content (0.25 M K_2_SO_4_, 0.2 M K_2_SO_4_ + 0.1 M KCl or 0.5 M KCl, 40 mL). These tests were carried out under O_2_ or Ar- saturated conditions and an Ag/AgCl electrode was used as the reference electrode.

2 $${E}_{{\rm{RHE}}}={E}_{{\rm{Ag}}/{\rm{AgCl}}}+0.19+0.059\times {\rm{pH}}$$

We evaluated 2e^–^ ORR activity and H_2_O_2_ selectivity using the rotating ring-disk electrode (RRDE) setup (AFMSRCE type, Pine, Physicochemical Co. Ltd.). The working electrode was prepared by drop-casting the catalyst ink onto a RRDE to form a catalyst layer with a loading of approximately 0.6 mg cm^−2^ (15 μL catalyst ink, disk electrode area 0.2375 cm^2^). Polarization curves were recorded at a scan rate of 10 mV s^−1^ and a rotation rate of 1600 rpm in O_2_ or Ar- saturated 0.3 M K_2_SO_4_ electrolyte (100 mL). The H_2_O_2_ selectivity was calculated from the disk and ring currents using the equation:

3 $${{\rm{H}}}_{2}{{\rm{O}}}_{2}\%=\frac{2\times \frac{{I}_{{\rm{ring}}}}{N}}{{I}_{{\rm{disk}}}+\frac{{I}_{{\rm{ring}}}}{N}}\times 100\% $$

Where ${I}_{{\rm{disk}}}$ and$\,{I}_{{\rm{ring}}}$ are the disk and ring currents, respectively, and $N$ is the collection efficiency of the ring electrode (0.37 for the AFMSRCE electrode). Oxygen evolution reaction (OER) activities of IrTaTi and Pt were evaluated in 0.5 M H_2_SO_4_ by LSV with a scan rate of 10 mV s^−1^ and multi-step chronopotentiometry.

### H_2_O_2_ electrosynthesis performance measurements

Electrolysis evaluation for H_2_O_2_ synthesis utilized a custom flow-by reactor (double-chamber cell) featuring 4 cm^2^ gas diffusion electrodes, operated with single-pass catholyte (0.3 M K_2_SO_4_ in 0.1 M H_2_SO_4_, simulated seawater or natural seawater) and recirculated 0.5 M H_2_SO_4_ anolyte. The assembly configuration and components of the electrolysis cell were shown in Supplementary Fig. 7. The proton exchange membrane (Nafion 117, 3 × 3 cm^2^, thickness 183 μm) was used to separate the cathode and anode. Prior to use, the membrane was cleaned by boiling in 3% H_2_O_2_ for 1 h, followed by boiling in deionized water for 1 h, and then boiled in 0.5 M H_2_SO_4_ for 1 h to ensure proton exchange. The membrane was subsequently rinsed with deionized water and stored in deionized water. H_2_O_2_ electrosynthesis was carried out under constant current conditions using a power supply. Pure O_2_ was supplied to the cathode side facing the gas chamber at a flow rate of 30 mL min^−1^, controlled by a mass flow controller (Sevenstar D07, China). All experiments were conducted at room temperature, which ranged between approximately 25 °C and 30 °C. The H_2_O_2_ concentration was determined by the cerium sulfate titration method based on the stoichiometric reaction between Ce^4+^ and H_2_O_2_. In a typical measurement, 10–100 μL of the H_2_O_2_ solution collected from the reactor outflow was added to a known volume of 1 mM Ce^4+^ solution, reducing Ce^4+^ to Ce^3+^. The remaining Ce^4+^ concentration was measured spectrophotometrically at 316 nm and the H_2_O_2_ concentration was calculated from the Ce^4+^ consumption. The Faradaic efficiency for H_2_O_2_ is calculated by the formula:

4 $$\mathrm{FE}\left(\% \right)=\frac{{C}_{{{\rm{H}}}_{2}{{\rm{O}}}_{2}}\times {Q}_{\mathrm{catholyte}}\times n\times F}{I\times 3600}\times 100\% $$

Where ${C}_{{{\rm{H}}}_{2}{{\rm{O}}}_{2}}$ and ${Q}_{\mathrm{catholyte}}$ represent H_2_O_2_ concentration (mol L^−1^) and flow rate of H_2_O_2_ solution (L h^−1^), respectively. $I$ represents applied current (A). $n$ is the number of electrons for 2e^–^ ORR to H_2_O_2_ (*n* = 2), and $F$ is the Faraday constant (96485 C mol^-1^). Byproduct H_2_ was detected using a gas chromatograph (Shimadzu GC-2014) equipped with a thermal conductivity detector (TCD), and its Faradaic efficiency was calculated as follows:

5 $${\rm{FE}}\left(\% \right)=\frac{{C}_{{{\rm{H}}}_{2}}\times V\times n\times F}{{V}_{{\rm{m}}}\times I\times 3600}\times 100\% $$

Where ${C}_{{{\rm{H}}}_{2}}$ and $V$ represent the H_2_ concentration (mol mol^−1^) and the total volume of collected gas (L), respectively. $I$ represents applied current (A). $n$ is the number of electrons transferred per mole of H_2_ generated (*n* = 2). ${V}_{{\rm{m}}}$ is the molar volume of gas (24.45 L mol^-1^ at 25 °C and atmospheric pressure), and $F$ is the Faraday constant (96485 C mol^−1^). The seawater used in this study was collected from the Yellow Sea off the coast of Dalian city (39° N, 122° E). Prior to electrochemical testing, it was pre-filtered through a 0.22 μm polytetrafluoroethylene membrane to remove suspended solids. The concentrations of Na^+^, K^+^, Mg^2+^, and Ca^2+^ in the samples were determined using an ion chromatograph (Thermo Scientific DIONEX AQUION RFIC) equipped with a Dionex IonPac CS16 5 × 150 mm separation column and a 30 mM aqueous methanesulfonic acid solution as the mobile phase. A mixed standard solution containing 50.0 mg L^−1^ of K^+^, 250 mg L^−1^ of Na^+^, 250 mg L^−1^ of Ca^2+^, and 50.0 mg L^−1^ of Mg^2+^ was prepared. It was then diluted into a series of standard samples with varying concentrations to establish a calibration curve.

### ATR-SEIRAS measurements

In situ attenuated total reflectance surface-enhanced infrared absorption spectroscopy (ATR-SEIRAS) experiments were performed on a Bruker Vertex80V Fourier transform infrared spectrometer equipped with a liquid nitrogen-cooled mercury cadmium telluride (MCT) detector. The working electrode was fabricated through a two-step deposition protocol^8^: (1) chemical deposition of a 50-nm Au film on a hemispherical Si prism substrate, followed by (2) controlled drop-casting of catalyst ink to achieve uniform surface coverage. All electrochemical measurements were conducted in a dual-compartment H-cell configuration, employing 0.3 M K_2_SO_4_ in 0.1 M H_2_SO_4_ as the catholyte and 0.5 M H_2_SO_4_ as the anolyte. Spectral acquisition was synchronized with the application of different potentials under O_2_ conditions, with background spectra collected in Ar-saturated electrolyte prior to each measurement series.

### Density functional theory calculations

We investigated the *OOH binding properties on diverse carbon-based models through density functional theory (DFT) calculations implemented in the Vienna Ab-initio Simulation Package (VASP)^62^. The electron-ion interaction was described using projector-augmented wave (PAW) pseudopotentials combined with the Perdew-Burke-Ernzerhof (PBE) exchange-correlation functional under the generalized gradient approximation (GGA) framework^37,63^. Computational parameters were carefully optimized with a plane-wave cutoff energy of 520 eV and gamma-centered 2 × 2 × 1 *k*-point mesh sampling. Structural relaxations were conducted until atomic forces reached convergence criteria below 0.01 eV Å^−1^, while electronic iterations maintained rigorous energy convergence thresholds of 1 × 10^−6 ^ eV per atom. The simulation methodology and data processing protocols are based on well-validated computational approaches and post-processing techniques, as documented in previous studies^35,36,64,65^. We performed ab initio molecular dynamics (AIMD) simulations to investigate the electrocatalytic interface formed by OCNT-based catalysts and aqueous electrolyte. The acidic system was modeled with a 1:72 molar ratio of H_3_O^+^ to H_2_O molecules, corresponding to a proton concentration equivalent to pH 0.1. The OCNT-water interface was modeled by positioning 72 explicit water molecules above the OCNT slab, with acidic (H_3_O^+^) electrolyte environment modeled through appropriate ion additions. Following this protocol, we built catalytic interfaces for OCNT-OTAB catalyst with different active motifs. To model the 2e^–^ ORR to H_2_O_2_ under high-current-density operation in acidic electrolyte, we constructed a locally proton- deficient catalyst–water interface, while positioning the solvated protons (H_3_O^+^) away from the interfacial region. The introduction of H_3_O^+^ to the electrocatalytic interface triggers spontaneous charge compensation, where an equivalent number of electrons are injected into the catalytic surface to maintain electroneutrality and modulate the electrode potential (*U*_RHE_). The ${U}_{\mathrm{RHE}}$ was calculated using the equation

6 $${U}_{\mathrm{RHE}}=\frac{(\varPhi -{\varPhi }_{\mathrm{SHE}})}{{\rm{e}}}+0.0592\times \mathrm{pH}$$

where $\varPhi$ and ${\rm{e}}$ represent the work function and elementary charge, respectively. ${\varPhi }_{{\rm{SHE}}}$ = 4.44. Potential values were averaged from five representative configurations along the AIMD trajectory. All simulations employed a 400 eV plane-wave cutoff energy and utilized a gamma-centered 1 × 1 × 1 *k*-mesh without symmetry constraints. We incorporated van der Waals interactions through Grimme’s DFT-D3 correction with zero-damping function. Full atomic relaxation was permitted during simulations, with temperature control (300 K) maintained in the NVT ensemble using a Nose-Hoover thermostat. Each AIMD simulation spanned at least 4 ps with a 1.0 fs integration timestep, aiming to provide adequate sampling of the catalytic interface dynamics. The optimized structures for *OOH adsorption and Cl⁻ adsorption on carbon sites, and the initial and final models from AIMD simulations are provided in Supplementary Data 1.

## Supplementary information

Supplementary Information

Description of Additional Supplementary Files

Supplementary Data 1

Transparent Peer Review file

## Source data

Source Data

## Data Availability

Source data are provided with this paper.

## References

[1] Li, H. et al. Advances in the slurry reactor technology of the anthraquinone process for H_2_O_2_ production. *Front. Chem. Sci. Eng.* **12**, 124–131 (2017).

[2] Ingle, A. A. et al. Progress and prospective of heterogeneous catalysts for H_2_O_2_ production via anthraquinone process. *Environ. Sci. Pollut. Res. Int.* **29**, 86468–86484 (2022). 35710969 10.1007/s11356-022-21354-z PMC9203146

[3] Berl, E. A new cathodic process for the production of H_2_O_2_. *Trans. Electrochem. Soc.* **76**, 359 (1939).

[4] Deng, Z. et al. Advancing H_2_O_2_ electrosynthesis: enhancing electrochemical systems, unveiling emerging applications, and seizing opportunities. *Chem. Soc. Rev.* **53**, 8137–8181 (2024). 39021095 10.1039/d4cs00412d

[5] Wu, Y. et al. Electrochemically synthesized H_2_O_2_ at industrial-level current densities enabled by in situ fabricated few-layer boron nanosheets. *Nat. Commun.* **15**, 10843 (2024). 39737981 10.1038/s41467-024-55071-7 PMC11685507

[6] Jia, S. et al. Modulated nickel single-atom sites as highly active catalysts for the synthesis of neutral H_2_O_2_ at ampere-level current densities. *ACS Nano* **19**, 22402–22413 (2025). 40491021 10.1021/acsnano.5c06049

[7] Song, M. et al. Single-atom catalysts for H_2_O_2_ electrosynthesis via two-electron oxygen reduction reaction. *Adv. Funct. Mater.* **33**, 2212087 (2023).

[8] Cao, P. et al. Metal single-site catalyst design for electrocatalytic production of hydrogen peroxide at industrial-relevant currents. *Nat. Commun.* **14**, 172 (2023). 36635287 10.1038/s41467-023-35839-z PMC9837053

[9] Ding, S. et al. An abnormal size effect enables ampere-level O_2_ electroreduction to hydrogen peroxide in neutral electrolytes. *Energy Environ. Sci.* **16**, 3363–3372 (2023).

[10] Jung, E. et al. Recent advances in electrochemical oxygen reduction to H_2_O_2_: catalyst and cell design. *ACS Energy Lett.* **5**, 1881–1892 (2020).

[11] Ni, C. et al. Inhibiting joule heating to enhance the hydrogen peroxide electrosynthesis efficiency at industrial level current density. *ACS Appl. Energy Mater.* **7**, 3116–3124 (2024).

[12] Levikhin, A. A. et al. Hydrogen peroxide—a promising oxidizer for rocket engines: physical and chemical properties: decomposition in the liquid phase. *Adsorption* **30**, 2187–2217 (2024).

[13] Fan, L. et al. Selective production of ethylene glycol at high rate via cascade catalysis. *Nat. Catal.* **6**, 585–595 (2023).

[14] He, L. P. et al. Leaching process for recovering valuable metals from the LiNi_1/3_Co_1/3_Mn_1/3_O_2_ cathode of lithium-ion batteries. *Waste Manage* **64**, 171–181 (2017). 10.1016/j.wasman.2017.02.011 28325707

[15] Chang, Q. W. et al. Promoting H_2_O_2_ production via 2-electron oxygen reduction by coordinating partially oxidized Pd with defect carbon. *Nat. Commun.* **11**, 2178 (2020). 32358548 10.1038/s41467-020-15843-3 PMC7195490

[16] Verdaguer-Casadevall, A. et al. Trends in the electrochemical synthesis of H_2_O_2_: enhancing activity and selectivity by electrocatalytic site engineering. *Nano Lett.* **14**, 1603–1608 (2014). 24506229 10.1021/nl500037x

[17] Deng, Z. et al. Pd 4 d orbital overlapping modulation on Au@Pd manowires for efficient H_2_O_2_ production. *J. Am. Chem. Soc.* **146**, 2816–2823 (2024). 38230974 10.1021/jacs.3c13259

[18] Lin, Z. et al. Atomic Co decorated free-standing graphene electrode assembly for efficient hydrogen peroxide production in acid. *Energy Environ. Sci.* **15**, 1172–1182 (2022).

[19] Sun, Y. et al. Activity–selectivity trends in the electrochemical production of hydrogen peroxide over single-site metal–nitrogen–carbon catalysts. *J. Am. Chem. Soc.* **141**, 12372–12381 (2019). 31306016 10.1021/jacs.9b05576

[20] Huang, H. et al. Enhancing H_2_O_2_ electrosynthesis at industrial-relevant current in acidic media on diatomic cobalt sites. *J. Am. Chem. Soc.* **146**, 9434–9443 (2024). 38507716 10.1021/jacs.4c02031

[21] Zhang, X. et al. Electrochemical oxygen reduction to hydrogen peroxide at practical rates in strong acidic media. *Nat. Commun.* **13**, 2880 (2022). 35610199 10.1038/s41467-022-30337-0 PMC9130276

[22] Cao, P. K. et al. Highly efficient acidic electrosynthesis of hydrogen peroxide at industrial-level current densities promoted by alkali metal cations. *Angew. Chem. Int. Ed.* **136**, e202406452 (2024). 10.1002/anie.202406452 38735843

[23] Hübner, J. L. et al. Cation effects on the acidic oxygen reduction reaction at carbon surfaces. *ACS Energy Lett* **9**, 1331–1338 (2024). 38633991 10.1021/acsenergylett.3c02743 PMC11019649

[24] Tian, Y. et al. Alkali metal cations induce pseudo-outer-sphere oxygen reduction reaction mechanism in electrolyte-catalyst synergy. *J. Am. Chem. Soc.* **147**, 35118–35127 (2025). 40947988 10.1021/jacs.5c12947

[25] Yu, L. et al. Direct seawater electrolysis for hydrogen production. *Nat. Rev. Mater.* **10**, 857–873 (2025).

[26] Peng, Q. et al. Prospects of advanced oxidation processes for high-salinity coking wastewater treatment: a strategy to support sustainable management. *Resour. Conserv. Recycl.* **212**, 107880 (2025).

[27] Wang, X. et al. A chlorine-resistant self-doped nanocarbon catalyst for boosting hydrogen peroxide synthesis in seawater. *Angew. Chem. Int. Ed.* **64**, e202419049 (2025). 10.1002/anie.202419049 39584455

[28] Zhao, Q. et al. Approaching a high-rate and sustainable production of hydrogen peroxide: oxygen reduction on Co–N–C single-atom electrocatalysts in simulated seawater. *Energy Environ. Sci.* **14**, 5444–5456 (2021).

[29] Nie, J. et al. Accelerating water dissociation to achieve ampere-level hydrogen peroxide electrosynthesis in brine and seawater. *Nat. Commun.* **16**, 5895 (2025). 40593612 10.1038/s41467-025-60950-8 PMC12214788

[30] Zhang, C. et al. Stable and high-yield hydrogen peroxide electrosynthesis from seawater. *Nat. Sustain.* **8**, 542–552 (2025).

[31] Gao, F. Y. et al. Seawater electrolysis technologies for green hydrogen production: challenges and opportunities. *Curr. Opin. Chem. Eng.* **36**, 100827 (2022).

[32] Viswanathan, V. et al. Unifying the 2e^-^ and 4e^-^ Reduction of Oxygen on Metal Surfaces. *J. Phys. Chem. Lett.* **3**, 2948–2951 (2012). 26292231 10.1021/jz301476w

[33] Liu, S. et al. Mechanism in pH effects of electrochemical reactions: a mini-review. *Carbon Lett.* **34**, 1269–1286 (2024).

[34] Ramaswamy, N. & Mukerjee, S. Influence of inner- and outer-sphere electron transfer mechanisms during electrocatalysis of oxygen reduction in alkaline media. *J. Phys. Chem. C* **115**, 18015–18026 (2011).

[35] Kulkarni, A. et al. Understanding catalytic activity trends in the oxygen reduction reaction. *Chem. Rev.* **118**, 2302–2312 (2018). 29405702 10.1021/acs.chemrev.7b00488

[36] Siahrostami, S. et al. Enabling direct H_2_O_2_ production through rational electrocatalyst design. *Nat. Mater.* **12**, 1137–1143 (2013). 24240242 10.1038/nmat3795

[37] Waegele, M. M. et al. How cations affect the electric double layer and the rates and selectivity of electrocatalytic processes. *J. Chem. Phys.* **151**, 160902 (2019). 31675864 10.1063/1.5124878

[38] Hussain, G. et al. How cations determine the interfacial potential profile: Relevance for the CO_2_ reduction reaction. *Electrochim. Acta* **327**, 135055 (2019).

[39] Wang, W. et al. Intrinsic carbon defects for the electrosynthesis of H_2_O_2_. *J. Phys. Chem. Lett.* **13**, 8914–8920 (2022). 36129314 10.1021/acs.jpclett.2c02684

[40] Han, G. F. et al. Building and identifying highly active oxygenated groups in carbon materials for oxygen reduction to H_2_O_2_. *Nat. Commun.* **11**, 2209 (2020). 32371867 10.1038/s41467-020-15782-z PMC7200778

[41] Chai, G. L. et al. Two-electron oxygen reduction on carbon materials catalysts: Mechanisms and active Sites. *J. Phys. Chem. C* **121**, 14524–14533 (2017).

[42] Yan, H. M. et al. Revealing the potential-dependent rate-determining step of oxygen reduction reaction on single-atom catalysts. *J. Am. Chem. Soc.* **147**, 3724–3730 (2025). 39808617 10.1021/jacs.4c16098

[43] Lu, Z. et al. High-efficiency oxygen reduction to hydrogen peroxide catalysed by oxidized carbon materials. *Nat. Catal.* **1**, 156–162 (2018).

[44] Xing, Z. et al. Interplay of active sites and microenvironment in high-rate electrosynthesis of H_2_O_2_ on doped carbon. *ACS Catal.* **13**, 2780–2789 (2023).

[45] Xia, C. et al. Direct electrosynthesis of pure aqueous H_2_O_2_ solutions up to 20% by weight using a solid electrolyte. *Science* **366**, 226–231 (2019). 31601767 10.1126/science.aay1844

[46] Zhou, J. et al. Tuning the reactivity of carbon surfaces with oxygen-containing functional groups. *Nat. Commun.* **14**, 2293 (2023). 37085515 10.1038/s41467-023-37962-3 PMC10121666

[47] Zhang, C. et al. A pentagonal defect-rich metal-free carbon electrocatalyst for boosting acidic O_2_ reduction to H_2_O_2_ production. *J. Am. Chem. Soc* **145**, 11589–11598 (2023). 37158560 10.1021/jacs.3c00689

[48] Bu, Y. et al. Carbon-based electrocatalysts for efficient hydrogen peroxide production. *Adv. Mater.* **33**, e2103266 (2021). 34562030 10.1002/adma.202103266

[49] Cao, P. K. et al. Durable and selective electrochemical H_2_O_2_ synthesis under a large current enabled by the cathode with highly hydrophobic three-phase architecture. *ACS Catal.* **11**, 13797–13808 (2021).

[50] Ge, W. et al. Modulating interfacial hydrogen-bond environment by electrolyte engineering promotes acidic CO_2_ electrolysis. *ACS Catal.* **14**, 10529–10537 (2024).

[51] Fang, Y. et al. Boosting hydrogen peroxide electrosynthesis via modulating the interfacial hydrogen-bond environment. *Angew. Chem. Int. Ed.* **62**, e202304413 (2023). 10.1002/anie.202304413 37160619

[52] Wu, K. H. et al. Highly selective hydrogen peroxide electrosynthesis on carbon: In situ interface engineering with surfactants. *Chem* **6**, 1443–1458 (2020).

[53] Fan, Y. et al. Mechanistic insights into surfactant-modulated electrode–electrolyte interface for steering H_2_O_2_ electrosynthesis. *J. Am. Chem. Soc.* **146**, 7575–7583 (2024). 38466222 10.1021/jacs.3c13660

[54] Adler, Z. et al. Hydrogen peroxide electrosynthesis in a strong acidic environment using cationic surfactants. *Precis. Chem.* **2**, 129–137 (2024). 39473527 10.1021/prechem.3c00096 PMC11504663

[55] Li, P. et al. Hydrogen bond network connectivity in the electric double layer dominates the kinetic pH effect in hydrogen electrocatalysis on Pt. *Nat. Catal.* **5**, 900–911 (2022).

[56] Ringe, S. et al. Understanding cation effects in electrochemical CO_2_ reduction. *Energy Environ. Sci.* **12**, 3001–3014 (2019).

[57] Resasco, J. A universal model of cation effects in electrocatalysis. *JACS Au* **5**, 5253–5266 (2025). 41311948 10.1021/jacsau.5c01115 PMC12648287

[58] Le, J. B. et al. Molecular understanding of cation effects on double layers and their significance to CO-CO dimerization. *Natl. Sci. Rev*. **10**_,_ nwad105 (2023). 10.1093/nsr/nwad105 PMC10575609 37842071

[59] Xie, Y. et al. High carbon utilization in CO_2_ reduction to multi-carbon products in acidic media. *Nat. Catal.* **5**, 564–570 (2022).

[60] Arquer, F. P. G. CO_2_ electrolysis to multicarbon products at activities greater than 1 A cm^−2^. *Science* **367**, 661–666 (2020). 10.1126/science.aay4217 PMC13531662 32029623

[61] Qian, S. J. et al. Advances in computational electrocatalysis: modeling reaction kinetics in realistic electrochemical environments. *J. Phys. Chem. Lett.* **17**, 3394–3405 (2026). 41823175 10.1021/acs.jpclett.6c00391

[62] Kresse, G. & Hafner, J. Ab initio molecular dynamics for liquid metals. *Phys. Rev. B* **47**, 558–561 (1993). 10.1103/physrevb.47.558 10004490

[63] Blochl, P. E. Projector augmented-wave method. *Phys. Rev. B* **50**, 17953–17979 (1994). 10.1103/physrevb.50.17953 9976227

[64] Nørskov, J. K. et al. Origin of the overpotential for oxygen reduction at a fuel-cell cathode. *J. Phys. Chem. B* **108**, 17886–17892 (2004). 39682080 10.1021/jp047349j

[65] Wang, V. et al. VASPKIT: a user-friendly interface facilitating high-throughput computing and analysis using VASP code. *Comput. Phys. Commun.* **267**, 108033 (2021).

