# Supplementary material for: Cation-tuned acidic electrified interface for hydrogen peroxide electrosynthesis with industrial-level current densities in natural seawater
Source: Nat Commun. 2026 Apr 20;17:5443. doi: 10.1038/s41467-026-72026-2 (PMC13279823; doi:10.1038/s41467-026-72026-2)
Supplement: Supplementary file 1 — Supplementary Information [file 41467_2026_72026_MOESM1_ESM.pdf]

Supplementary Information

Cation-tuned acidic electrified interface for hydrogen peroxide electrosynthesis with industrial-level current densities in natural seawater

Peike Cao[1], Xuanchen Liu[1], Yanming Liu[1], Zihao Zhao[1], Shuo Chen[1], Hongtao Yu[1], Jingguang G. Chen[2]*, Xie Quan[1]*

[1] Key Laboratory of Industrial Ecology and Environmental Engineering (Ministry of Education, China), School of Environmental Science and Technology, Dalian University of Technology, Dalian, 116024, PR China.
[2] Department of Chemical Engineering, Columbia University, New York, NY, 10027, USA.
* Corresponding email: jgchen@columbia.edu; quanxie@dlut.edu.cn

This PDF file includes:

Supplementary Figs. 1 to 75
Supplementary Tables 1 to 13
Supplementary References 1 to 16

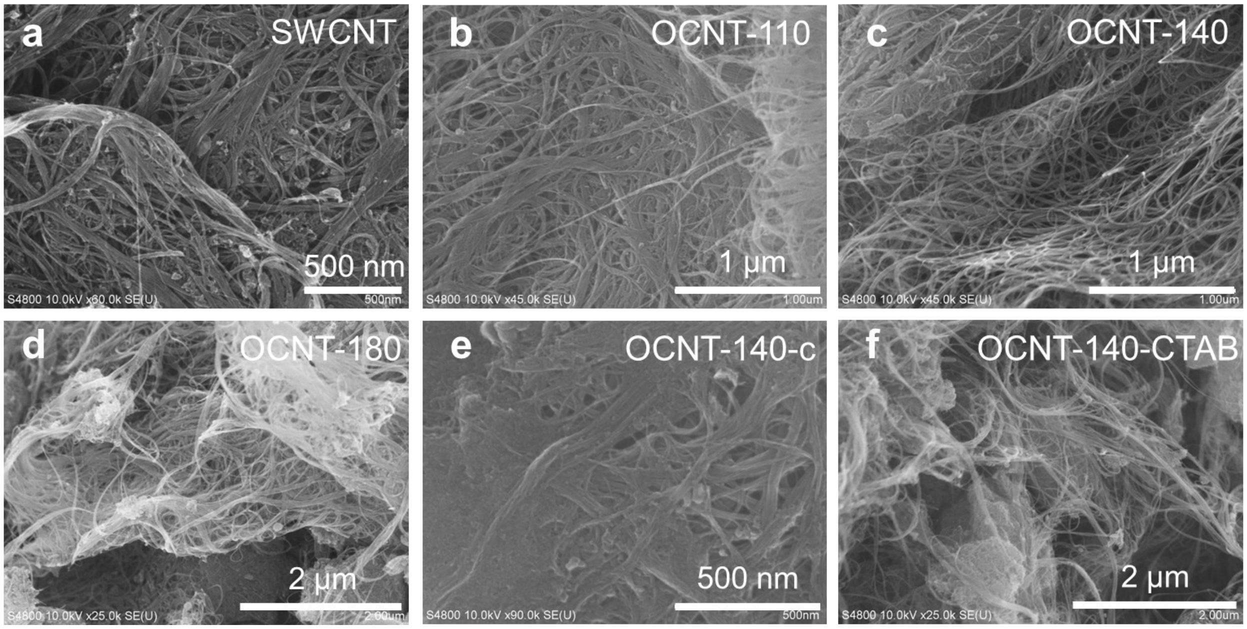

Supplementary Fig. 1 SEM images of CNTs before and after oxidation treatment. **a,** pristine SWCNT; **b-d,** oxidized OCNTs, namely OCNT-110, OCNT-140, and OCNT-180, were prepared at heating temperatures of 110°C, 140°C, and 180°C, respectively, using 27 wt% HNO_3. **e,** OCNT-140-c was synthesized using 67 wt% concentrated HNO_3. **f,** OCNT-140-CTAB was synthesized by loading CTAB onto OCNT-140.

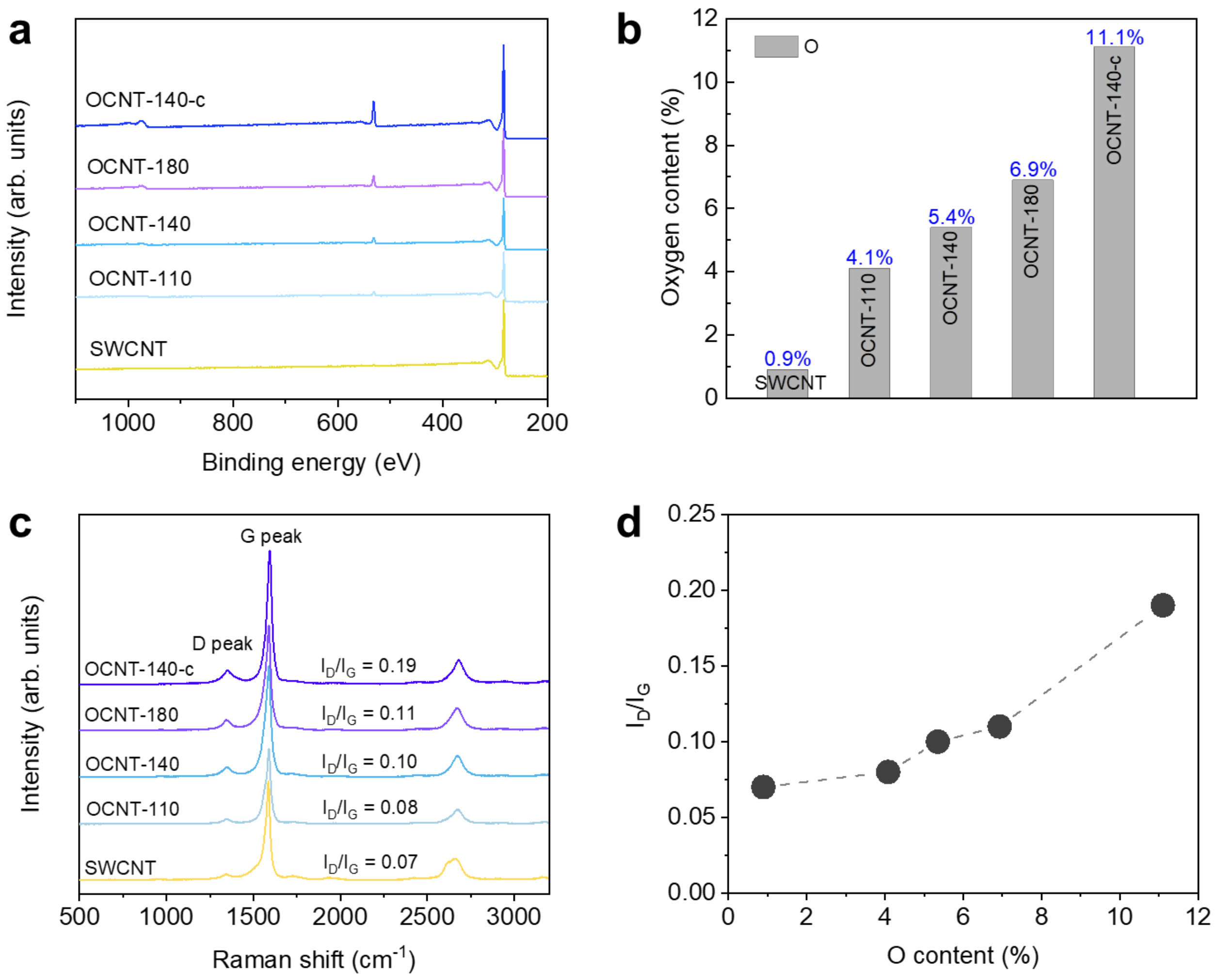

Supplementary Fig. 2 Oxygen doping and defects in CNTs before and after oxidation treatment. a, XPS spectra. **b,** Oxygen contents as quantified by XPS. **c,** Raman spectra with I_D/I_G values calculated from the ratio of the maximum intensities of the D and G peaks. **d,** Trend of I_D/I_G values as oxygen content increases. (Source data for Supplementary Fig. 2 are provided as a Source Data file.)

The D-band in Raman spectra is associated with defects and disordered carbon, while the G-band relates to C=C bonds in graphitized carbon.

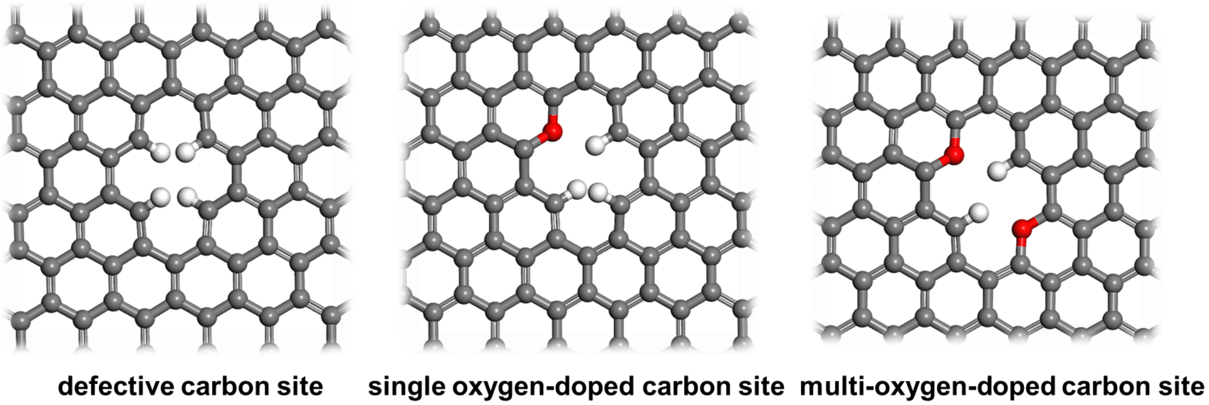

Supplementary Fig. 3 Schematic atomic models of defective carbon sites functionalized with oxygen groups. White, gray, and red spheres represent H, C and O atoms, respectively.

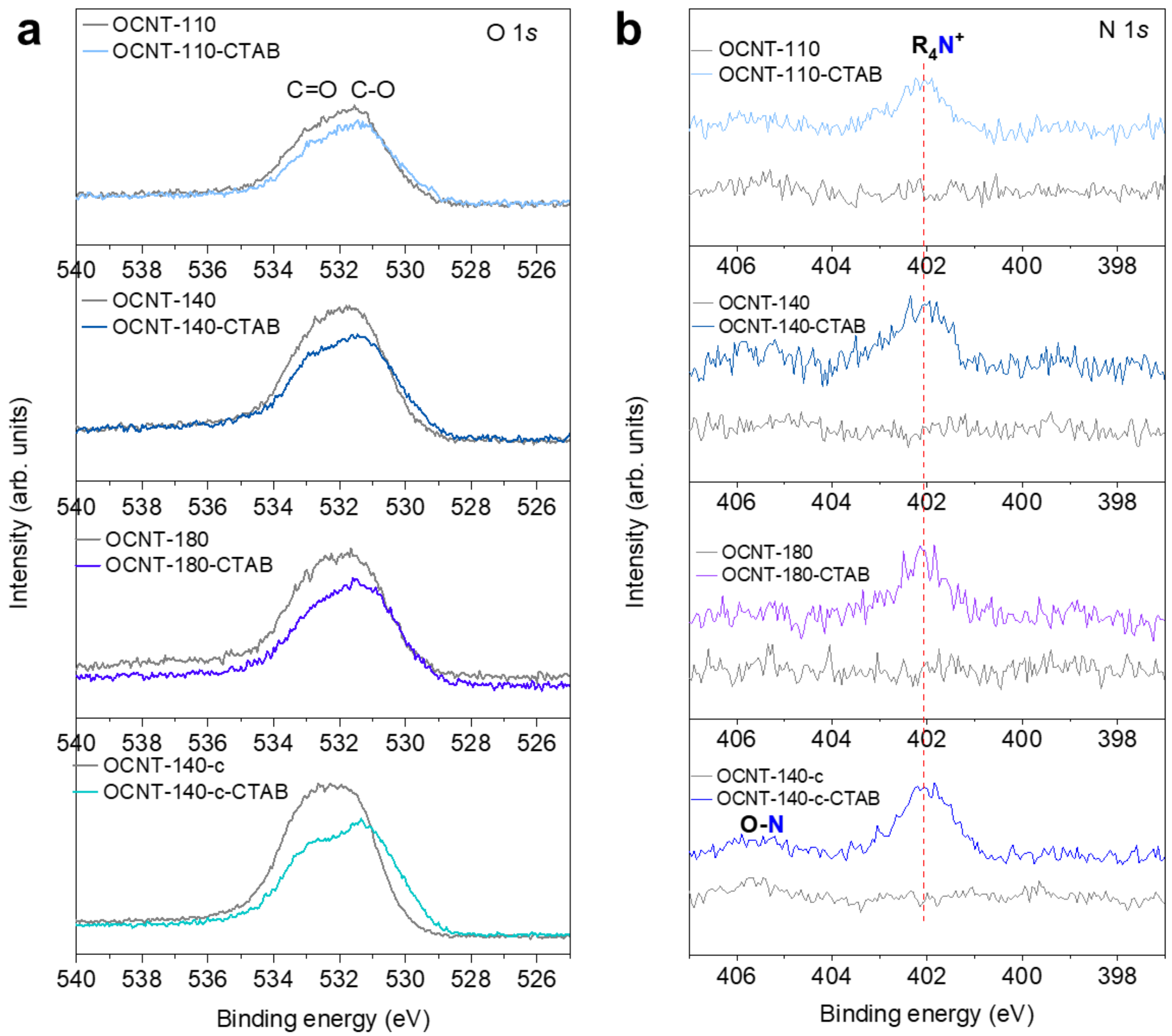

Supplementary Fig. 4 Analysis of oxygen and nitrogen-containing groups in OCNTs and OCNT-CTAB catalysts using high-resolution XPS spectra. **a,** O 1*s*; **b,** N 1*s*. (Source data for Supplementary Fig. 4 are provided as a Source Data file.)

The high-resolution O 1*s* spectra of OCNTs contained two sub-peaks, corresponding to C-O at 531.3 eV and C=O at 532.8 eV, indicating that the oxygen functional groups in the OCNTs primarily exist as C-O and C=O.

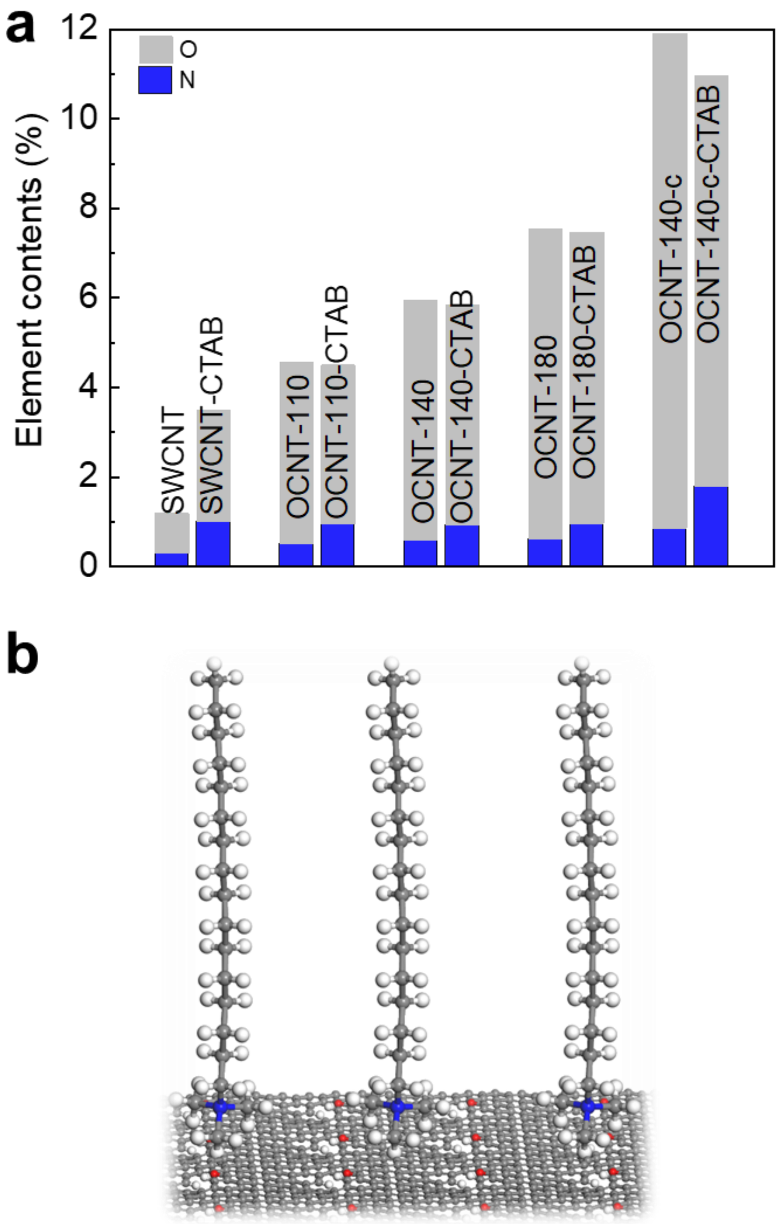

Supplementary Fig. 5 Characterization of oxygen and nitrogen contents in OCNTs and OCNTs-CTAB. **a,** Oxygen and nitrogen contents as quantified by XPS. **b,** Schematic structure of OCNT-140-CTAB. White, gray, red, and blue spheres represent H, C, O, and N atoms, respectively. (Source data for Supplementary Fig. 5 are provided as a Source Data file.)

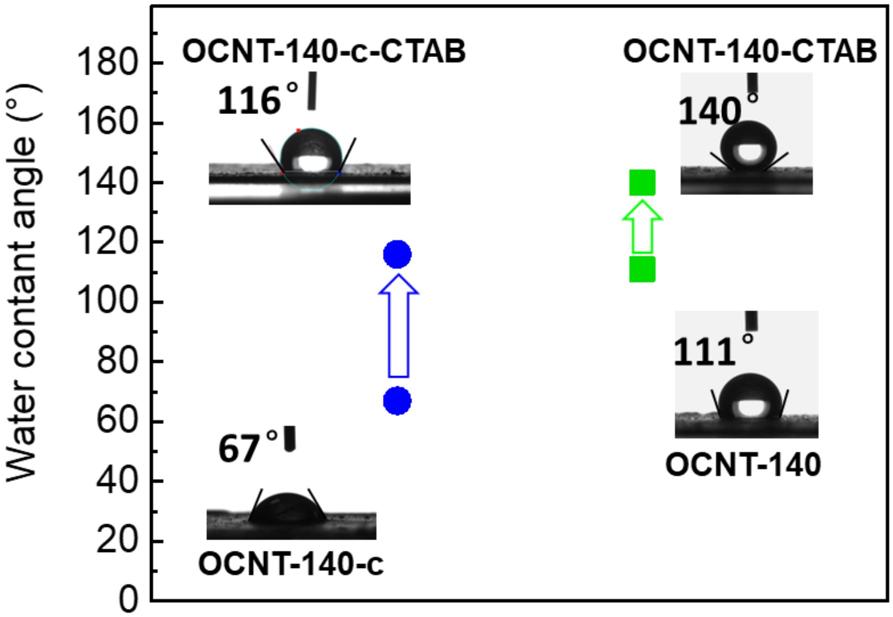

Supplementary Fig. 6 Water contact angles for the electrodes: OCNT-140 (also known as OCNT), OCNT-140-c, OCNT-140-CTAB (also referred to as OCNT-CTAB), and OCNT-140-c-CTAB. (Two independent water contact angles measurements were performed for each sample, and the average value was reported.)

Three types of interactions are considered for CTAB molecules and OCNT: (1) electrostatic attraction: positively charged cationic $-N^{+}(CH_3)_3$ moiety, which would spontaneously adsorb onto the negatively charged OCNT surface; (2) hydrophobic interactions: nonpolar molecules or regions avoiding water, often clustering together; (3) hydrogen bonds: stronger interactions typically involving hydrogen atoms bonded to electronegative atoms like oxygen or nitrogen.

a

b

c

d

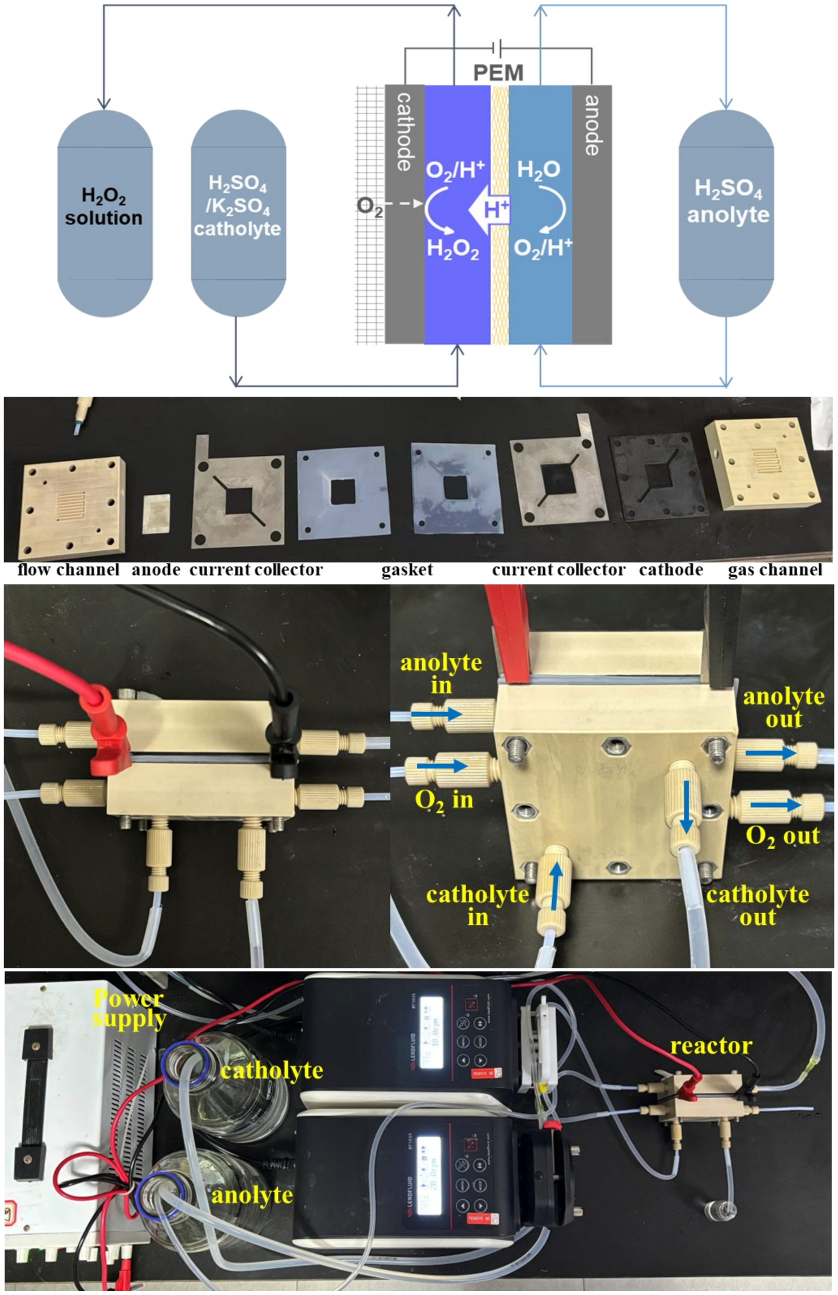

Supplementary Fig. 7 Illustration of the performance evaluation for acidic H_2O_2 electrosynthesis in a flow-type electrolytic reactor. **a,** Schematic featuring a gas-diffusion cathodic electrode and a platinum anode separated by a proton exchange membrane; **b-d,** Photos of electrolytic reactor and system.

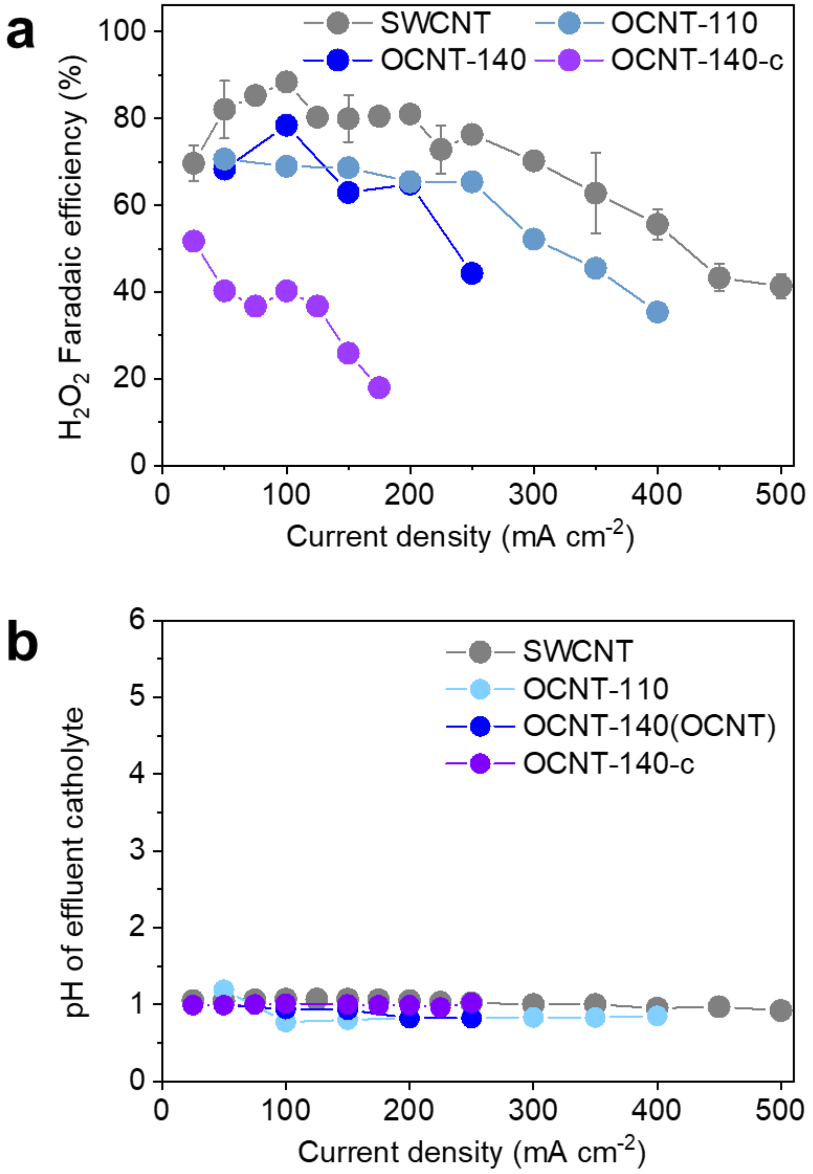

Supplementary Fig. 8 Faradaic efficiency of acidic H_2O_2 electrosynthesis using SWCNT and OCNTs electrodes. a, Faradaic efficiency for H_2O_2 production. **b,** pHs of the effluent catholyte. (Experimental conditions: cathode working area of 4 cm^2, 0.3 M K_2SO_4 in 0.1 M H_2SO_4 as the catholyte, 0.5 M H_2SO_4 as the anolyte, O_2 flow rate of 30 mL min^{-1}. The pH value was measured once for each point. Data for Faradaic efficiency represent the average of triplicate measurements, with error bars indicating standard deviations. Source data for Supplementary Fig. 8 are provided as a Source Data file.)

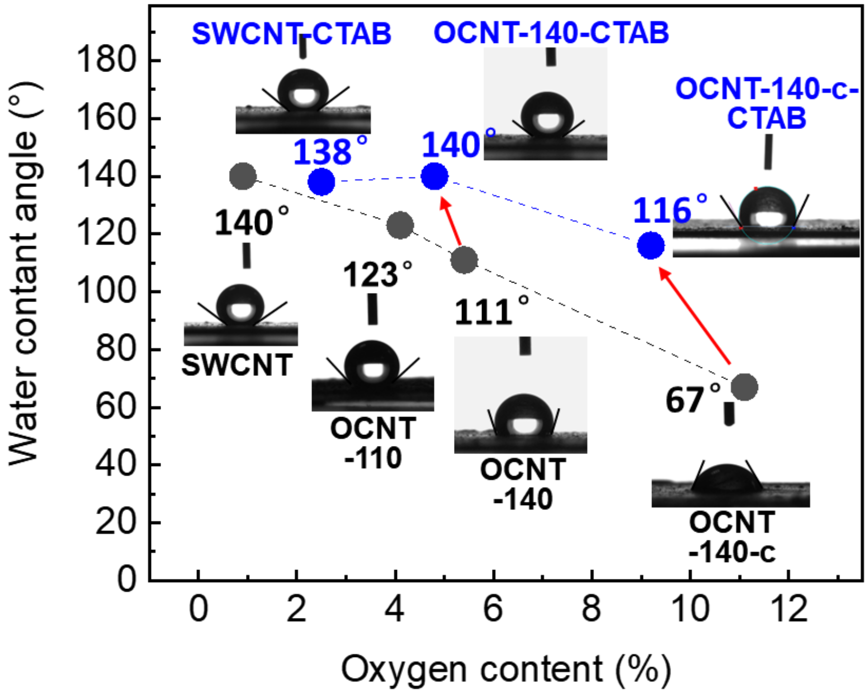

Supplementary Fig. 9 Water contact angles for the electrodes: SWCNT, OCNT-110, OCNT-140 (also known as OCNT), OCNT-140-c, SWCNT-CTAB, OCNT-140-CTAB (also referred to as OCNT-CTAB), and OCNT-140-c-CTAB. Two independent water contact angles measurements were performed for each sample, and the average value was reported.

The wettability of the electrode surface was characterized by contact angle measurements. When using water as the probe liquid, a larger contact angle indicates a more hydrophobic surface, while a smaller contact angle indicates a more hydrophilic surface.

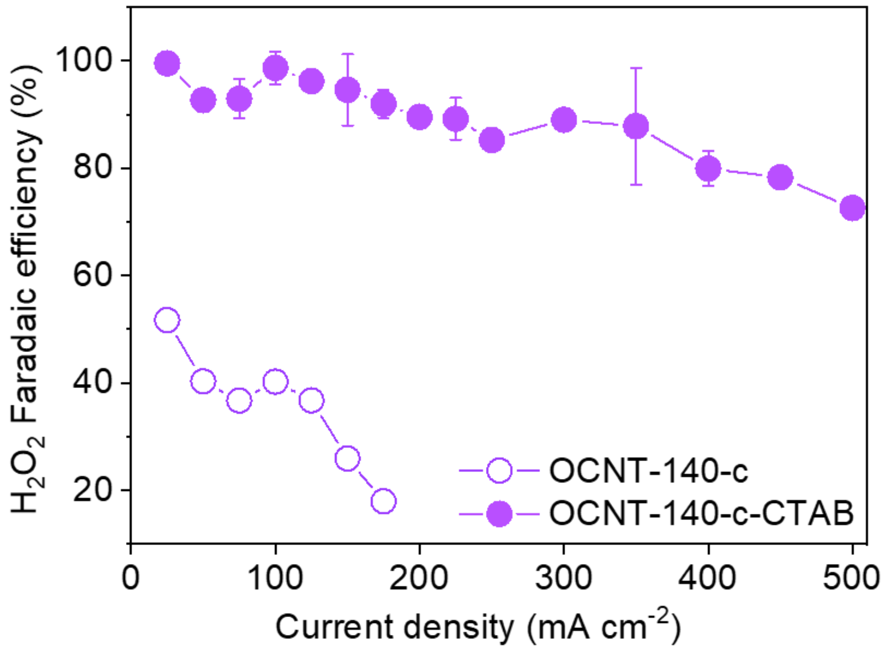

Supplementary Fig. 10 Faradaic efficiency of acidic H_2O_2 electrosynthesis using OCNT-140-c and OCNT-140-c-CTAB electrodes. (Experimental conditions: cathode working area of 4 cm^2, 0.3 M K_2SO_4 in 0.1 M H_2SO_4 as the catholyte, 0.5 M H_2SO_4 as the anolyte, O_2 flow rate of 30 mL min^{-1}. Data for Faradaic efficiency represent the average of triplicate measurements, with error bars indicating standard deviations. Source data for Supplementary Fig. 10 are provided as a Source Data file.)

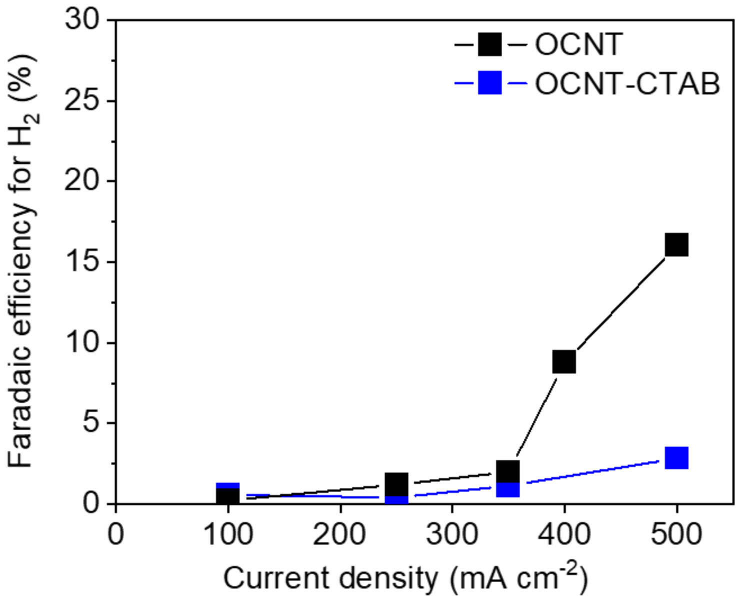

Supplementary Fig. 11 H_2 production efficiency for the OCNT and OCNT-CTAB catalysts at 50-500 mA cm^{-2}. (Experimental conditions: cathode working area of 4 cm^2, 0.3 M K_2SO_4 in 0.1 M H_2SO_4 as the catholyte, 0.5 M H_2SO_4 as the anolyte, O_2 flow rate of 30 mL min^{-1}. H_2 concentration was measured once for each point. The pH value was measured once for each point. Source data for Supplementary Fig. 11 are provided as a Source Data file.)

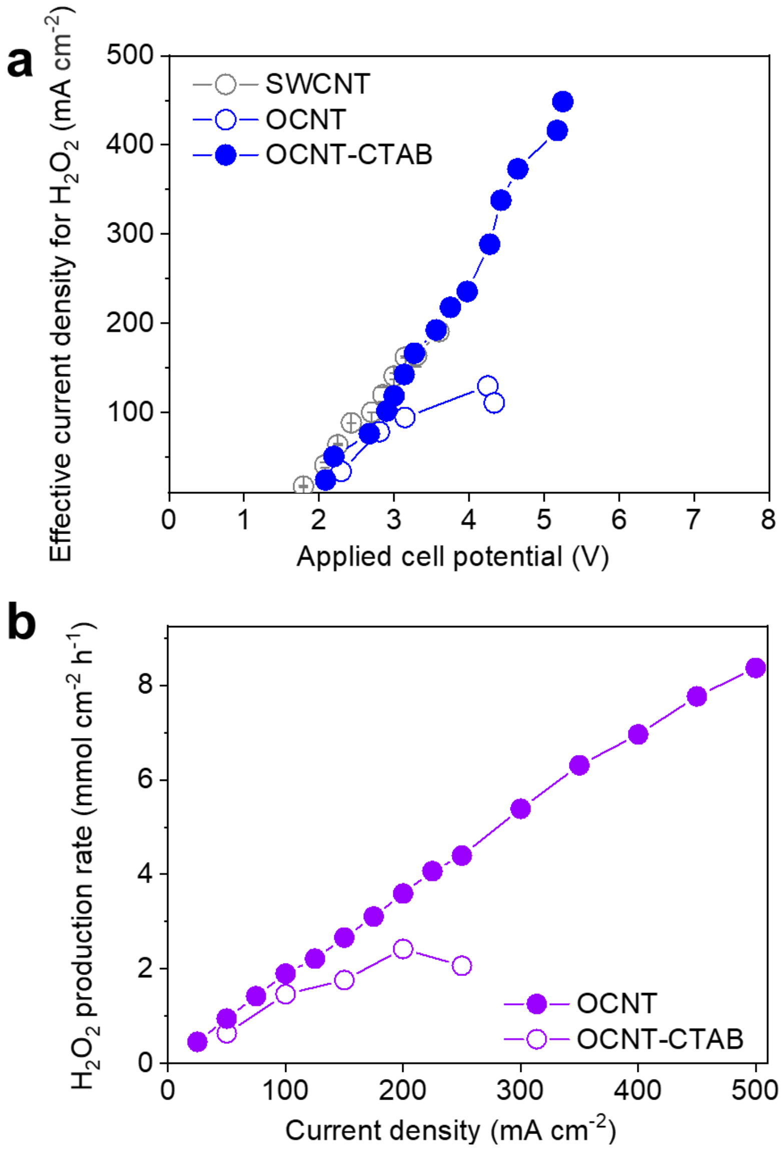

Supplementary Fig. 12 Performance comparison on SWCNT, OCNT, and OCNT-CTAB catalysts. a, Effective current as a function of the applied cell potential for acidic H_2O_2 electrosynthesis. **b,** H_2O_2 production rate. (Experimental conditions: cathode working area of 4 cm^2, 0.3 M K_2SO_4 in 0.1 M H_2SO_4 as the catholyte, 0.5 M H_2SO_4 as the anolyte, O_2 flow rate of 30 mL min^{-1}. Data for effective current density and H_2O_2 production rate represent the average of triplicate measurements. The cell potential was recorded as the average of two or three measurements. Source data for Supplementary Fig. 12 are provided as a Source Data file.)

The results highlighted enhanced effective currents for H_2O_2 production on the OCNT-CTAB catalyst compared to both SWCNT and OCNT catalysts under varying applied cell potentials. This suggests that the CTAB modification markedly promotes higher efficiency in H_2O_2 production.

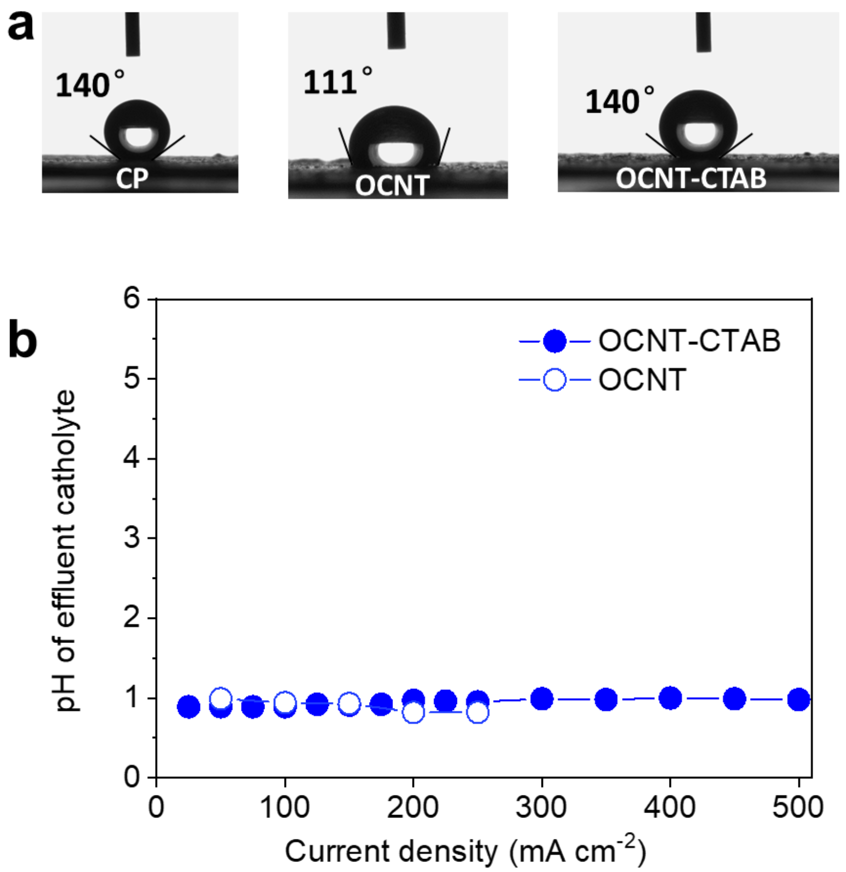

Supplementary Fig. 13 Evaluation of acidic H_2O_2 electrosynthesis. a, Water contact angles of the CP (bare carbon paper substrate), OCNT (OCNT-140) and OCNT-CTAB (OCNT-140-CTAB) electrodes. **b,** pHs of the effluent catholyte. (Experimental conditions: cathode working area of 4 cm^2, 0.3 M K_2SO_4 in 0.1 M H_2SO_4 as the catholyte, 0.5 M H_2SO_4 as the anolyte, O_2 flow rate of 30 mL min^{-1}. Two independent water contact angles measurements were performed for each sample, and the average value was reported. The pH value was measured once for each point. Source data for Supplementary Fig. 13 are provided as a Source Data file.)

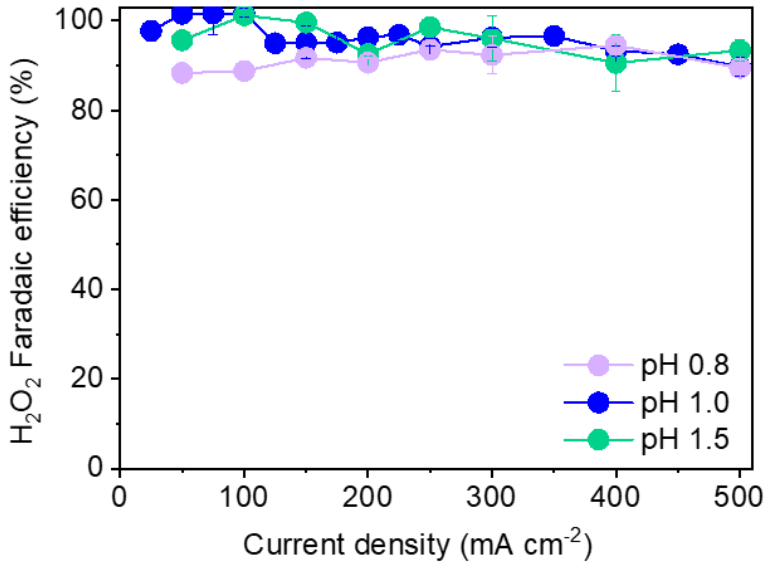

Supplementary Fig. 14 Effect of the catholyte pH on acidic H_2O_2 electrosynthesis using OCNT-CTAB electrode. (Experimental conditions: cathode working area of 4 cm^2, 0.3 M K_2SO_4 in 0.1 M H_2SO_4 as the catholyte, 0.5 M H_2SO_4 as the anolyte, O_2 flow rate of 30 mL min^{-1}. Data for Faradaic efficiency represent the average of triplicate measurements, with error bars indicating standard deviations. Data for pH values represent the average of triplicate measurements. Source data for Supplementary Fig. 14 are provided as a Source Data file.)

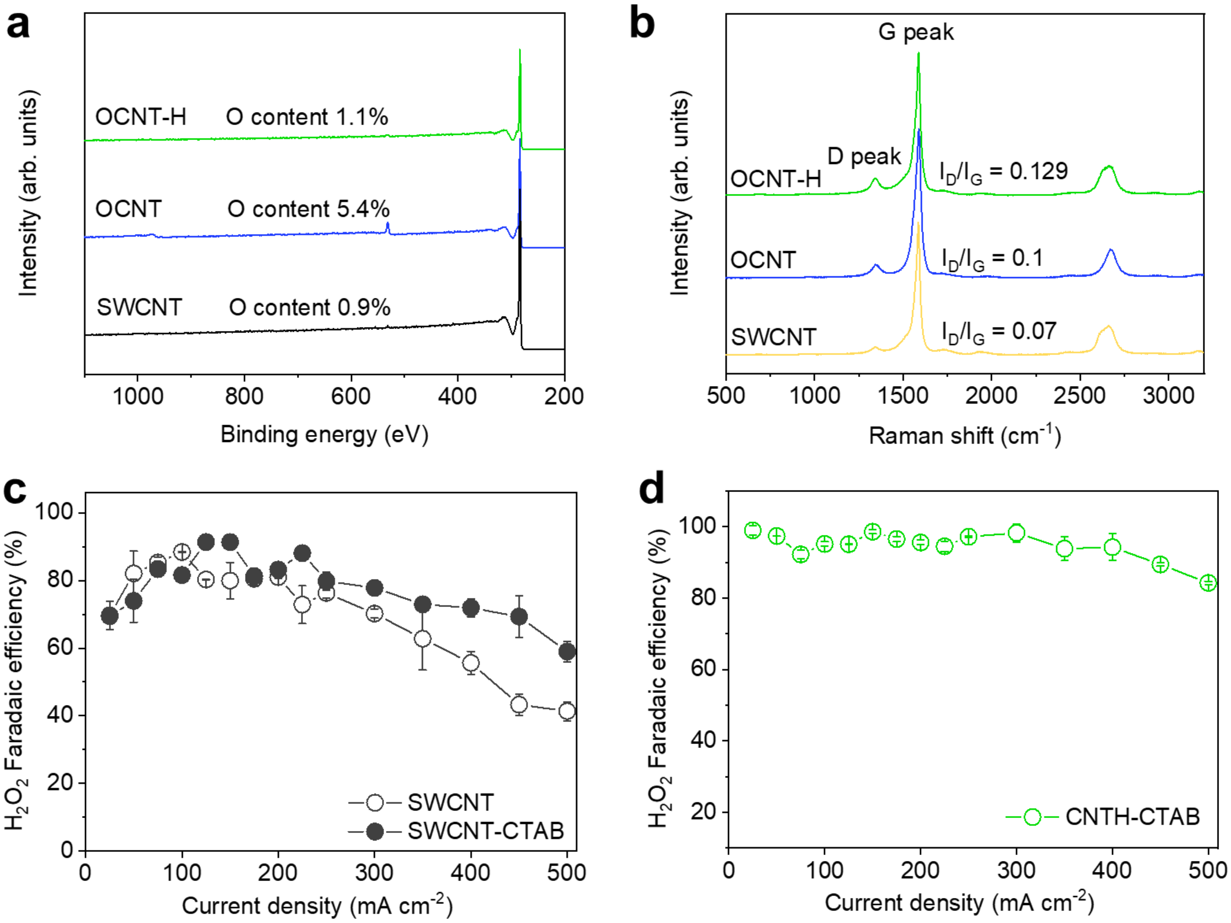

Supplementary Fig. 15 Evaluation of the OCNT-H catalyst prepared by removing oxygen groups through heat treatment in a reductive H_2 atmosphere. a, XPS spectra. **b,** Raman spectra. **c-d,** Faradaic efficiency of acidic H_2O_2 electrosynthesis in 0.1 M H_2SO_4 with 0.3 M K_2SO_4 catholyte and 0.5 M H_2SO_4 anolyte. (Experimental conditions: cathode working area of 4 cm^2, 0.3 M K_2SO_4 in 0.1 M H_2SO_4 as the catholyte, 0.5 M H_2SO_4 as the anolyte, O_2 flow rate of 30 mL min^{-1}. Data for Faradaic efficiency represent the average of triplicate measurements, with error bars indicating standard deviations. Source data for Supplementary Fig. 15 are provided as a Source Data file.)

XPS analysis indicated that after hydrogen treatment, the oxygen content of the OCNT-H catalyst was reduced to 1.1%, which is close to that of the original, unoxidized SWCNT (0.9%). In Raman spectroscopy, I_D represents signals associated with carbon defects (such as carbon atom vacancies and oxygen groups), while I_G corresponds to the signal for intact graphite vibrations. The ratio of I_D/I_G serves as an indicator of defect degree: a higher value indicates a greater defect density. The results revealed that the defect density of the OCNT-H catalyst is significantly higher than that of the SWCNT, and even exceeds the defect density of the oxygenated OCNT.

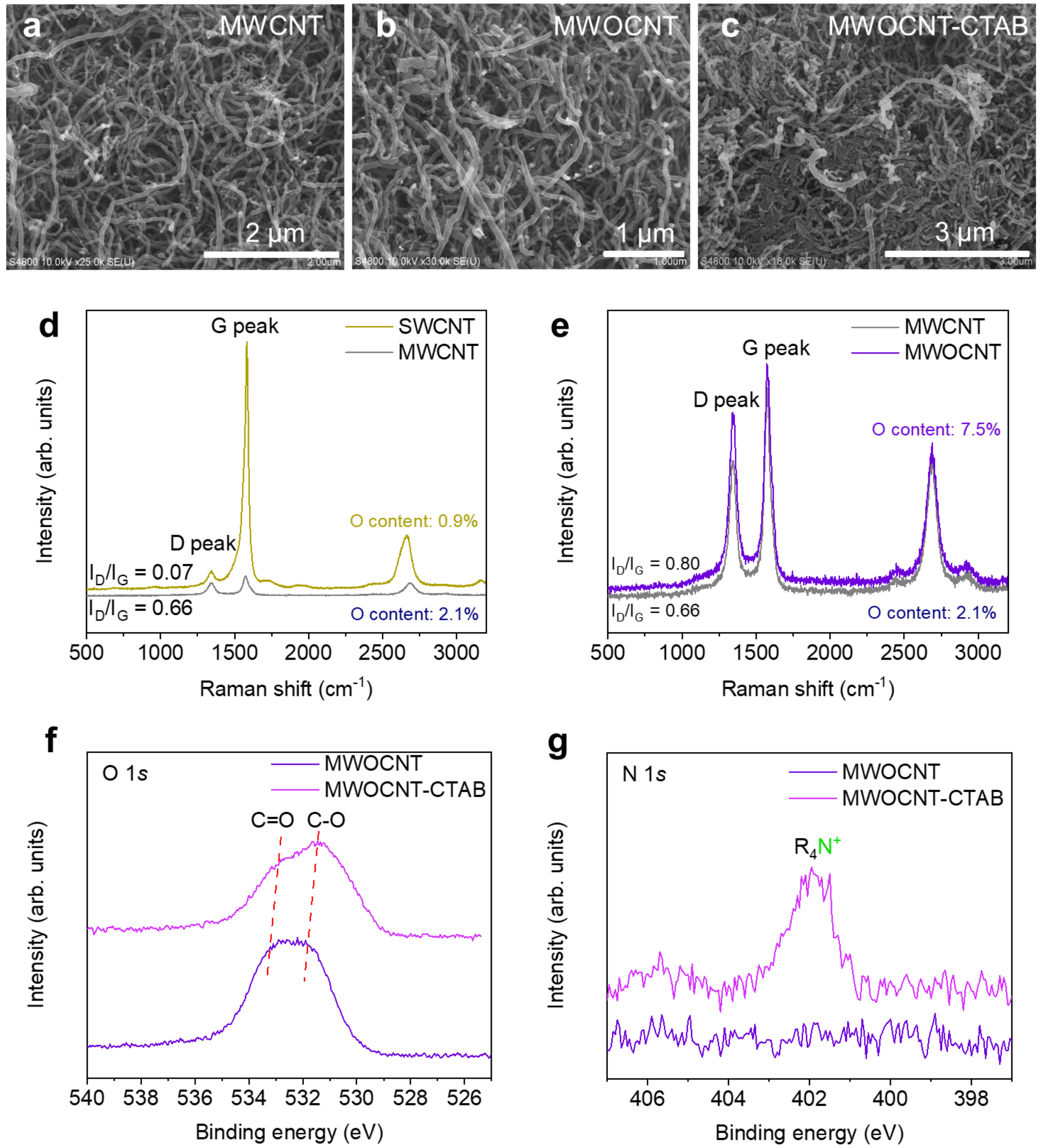

Supplementary Fig. 16 Characterization of pristine MWCNT and oxidized MWOCNT, achieved by heating at 140 °C using 67 wt% concentrated HNO_3. a-c, SEM images of MWCNT, MWOCNT, and MWOCNT-CTAB. **d-e,** Raman spectra (O contents of MWCNT and MWOCNT were measured by XPS). **f-g,** O 1*s* and N 1*s* XPS high-resolution spectra. Source data for Supplementary Fig. 16 are provided as a Source Data file.

Multi-walled carbon nanotubes (MWCNTs) exhibit a significantly stronger D peak intensity compared to SWCNTs, indicating a higher defect density in MWCNTs. Despite this, MWCNTs possess a more stable structure than SWCNTs. This inherent stability leads to a reduced introduction of oxygen functional groups onto MWCNTs (oxygen content: 7.5% for MWOCNT *vs* 11.1% for OCNT-140-c) when subjected to the same oxidation method, thereby contributing to their distinct Raman characteristics.

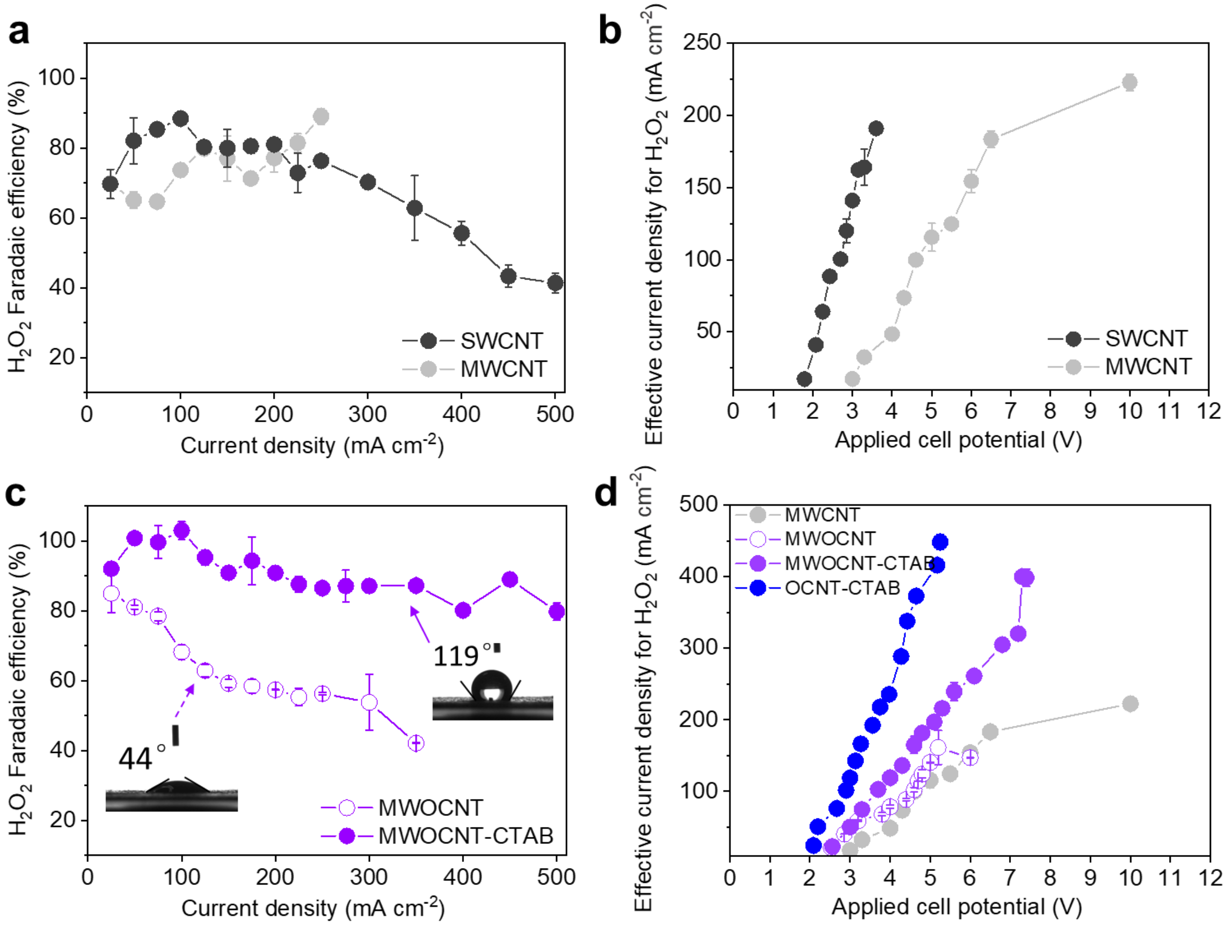

Supplementary Fig. 17 Performance evaluation of MWCNT, MWOCNT and MWOCNT-CTAB electrodes for acidic H_2O_2 electrosynthesis. **a-b,** SWCNT and MWCNT electrodes. **c-d,** MWOCNT and MWOCNT-CTAB. (Experimental conditions: cathode working area of 4 cm^2, 0.3 M K_2SO_4 in 0.1 M H_2SO_4 as the catholyte, 0.5 M H_2SO_4 as the anolyte, O_2 flow rate of 30 mL min^{-1}. Data for Faradaic efficiency represent the average of triplicate measurements, with error bars indicating standard deviations. Data for effective current density represent the average of triplicate measurements. Source data for Supplementary Fig. 17 are provided as a Source Data file.)

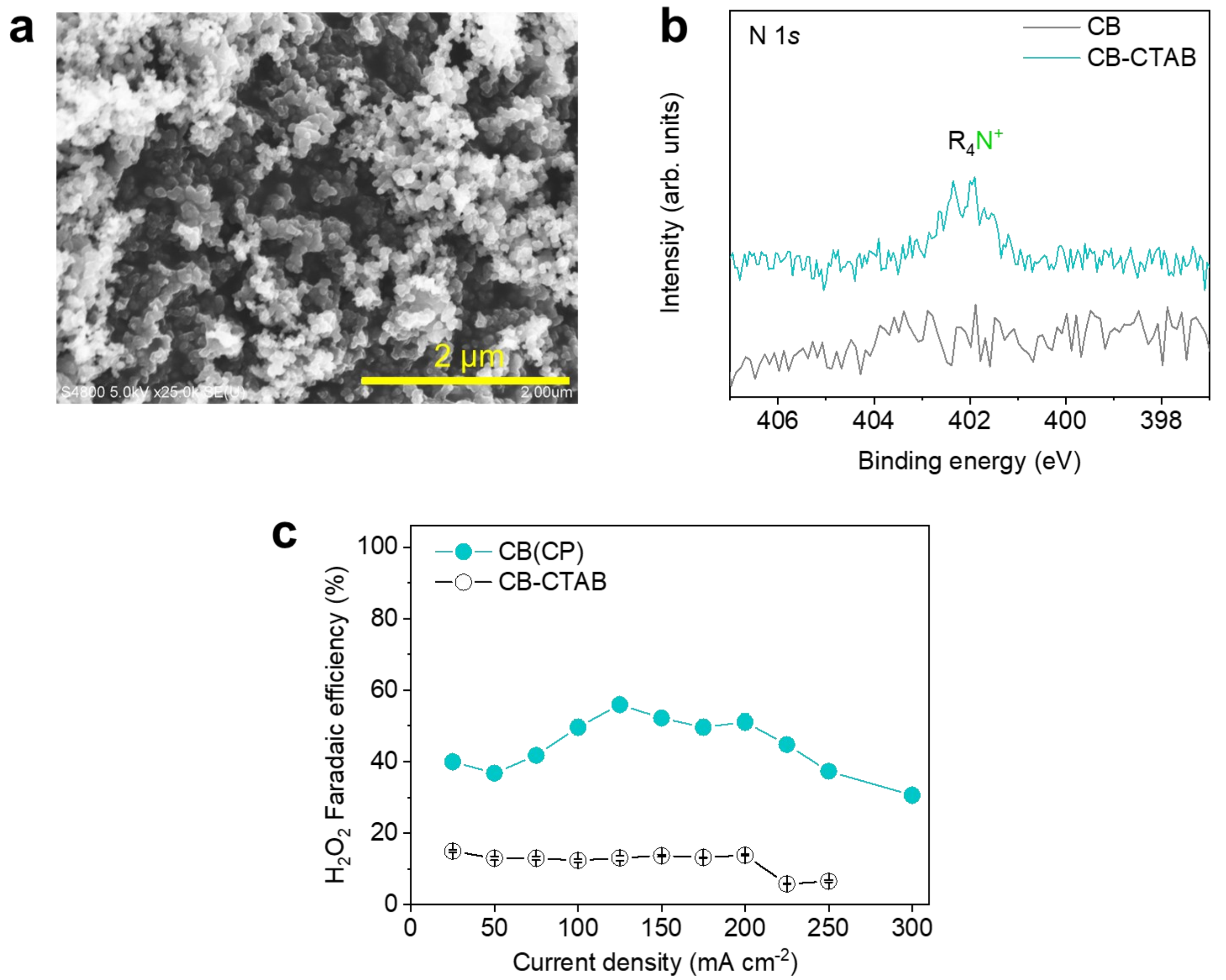

Supplementary Fig. 18 Evaluation of the CB and CB-CTAB electrodes. **a,** SEM image of CB-CTAB. **b,** N 1*s* XPS high-resolution spectra. **c,** H_2O_2 Faradaic efficiency of acidic H_2O_2 electrosynthesis. (Experimental conditions: cathode working area of 4 cm^2, 0.3 M K_2SO_4 in 0.1 M H_2SO_4 as the catholyte, 0.5 M H_2SO_4 as the anolyte, O_2 flow rate of 30 mL min^{-1}. Data for Faradaic efficiency represent the average of triplicate measurements, with error bars indicating standard deviations. Source data for Supplementary Fig. 18 are provided as a Source Data file.)

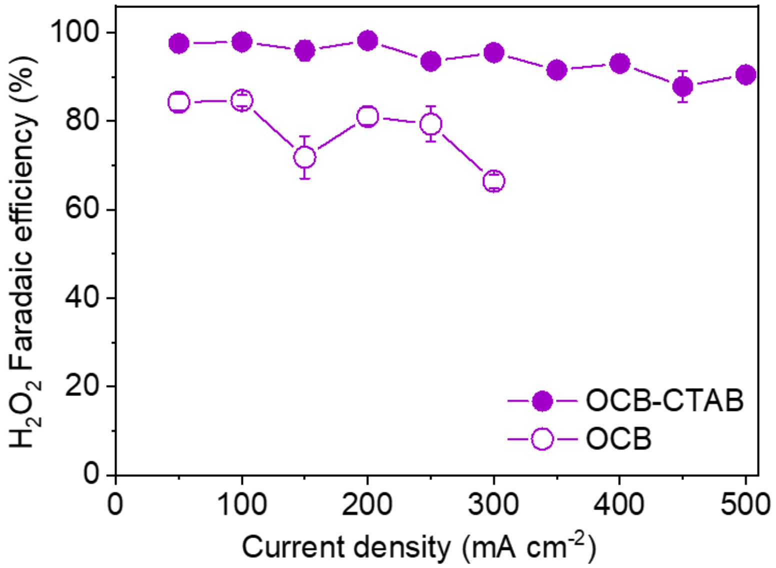

Supplementary Fig. 19 Performance evaluation of OCB and OCB-CTAB electrodes for acidic H_2O_2 electrosynthesis. (Experimental conditions: cathode working area of 4 cm^2, 0.3 M K_2SO_4 in 0.1 M H_2SO_4 as the catholyte, 0.5 M H_2SO_4 as the anolyte, O_2 flow rate of 30 mL min^{-1}. Data for Faradaic efficiency represent the average of triplicate measurements, with error bars indicating standard deviations. Source data for Supplementary Fig. 19 are provided as a Source Data file.)

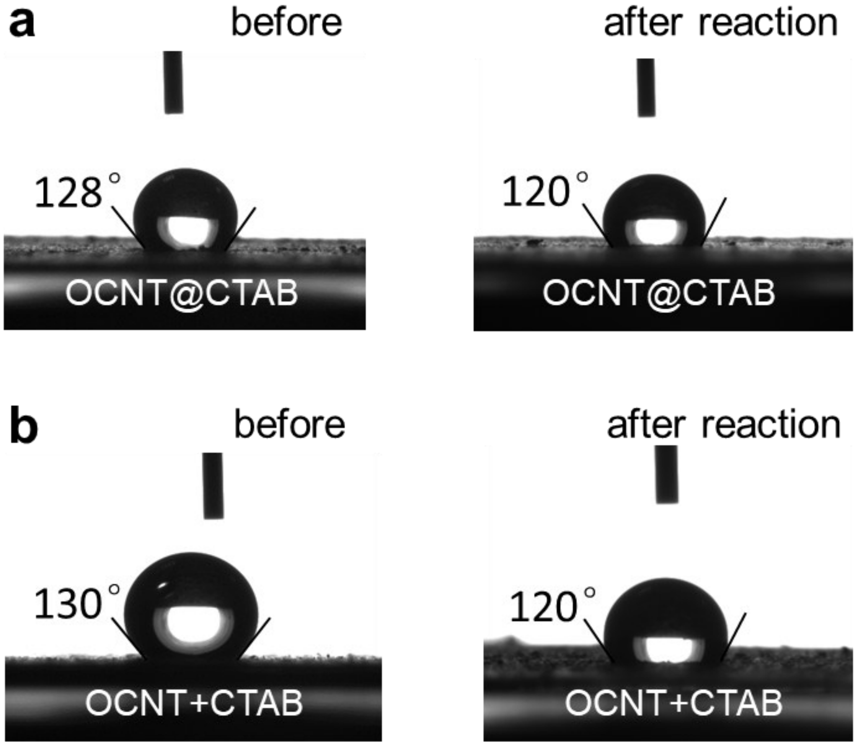

Supplementary Fig. 20 Water contact angles of on the surfaces of CTAB-modified OCNT electrodes. a, OCNT@CTAB electrode by coating CTAB on the electrode surface. **b,** Adding of CTAB into the OCNT catalyst ink. (Two independent water contact angles measurements were performed for each sample, and the average value was reported.)

OCNT@CTAB and OCNT+CTAB refer to the CTAB coating on the electrode surface and the addition of CTAB into the OCNT catalyst ink, respectively. Altering the preparation method changes the interaction between CTAB and OCNT. This modification leads to a significant decline in performance, even falling below that of the OCNT electrode without CTAB, despite the hydrophobicity remaining high.

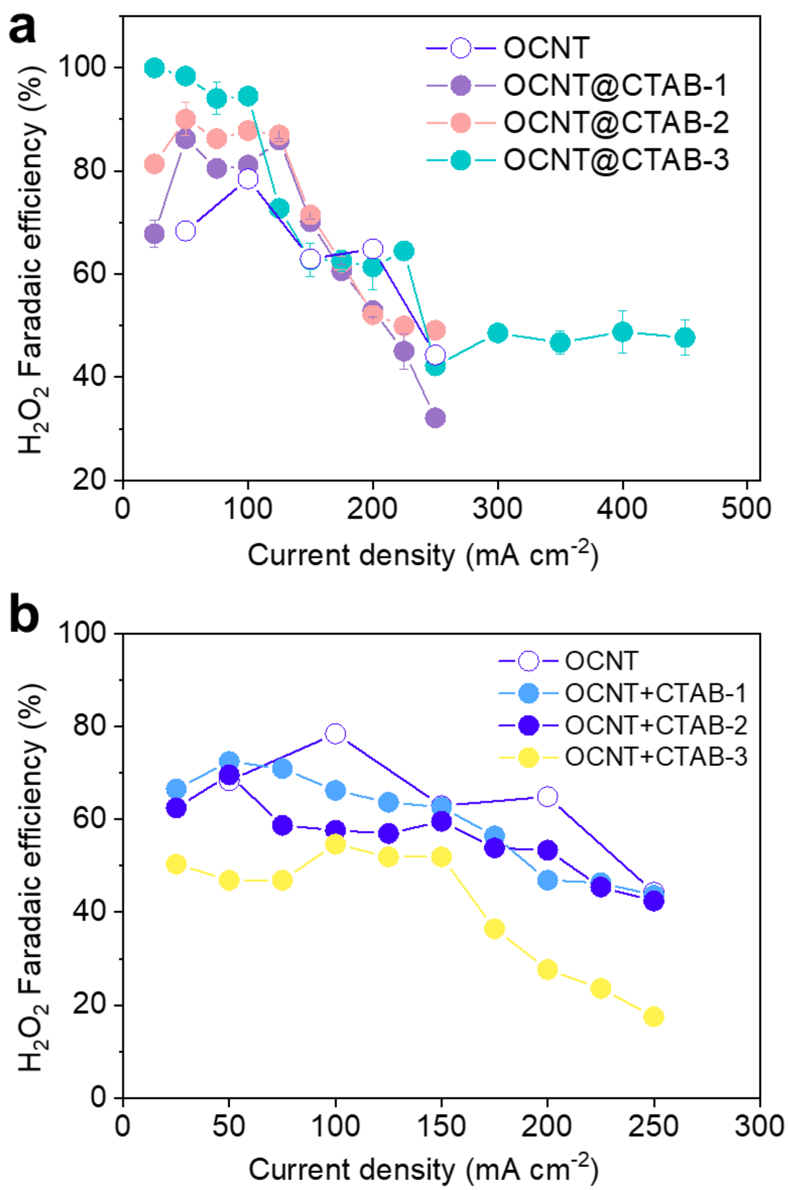

Supplementary Fig. 21 H_2O_2 Faradaic efficiency of the OCNT@CTAB and OCNT+CTAB electrodes for acidic H_2O_2 electrosynthesis. **a,** OCNT@CTAB-1, OCNT@CTAB-2, and OCNT@CTAB-3 present the CTAB loadings of 14, 28 and 55 μg cm^{-2} using the method of coating the CTAB into the electrode surface. **b,** OCNT+CTAB-1, OCNT+CTAB-2, and OCNT+CTAB-3 present the CTAB loadings of 14, 28 and 55 μg cm^{-2} using the method of adding the CTAB into the catalyst ink. (Experimental conditions: cathode working area of 4 cm^2, 0.3 M K_2SO_4 in 0.1 M H_2SO_4 as the catholyte, 0.5 M H_2SO_4 as the anolyte, O_2 flow rate of 30 mL min^{-1}. Data for Faradaic efficiency represent the average of triplicate measurements, with error bars indicating standard deviations. Source data for Supplementary Fig. 21 are provided as a Source Data file.)

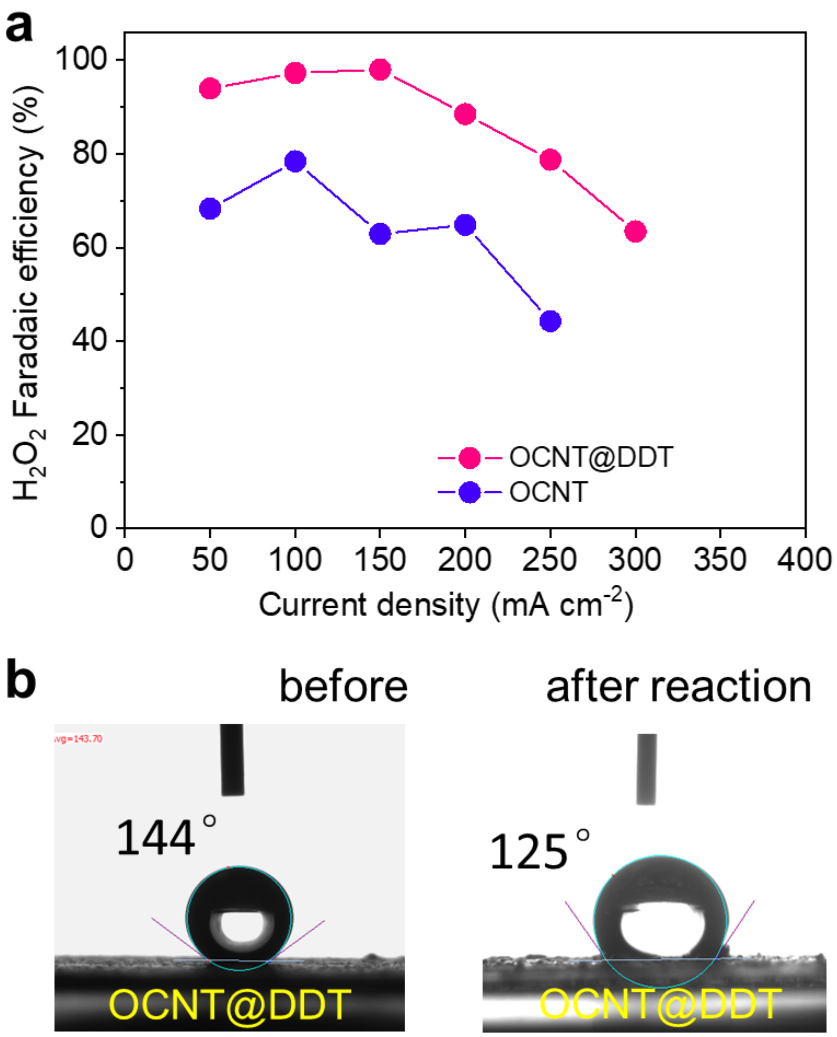

Supplementary Fig. 22 Performance evaluation of acidic H_2O_2 electrosynthesis using OCNT@DDT and OCNT electrodes. a, Faradaic efficiency for H_2O_2 production. **b,** Water contact angles of the OCNT@DDT electrode before and after reaction. (Experimental conditions: cathode working area of 4 cm^2, 0.3 M K_2SO_4 in 0.1 M H_2SO_4 as the catholyte, 0.5 M H_2SO_4 as the anolyte, O_2 flow rate of 30 mL min^{-1}. Data for Faradaic efficiency represent the average of triplicate measurements, with error bars indicating standard deviations. Source data for Supplementary Fig. 22 are provided as a Source Data file.)

OCNT@DDT was prepared by coating dodecanethiol (DDT) onto the OCNT electrode surface.

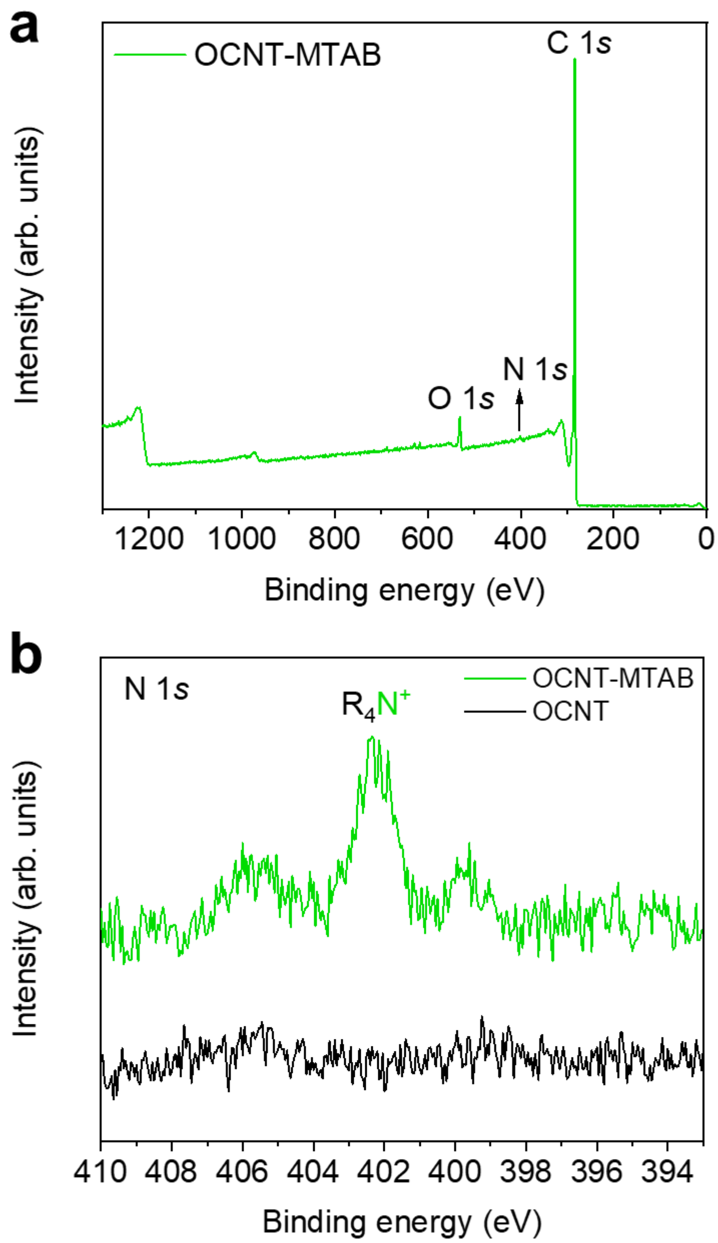

Supplementary Fig. 23 XPS of OCNT-MTAB catalyst. a, XPS survey spectra. **b,** High-resolution N 1*s* spectrum. (Source data for Supplementary Fig. 23 are provided as a Source Data file.)

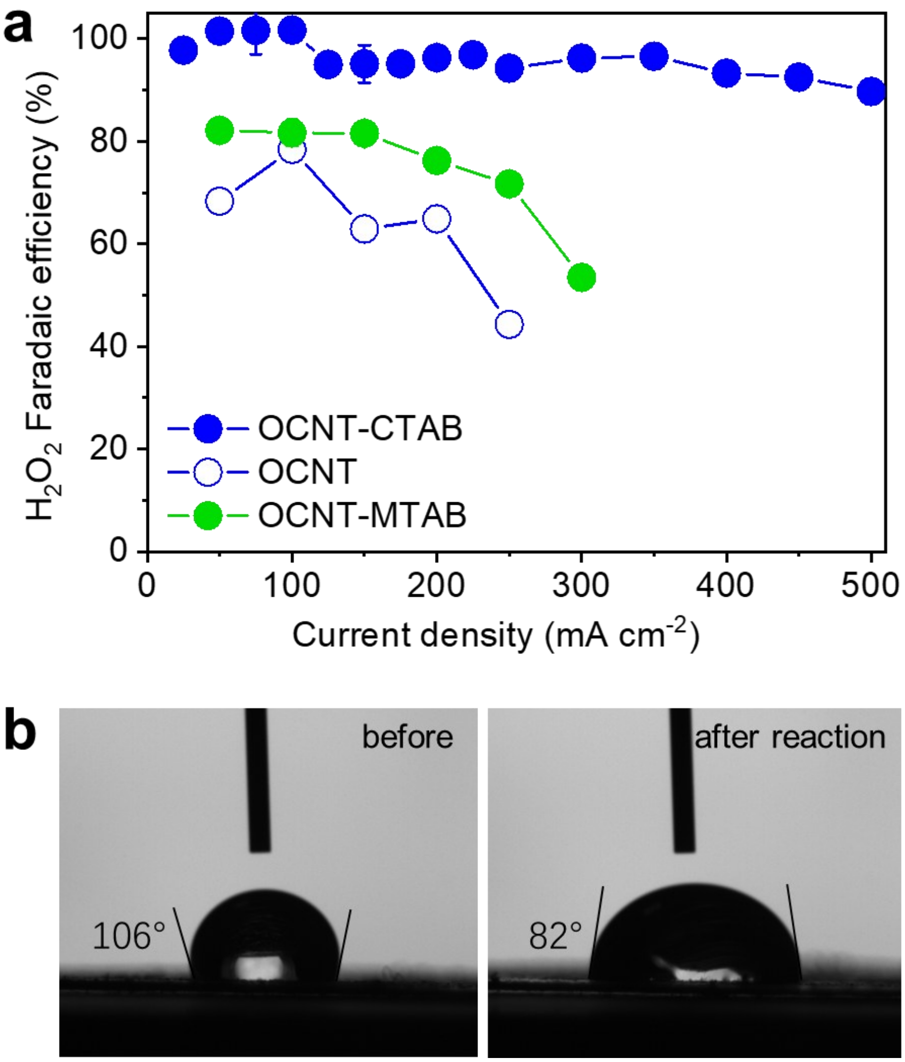

Supplementary Fig. 24 Performance evaluation of acidic H_2O_2 electrosynthesis using OCNT-MTAB electrode. **a,** Faradaic efficiency for H_2O_2 production. **b,** Water contact angles of the OCNT-MTAB electrode before and after reaction. (Experimental conditions: cathode working area of 4 cm^2, 0.3 M K_2SO_4 in 0.1 M H_2SO_4 as the catholyte, 0.5 M H_2SO_4 as the anolyte, O_2 flow rate of 30 mL min^{-1}. Data for Faradaic efficiency represent the average of triplicate measurements, with error bars indicating standard deviations. Source data for Supplementary Fig. 24 are provided as a Source Data file.)

OCNT-MTAB refers to a short-chain cationic surfactant (tetramethylammonium bromide, MTAB) modified OCNT catalyst.

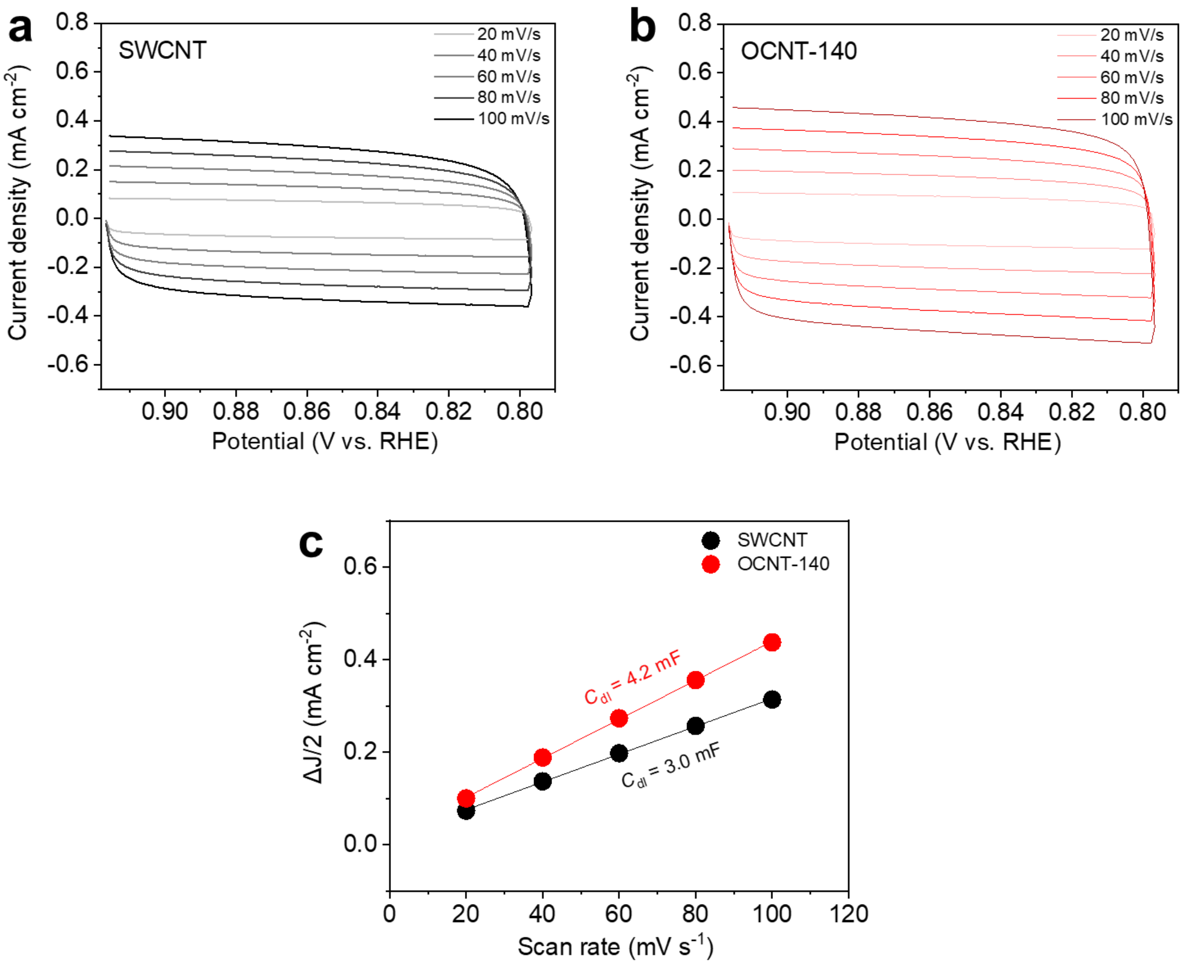

Supplementary Fig. 25 Electrochemical surface area evaluation of catalysts. a-b, Cyclic voltammetry curves of SWCNT and OCNT-140 catalysts at scan rates of 20, 40, 60, 80, and 100 mV s^{-1}. **c,** Linear fitting of the current density at 0.86 V vs. RHE against scan rate for estimating the double-layer capacitance of the catalysts. (Experimental conditions: catalyst loading of 0.5 mg cm^{-2}, 0.3 M K_2SO_4 in 0.1 M H_2SO_4 as the electrolyte. The reported potentials are not *iR*-corrected. Source data for Supplementary Fig. 25 are provided as a Source Data file.)

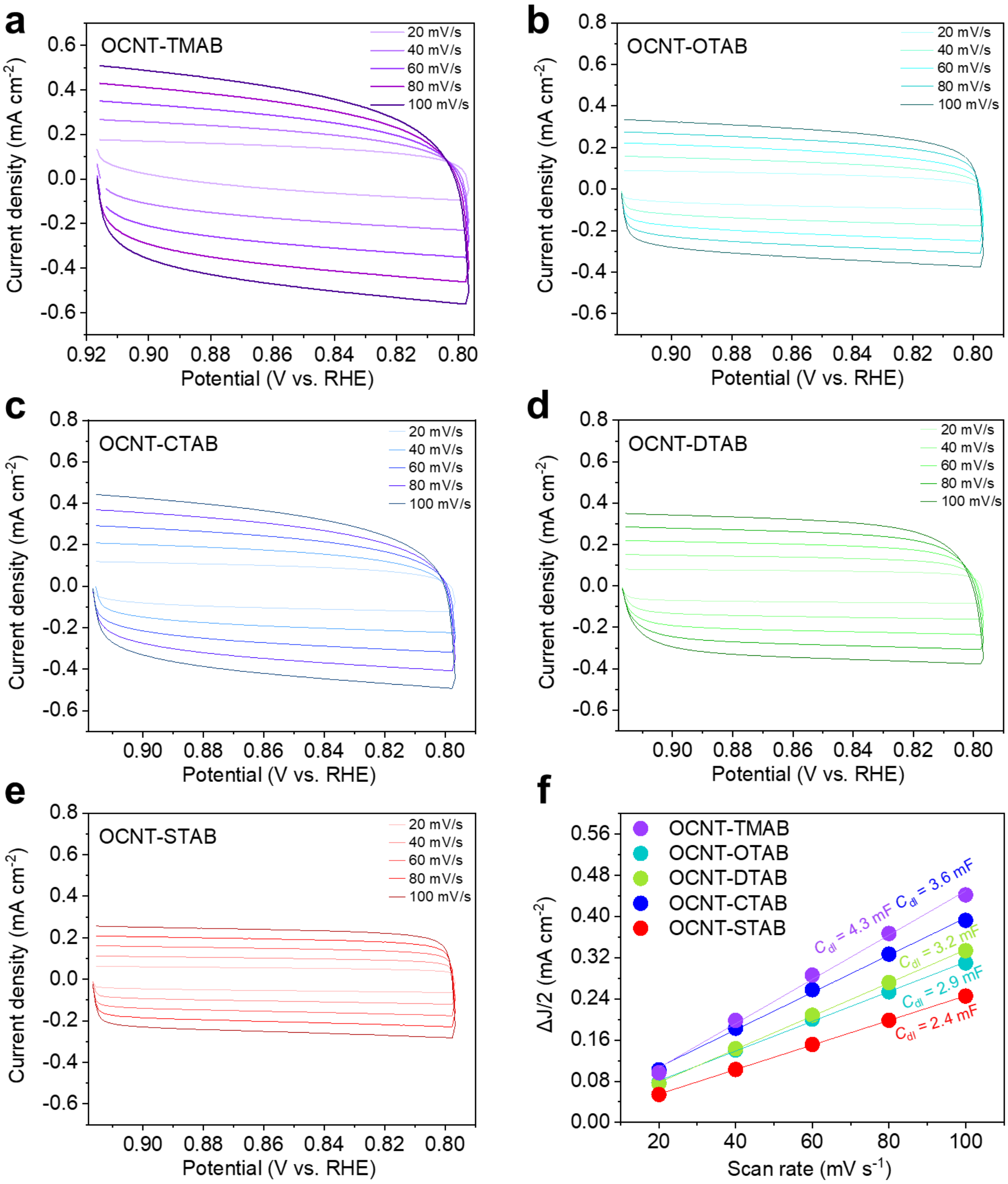

Supplementary Fig. 26 Electrochemical surface area evaluation of catalysts. a-e, Cyclic voltammetry curves of OCNT-TMAB, OCNT-OTAB, OCNT-CTAB, OCNT-DTAB, and OCNT-STAB catalysts at scan rates of 20, 40, 60, 80, and 100 mV s^{-1}. **f,** Linear fitting of the current density at 0.86 V vs. RHE against scan rate for estimating the double-layer capacitance of the catalysts. (Experimental conditions: catalyst loading of 0.5 mg cm^{-2}, 0.3 M K_2SO_4 in 0.1 M H_2SO_4 as the electrolyte. The reported potentials are not *iR*-corrected. Source data for Supplementary Fig. 26 are provided as a Source Data file.)

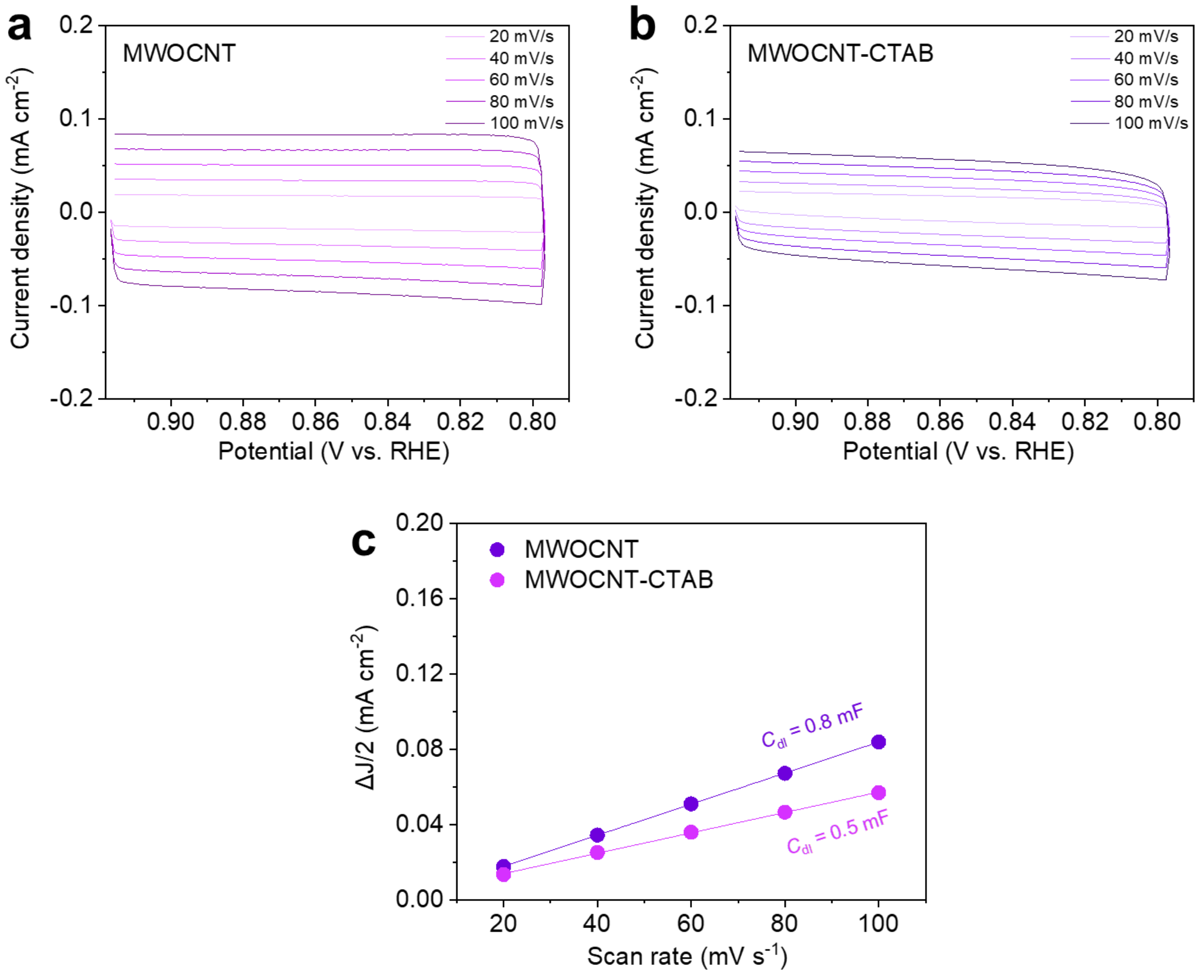

Supplementary Fig. 27 Electrochemical surface area evaluation of catalysts. a-b, Cyclic voltammetry curves of MWOCNT and MWOCNT-CTAB catalysts at scan rates of 20, 40, 60, 80, and 100 mV s^{-1}. **c,** Linear fitting of the current density at 0.86 V vs. RHE against scan rate for estimating the double-layer capacitance of the catalysts. (Experimental conditions: catalyst loading of 0.5 mg cm^{-2}, 0.3 M K_2SO_4 in 0.1 M H_2SO_4 as the electrolyte. The reported potentials are not *iR*-corrected. Source data for Supplementary Fig. 27 are provided as a Source Data file.)

To evaluate the electronic effect of cationic modifier, we measured the electrochemical double-layer capacitance to evaluate the electrochemically active surface. Results indicated that oxidation enhanced the SWCNT capacitance from 3.0 to 4.2 mF cm^{-2} (**Supplementary Fig. 25**), which can be attributed to the higher oxygen content in the OCNT that provides additional electrochemical active sites and thus improves charge storage. Modification with the short-chain cationic MTAB preserved the capacitance of OCNT (**Supplementary Fig. 26**), however, long-chain cationic modifiers lowered the capacitance, likely by interacting with oxygen sites and inhibiting electron storage. This effect from cationic polymer modification that led to a substantial capacitance reduction was similarly observed in MWOCNT and MWOCNT-CTAB (**Supplementary Fig. 27**). Therefore, the ECSA data suggest that the enhanced H_2O_2 electrosynthesis performance is likely not attributable to the electronic effect of the cationic modifier.

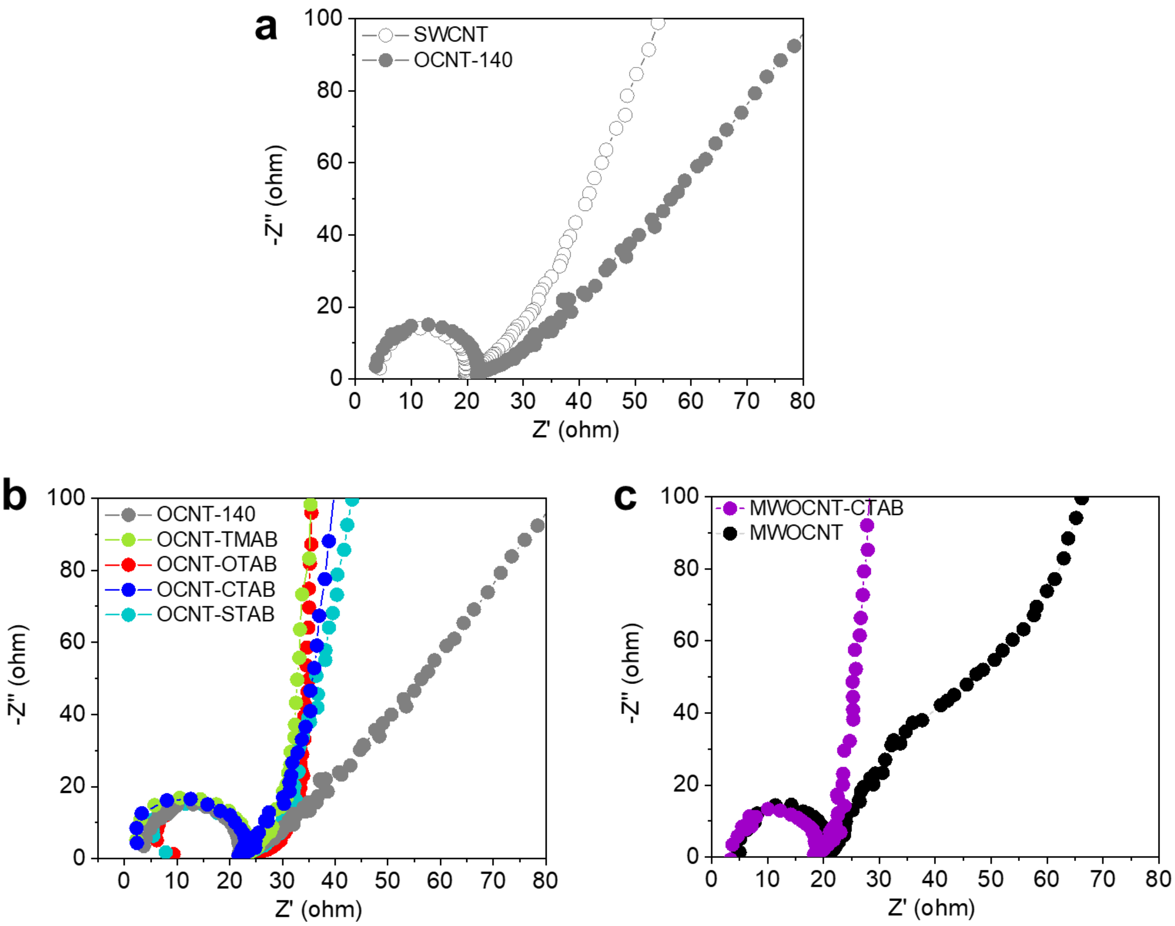

Supplementary Fig. 28 Electrochemical impedance spectroscopy (EIS) results. a, SWCNT and OCNT-140 catalysts. **b,** OCNT-TMAB, OCNT-OTAB, OCNT-CTAB, and OCNT-STAB catalysts. **c,** MWOCNT and MWOCNT-CTAB. Nyquist plots of catalysts measured in 0.3 M K_2SO_4 containing 0.1 M H_2SO_4 at open-circuit voltage. (Experimental conditions: catalyst loading of 0.5 mg cm^{-2}, 0.3 M K_2SO_4 in 0.1 M H_2SO_4 as the electrolyte. The electrolyte resistance was determined to be 3.5 ± 0.5 Ω, however, all reported potentials are not *iR*-corrected. Source data for Supplementary Fig. 28 are provided as a Source Data file.)

To evaluate the electronic and mass-transfer effects of cationic modifier, we measured the electrochemical double-layer capacitance to evaluate the electrochemically active surface area and electrochemical impedance spectroscopy (EIS) to probe the interfacial structure. The Nyquist plots from EIS measurements reveal that a smaller semicircle radius in the high-frequency region indicates faster electron transfer kinetics, while a steeper slope in the low-frequency region represents faster mass transport. EIS resultsshowed that oxidation treatment and cationic polymer modification only have slight impact on electron transfer kinetics, possibly because the modification involves molecular-level interactions with OCNT rather than physical mixing, which didn't adversely affect conductivity. Interestingly, after modification with cationic polymers, the mass transport capability was significantly enhanced, but the difference in mass transport capacity among different chain lengths was relatively small.

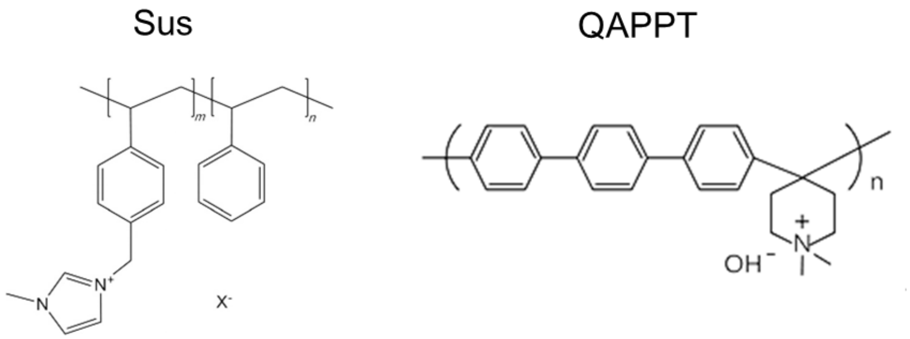

Supplementary Fig. 29 Chemical structures Sus and QAPPT.

OCNT-Sus and OCNT+Sus refer to the modification of OCNT using Sustainion imidazolium-functionalized polymers and the addition of the polymer into the OCNT catalyst ink, respectively. The same principle applies to Fum (Fumion FAA-3 ionomer), a polyaromatic polymer with quaternary ammonium functional groups, and QAPPT, a quaternary ammonia poly(N-methyl-piperidine-co-p-terphenyl). https://pubs.rsc.org/en/content/articlelanding/2019/ee/c9ee01204d

Altering the preparation method changes the interaction between the quaternary ammonium polymers and OCNT, resulting in a significant difference in performance enhancement.

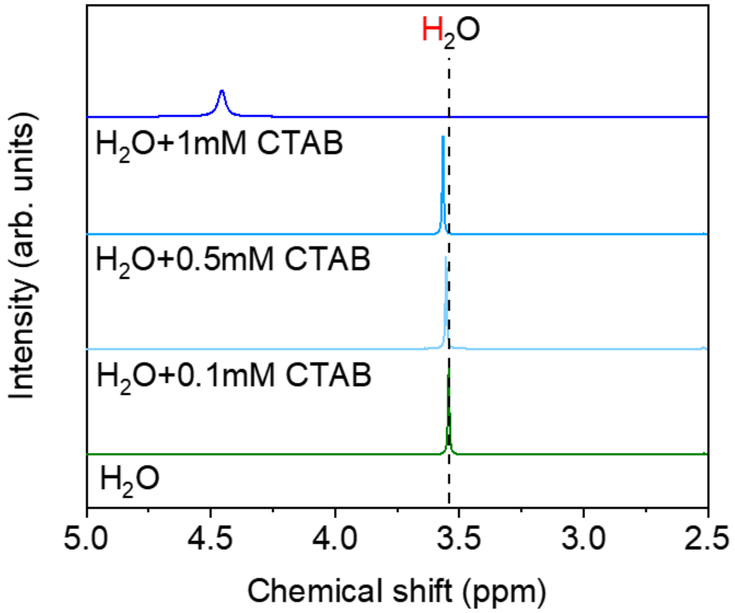

Supplementary Fig. 30 ^{1}H of H_2O NMR spectra with and without the addition of CTAB. (Source data for Supplementary Fig. 30 are provided as a Source Data file.)

The ^{1}H chemical shift of H_2O shifts to higher (downfield) with the addition of CTAB, indicating that the hydrogen atoms are losing electrons. This shift can be attributed to the hydrogen bonding interactions between the hydrogen atoms of H_2O and the electronegative nitrogen atoms in the CTAB molecule. Moreover, the interaction between H_2O and CTAB increases as the concentration of CTAB rises.

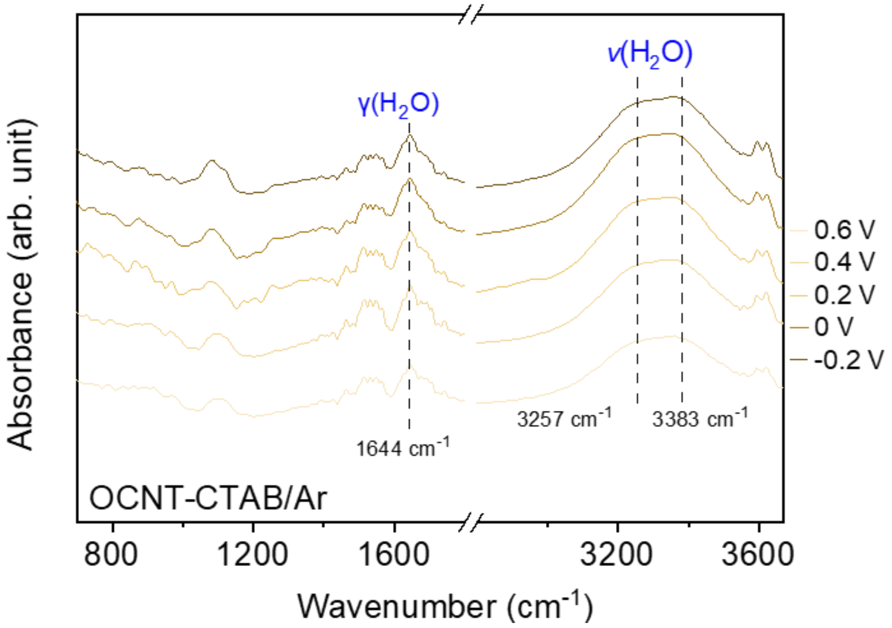

Supplementary Fig. 31 *In situ* ATR-SEIRAS measurements using the OCNT-CTAB electrode at gradually negatively shifted potentials in an Ar-saturated electrolyte. (Experimental conditions: 0.3 M K_2SO_4 in 0.1 M H_2SO_4 as the catholyte, 0.5 M H_2SO_4 as the anolyte. The reported potentials are not *iR*-corrected. Source data for Supplementary Fig. 31 are provided as a Source Data file.)

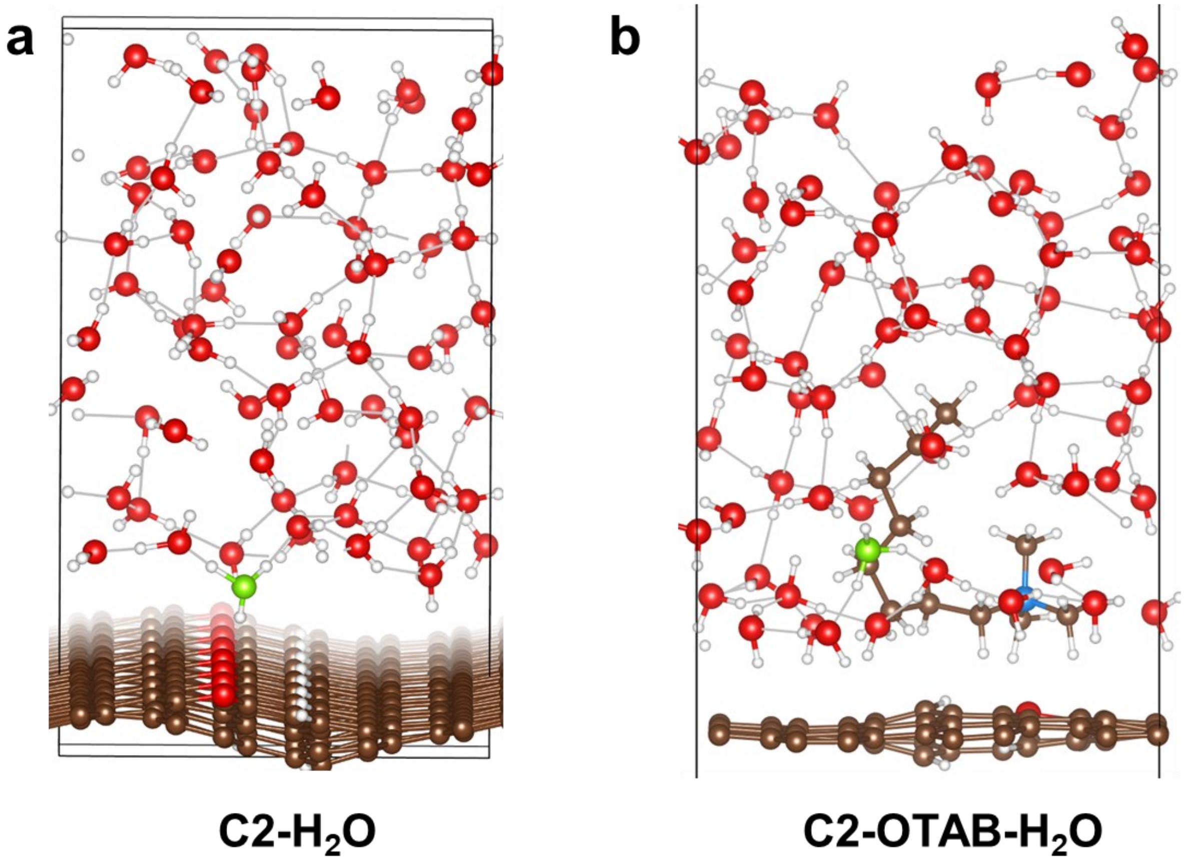

Supplementary Fig. 32 Acidic C2–water interface with proton near the interface. a, C2-H_2O. **b,** C2-OTAB-H_2O. White, brown, blue, and red spheres represent H, C, N and O atoms, respectively, and green sphere represents H of H_3O^+. (Source data for Supplementary Fig. 32 are provided as a Source Data file.)

We further employed AIMD to simulate the 2e$^-$ ORR pathway toward H_2O_2 via sequential hydrogenation of *O_2 to *OOH and *OOH to *H_2O_2 at cationic-modified carbon interfaces within H_2O/H_3O^+ electrolyte. To model the acidic system (C-H_2O-H^+, pH = 0), an H_3O^+ ion was introduced into the water layer. This addition of H_3O^+ triggers spontaneous charge compensation at the electrocatalytic interface, injecting an equivalent number of electrons into the catalyst surface to maintain electroneutrality and modulate the electrode potential. As a result, the electrode potentials are established at −0.94 V vs. RHE for C2-H_2O-H^+ and −1.12 V vs. RHE for C2-OTAB-H_2O-H^+. Although these potentials are sufficiently negative to drive O_2 protonation in principle, they likely concurrently exceed the theoretical threshold for cleaving the O–O bond in the *OOH intermediate, which promotes H_2O formation and leads to low H_2O_2 selectivity.

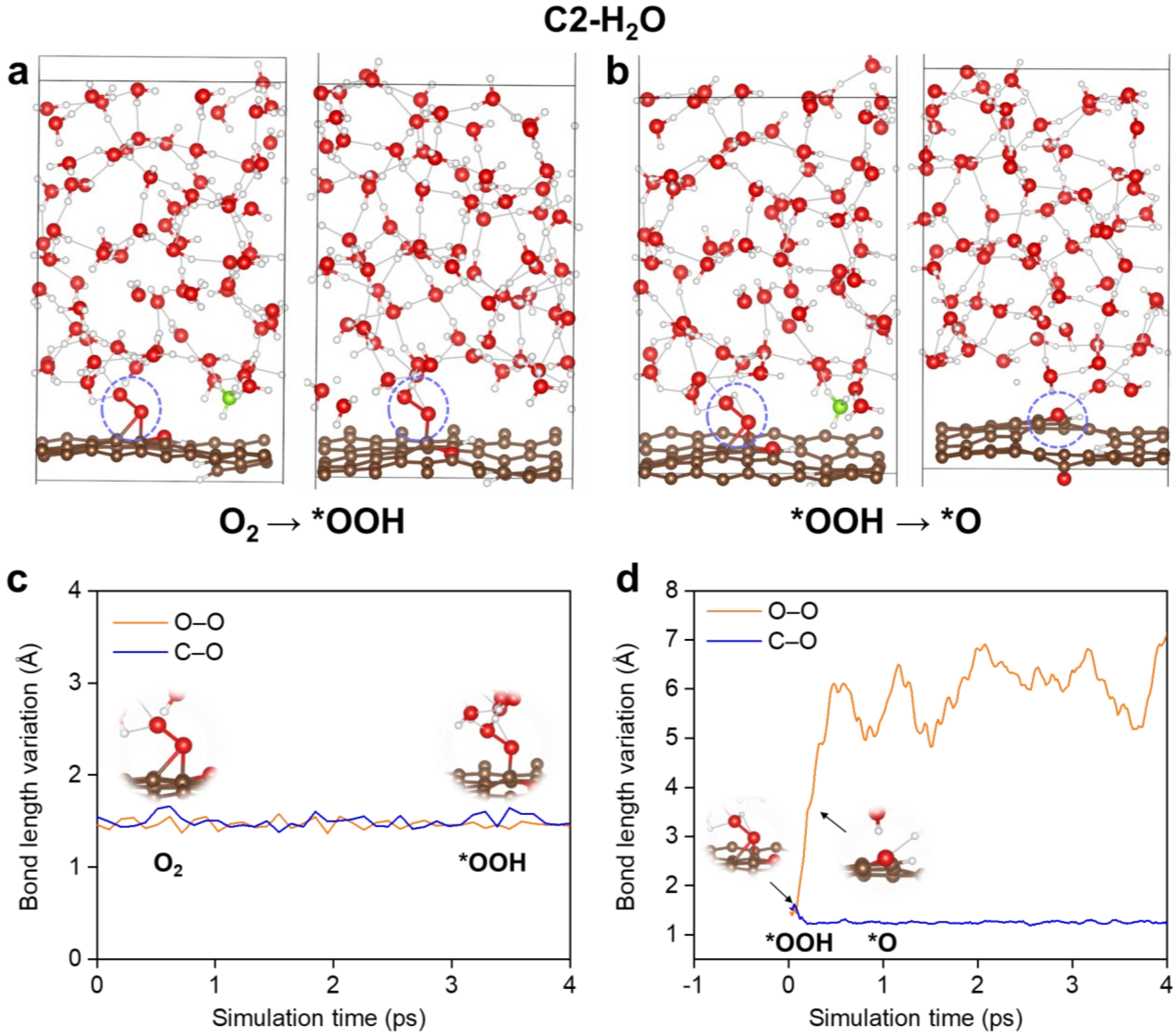

Supplementary Fig. 33 ORR process of AIMD simulations on the C2–water interface with proton. a, c, Representative snapshots for the evolution of $*O_2$ and bond length variation of O–O in the *OOH intermediate and C–O between the O in *OOH and the C site in the C2 carbon catalyst (the insets in Supplementary Fig. 33c show snapshots of $*O_2$ and *OOH adsorption taken from partial view of Supplementary Fig. 33a). **b, d,** Representative snapshots for the evolution of *OOH and bond length variation of O–O in the *OOH intermediate and C–O between the O in *OOH and the C site in the C2 carbon catalyst (the insets in Supplementary Fig. 33d show snapshots of *OOH adsorption and cleavage taken from partial view of Supplementary Fig. 33b). (White, brown, and red spheres represent H, C, and O atoms, respectively, and green sphere represents H of H_3O^+. Source data for Supplementary Fig. 33 are provided as a Source Data file.)

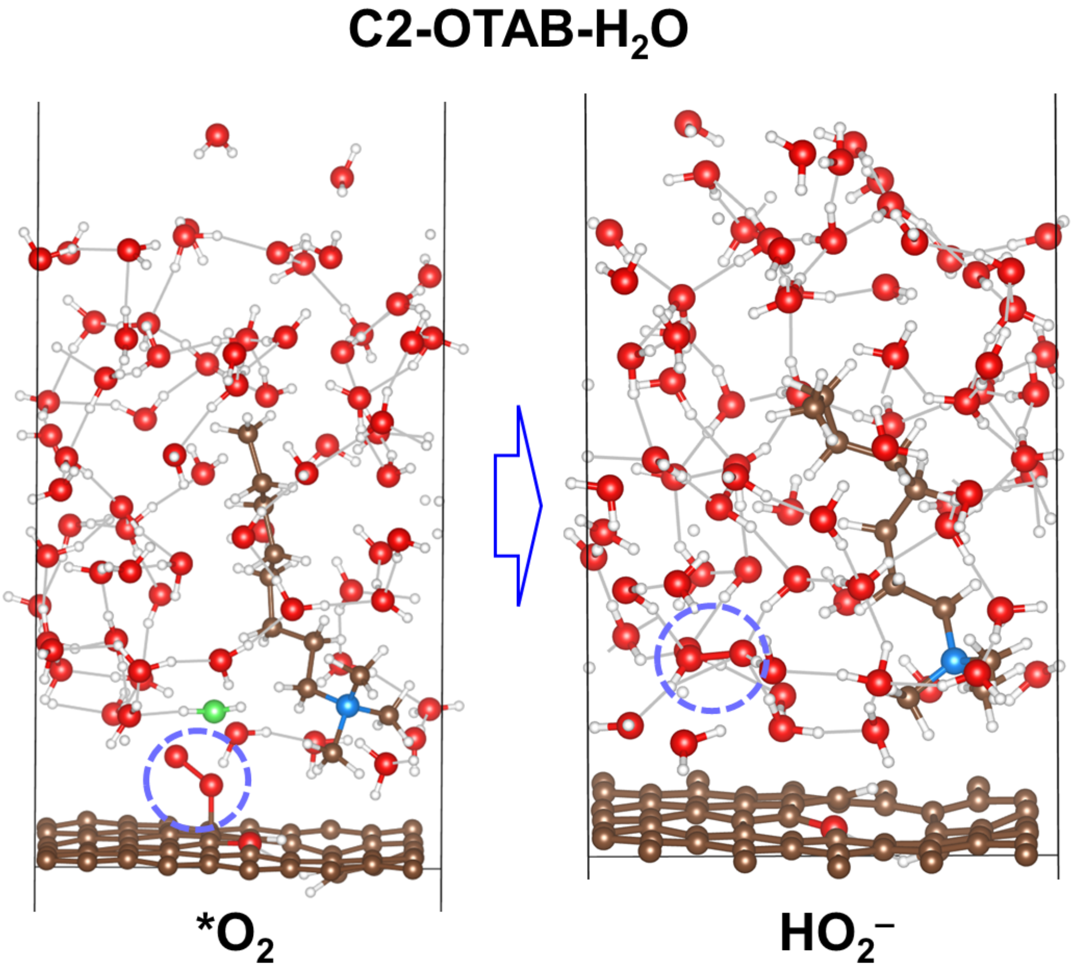

Supplementary Fig. 34 Evolution of *O_2 at the interface of C2-OTAB-H_2O (proton near the interface) through AIMD simulations. (White, brown, blue, and red spheres represent H, C, N and O atoms, respectively, and green sphere represents H of H_3O^+. Source data for Supplementary Fig. 34 are provided as a Source Data file.)

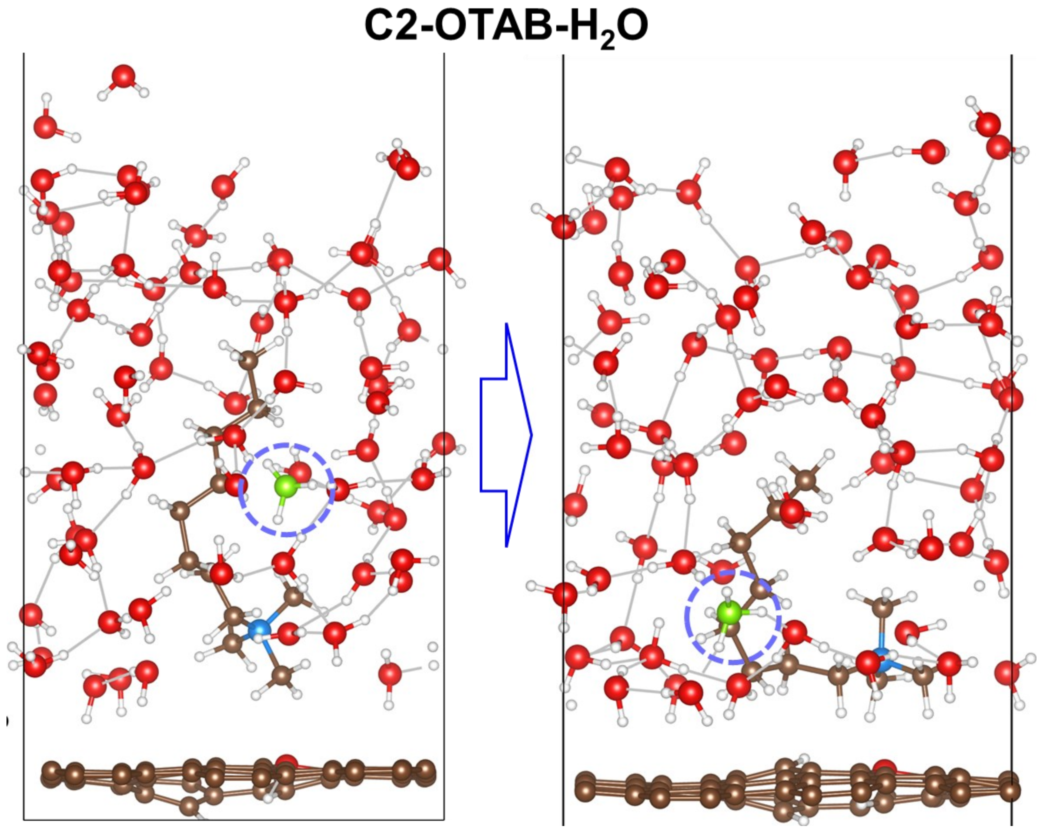

Supplementary Fig. 35 Evolution process of proton at the interface of C2-OTAB-H_2O through AIMD simulations. (White, brown, blue, and red spheres represent H, C, N and O atoms, respectively, and green sphere represents H of H_3O^+. Source data for Supplementary Fig. 35 are provided as a Source Data file.)

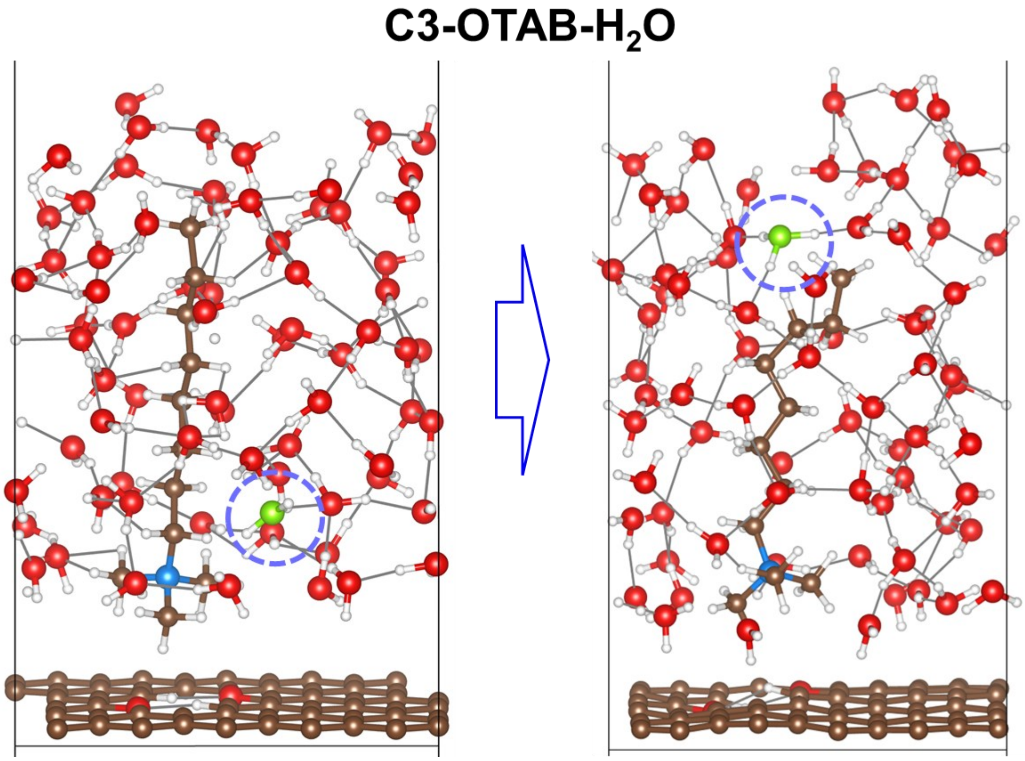

Supplementary Fig. 36 Evolution process of proton at the interface of C3-OTAB-H_2O (proton near the interface) through AIMD simulations. (White, brown, blue, and red spheres represent H, C, N and O atoms, respectively, and green sphere represents H of H_3O^+. Source data for Supplementary Fig. 36 are provided as a Source Data file.)

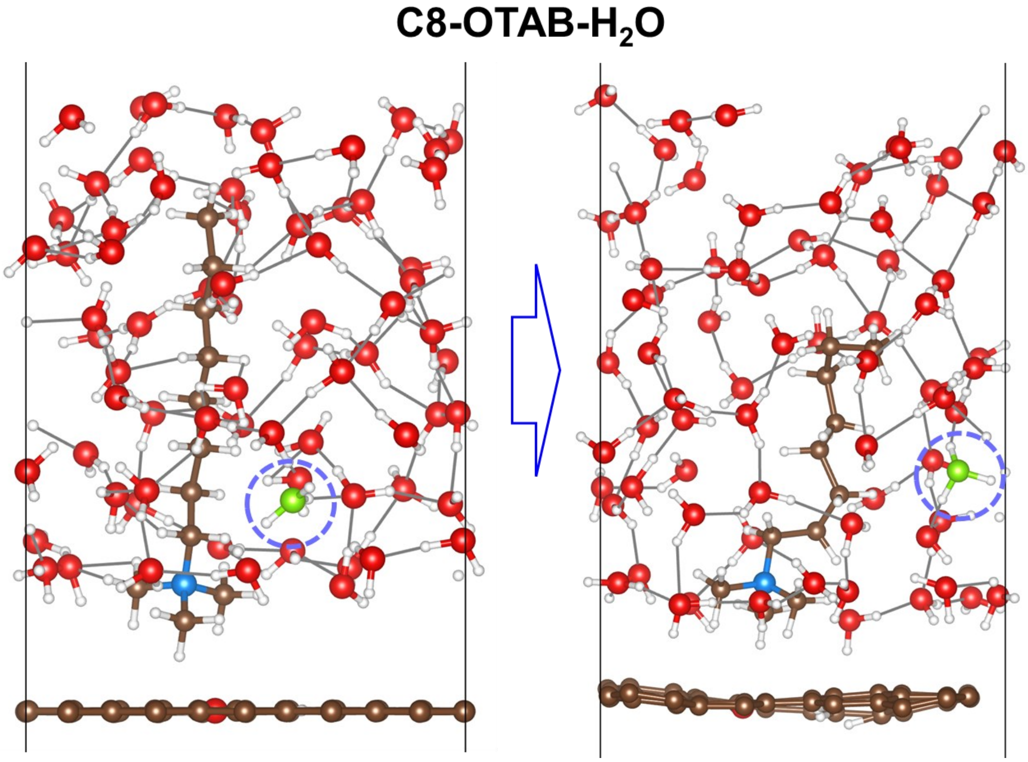

Supplementary Fig. 37 Evolution process of $^*O_2$ at the interface of C8-OTAB-H_2O (proton near the interface) through AIMD simulations. (White, brown, blue, and red spheres represent H, C, N and O atoms, respectively, and green sphere represents H of H_3O^+. Source data for Supplementary Fig. 37 are provided as a Source Data file.)

We first examined the evolution trajectory of a randomly positioned hydrated H^+ at the interface of the C2-OTAB-H_2O. The evolution trajectory indicated that the H^+ primarily transfers protons through the hydrogen bond network of H_2O. As shown in Supplementary Fig. 35, the distance between H^+ and N^+ in OTAB was initially approximately 6.7 Å. After trajectory evolution, the distance increased to 9.3 Å. This motion and evolution may be related to the electrostatic repulsion between N^+ in OTAB and H_3O^+, which may be helpful to suppress the direct hydrogenation of *OOH by H_3O^+, while promoting the hydrogenation pathway in which H_2O provides protons. We further observed this phenomenon at both the C3 and C8 sites. At the C3 site, the distance between H^+ and N^+ in OTAB increased from 5.48 Å to 10.4 Å due to repulsion. Similarly, at the C8 site, the distance increased from 5.48 Å to 7.52 Å as a result of repulsion.

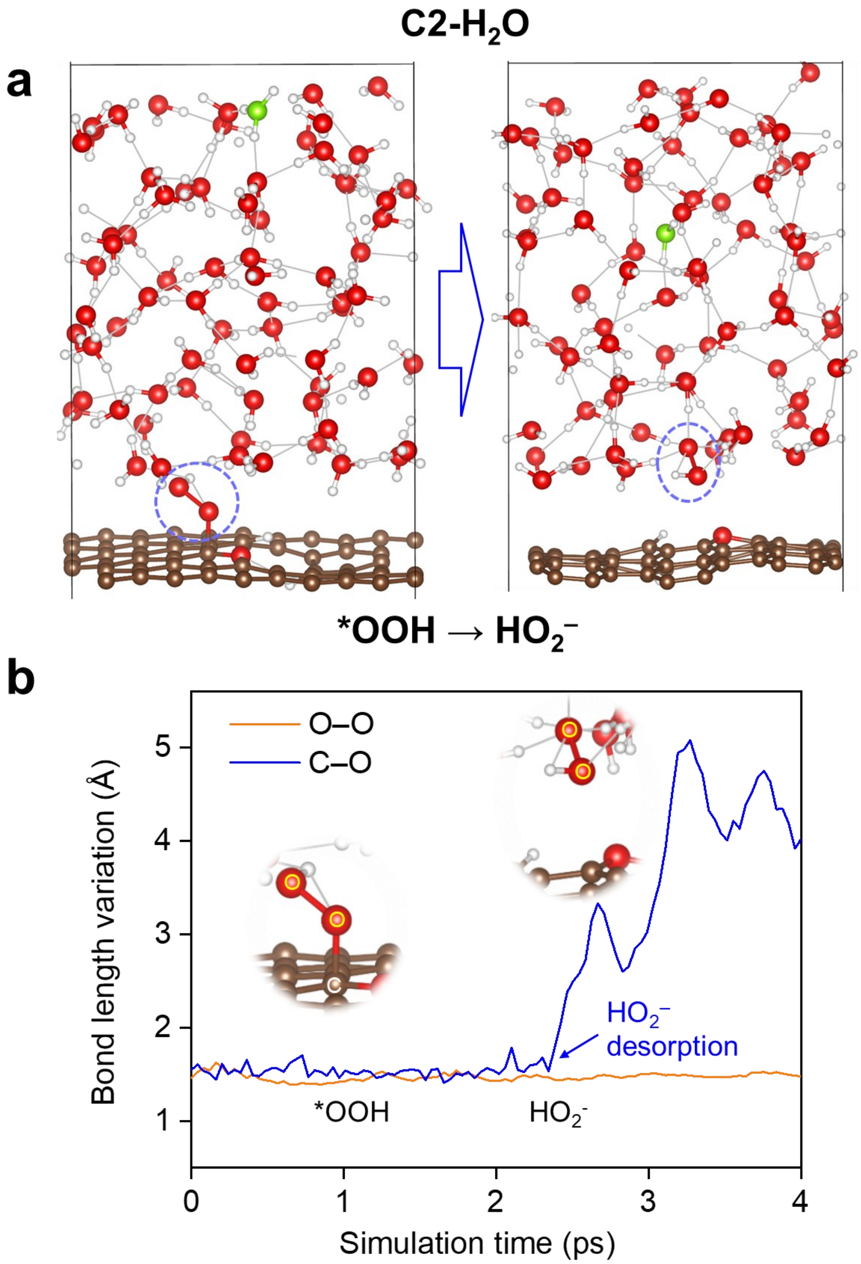

Supplementary Fig. 38 Evolution process of *OOH on the C2-H_2O interface with proton away from the interface through AIMD simulations. a, Snapshot of the *OOH evolution process. **b,** Bond length variation of O–O in the *OOH intermediate and C–O between the O in *OOH and the C site in the C2 carbon catalyst during the evolution of *OOH (the insets in Supplementary Fig. 38b show snapshots of *OOH adsorption and desorption taken from partial view of Supplementary Fig. 38a). (White, brown, and red spheres represent H, C and O atoms, respectively, and green sphere represents H of H_3O^+. Source data for Supplementary Fig. 38 are provided as a Source Data file.)

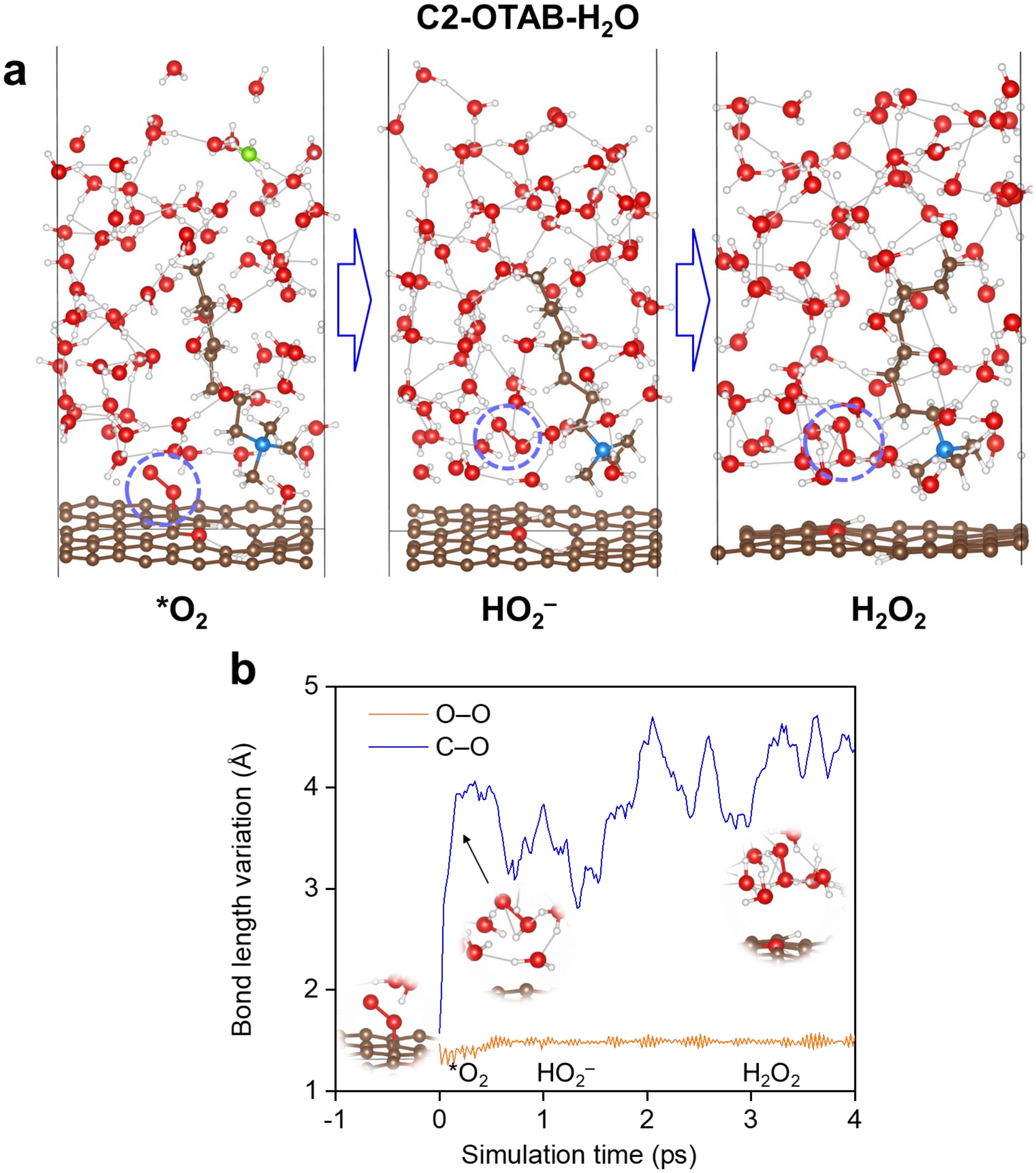

Supplementary Fig. 39 Evolution process of $^*O_2$ at the interface of C2-OTAB-H_2O with proton away from the interface through AIMD simulations. a, Snapshot of the $^*O_2$ evolution process. **b,** Bond length variation of O–O in the $^*O_2$ and *OOH intermediate and C–O between the O in the $^*O_2$ and *OOH and the C site in the C2 carbon catalyst during the evolution of *OOH (the insets in Supplementary Fig. 39b show snapshots of $^*O_2$ adsorption, HO_2^- desorption and H_2O_2 formation taken from partial view of Supplementary Fig. 39a). (White, brown, blue, and red spheres represent H, C, N and O atoms, respectively, and green sphere represents H of H_3O^+. Source data for Supplementary Fig. 39 are provided as a Source Data file.)

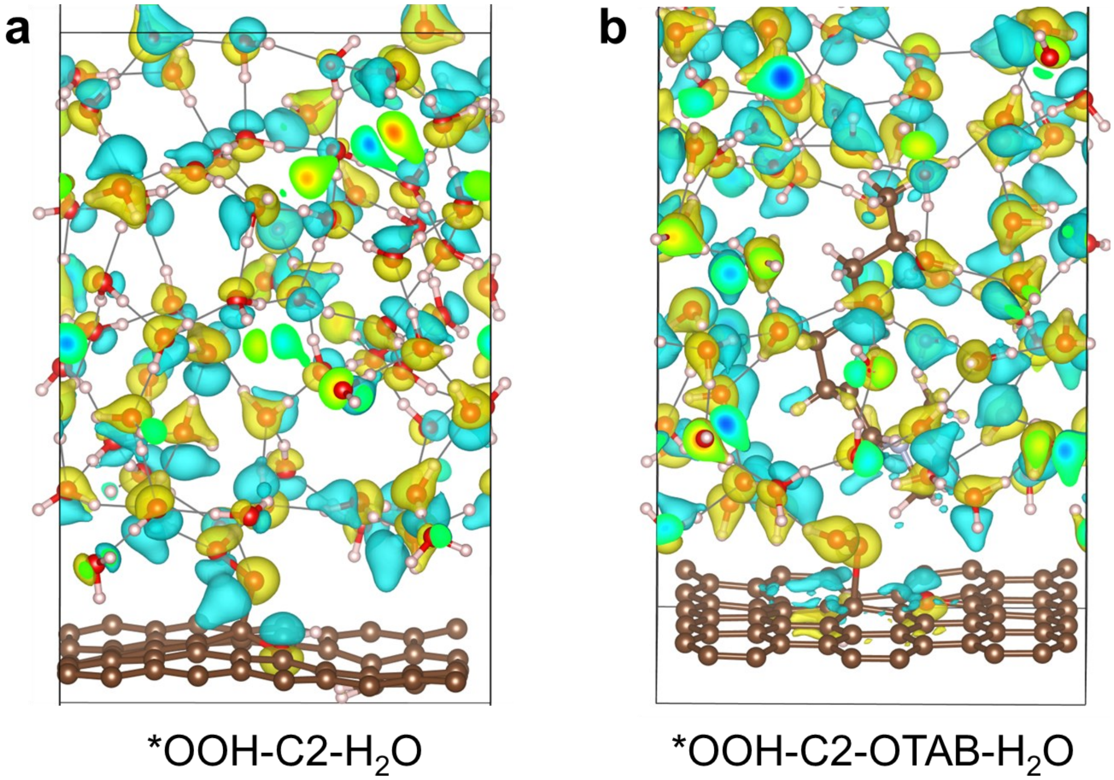

Supplementary Fig. 40 Differential charge density distributions of adsorbed *OOH. **a,** C2-H_2O. **b,** C2-OTAB-H_2O. White, brown, blue, and red spheres represent H, C, N and O atoms, respectively, and green sphere represents H of H_3O^+.

In the differential charge density map, blue region indicates electron density depletion (loss), and yellow indicates accumulation (gain). The pattern indicates that upon *OOH adsorption, electron density is transferred from the catalytic C site to the interacting O atom of OOH.

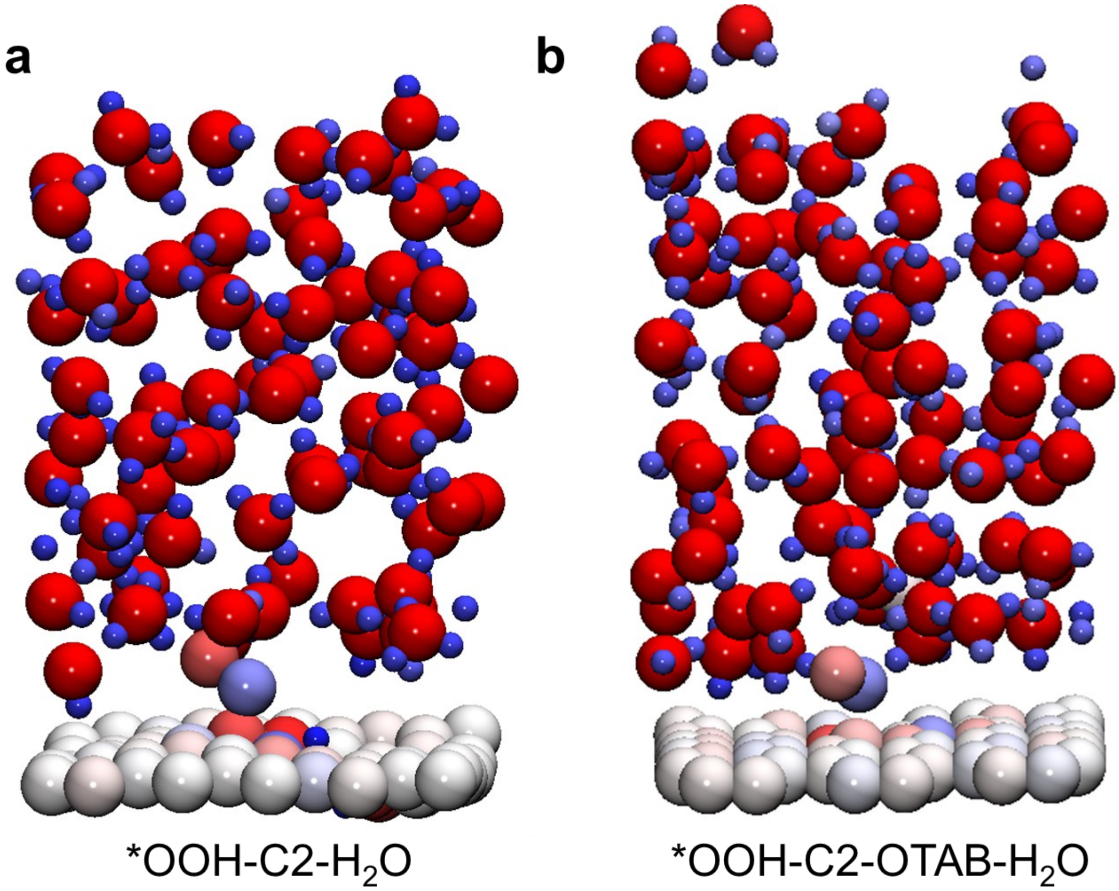

Supplementary Fig. 41 Atom charge distribution of adsorbed *OOH on the C2-H_2O and C2-OTAB-H_2O surfaces. a, C2-H_2O. **b,** C2-OTAB-H_2O. Red and blue indicate electron loss and gain, respectively. The results show charge depletion at the C atom and charge accumulation at the O atom of adsorbed *OOH.

We calculated the charge variation of *OOH at the catalytic site. When *OOH is adsorbed on the bare C2 site, the carbon site shows a charge change of -0.26 |e| before and after adsorption, with an O–O bond length of 1.51 Å. The shorter O–O bond length for *OOH adsorbed on the bare C2 site indicates a stronger interaction with the site, which can easily lead to O–O bond cleavage.

In contrast, when *OOH is adsorbed on the CTAB-modified C2 site, the carbon site exhibits a charge change of -0.19 |e| before and after adsorption, with an O–O bond length of 1.84 Å. The longer O–O bond length on the CTAB-modified C2 site reflects a weaker interaction, which is beneficial for *OOH desorption.

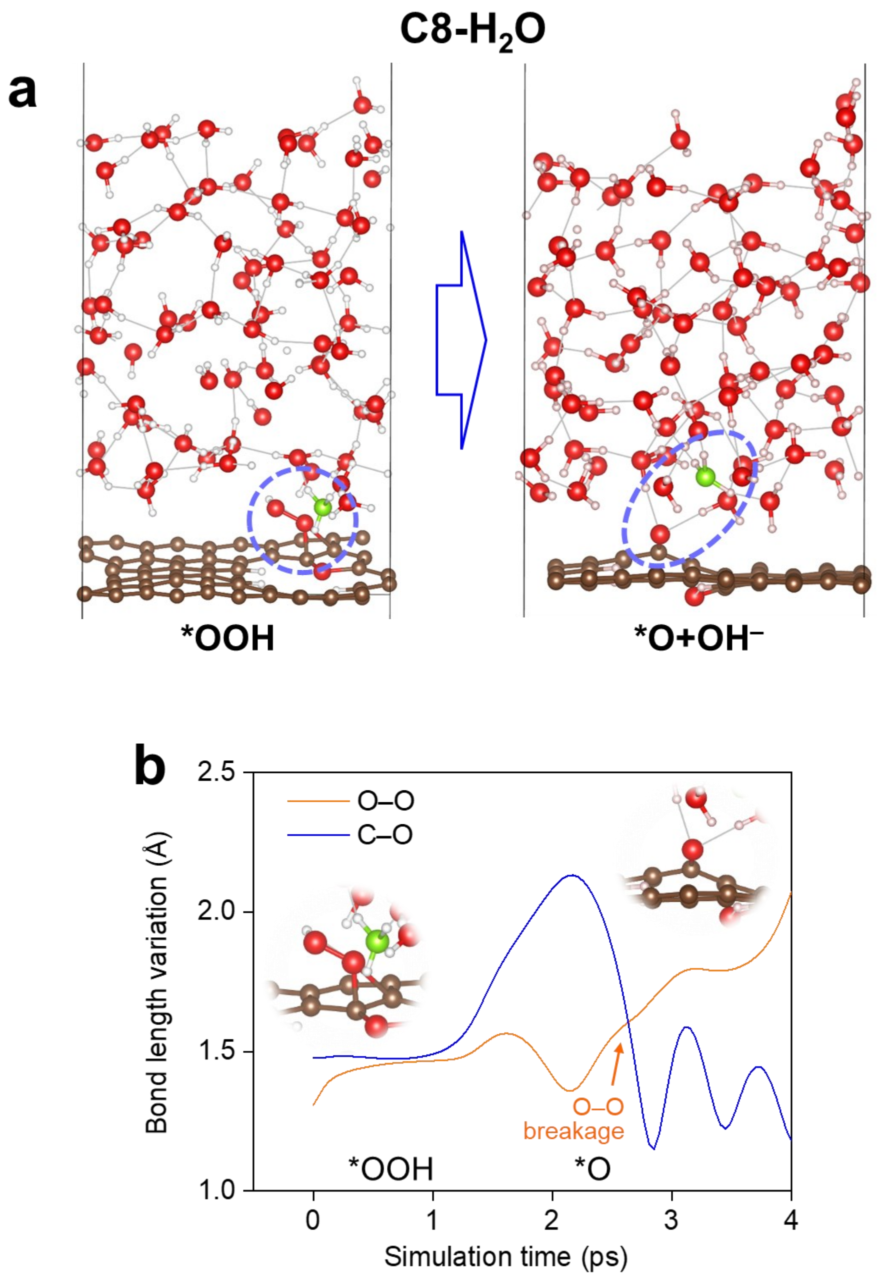

Supplementary Fig. 42 Evolution process of *OOH at the interface of C8-H_2O through AIMD simulations. **a,** Snapshot of the *OOH evolution process. **b,** Bond length variation of *O–O in the *OOH and C–O between the O in *OOH and the C site in the C8 carbon catalyst (the insets in Supplementary Fig. 42b show snapshots of *OOH adsorption and breakage taken from partial view of Supplementary Fig. 42a). (White, brown, and red spheres represent H, C and O atoms, respectively, and green sphere represents H of H_3O^+. Source data for Supplementary Fig. 42 are provided as a Source Data file.)

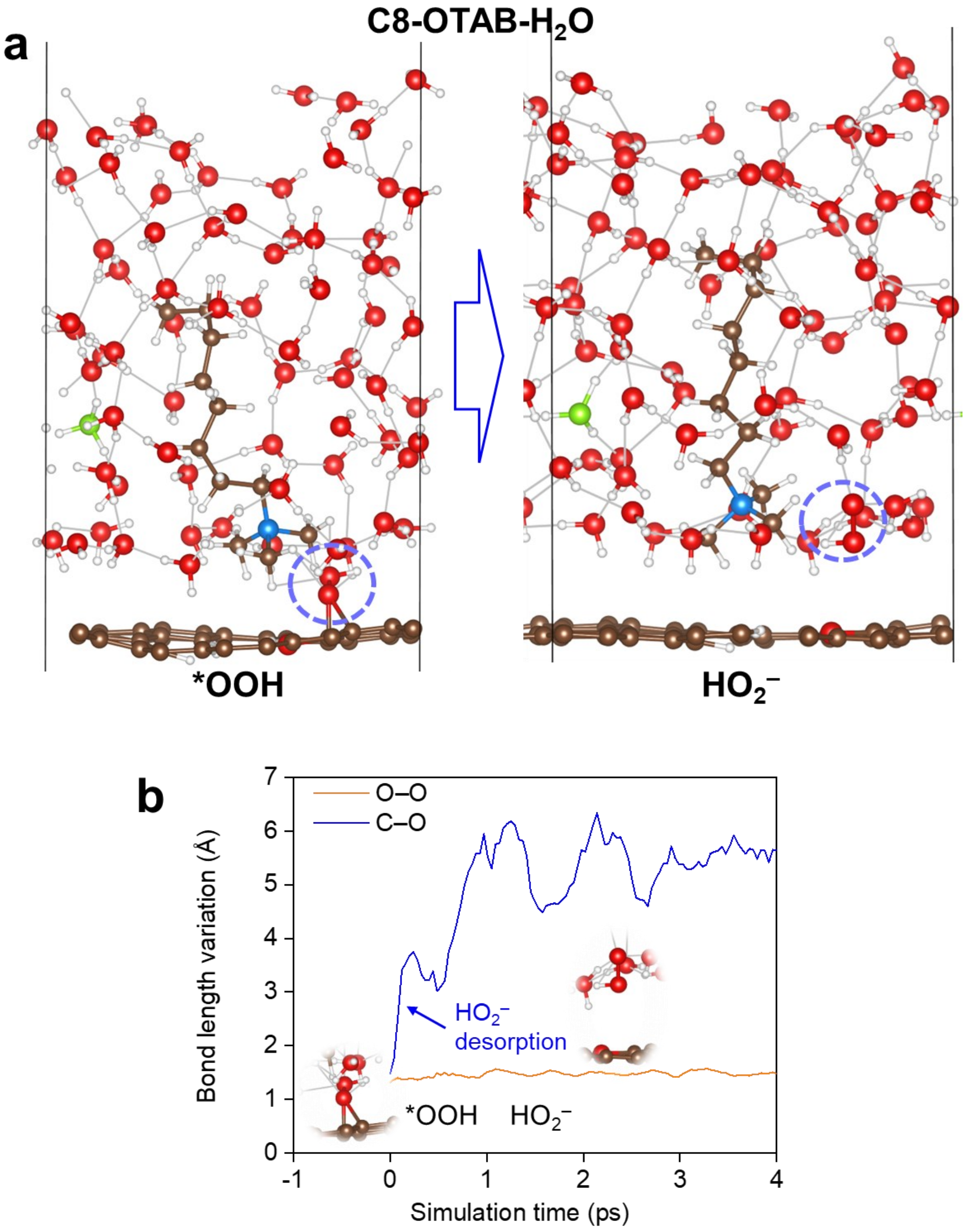

Supplementary Fig. 43 Evolution process of *OOH at the interface of C8-OTAB-H_2O through AIMD simulations. a, Snapshot of the *OOH evolution process. **b,** Bond length variation of *O–O in the *OOH and C–O between the O in *OOH and the C site in the C8 carbon catalyst (the insets in Supplementary Fig. 43b show snapshots of *OOH adsorption and breakage taken from partial view of Supplementary Fig. 43a). (White, brown, blue, and red spheres represent H, C, N and O atoms, respectively, and green sphere represents H of H_3O^+. Source data for Supplementary Fig. 43 are provided as a Source Data file.)

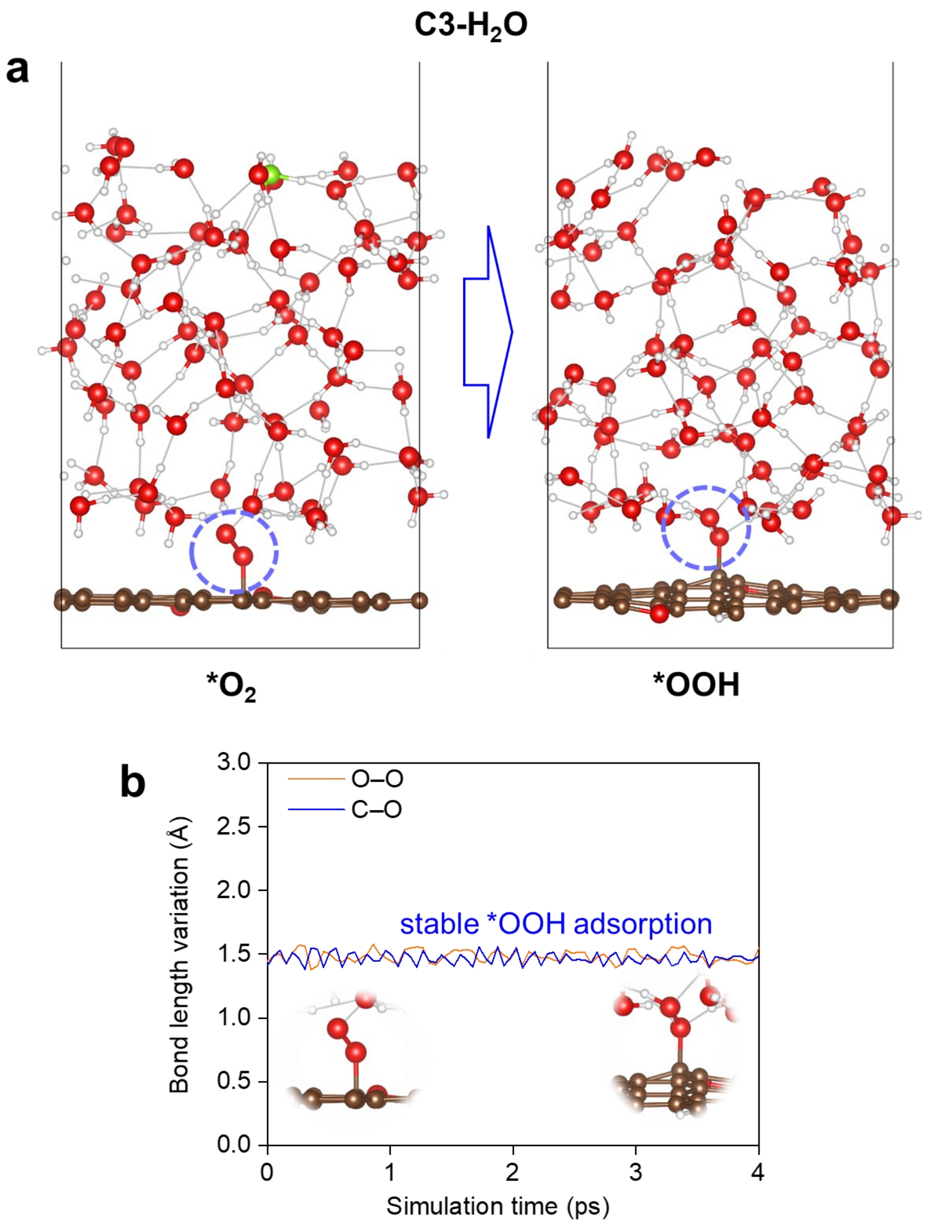

Supplementary Fig. 44 Evolution process of $^*O_2$ at the interface of C3-H_2O through AIMD simulations. a, Snapshot of the$^*O_2$ evolution process. **b,** Bond length variation of *O–O in the $^*O_2$ and C–O between the O in $^*O_2$ and the C site in the C3 carbon catalyst (the insets in Supplementary Fig. 44b show snapshots of $^*O_2$ and *OOH adsorption taken from partial view of Supplementary Fig. 44a). (White, brown, and red spheres represent H, C and O atoms, respectively, and green sphere represents H of H_3O^+. Source data for Supplementary Fig. 44 are provided as a Source Data file.)

It should be noted that the calculated electrode potentials for both unmodified and cation-modified C8 and C3 interfaces ranged from −0.95 to −1.14 V, demonstrating similar driving potential to the C2 system.

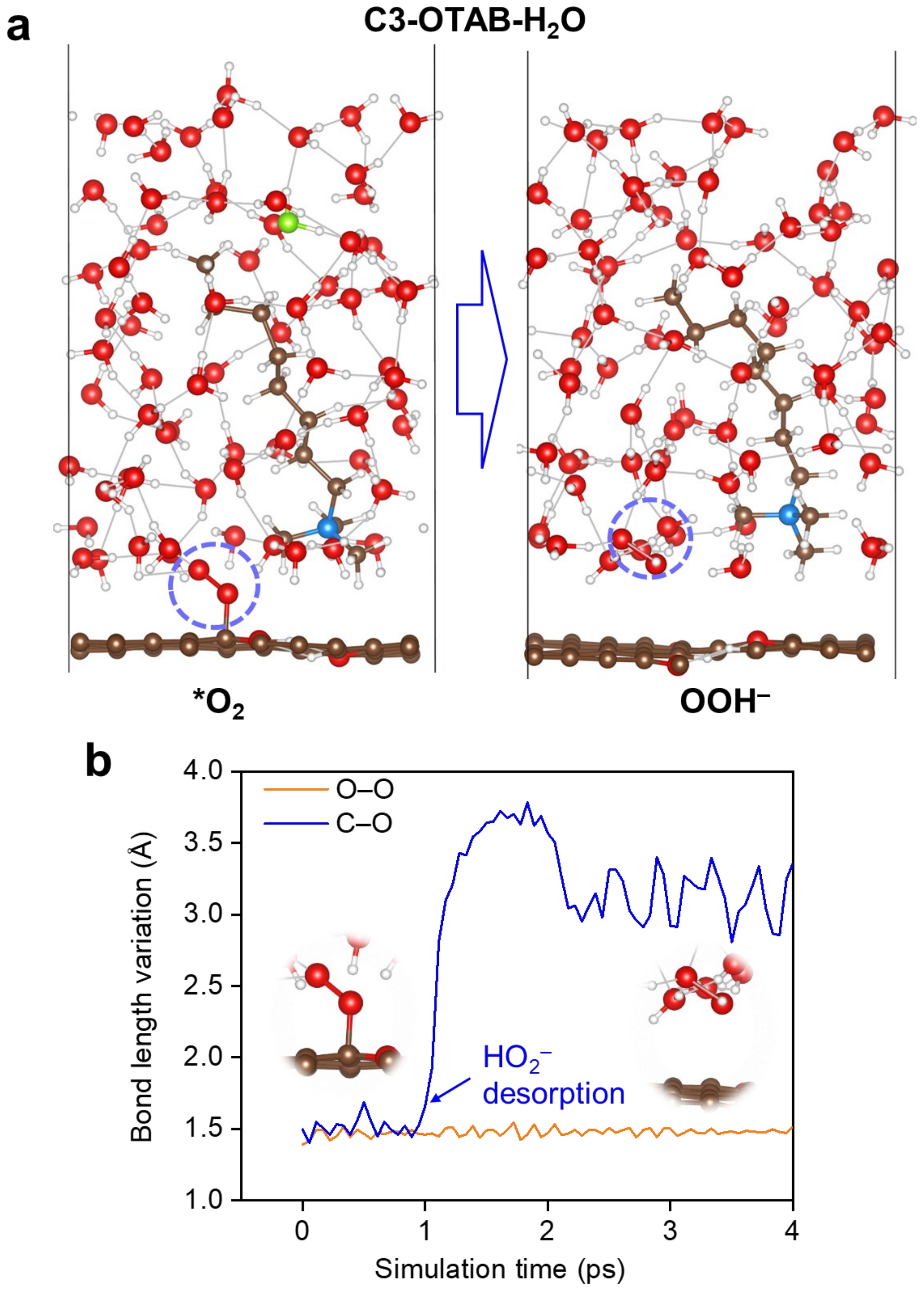

Supplementary Fig. 45 Evolution process of $*O_2$ at the interface of C3-OTAB-H_2O through AIMD simulations. a, Snapshot of the $*O_2$ evolution process. **b,** Bond length variation of *O–O in the $*O_2$ and C–O between the O in $*O_2$ and the C site in the C3 carbon catalyst (the insets in Supplementary Fig. 45b show snapshots of $*O_2$ adsorption and HO_2^- desorption taken from partial view of Supplementary Fig. 45a). (White, brown, blue, and red spheres represent H, C, N and O atoms, respectively, and green sphere represents H of H_3O^+. Source data for Supplementary Fig. 45 are provided as a Source Data file.)

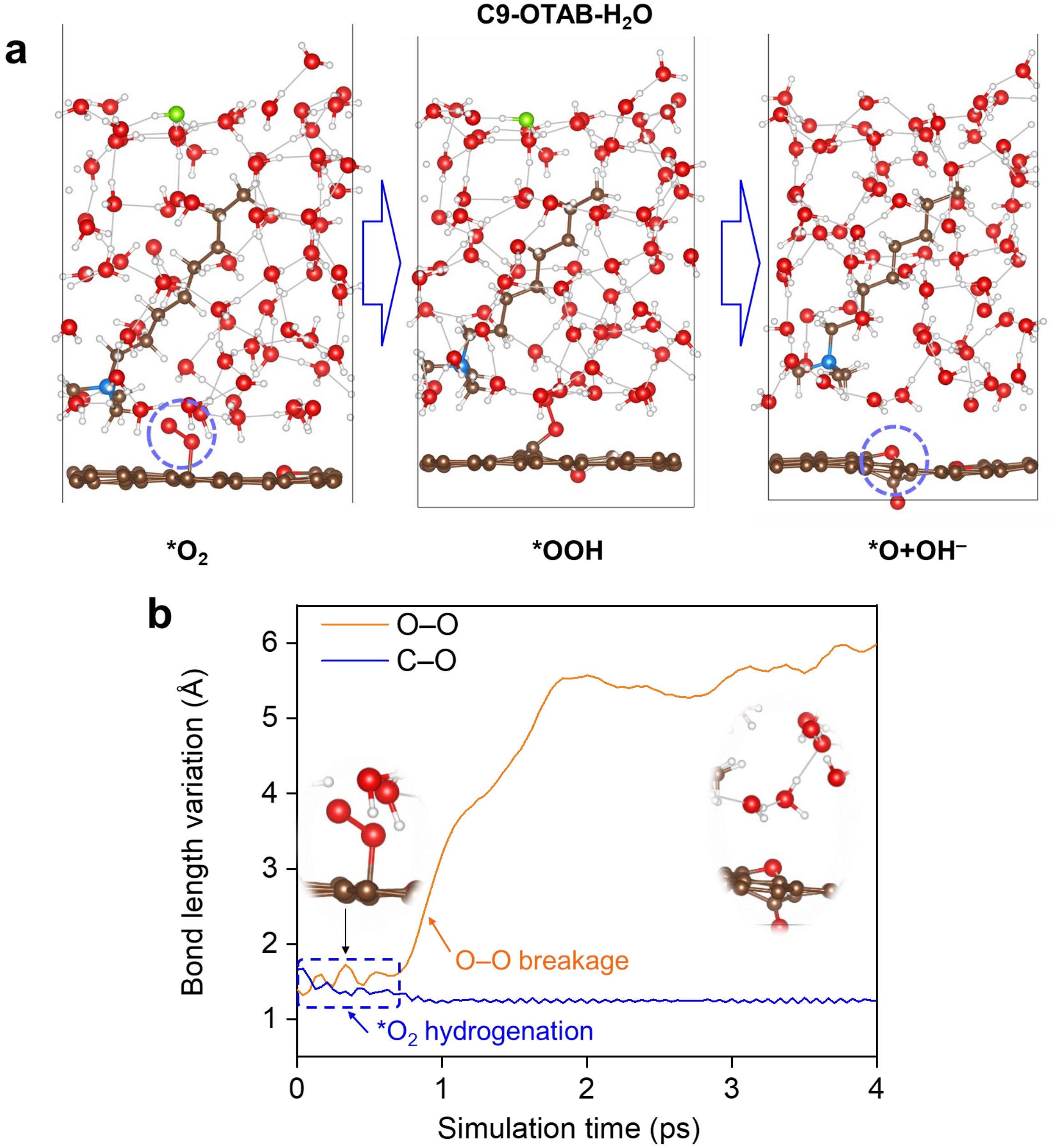

Supplementary Fig. 46 Evolution process of *O_2 at the interface of C9-OTAB-H_2O through AIMD simulations. a, Snapshot of the *O_2 evolution process. **b,** Bond length variation of *O–O in the *O_2 and C–O between the O in *O_2 and the C site in the C9 carbon catalyst (the insets in Supplementary Fig. 46b show snapshots of *O_2 adsorption and *OOH breakage taken from partial view of Supplementary Fig. 46a). (White, brown, blue, and red spheres represent H, C, N and O atoms, respectively, and green sphere represents H of H_3O^+. Source data for Supplementary Fig. 46 are provided as a Source Data file.)

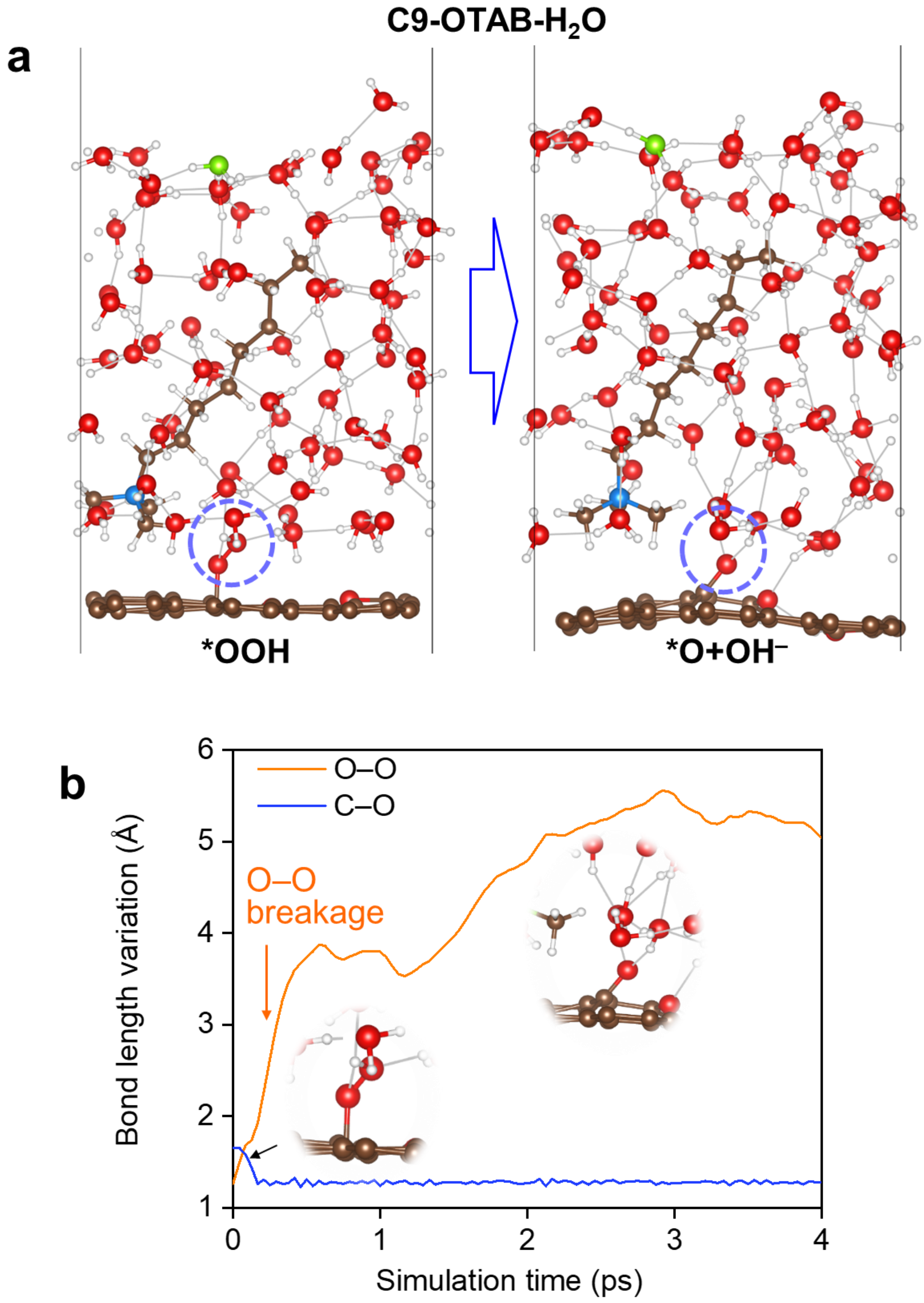

Supplementary Fig. 47 Evolution process of *OOH at the interface of C9-OTAB-H_2O through AIMD simulations. a, Snapshot of the *OOH evolution process. **b,** Bond length variation of *O–O in the *OOH and C–O between the O in *OOH and the C site in the C9 carbon catalyst (the insets in Supplementary Fig. 47b show snapshots of *OOH adsorption and breakage taken from partial view of Supplementary Fig. 47a). (White, brown, blue, and red spheres represent H, C, N and O atoms, respectively, and green sphere represents H of H_3O^+. Source data for Supplementary Fig. 47 are provided as a Source Data file.)

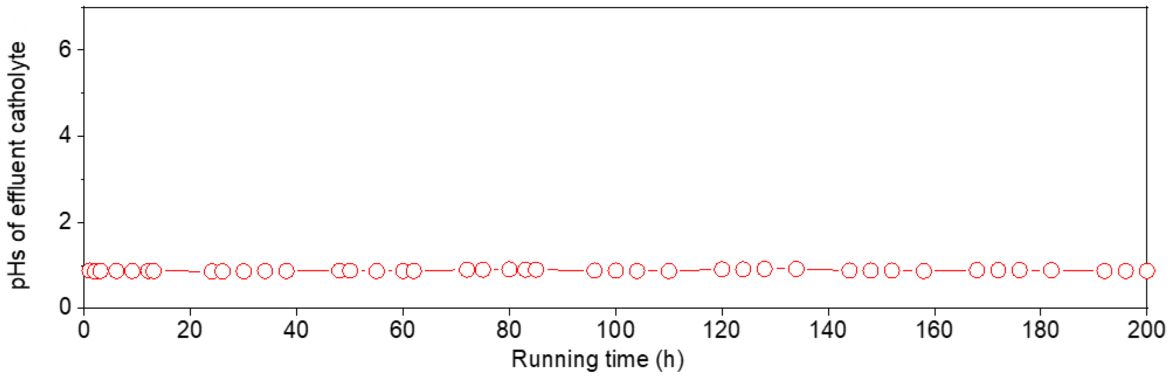

Supplementary Fig. 48 pH of effluent catholyte during stability test conducted at 300 mA cm^{-2} for the OCNT-CTAB catalyst. (The pH value was measured once for each point. Source data for Supplementary Fig. 48 are provided as a Source Data file.)

The slight decrease in Faradaic efficiency observed in **Fig. 4a** of the main text over each cycle was primarily due to electrode flooding, a common issue in high-current electrolysis, where the electrolyte permeated into the electrode. This flooding was easily addressed by drying the electrode, which restored its performance. The pH of the effluent catholyte remained acidic.

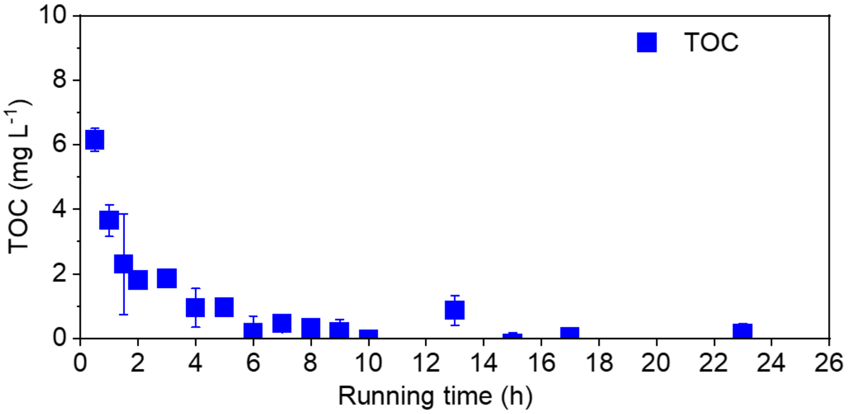

Supplementary Fig. 49 Total organic carbon (TOC) concentration in the effluent H_2O_2 solution during a repeated 23-hour stability electrolysis test. (Data for TOC represent the average of triplicate measurements, with error bars indicating standard deviations. Source data for Supplementary Fig. 49 are provided as a Source Data file.)

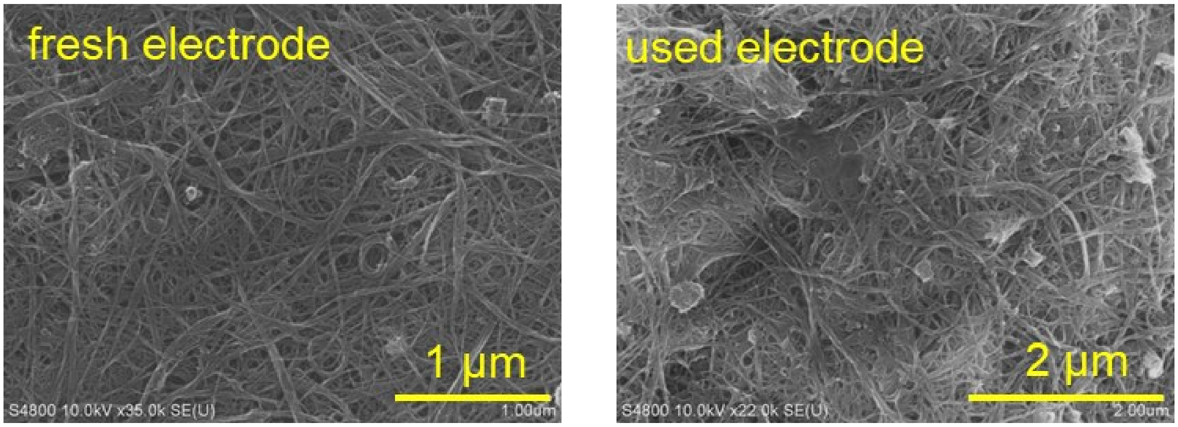

Supplementary Fig. 50 OCNT-CTAB electrode photographs before and after stability test conducted at 300 mA cm^{-2} for 200 hours. All reported data represent the average of triplicate measurements, with error bars indicating standard deviations.

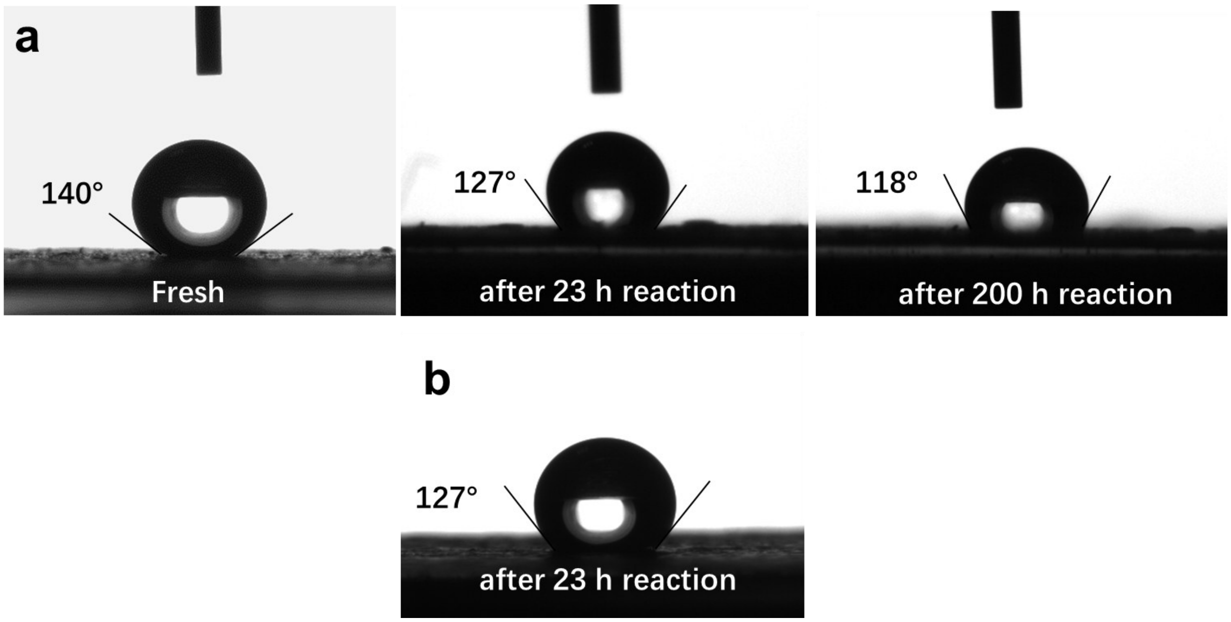

Supplementary Fig. 51 Water contact angles of the OCNT-CTAB electrode before and after reaction conducted at 300 mA cm^{-2} for 23 and 200 hours. a, catalytic layer. **b,** back side of electrode (gas diffusion layer). Two independent water contact angles measurements were performed for each sample, and the average value was reported. Prior to reaction, the backside GDL surface was superhydrophobic, with a contact angle estimated at >150°. In this state, droplets beaded up and rolled off easily, making stable measurement unattainable.

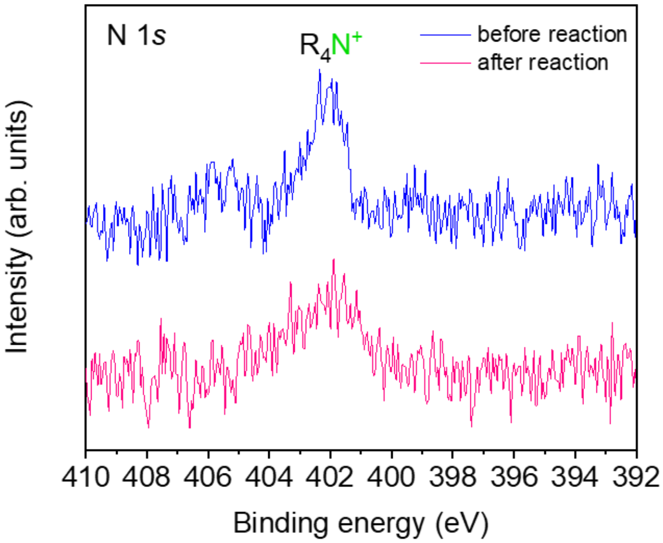

Supplementary Fig. 52 XPS N 1*s* high-resolution spectra of the OCNT-CTAB electrode after reaction conducted at 300 mA cm^{-2} for 200 hours. (Source data for Supplementary Fig. 52 are provided as a Source Data file.)

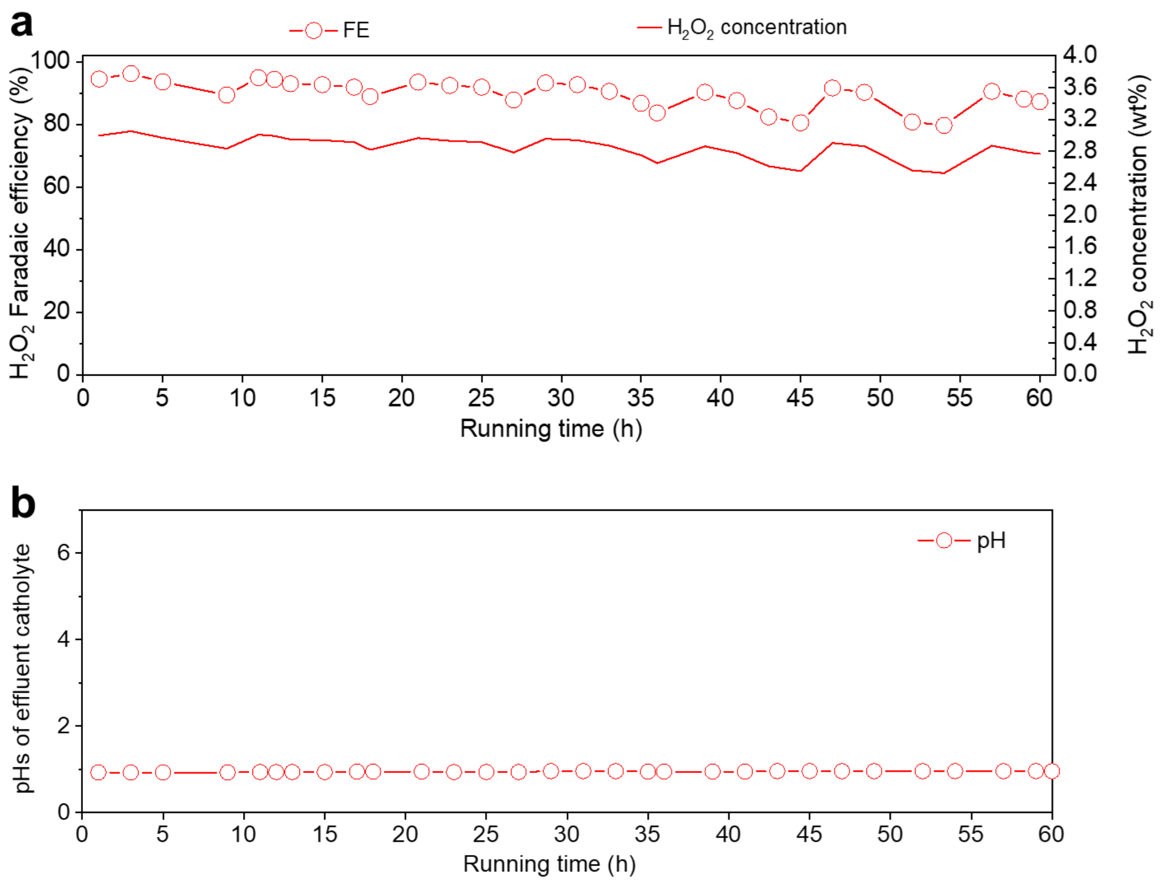

Supplementary Fig. 53 Stability test conducted at 400 mA cm^{-2} for the OCNT-CTAB catalyst. a, H_2O_2 Faradaic efficiency. **b,** pH of catholyte. (The data points for H_2O_2 Faradaic efficiency were measured twice, and the pH value was measured once for each point. Source data for Supplementary Fig. 53 are provided as a Source Data file.)

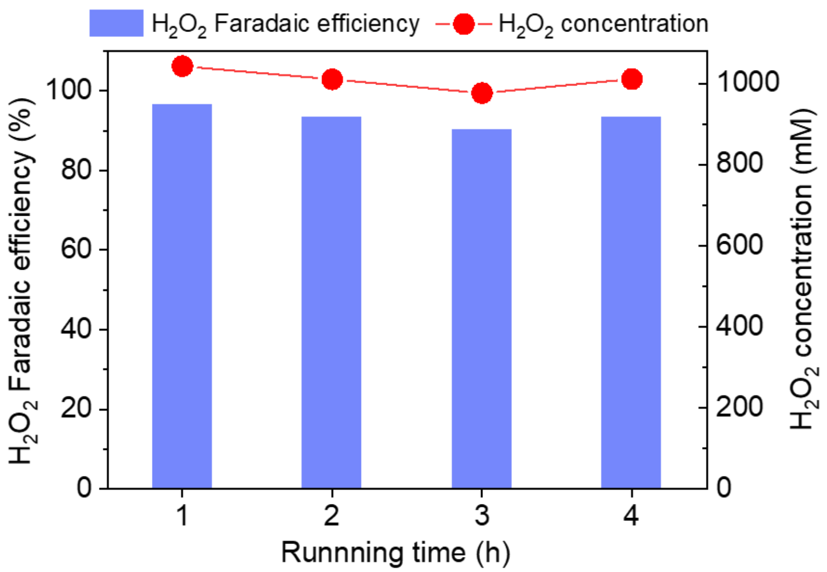

Supplementary Fig. 54 Performance investigation of acidic H_2O_2 electrosynthesis at 300 mA cm^{-2} using OCNT-CTAB cathode and Pt anode in 0.1 M H_2SO_4 with 0.3 M Na_2SO_4 catholyte (flow rate of 20.7 mL h^{-1}) and 0.5 M H_2SO_4 anolyte. (The data points for H_2O_2 Faradaic efficiency were measured at least twice. Source data for Supplementary Fig. 54 are provided as a Source Data file.)

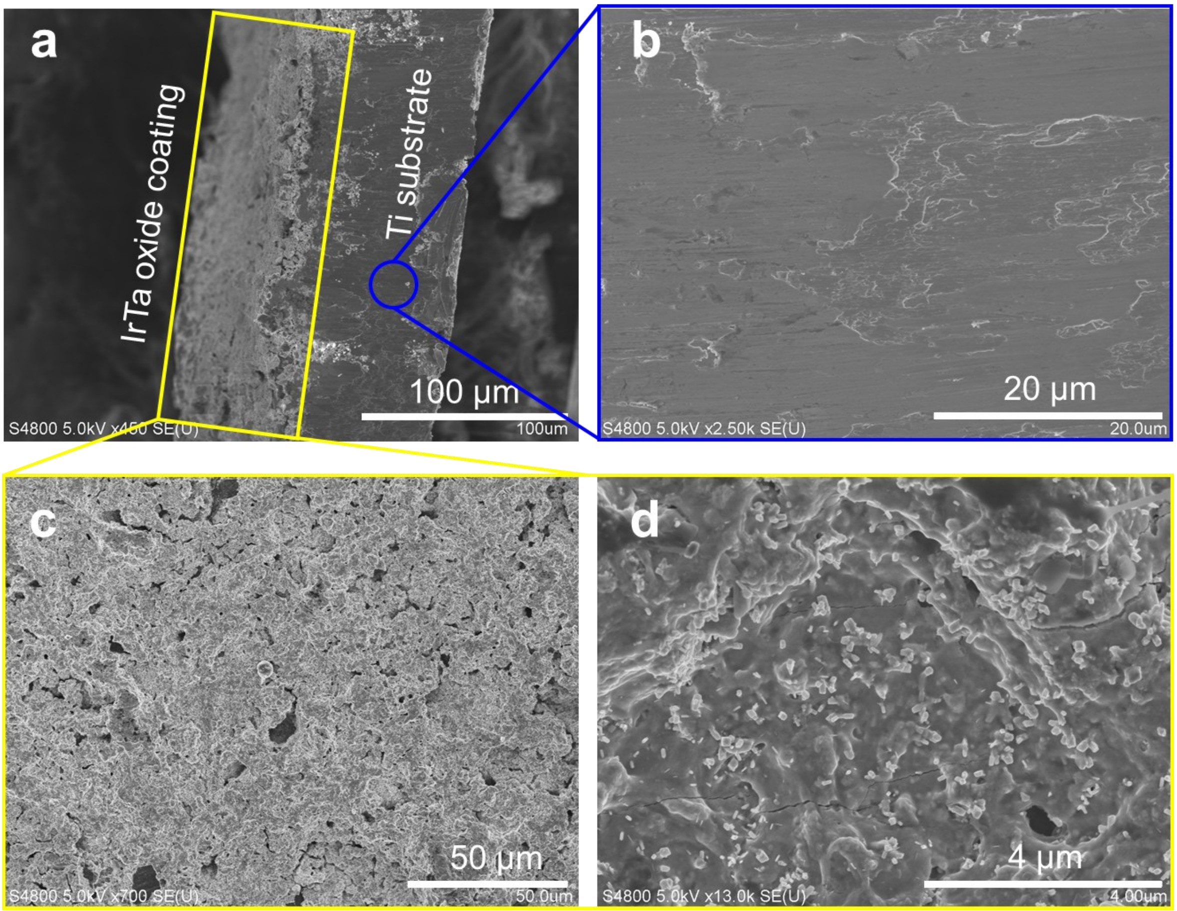

Supplementary Fig. 55 SEM images of IrTaTi electrode. a, Electrode cross-sectional view. **b,** High-resolution image of the Ti substrate. **c-d,** High-resolution images of the IrTa oxide coating.

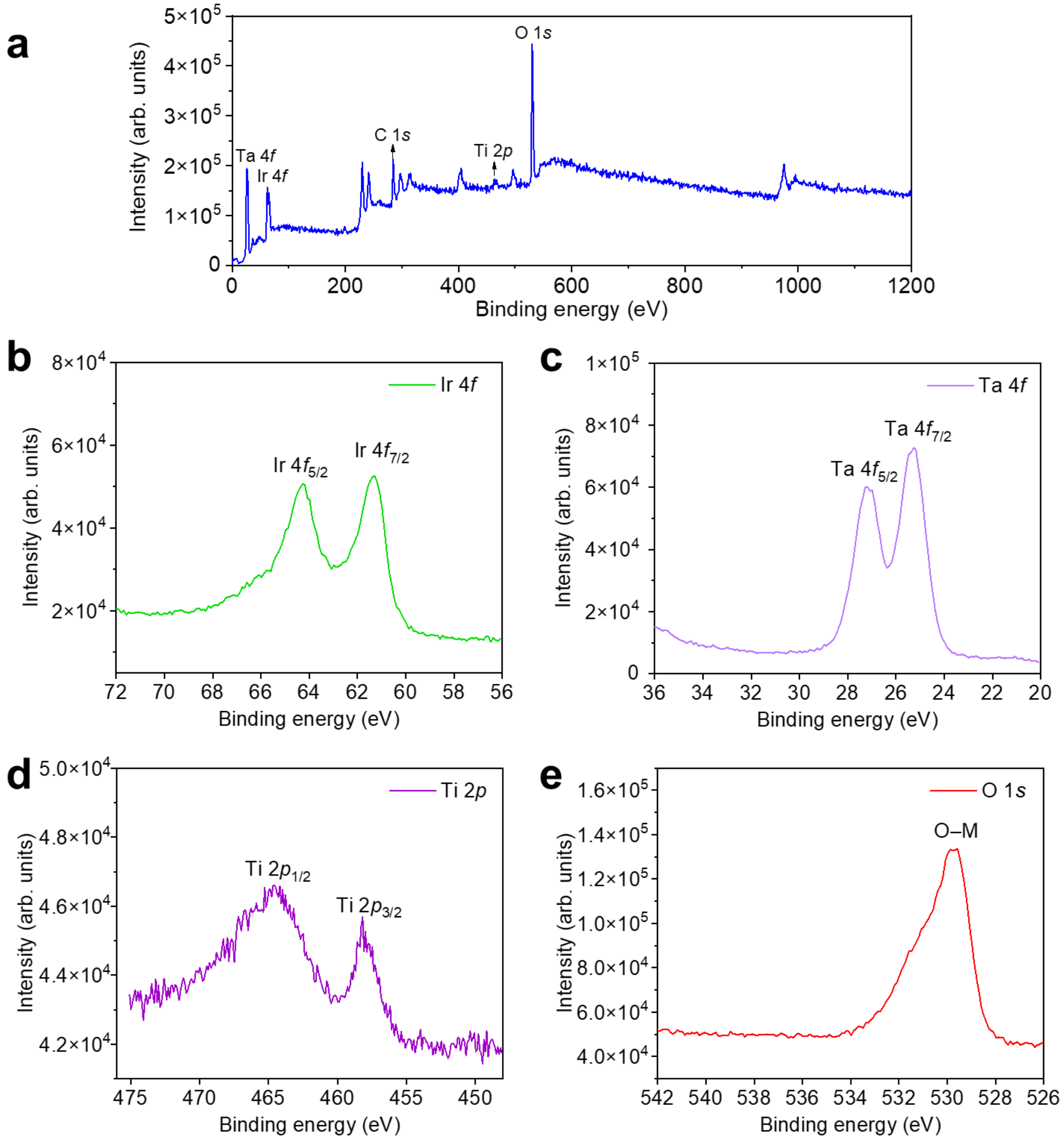

Supplementary Fig. 56 Elemental composition of the IrTaTi oxide anode measured by XPS spectra. a, XPS survey spectrum. **b-e,** High-resolution spectra of Ir 4*f*, Ta 4*f*, Ti 2*p*, and O 1*s*. (Source data for Supplementary Fig. 56 are provided as a Source Data file.)

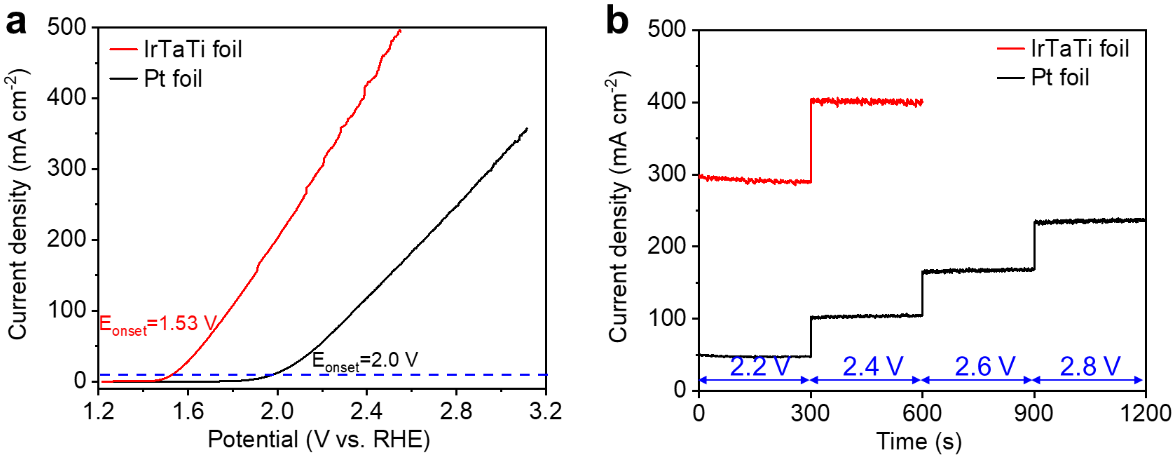

Supplementary Fig. 57 Electrochemical performance comparison of IrTaTi and Pt for the oxygen evolution reaction. a, Linear sweep voltammetry curves at a scan rate of 10 mV s^{-1} in 0.5 M H_2SO_4. **b,** Chronoamperometric responses obtained via multi-step chronopotentiometry. (The reported potentials are not *iR*-corrected. Source data for Supplementary Fig. 57 are provided as a Source Data file.)

SEM images revealed a nanoparticle-based IrTa oxide coating on the Ti substrate, and XPS analysis confirmed its elemental composition, with atomic percentages of Ir (7.0%), Ta (14.4%), Ti (4.9%), and O (73.6%). In the survey spectrum, the primary peaks at 26, 62, 464, and 530 eV were assigned to Ta 4*f*, Ir 4*f*, Ti 2*p*, and O 1*s* core levels, respectively, while minor features at approximately 230 eV, 296 eV, and 313 eV were tentatively attributed to Mo 3*d*, Ir 4*d*, and a carbon-related signal. High-resolution spectral analysis further elucidated the chemical states: the Ir 4*f* doublet (61.4 and 64.3 eV) is characteristic of Ir^{4+} in IrO_2, the Ta 4*f* doublet (25.3 and 27.1 eV) corresponds to Ta^{5+} in Ta_2O_5, the Ti 2*p* doublet (458.2 and 464.5 eV) indicates Ti^{4+} in TiO_2, and the dominant O 1*s* peak at ~530 eV confirms the prevalence of metal-oxygen (O–M) bonding within the coating.

The electrochemical performance of IrTaTi and Pt for the oxygen evolution reaction (OER) was evaluated in 0.5 M H_2SO_4 by linear sweep voltammetry and multi-step chronopotentiometry. The IrTaTi anode exhibited a lower onset potential and required 0.55–0.77 V less overpotentials than that of the Pt electrode to deliver a current density of 100–350 mA cm^{-2}. This significantly higher current density at the same applied potential confirms the superior OER activity of IrTaTi, which contributes to a lower overall cell voltage.

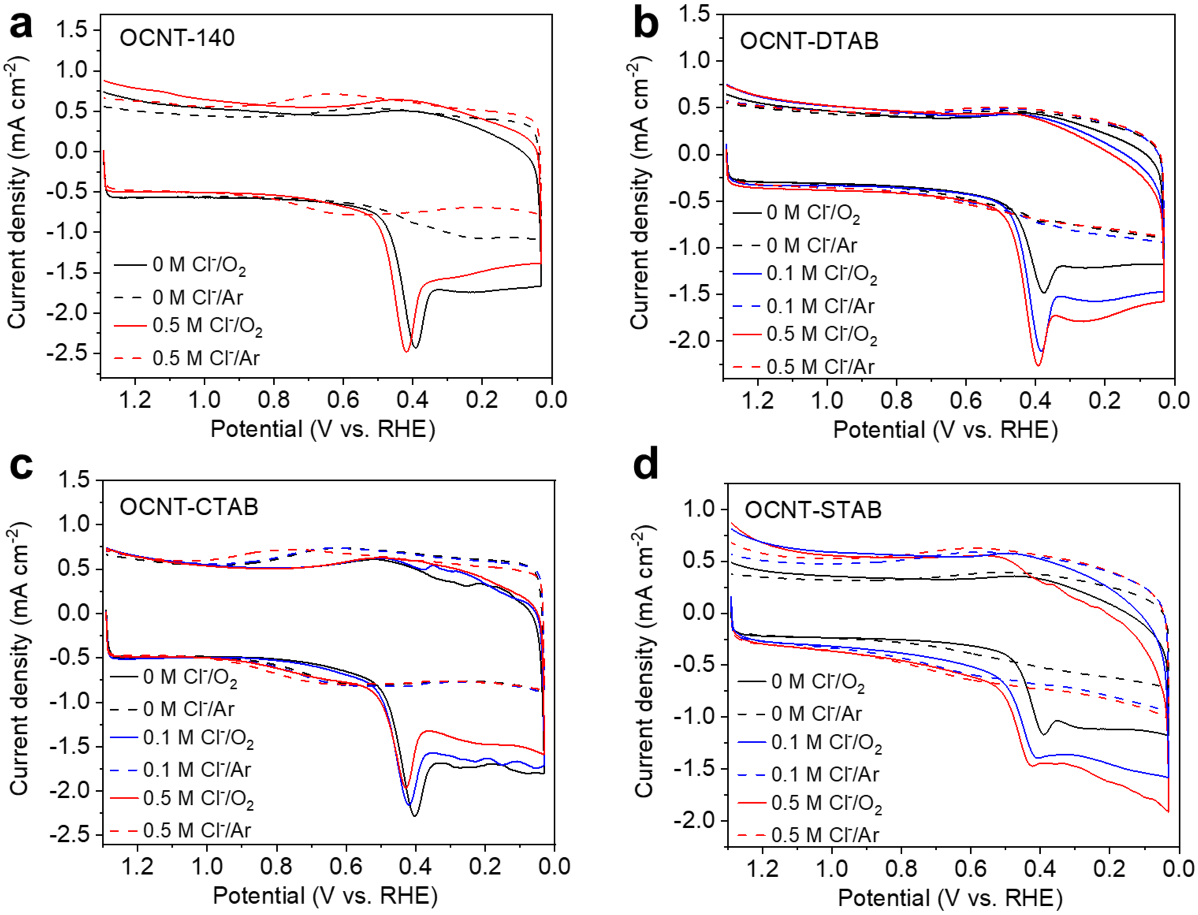

Supplementary Fig. 58 Assessment of the Cl^- effect on the ORR using cyclic voltammetry curves. a, OCNT-140. **b,** OCNT-DTAB. **c,** OCNT-CTAB. **d,** OCNT-STAB. (Scan rate of 50 mV s^{-1}, electrolytes containing different Cl^- ratios, the corresponding ion composition of each solution is listed in Supplementary Table 1. The reported potentials are not *iR*-corrected. Source data for Supplementary Fig. 58 are provided as a Source Data file.)

Supplementary Table 1 Ion compositions of solution used for ORR tests.

Solutions	K^+ concentrations	Ion compositions
0 M Cl^-	0.5 M	0.25 M K_2SO_4
0.1 M Cl^-	0.5 M	0.2 M K_2SO_4+0.1 M KCl
0.5 M Cl^-	0.5 M	0.5 M KCl

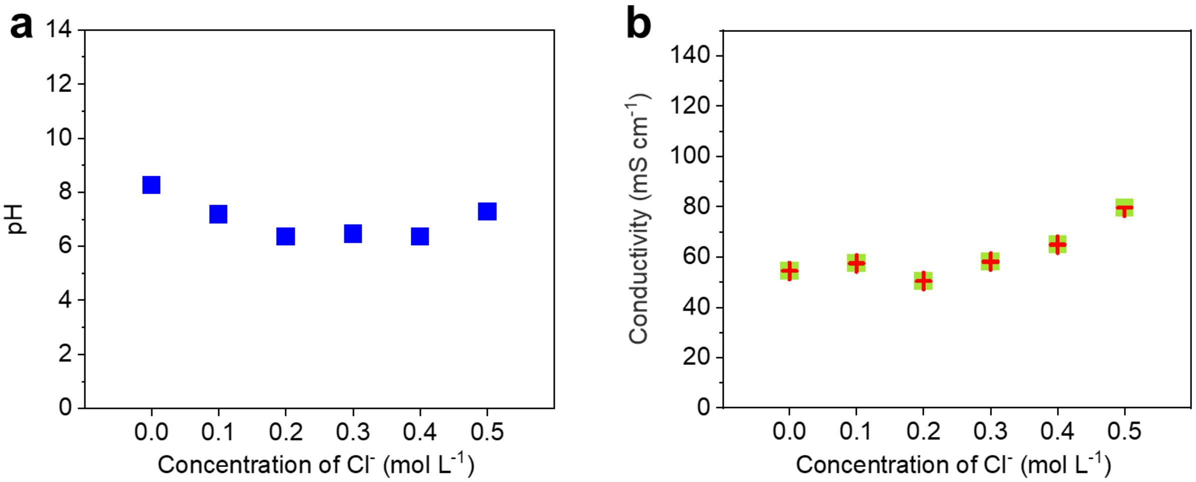

Supplementary Fig. 59 Electrolytes property of containing different Cl^- ratios. a, the pH. **b,** Conducitivity. (Data points for pH represent the average of twice measurements. Data for conductivity represent the average of triplicate measurements, with error bars indicating standard deviations. Source data for Supplementary Fig. 59 are provided as a Source Data file.)

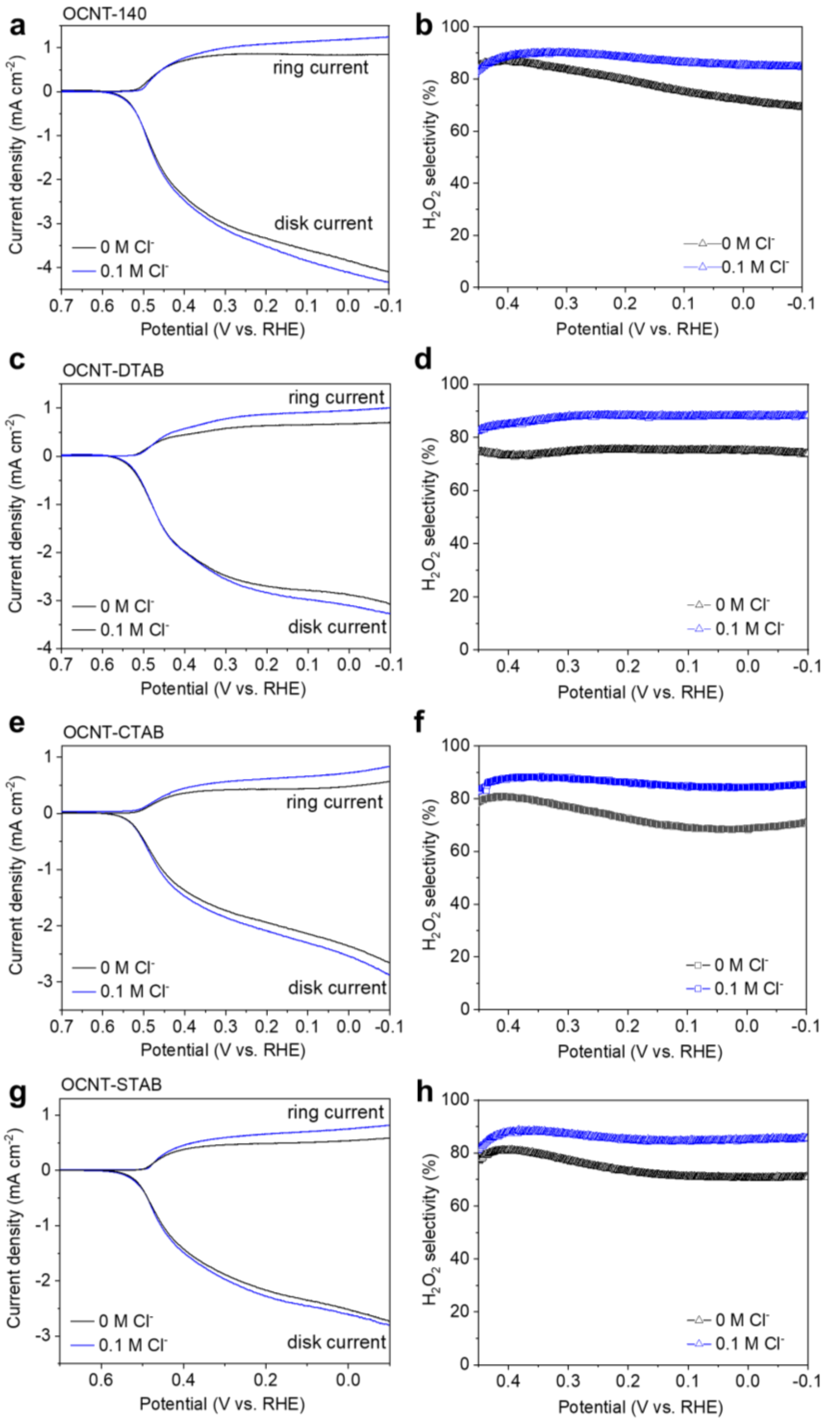

Supplementary Fig. 60 Assessment of the Cl^- effect on the ORR selectivity for H_2O_2 production on OCNT-140, OCNT-DTAB, OCNT-CTAB, and OCNT-STAB catalysts. **T**he left panel (**a, c, e, g**) shows the ORR polarization curve and the corresponding H_2O_2 oxidation current, while the right panel (**b, d, f, h**) shows the calculated H_2O_2 selectivity. (Rotating ring-disk electrode (RRDE) tests were performed at a scan rate of 10 mV s^{-1} in 0.5 M K2SO2 electrolytes with and without 0.1 M Cl^-. The reported potentials are not *iR*-corrected. Source data for Supplementary Fig. 60 are provided as a Source Data file.)

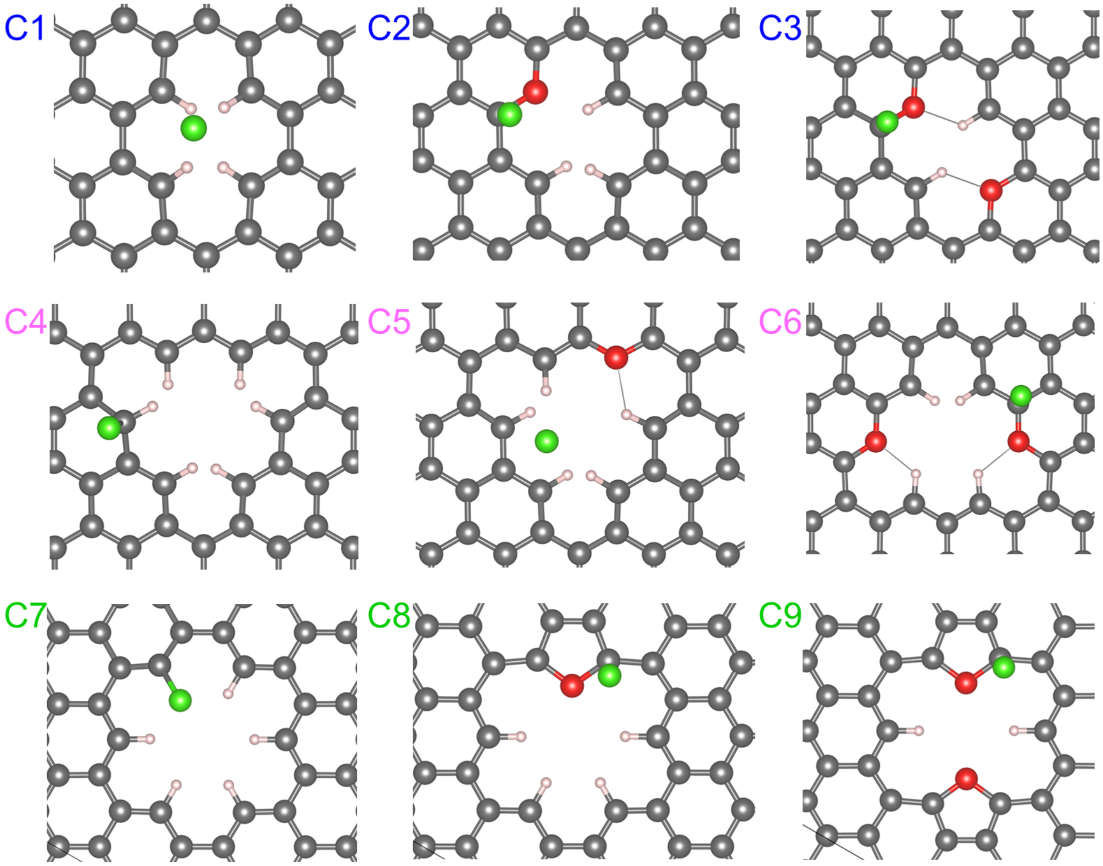

Supplementary Fig. 61 Chloride ion adsorption on different defect carbon and oxygen-modified carbon. (White, gray, red, and green spheres represent H, C, O, and Cl atoms, respectively).

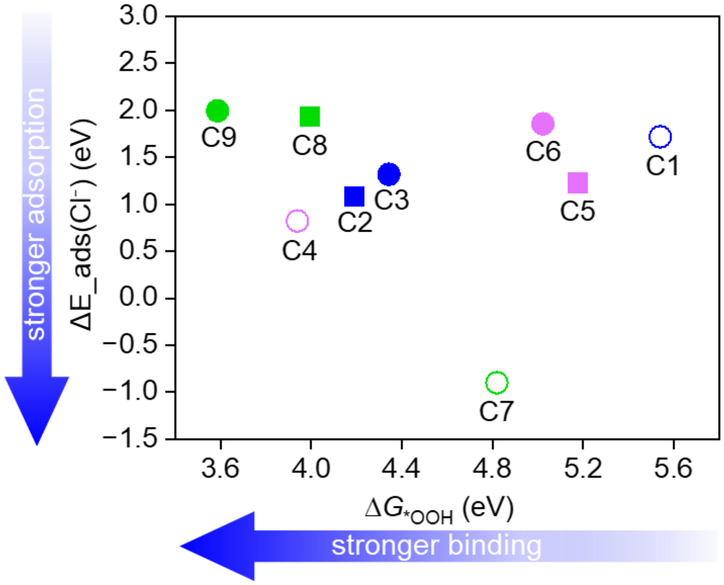

Supplementary Fig. 62 Relationship between Cl^- adsorption energies at 0 V and *OOH binding energies on defect-engineered and oxygen-modified carbon sites.

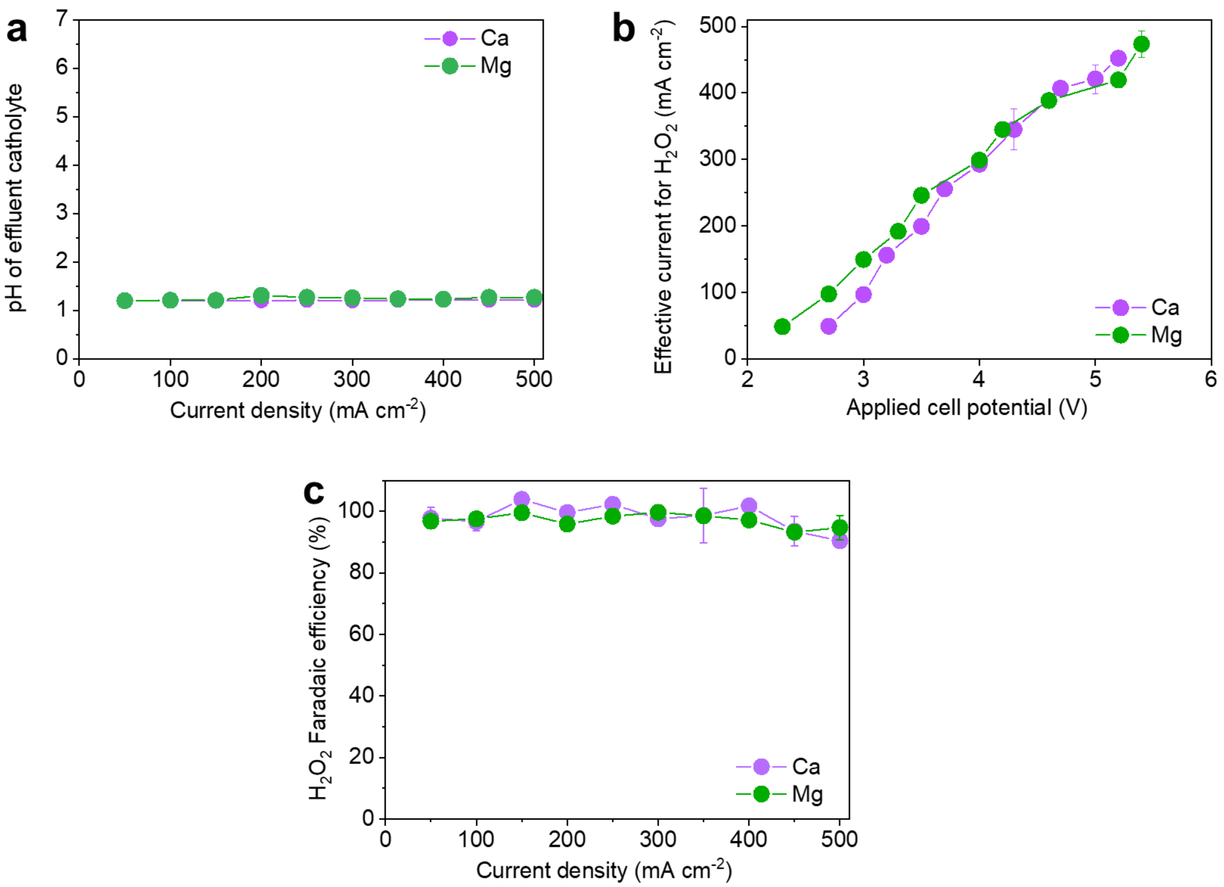

Supplementary Fig. 63 Evaluation of the impact of Ca^{2+} and Mg^{2+} on the performance of H_2O_2 electrosynthesis. a, pH of catholyte. **b,** Effective currents for H_2O_2 production. **c,** Faradaic efficiency for H_2O_2. (Experimental conditions: Cathode: CTAB-OCNT with a working area of 4 cm^2; Catholyte: simulated acidified seawater (0.1 M H_2SO_4+0.3 M K_2SO_4+0.5 g/L $CaSO_4$ or 2.43 g/L $MgSO_4$, pH 1.3); Anolyte: 0.5 M H_2SO_4. Reported data for effective currents and Faradaic efficiency represent the average of triplicate measurements, with error bars indicating standard deviations. Source data for Supplementary Fig. 63 are provided as a Source Data file.)

Seawater-based catholytes present a critical challenge of severe precipitation of Ca^{2+}/Mg^{2+} on the cathode due to the localized alkaline environment generated during the cathodic electrochemical process. To address this, we first demonstrated high-performance H_2O_2 electrosynthesis in an acidic electrolyte containing Ca^{2+} and Mg^{2+} at concentrations matching natural seawater levels.

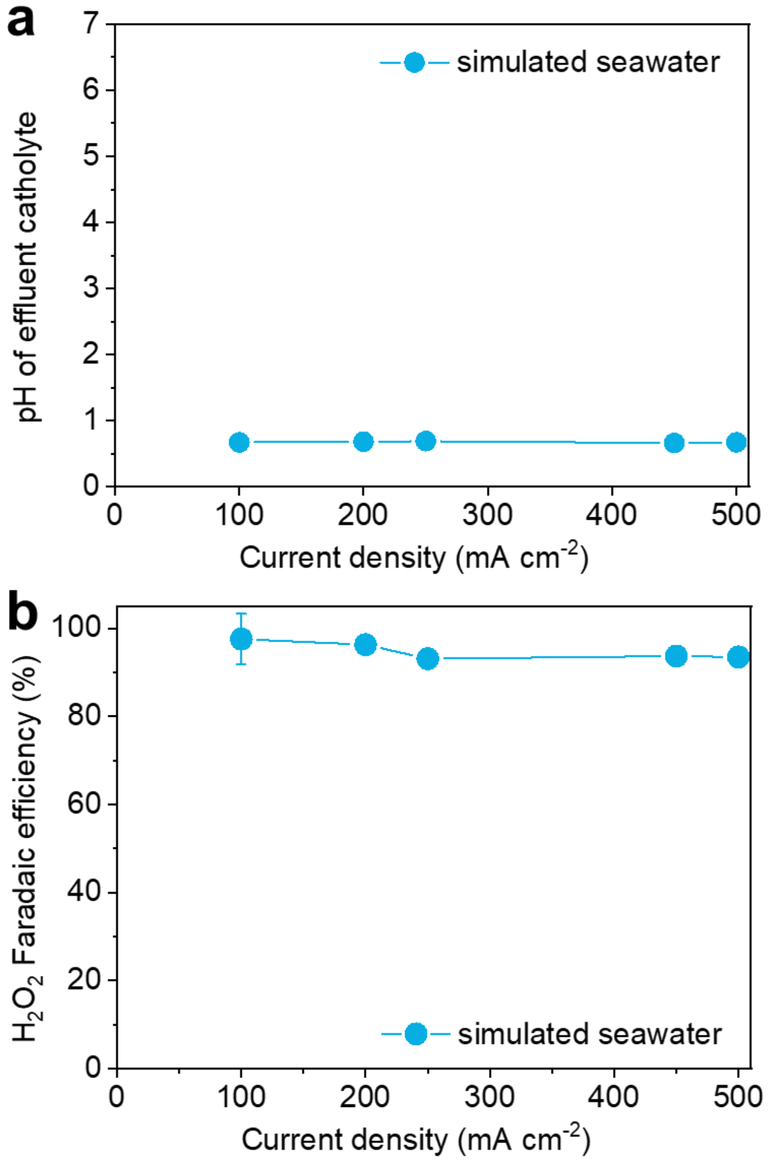

Supplementary Fig. 64 Performance evaluation of H_2O_2 electrosynthesis. a, pH of catholyte. **b,** Faradaic efficiency for H_2O_2. (Experimental conditions: Cathode: CTAB-OCNT with a working area of 4 cm^2; Catholyte: simulated acidified seawater (0.5 M NaCl+0.5 g/L $CaSO_4$ + 2.43 g/L $MgSO_4$, pH 0.8); Anolyte: 0.5 M H_2SO_4. Reported data for Faradaic efficiency represent the average of triplicate measurements, with error bars indicating standard deviations. The pH value was measured once for each point. Source data for Supplementary Fig. 64 are provided as a Source Data file.)

The simulated seawater was prepared based on the composition of natural seawater. To mitigate the precipitation of calcium and magnesium ions when used as the catholyte, the solution was acidified.

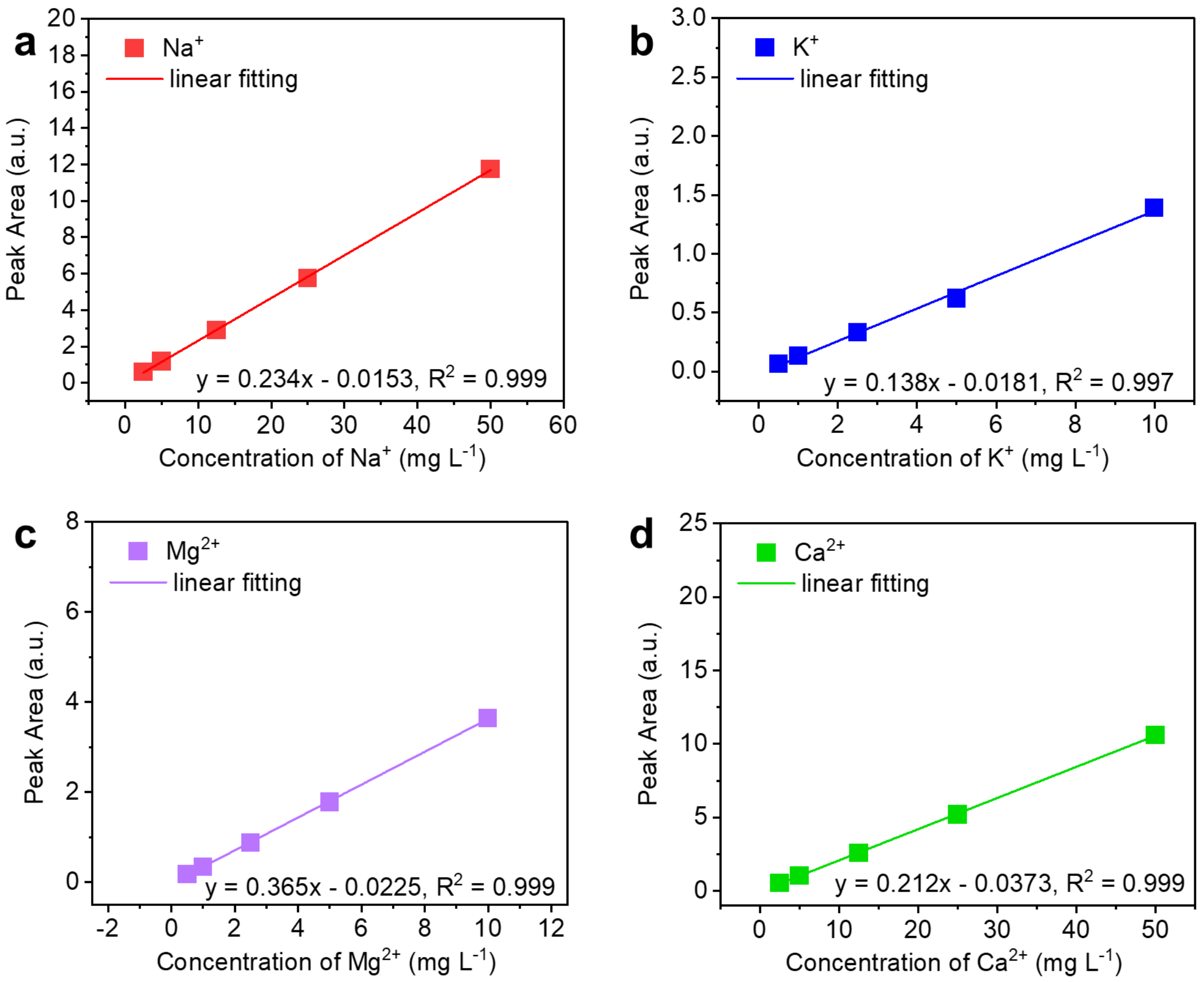

Supplementary Fig. 65 Established linear calibration curves of peak area and ion concentration. a, Na^+. **b,** K^+. **c,** Mg^{2+}. **d,** Ca^{2+}.

Supplementary Table 2 Concentrations of Na^+, K^+, Mg^{2+}, and Ca^{2+} in the raw Yellow Sea seawater sample collected from the Yellow Sea off the coast of Dalian city (39° N, 122° E).

Cations	Concentrations (mg L^{-1})	Concentrations (mM)
Na^+	9854	429
K^+	411	11
Mg^{2+}	1303	54
Ca^{2+}	555	14

Supplementary Table 3 Concentrations of Na^+, K^+, Mg^{2+}, and Ca^{2+} in the raw Yellow Sea seawater sample collected from the Yellow Sea off the coast of Qingdao city (36° N, 120° E) on 22 June 2025.

Cations	Concentrations (mg L^{-1})	Concentrations (mM)

Na^+	9735	423
K^+	396	10
Mg^{2+}	1290	53
Ca^{2+}	544	14

Seawater alkalinity, defined as the equivalent sum of all acid-neutralizing substances (primarily HCO_3^-, CO_3^{2-}, $B(OH)_4^-$, OH^-), was determined according to the Chinese National Standard (GB/T 12763.4-2007). The method involved adding an excess of hydrochloric acid to acidify the sample to pH 1; the alkalinity was then calculated by subtracting the residual acid from the total amount added. For a seawater sample collected from the Yellow Sea off Dalian (39° N, 122° E) with an initial pH of 8.0, this titration yielded a total alkalinity of 176 mmol/kg.

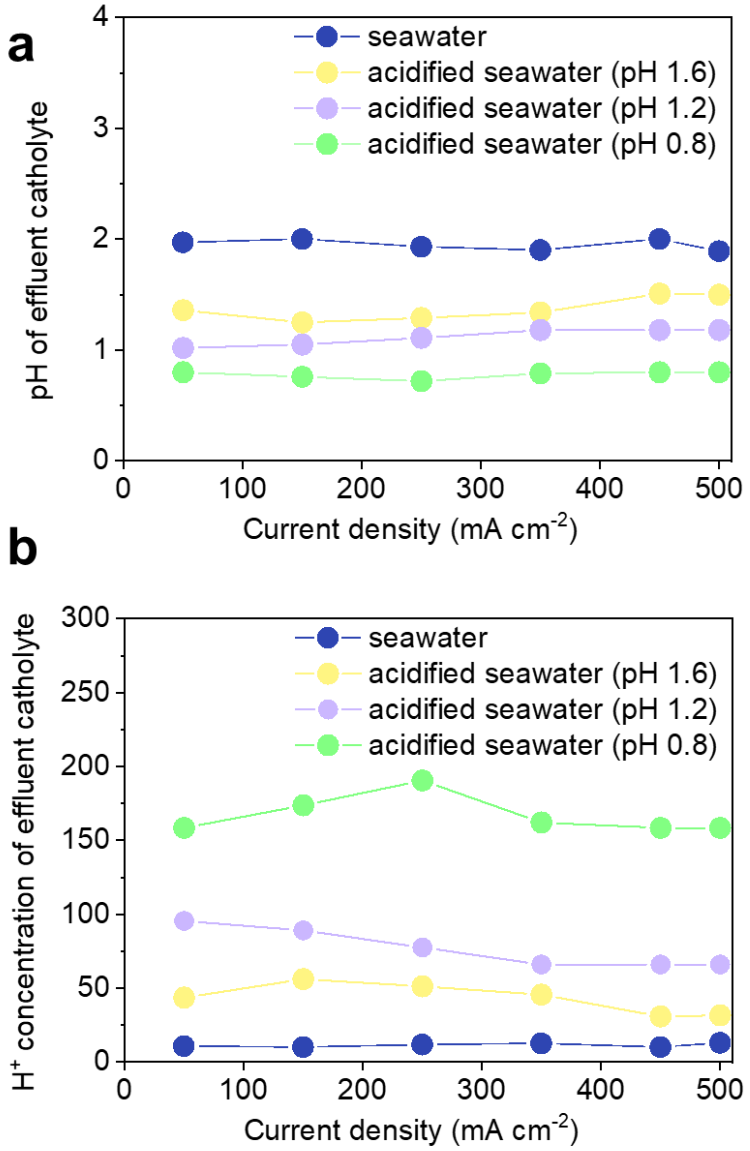

Supplementary Fig. 66 Evaluation of H_2O_2 electrosynthesis in natural seawater. a, pH of catholyte. **b,** H^+ concentration. (Experimental conditions: Cathode: CTAB-OCNT with a working area of 4 cm^2; Catholyte: acidified natural seawater; Anolyte: 0.5 M H_2SO_4. The pH value was measured once for each point. Source data for Supplementary Fig. 66 are provided as a Source Data file.)

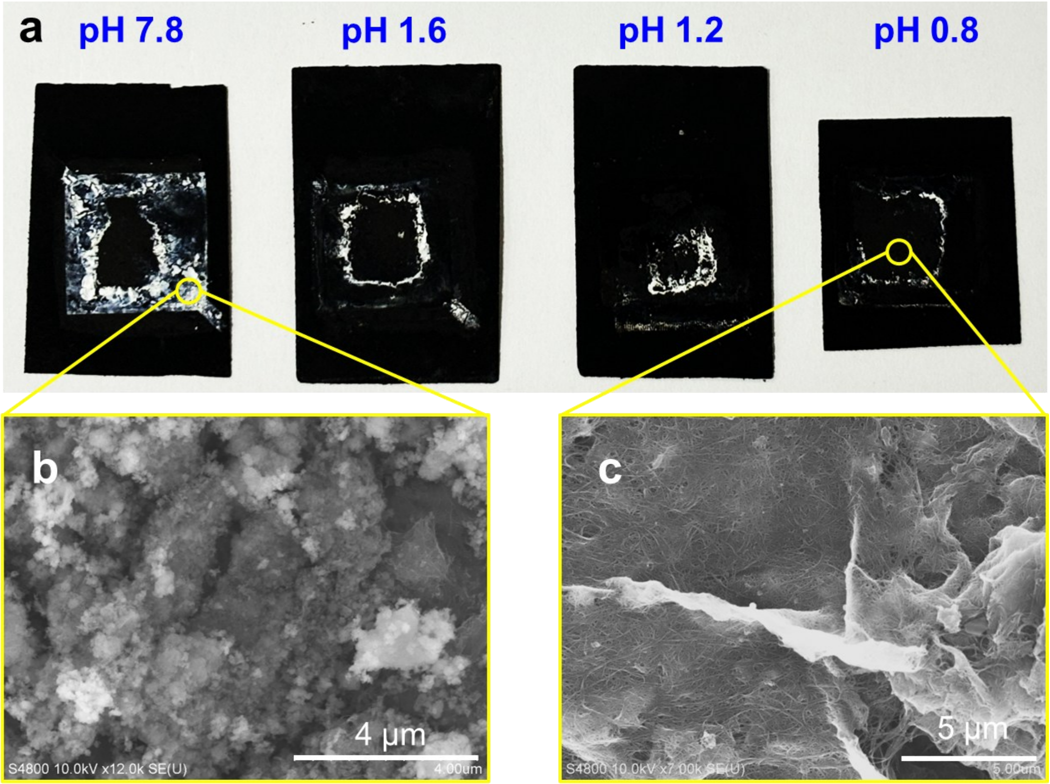

Supplementary Fig. 67 Morphology characterization of electrode. a, Electrode photographs after electrolysis using seawater with different initial pHs as the catholyte. **b-c,** SEM images. (Experimental conditions: Catholyte: natural seawater or acidified seawater; Anolyte: 0.5 M H_2SO_4; Galvanostatic electrolysis for 1 hour at currents ranging from 50 to 500 mA cm^{-2}.)

As shown in the SEM images, the region identified as the 'white precipitate area' is characterized by a dense coverage of nanoparticles on the electrode surface. Such a layer is consequently detrimental to the electrode's high-current performance and long-term stability for seawater electrolysis toward H_2O_2 production.

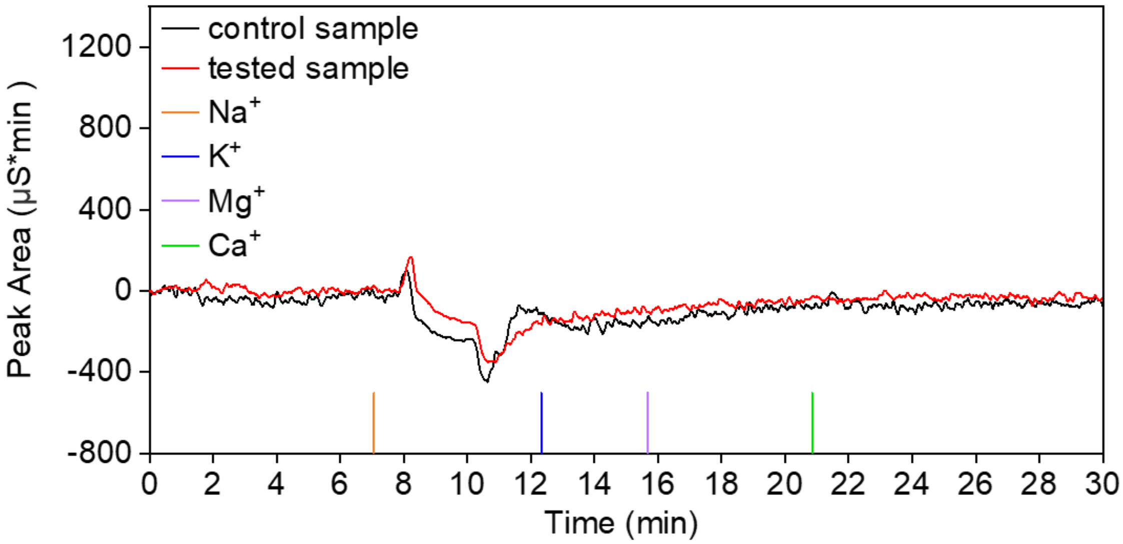

Supplementary Fig. 68 Ion chromatographic analysis of the sample. The signal peaks were compared with those of standard Na^+, K^+, Mg^{2+}, and Ca^{2+}. (The tested sample was the anode effluent from a system operated for 2 h at 500 mA cm^{-2}, with seawater as the cathode and 0.5 M H_2SO_4 as the anode. The control was the anode effluent from a system operated under the same duration and current, using 0.3 M K_2SO_4 + 0.1 M H_2SO_4 as the cathode and 0.5 M H_2SO_4 as the anode.)

No detectable Cl^- was found within the instrument's detection limit. Both Cl^- and metal ions (Na^+, K^+, Mg^{2+}, Ca^{2+}) weren't detected in the anode effluent above the instrument's detection limit during H_2O_2 electrosynthesis. The absence of these species indicates negligible ion crossover, thereby minimizing the risk of adverse side reactions at the anode.

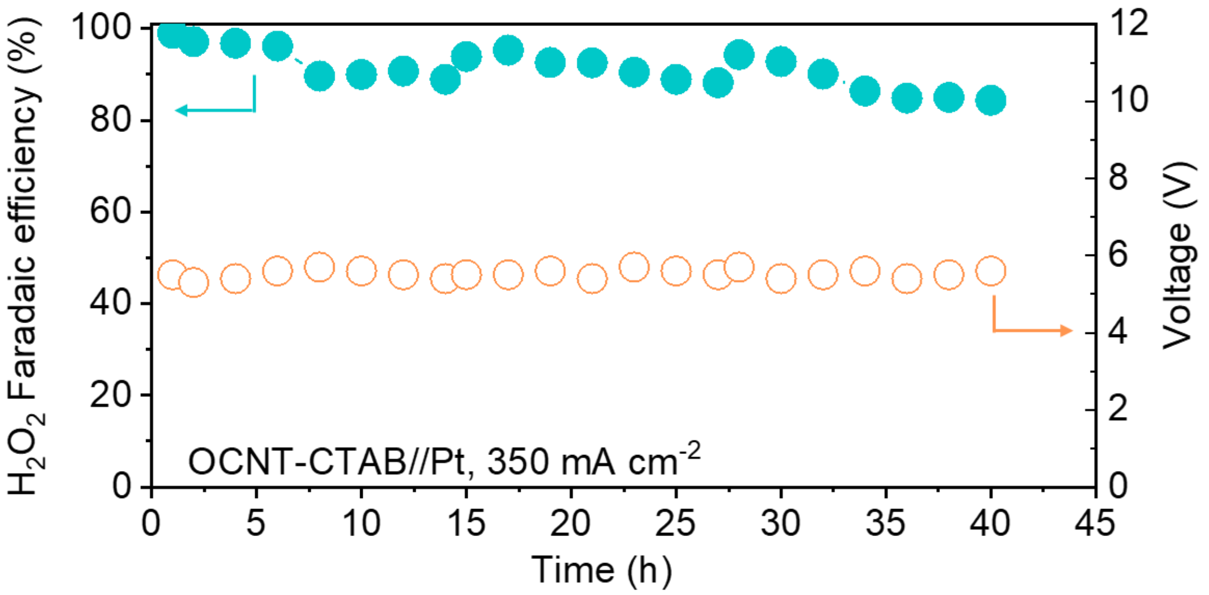

Supplementary Fig. 69 H_2O_2 Faradaic efficiency of the OCNT-CTAB electrode during the stability test. (Test was conducted at 350 mA cm^{-2} using acidified natural seawater with initial pH of 0.8 as the catholyte and 0.5 M H_2SO_4 as the anolyte. Source data for Supplementary Fig. 69 are provided as a Source Data file.)

After a period of operation, the electrode experiences a slight decrease in Faradaic efficiency. This is primarily because a small amount of electrolyte permeates to the back of the electrode, which slightly impedes oxygen transport and leads to efficiency variations. This issue was readily mitigated through a drying procedure, which restored the system to its original performance.

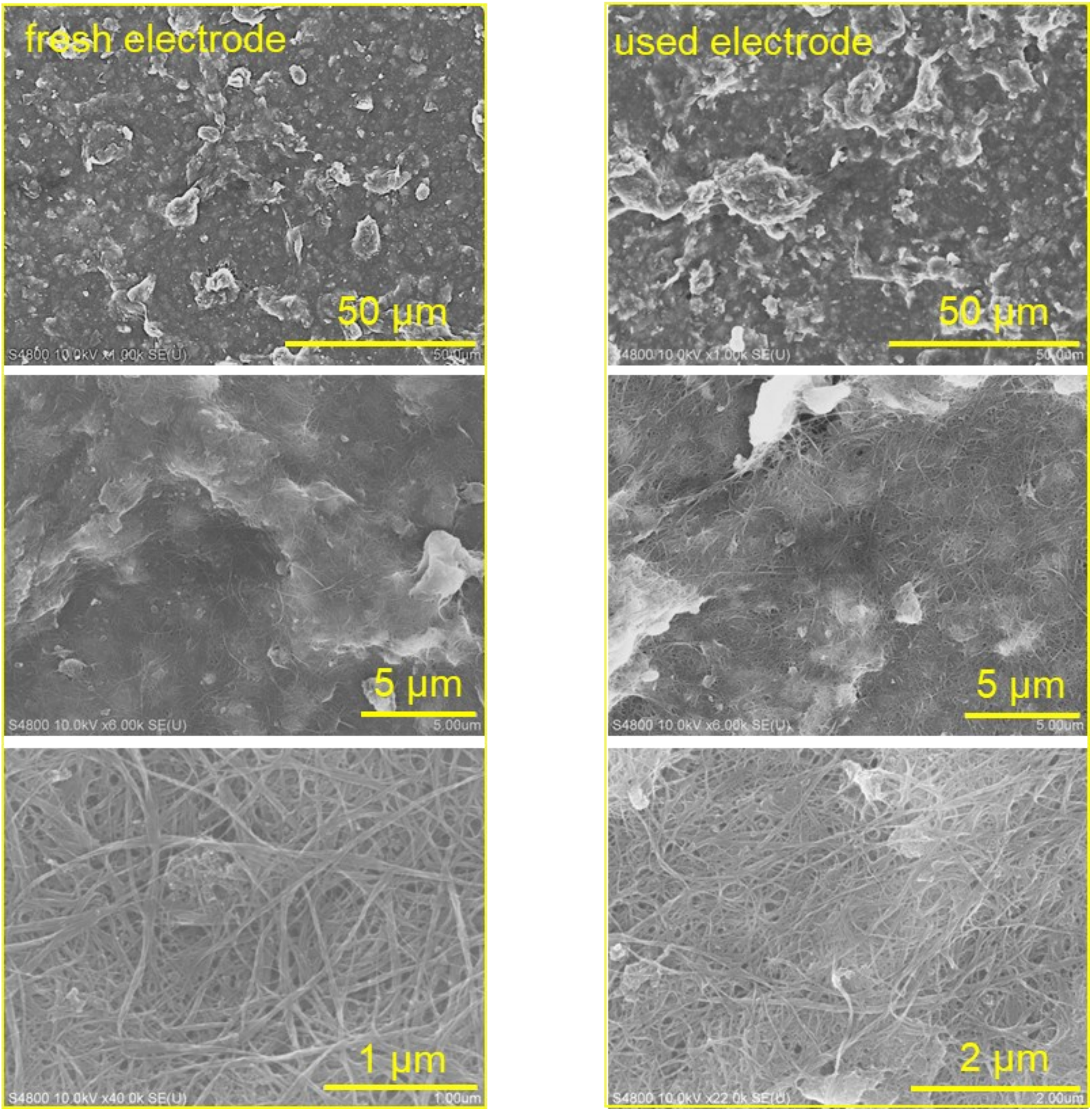

Supplementary Fig. 70 Photographs of the OCNT-CTAB electrode before and after electrolysis. (Experiment was conducted at 300 mA cm^{-2} for 40 hours using acidified natural seawater with initial pH of 0.8 as the catholyte and 0.5 M H_2SO_4 as the anolyte.)

Compared with a fresh electrode, the surface of the electrode after electrolysis in acidic seawater was virtually free of observable precipitates or impurities.

Supplementary Table 4 Recently reported high-performance electrocatalysts and their current densities and Faradaic efficiencies for electrochemical 2e⁻ ORR to produce H_2O_2 in acidic electrolytes.

Catalysts	Currents density (mA cm^{-2})	Electrode area (cm^{-2})	FEs for H_2O_2 (%)	Production rate (μmol/cm^2/h)	Catholyte	References
Se_2-Pt	250	1	89.2%	4.16 mol g_{cat}^{-1} h^{-1}	0.1 M $HClO_4$	1
$CoSe_2$	63	1	>90%	900 μmol cm^{-2} h^{-1}	0.5 M H_2SO_4	2
TiC	50–500	4	84–100%	856–7842 μmol cm^{-2} h^{-1}	0.1 M H_2SO_4 +0.3 M K_2SO_4	3
CoN_4/VG	8.4	25	~70%	304 μmol cm^{-2} h^{-1}	0.1 M $HClO_4$	4
$Co_2N_4O_2$	400	1	78%	/	0.1 M $HClO_4$	5
CoN_4/C-O-C	500	1	~78%	49 mol g_{cat}^{-1} h^{-1}	0.5 M H_2SO_4	6
Co_1-NBC	350	/	~80%	5962 μmol cm^{-2} h^{-1}	0.5 M H_2SO_4	7
Co_1-HCNF	400	5	~80%	6140 μmol cm^{-2} h^{-1}	0.5 M H_2SO_4	8
carbon black	150	1	90%	/	0.1 M H_2SO_4	9
carbon black	100–400	1	50–92%	~6500 μmol cm^{-2} h^{-1}	0.1 M H_2SO_4 +0.05 M Na_2SO_4	10
defective carbon	~30	4	100%	/	0.1 M $HClO_4$	11
OCNT-CTAB	500	4	~90%	~8400 μmol cm^{-2} h^{-1}	0.1 M H_2SO_4 +0.3 M K_2SO_4 pH ~1	This work
	1125	4	83%	17438 μmol cm^{-2} h^{-1}	0.1 M H_2SO_4 +0.3 M K_2SO_4 pH ~1	This work

This study focuses on systems that sustain acidic H_2O_2 production for meaningful comparison. The systems for comparison all was tested in a double-cell configuration. In systems with acidic/neutral anolytes containing competing cations (e.g., alkali metals), these cations migrate to the cathode instead of protons. This leads to insufficient proton supply at the cathode and catholyte alkalization, especially at high current densities, thereby preventing the production of acidic H_2O_2 solutions—even when acidic electrolytes are used in the cathode compartment. Moreover, the pH difference between the anode and cathode provides an additional potential difference as a driving force for the reaction[12], making it difficult to clearly distinguish the contribution of this process.

Supplementary Table 5 Recently reported high-performance electrocatalysts and their current densities and Faradaic efficiencies for electrochemical 2e⁻ ORR to produce H_2O_2 in simulated or natural seawater.

Catalysts	Onset potential (V vs RHE)/ H_2O_2 selectivity	Currents density (mA cm^{-2})	FEs (%)	Production rate	Catholytes	References
Co-N-C	0.55 V/93.7% (0.5 M NaCl)	50	95.6%	4.5 mol g_{cat}^{-1} h^{-1}	0.5 M NaCl	[13]
$NiPS_3$	0.564 V/82% (0.5 M NaCl)	60	84%	/	seawater	[14]
Nanocarbon	0.57 V/95% (0.5 M NaCl)	~50	80 %	1200 mg L^{-1} h^{-1}	simulated seawater	[15]
$Ni(OH)_2$/CNT	~0.45 V/~90% (3.6 wt.% NaCl)	100	~80 %	1420 µmol cm^{-2} h^{-1}	natural seawater	[16]
OCNT-CTAB	~0.5 V/90% (0.1 M KCl+0.3 M K_2SO_4)	500	~89%	~8400 µmol cm^{-2} h^{-1}	acidified seawater	This work

The operating costs for the electrochemical synthesis of H_2O_2 in this work was evaluated based on Chinese market conditions, which primarily includes electricity consumption, electrolyte consumption, oxygen and water consumption costs.

C = C_1 + C_2 + C_3 + C_4+ C_5 + C_5 + C_6

where C represents the total operating cost per kilogram of H_2O_2. **C_1**, **C_2**, **C_3**, and **C_4** denote the electricity cost, electrolyte cost, oxygen cost, and water cost per kilogram of H_2O_2, respectively. **C_5** and **C_6** represents the cost of concentrated acid for acidifying seawater, and neutralizing the acidified seawater. All monetary values are in US dollars ($).

1. **Electricity Cost C_1**

The electricity cost per kilogram of H_2O_2 is calculated as:

$$\mathbf{C_1}=\frac{U\times I}{c\times v}\times A \quad (1)$$

where:

C_1: Electricity cost ($/kg H_2O_2)

U: Cell voltage (V), *I*: Current (A)

c: Synthesized H_2O_2 concentration (wt%), *v*: H_2O_2 production rate (mL h^{-1})

A: Industrial electricity price in China = 0.071 $/kWh

The hydraulic power required to transport the catholyte and anolyte is typically calculated using the formula:

$$\mathrm{P}=\frac{(\rho\times g\times \mathrm{H}\times \mathrm{Q})}{\eta} \quad (2)$$

where:

ρ is the fluid density (1000 kg/m^3 for water),

g is the gravitational acceleration (9.81 m/s^2),

H is the head (approximately 0.2 m),

Q is the flow rate (sum of anode and cathode flow rates: 41.4 mL/h),

η is the pump efficiency (taken as 0.7).

In this work, the estimated power of the peristaltic pump is approximately 2 mW, which is negligible.

2. **Catholyte Cost C_2**

The electrolyte cost per kilogram of H_2O_2 is calculated as:

$$\mathbf{C_2}=\frac{c(\mathrm{M_2SO_4})\times \mathrm{M}(\mathrm{M_2SO_4})}{c/100}\times \mathrm{P}(\mathrm{M_2SO_4})+\frac{c(\mathrm{H_2SO_4})\times \mathrm{M}(\mathrm{H_2SO_4})}{c/100}\times \mathrm{P}(\mathrm{H_2SO_4}) \quad (3)$$

where:

C_2: Electrolyte cost ($/kg H_2O_2)

c: Synthesized H_2O_2 concentration (wt%),

c(M_2SO_4)/*c*(H_2SO_4): Electrolyte and H_2SO_4 concentration (mol L^{-1})

$M(M_2SO_4)/M(H_2SO_4)$: Molecular weight (K_2SO_4 = 174 g mol^{-1}, Na_2SO_4 = 142 g mol^{-1}, H_2SO_4 = 98 g mol^{-1})
$P(M_2SO_4)$: Market price of electrolyte ($/t) in China, industrial K_2SO_4: 500 $/t, industrial Na_2SO_4: 57.1 $/t, H_2SO_4: 128.6 $/t.
The anode electrolyte is recycled during electrolysis and is not included in this calculation. Notably, the cost of the catholyte is somewhat overestimated, as the cost reduction resulting from the recovery and reuse of electrolyte salts has not been factored in.

3. **Oxygen Cost C_3**

The oxygen cost per kilogram of H_2O_2 is calculated as:

$$\mathbf{C_3} = \frac{v(O_2) \times P(O_2)}{c/100 \times v} \tag{4}$$

where:
C_3: Oxygen cost ($/kg H_2O_2)
c: Synthesized H_2O_2 concentration (wt%), *v*: H_2O_2 production rate (L h^{-1})
$v(O_2)$: Oxygen flow rate = 1.2 L h^{-1}
$P(O_2)$: Oxygen price ($/L), industrial liquid oxygen price in China: 64.29 $/t (1 tonne liquid O_2 = 1008 m^3 gaseous O_2).

4. **Water Cost C_4**

The water cost per kilogram of H_2O_2 is calculated as:

$$\mathbf{C_4} = \frac{P(H_2O)/1000}{c/100} \tag{5}$$

where:
C_4: Water cost ($/kg$H_2O_2$)
c: Synthesized H_2O_2 concentration (wt%)
$P(H_2O)$: Industrial water price in China = 0.57 $/t

5. **Other cost considerations**

To assess the acid cost for H_2O_2 electrolysis in seawater, the required amount of concentrated hydrochloric acid (37 wt%, analytical grade) to acidify seawater to pH 1.2 and 1.6 was determined on a per-liter basis.

$$\mathbf{C_5} = \frac{V_{add} \times \eta \times \rho}{c \times M} \times P \tag{6}$$

where:
C_5: the cost of concentrated hydrochloric acid consumed in producing 1 kg H_2O_2 (on a 100% basis), with units of $/kg H_2O_2.
V_{add}: the amounts of concentrated hydrochloric acid (37wt%) used for acidification, where 5.7 L and 16.6 L per ton of seawater with initial pH 8.0 were consumed to reach pH 1.6 and 1.2, respectively, as calculated from **Supplementary Table 6**.
η: the conversion factor from the amount of 37 wt% HCl to the equivalent amount of 31 wt%, with an approximate value of 1.2.

ρ: mass density of the concentrated hydrochloric acid (31 wt%: 1.152 kg L^{-1}; 37 wt%: 1.179 kg/L).

c: synthesized H_2O_2 concentration (mmol L^{-1}).

M: molecular weight (H_2O_2 = 34 g mol^{-1}).

P(concentrated hydrochloric acid): The price of concentrated hydrochloric acid is closely related to its purity grade. Although industrial-grade concentrated hydrochloric acid is available at a very low cost ($1.43/t), its significant content of Fe^{3+} interferes with H_2O_2 synthesis, making it unsuitable for use in this system. A higher-purity grade, suitable for chemical synthesis (often at 31% concentration with heavy metals content < 0.005%), is priced around $14.3/t. It is important to note that the market price of concentrated hydrochloric acid is highly volatile and closely tied to the dynamics of the chlor-alkali industry. This assessment uses the current Chinese market price (as of December 2025) as the evaluation baseline.

We adopted a similar approach to assess the cost of using concentrated sulfuric acid as the acid agent and the cost (**C6**) of neutralization using $Ca(OH)_2$.

$$\mathbf{C_6} = \frac{m_{\mathrm{add}}}{c \times M} \times P \quad (7)$$

where:

$\mathbf{C_6}$: the cost of $Ca(OH)_2$ consumed in producing 1 kg H_2O_2 (on a 100% basis), with units of $/kg$H_2O_2$.

m_{add}: the amounts of $Ca(OH)_2$ used for neutralizing the acidified seawater, where 1.8 kg and 8.4 kg per ton seawater were consumed to reach pH 7.7-7.9 and 1.2, respectively.

c: synthesized H_2O_2 concentration (mmol L^{-1}).

P($Ca(OH)_2$): The price of industrial-grade $Ca(OH)_2$ (≥90% purity) is estimated to be $57/t based on the current Chinese market price.

Supplementary Table 6 The amount of concentrated HCl or H_2SO_4 consumed to adjust the pH of seawater (500 mL) from an initial pH of about 8.0 to pH 1.6, 1.2 or 1.0 (these experiments were repeated 2-4 times, and the data represent the mean values).

HCl system	Consumed concentrated HCl (mL)	H_2SO_4 system	Consumed concentrated H_2SO_4 (mL)
pH 1.6	2.85	pH 1.6	0.53
pH 1.2	8.29	pH 1.2	1.25
pH 1.0	11.4	pH 1.0	5.87

Supplementary Table 7 The amount of $Ca(OH)_2$ consumed to re-neutralize seawater (500 mL) to a near-neutral pH (7.1-8.4) (these experiments were repeated 2-4 times, and the data represent the mean values).

HCl system	Consumed $Ca(OH)_2$ (g)	H_2SO_4 system	Consumed $Ca(OH)_2$ (g)
pH 1.6	0.90	pH 1.6	0.69
pH 1.2	4.22	pH 1.2	1.90

Discussion:

We calculated the cost of using concentrated acid to acidify seawater to pH 1.2 and 1.6, taking hydrochloric acid or concentrated sulfuric acid as examples. The minimum cost for producing per kg of H_2O_2 is \$0.031 (for acidification to pH 1.6) and \$0.076 (for acidification to pH 1.2). When using calcium hydroxide to neutralize the liquid at pH 1.6 and pH 1.2, the costs are \$0.03 and \$0.047, respectively. The overall costs are \$0.06 and \$0.186, respectively. When sulfuric acid is used as the acidifying agent, the costs are \$0.093 and \$0.193, respectively. Between these two options, the former is less expensive, but the latter offers higher operational safety due to the volatility of hydrochloric acid, whereas concentrated sulfuric acid is more stable.

Furthermore, one point needs to be raised it that: acidic H_2O_2 is more stable than alkaline H_2O_2, particularly in environments with temperatures exceeding 50°C. Additionally, acidic H_2O_2 can be directly utilized in the Fenton process for industrial wastewater treatment, which is currently the most efficient and economical method for industrial wastewater treatment. A key characteristic of this process is that the aqueous solution must be acidified, and iron ions must be neutralized and removed after the reaction. Therefore, if intended for such a process, the costs associated with acid addition for acidic H_2O_2 and subsequent neutralization should not be considered as additional expenses.

Techno-economic assessments (TEA)

Regarding the TEA analysis of the electrolysis technology, the U.S. DOE developed and used the H2A Production Model (H2A) framework for the techno-economic analysis of hydrogen production pathways. The model includes major inputs such as capital costs, operation and maintenance, different fuel types, and financial considerations such as dollar year, plant life, and internal rate of return. Therefore, our cost analysis for the electrosynthesis of H_2O_2 references the calculation principles of the H2A model.
The technical and economic factors for H_2O_2 electrosynthesis include: operating costs, capital equipment expenditure, and labor cost.

1. The analysis is conducted with the production cost per unit weight of H_2O_2 ($/kgH$_2O_2$) as the metric. H_2O_2 electrosynthesis at the cathode, at its most basic level, requires O_2, H_2O and electricity to produce H_2O_2 along with by-product O_2 at the anode which may serve as a supply of cathode. The system boundary encompasses raw materials (water, oxygen, electrolyte replenishment), and electrical energy consumption. There are 8000 hours of operation every year, and the rest is used for equipment maintenance.

Supplementary Table 8 Operating costs for H_2O_2 production using OCNT-CTAB as the cathode and IrTaTi as the anode with 4 cm^2 working area.

Current density mA cm^{-2}	Yield rate *10^{-3} kg/h	Electricity cost kwh/kgH$_2$O$_2$	O_2 cost $/kgH$_2O_2$	H_2O cost $/kgH$_2O_2$	Total operating cost $/kgH$_2O_2$
250	0.52859	0.40539	0.1287	0.05405	0.58814
500	1.01293	0.59234	0.06716	0.02821	0.68771
750	1.58307	0.71741	0.04297	0.01805	0.77843
1000	1.98707	0.92023	0.03423	0.01438	0.96885

Supplementary Table 9 Estimated operating costs for H_2O_2 production per year when working area was scaled up 500 m^2.

Current density mA cm^{-2}	Yield rate kg/h	Output ton/year	Total operating cost $/year
250	660	5285	3108849
500	1266	10129	6966020
750	1978	15830	12323091
1000	2483	19870	19251727

Note: the calculations of electricity cost, O_2 cost and H_2O cost are shown above.

2. Capital equipment expenditure includes the electrolyzer stack and its supporting facilities such as power supplies, pumps, compressors, separation membranes/columns, etc. The electrolyzer stack cost accounts for 65% of the total capital expenditure, with the balance of plant (BOP) comprising the remaining 35%. Their installation and infrastructure costs are typically a multiple of the equipment cost,

e.g., 1.2 times. It also includes product separation and purification costs, and the capital cost of membrane separation is estimated based on distillation.

$$\text{Electrolyzer cost} = 500\ \text{m}^2 \times \$1619\ \text{m}^{-2} \times 1.2 = \$971{,}400 \quad (8)$$

$$\text{BOP cost} = \text{Electrolyzer} \div 65\% \times 35\% = \$523{,}061 \quad (9)$$

$$\text{Separation cost} = \$10664000 \times \left(\frac{\text{production rate L min}^{-1}}{1000\ \text{L min}^{-1}}\right)^{0.7} \times 0.7 \times 0.1 = \$1{,}657{,}346 \quad (10)$$

$$\text{Equipment Cost} = \text{Base Cost} \times \left(\frac{\text{Throughput}}{\text{Base Throughput}}\right)^{\text{Scaling Factor}} \times \text{Process Cost Correction Factor} \quad (11)$$

This part is estimated referring "H2A Production Model Version 3 2018 User Guide Draft". Process Cost Correction Factor: refers to the cost adjustment coefficient of a specific process (membrane separation) relative to the benchmark process (distillation). If the scaling factor exponent is 1.0, it means that the growth rate of equipment cost is exactly proportional to the growth rate of plant capacity. The scaling factor exponent is typically 1.0 or less. If the scaling factor exponent is 0.7, then the increase in equipment cost is proportional to the increase in plant capacity raised to the 0.7 power. Scaling factor reference (distillation column): The scaling factor for the distillation method typically ranges from 0.6 to 0.7.

$$\text{Depreciation cost} = \frac{(\text{Electrolyzer cost} + \text{BOP cost} + \text{Separation cost}) \times 3.25\%}{1 - \frac{1}{(1+3.25\%)^{20}}} = \$233{,}452/\text{year} \quad (12)$$

The maintenance cost is assumed 2.5% of capital cost per year (from H2A):

$$\text{Maintenance cost} = \text{Depreciation cost} \times 2.5\% = \$5{,}836/\text{year} \quad (13)$$

$$\text{Capital equipment expenditure} = \text{Depreciation cost} + \text{Maintenance cost} = \$239{,}288/\text{year} \quad (14)$$

3. Labor cost
   According to 2025 data from the National Bureau of Statistics of China, the average annual income for manufacturing employees in urban non-private units in 2024 was 15,428$; while for manufacturing employees in urban private units, the average annual income was 10,209$. Therefore, the annual labor cost is estimated to be $50,000 based on an average of 3-5 personnel responsible for operating the project.

Supplementary Table 10 Estimated total costs for H_2O_2 production per year when working area was scaled up 500 m^2.

Current density mA cm^{-2}	Total operating cost $/year	Capital equipment expenditure $/year		Operational cost accounting $/year	Output ton/year	Total $/kg
		Depreciation cost $/year	Maintenance cost $/year			
250	3108849	233,452	5,836	50,000	5285	0.64298

500	6966020	233,452	5,836	50,000	10129	0.71629
750	12323091	233,452	5,836	50,000	15830	0.79674
1000	19251727	233,452	5,836	50,000	19870	0.98344

Notes: After the electrosynthesis of H_2O_2 in a salt solution, product separation and electrolyte recovery present a key challenge. Possible solution strategies include post-processing methods such as concentration and salting-out or integrated separation, which couples with the electrolyzer.

(1) Concentration and salting-out methods, such as vacuum distillation, concentrate the electrolyte to crystallize out the salt or obtain a more concentrated H_2O_2 solution based on solubility differences. The costs involve: (1) Capital investment for the vacuum system, evaporator, condenser, and heat pump. (2) High operational energy costs, dominated by thermal energy, which is typically high. (3) Maintenance costs for addressing equipment corrosion and scaling. Economic Profile: The initial investment might be lower than for complex membrane systems. However, the absolute value of operational energy consumption is very high, constituting the major cost component. This method is not suitable for alkaline H_2O_2 solutions, as it leads to rapid decomposition and a consequent decrease in effective product yield. The above cost assessment for acidic H_2O_2 product separation adopts this method.

(2) Integrated separation couples the electrolyzer with other separation processes (e.g., electrodialysis) to simultaneously produce H_2O_2 and separate or concentrate the salt. The cost structure involves: (1) Capital investment for specialized equipment, including the membrane stack, electrodes, and control system. (2) Operational energy costs, primarily the electrical power required to drive ion migration. (3) Additional expenses for membrane cleaning and replacement due to fouling or damage. Economic Profile: The initial investment may be relatively high. However, operational energy consumption is comparatively lower and more controllable. The system shares similarities with the H_2O_2 electrosynthesis setup, potentially reducing the need for an entirely set of additional equipment.

There is another scenario in which the recovery and reuse of electrolytes (e.g., seawater) are not considered when doing so is economically impractical. Instead, the cost of the electrolyte is included in the total production cost. In such cases, subsequent issues related to H_2O_2 application and waste stream disposal, such as the disposal of acidic solutions and the cost of neutralizing acidic waste, must also be addressed. Accordingly, the TEA factors for H_2O_2 electrosynthesis are calculated as follows.

1. The operating cost for H_2O_2 production includes the costs for O_2, electricity, and pre-treatment and post-treatment of electrolyte.

Supplementary Table 11 Operating costs for H_2O_2 production in seawater using OCNT-CTAB as the cathode and IrTaTi as the anode with 4 cm^2 working area.

Current density mA cm^{-2}	Yield rate *10^{-3} kg/h	Electricity cost kwh/kgH_2O_2	O_2 cost \$/kgH_2O_2	Treatment cost \$/kgH_2O_2	Total operating cost \$/kgH_2O_2
50	0.11607	0.2954	0.58611	0.27762	1.15913
150	0.31304	0.4381	0.21731	0.10293	0.75835

250	0.52635	0.58353	0.12924	0.06122	0.774
350	0.74268	0.67323	0.0916	0.04339	0.80822
450	0.90684	0.8365	0.07502	0.03553	0.94705
500	1.008	0.86451	0.06749	0.03197	0.96397

Supplementary Table 12 Estimated operating costs for H_2O_2 production in seawater per year when working area was scaled up 500 m^2.

Current density mA cm^{-2}	Yield rate kg/h	Output ton/year	Total operating cost \$/year
50	145	1160	1345402
150	391	3130	2373938
250	657	5263	4073949
350	928	7426	6002488
450	1133	9068	8588228
500	1260	10080	9716817

Note: the calculation of electricity cost, O_2 cost and H_2O cost is shown above.

2. Capital equipment expenditure

$$\text{Electrolyzer cost} = 500\ \text{m}^2 \times \$1619\ \text{m}^{-2} \times 1.2 = \$971{,}400 \quad (8)$$

$$\text{BOP cost} = \text{Electrolyzer} \div 65\% \times 35\% = \$523{,}061 \quad (9)$$

$$\text{Depreciation cost} = \frac{(\text{Electrolyzer cost} + \text{BOP cost}) \times 3.25\%}{1 - \frac{1}{(1+3.25\%)^{20}}} = \$102{,}787/\text{year} \quad (15)$$

The maintenance cost is assumed 2.5% of capital cost per year (from H2A):

$$\text{Maintenance cost} = \text{Depreciation cost} \times 2.5\% = \$2{,}570/\text{year} \quad (16)$$

$$\text{Capital equipment expenditure} = \text{Depreciation cost} + \text{Maintenance cost} = \$105{,}357/\text{year} \quad (17)$$

3. Operational cost accounting

Consistent with the approach above, the cost here is calculated in the same manner. Therefore, the annual labor cost is estimated at \$50,000 based on an average of 3 to 5 personnel required for project operation.

Supplementary Table 13 Estimated total costs for H_2O_2 production in seawater per year when working area was scaled up 500 m^2.

Current density mA cm^{-2}	Total operating cost $/year	Capital equipment expenditure $/year		Operational cost accounting $/year	Output ton/year	Total $/kg
		Depreciation cost $/year	Maintenance cost $/year			
50	1345402	102,787	2,570	50,000	1160	1.29376
150	2373938	102,787	2,570	50,000	3130	0.80808
250	4073949	102,787	2,570	50,000	5263	0.80359
350	6002488	102,787	2,570	50,000	7426	0.82923
450	8588228	102,787	2,570	50,000	9068	0.96422
500	9716817	102,787	2,570	50,000	10080	0.97938

Commercially available H_2O_2 price is calculated as:

$$C = P_1 + P_2 \quad (18)$$

P_1: the price of 27.5 wt% H_2O_2, 257.1 $/t in China

P_2: Transportation cost of H_2O_2: 42.9–71.4 $/t in China (for a 500 km distance)

All formulas and parameters are standardized for clarity and cost calculation consistency.

Discussion:

A techno-economic assessment (TEA) was conducted comparing two scenarios: K_2SO_4-based and seawater-based processes. For the K_2SO_4-based process, additional cost considerations, beyond the operating costs discussed above, include H_2O_2 separation and electrolyte recycling. For the seawater-based process, electrolyte recycling was omitted, but costs for acid pretreatment and waste neutralization were included. The lowest achievable production costs are estimated at $0.64/kg$H_2O_2$ and $0.80/kg$H_2O_2$, respectively.

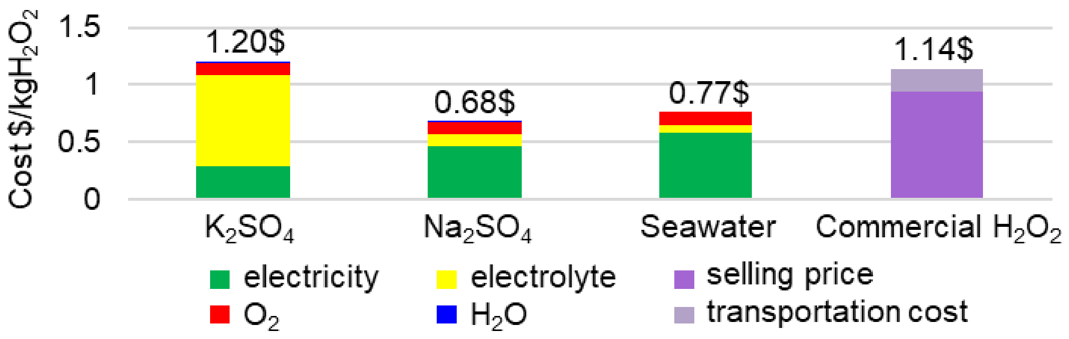

Supplementary Fig. 71 Optimized operating costs of acidic H_2O_2 electrosynthesis using acidified 0.3 M K_2SO_4, Na_2SO_4 or natural seawater as the catholyte.

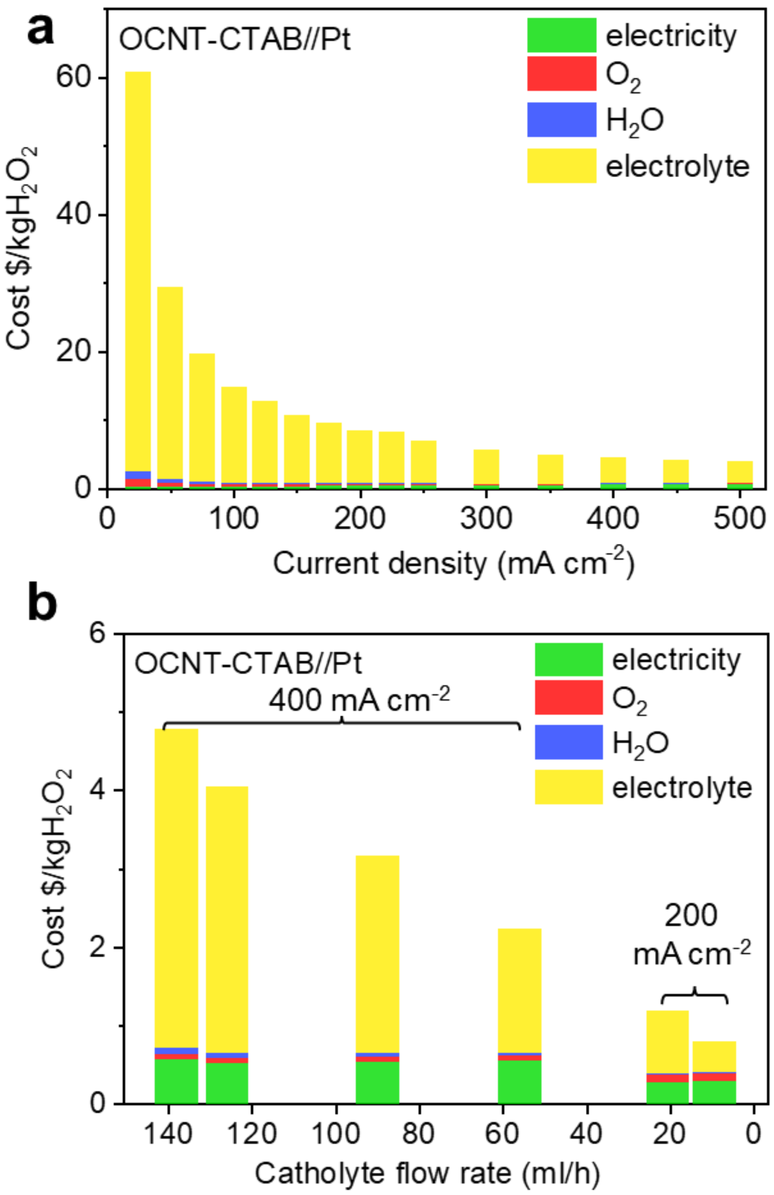

Supplementary Fig. 72 Cost investigation of acidic H_2O_2 electrosynthesis using OCNT-CTAB cathode and Pt anode in 0.1 M H_2SO_4 with 0.3 M K_2SO_4 catholyte and 0.5 M H_2SO_4 anolyte. **a**, Catholyte flow rate at about 140 mL/h. **b**, Catholyte flow rate at 10-140 mL/h.

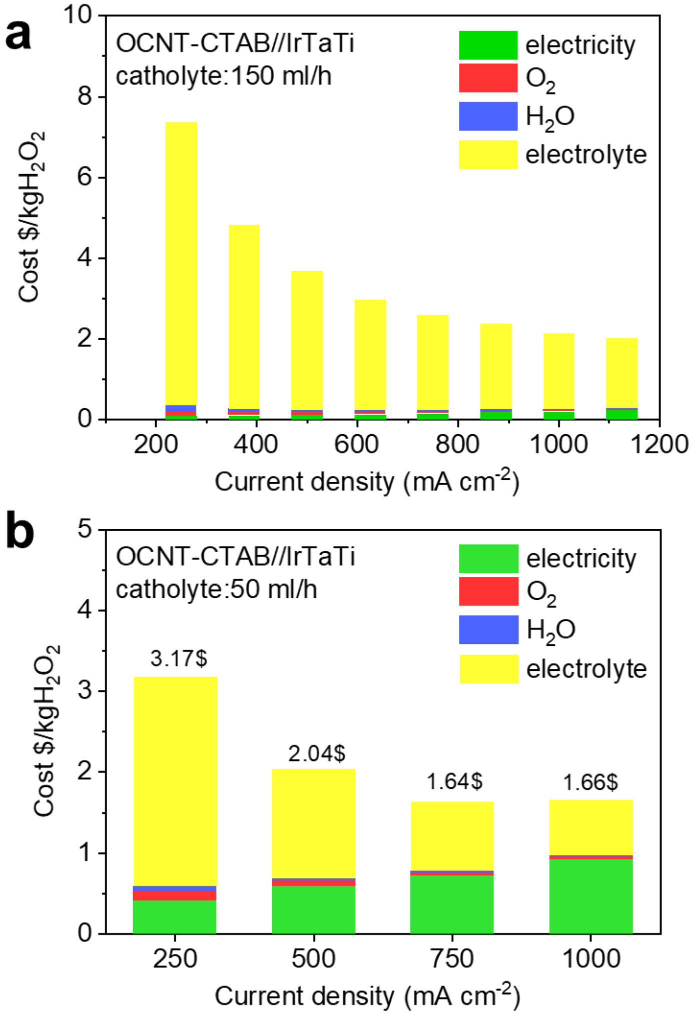

Supplementary Fig. 73 Cost investigation of acidic H_2O_2 electrosynthesis using OCNT-CTAB cathode and IrTaTi anode in 0.1 M H_2SO_4 with 0.3 M K_2SO_4 catholyte and 0.5 M H_2SO_4 anolyte. a, Catholyte flow rate at about 150 mL/h. **b**, Catholyte flow rate at 50 mL/h.

In the operating cost assessment using a Pt anode above, electrolyte consumption constituted a major portion of the overall expense, primarily due to the use of expensive K_2SO_4 electrolyte in the catholyte. It was observed that lower flow rates led to higher electrolyte consumption per unit of H_2O_2 produced. To evaluate this effect, the performance of H_2O_2 electrosynthesis using a commercial IrTaTi anode was assessed under two different flow rates of 150 mL/h and 50 mL/h. At the flow rate of 150 mL/h, the contribution of electrolyte cost to the total operating cost decreased gradually with increasing current density, eventually reaching a level comparable to that of electricity consumption. Under the 50 mL/h flow rate, the electrolyte cost was further significantly reduced, ultimately falling below the electricity cost.

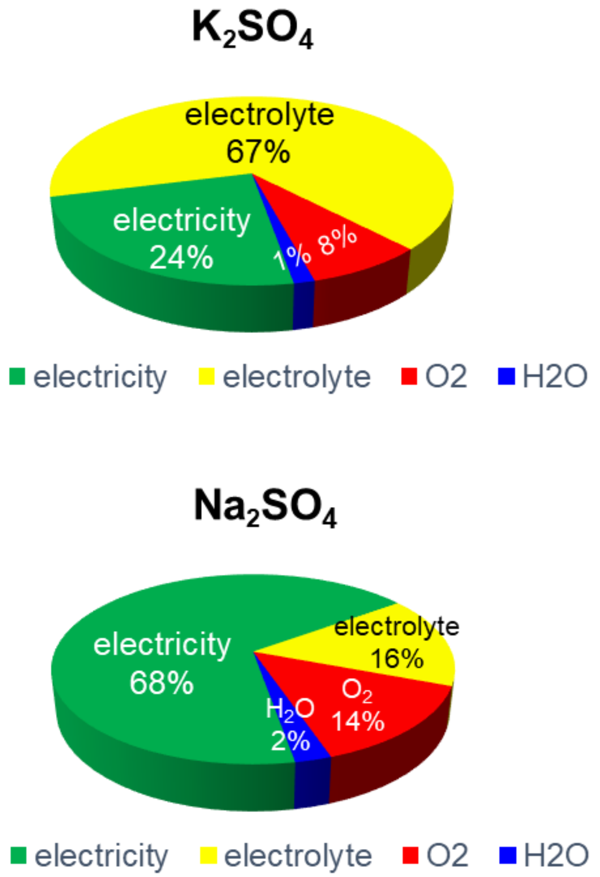

Supplementary Fig. 74 Cost investigation of acidic H_2O_2 electrosynthesis. (K_2SO_4: catholyte of 0.3 M K_2SO_4 in 0.1 M H_2SO_4, catholyte flow rate of 20.7 mL/h, 0.5 M H_2SO_4 anolyte, 200 mA cm^{-2}). Na_2SO_4: catholyte of 0.3 M Na_2SO_4 in 0.1 M H_2SO_4, catholyte flow rate of 20.7 mL/h, 0.5 M H_2SO_4 anolyte, 300 mA cm^{-2}).

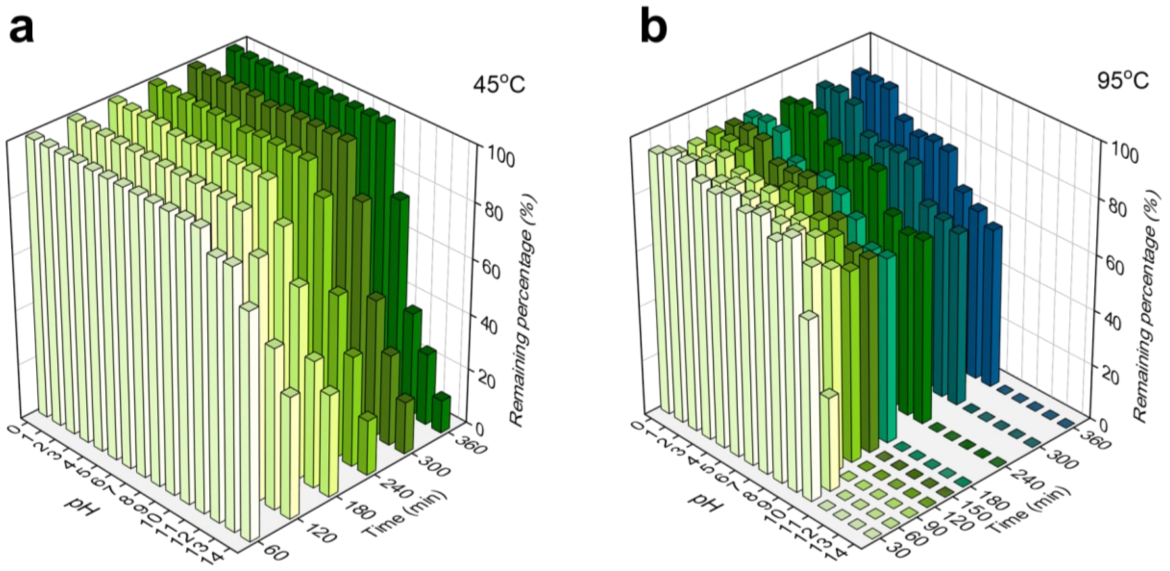

Supplementary Fig. 75 The retention of 1 wt% H_2O_2 as a function of solution pH. a, 45°C. **b,** 95°C. (Source data for Supplementary Fig. 75 are provided as a Source Data file.)

Supplementary References

1. Yu, Z. Y. *et al*. Selective and durable H_2O_2 electrosynthesis catalyst in acid by selenization induced straining and phasing. *Nat. Commun.* **15**, 9346 (2024).
2. Zhang, X. L. *et al*. Strongly coupled cobalt diselenide monolayers for selective electrocatalytic oxygen reduction to H_2O_2 under acidic conditions. *Angew. Chem. Int. Ed.* **60**, 26922-26931 (2021).
3. Cao, P. K. *et al*. Highly efficient acidic electrosynthesis of hydrogen peroxide at industrial-level current densities promoted by alkali metal cations. *Angew. Chem. Int. Ed.* **136**, e202406452 (2024).
4. Lin, Z. *et al*. Atomic Co decorated free-standing graphene electrode assembly for efficient hydrogen peroxide production in acid. *Energy Environ. Sci.* **15**, 1172-1182 (2022).
5. Huang, H. *et al*. Enhancing H_2O_2 electrosynthesis at industrial-relevant current in acidic media on diatomic cobalt sites. *J. Am. Chem. Soc.* **146**, 9434-9443 (2024).
6. Chen, Y. H. *et al*. Oxygen functional groups regulate cobalt-porphyrin molecular electrocatalyst for acidic H_2O_2 electrosynthesis at industrial-level current. *Angew. Chem. Int. Ed.* **63**, e202407163 (2024).
7. Chen, S. Y. *et al*. Tuning proton affinity on Co−N−C atomic interface to disentangle activity-selectivity trade-off in acidic oxygen reduction to H_2O_2. *Angew. Chem. Int. Ed.* **137**, e202418713 (2024).
8. Shim, J. *et al*. Efficient H_2O_2 electrosynthesis in acidic media via multiscale catalyst optimization. *Adv. Mater.* **37**, 2418489 (2025).
9. Adler, Z. *et al*. Hydrogen peroxide electrosynthesis in a strong acidic environment using cationic surfactants. *Precis. Chem.* **2**, 129-137 (2024).
10. Zhang, X. *et al*. Electrochemical oxygen reduction to hydrogen peroxide at practical rates in strong acidic media. *Nat. Commun*. **13**, 2880 (2022).
11. Zhang, C. *et al*. A pentagonal defect-rich metal-free carbon electrocatalyst for boosting acidic O_2 reduction to H_2O_2 production. *J. Am. Chem. Soc.* **145**, 11589−11598 (2023).
12. Fan, L. *et al*. High entropy alloy electrocatalytic electrode toward alkaline glycerol valorization coupling with acidic hydrogen production. *J. Am. Chem. Soc.* **144**, 7224-7235 (2022).
13. Zhao, Q. *et al*. Approaching a high-rate and sustainable production of hydrogen peroxide: oxygen reduction on Co–N–C single-atom electrocatalysts in simulated seawater. *Energy Environ. Sci.* **14**, 5444-5456 (2021).
14. Zhang, C. *et al*. Stable and high-yield hydrogen peroxide electrosynthesis from seawater. *Nat. Sustain.* **8**, 542-552 (2025).
15. Wang, X. *et al*. A chlorine-resistant self-doped nanocarbon catalyst for boosting hydrogen peroxide synthesis in seawater. *Angew. Chem. Int. Ed.* **64**, e202419049 (2025).
16. Nie, J. *et al*. Accelerating water dissociation to achieve ampere-level hydrogen peroxide electrosynthesis in brine and seawater. *Nat. Commun.* **16**, 5895 (2025).
