# Supplementary material for: Cation-tuned acidic electrified interface for hydrogen peroxide electrosynthesis with industrial-level current densities in natural seawater
Source: Nat Commun. 2026 Apr 20;17:5443. doi: 10.1038/s41467-026-72026-2 (PMC13279823; doi:10.1038/s41467-026-72026-2)
Supplement: Supplementary file 2 — Description of Additional Supplementary Files [file 41467_2026_72026_MOESM2_ESM.pdf]

Description of Additional Supplementary Files

File Name: Supplementary Data 1

Description: This folder contains raw data for Figure 3 in the main text and for Supplementary Figs. 32–47, 61, 62 in the Supplementary Information. It includes optimized structures for *OOH adsorption and Cl^- adsorption on carbon sites, as well as the initial and final models from AIMD simulations..
