# Supplementary material for: Cation-tuned acidic electrified interface for hydrogen peroxide electrosynthesis with industrial-level current densities in natural seawater
Source: Nat Commun. 2026 Apr 20;17:5443. doi: 10.1038/s41467-026-72026-2 (PMC13279823; doi:10.1038/s41467-026-72026-2)
Supplement: Supplementary file 4 — Transparent Peer Review file [file 41467_2026_72026_MOESM4_ESM.pdf]

nature portfolio

Peer Review File

Cation-tuned acidic electrified interface for hydrogen peroxide electrosynthesis with industrial-level current densities in natural seawater

Corresponding Author: Professor Xie Quan

This file contains all reviewer reports in order by version, followed by all author rebuttals in order by version.

Version 0:

Reviewer comments:

Reviewer #1

(Remarks to the Author)
This manuscript reports a combined experimental and computational investigation into electrocatalytic oxygen reduction for producing H2O2 at industrial-scale current densities under high-salinity conditions and in natural seawater. The content of the manuscript is well matched with the scope of Nature Communications, particularly in two key aspects: (1) The key experimental finding is that CTAB cations significantly enhance the catalytic performance of oxidized carbon, enabling acidic H2O2 electrosynthesis at industrial-level current densities (on the order of A cm-2) in both high-salinity electrolytes and natural seawater. (2) DFT calculations reveal that cationic modifiers counteract the over-binding of *OOH intermediates caused by electrified acidic interfaces and intrinsic site adsorption characteristics, while also promoting proton-independent *OOH desorption. This mechanistic interpretation is both novel and convincing. The use of explicit water models and AIMD simulations to represent realistic interfacial environments represents a particular strength, compared with the simplified models commonly employed in the literature.

I recommend acceptance of this manuscript by Nature Communications after the authors address the following minor comments.
1. In Figs. S28 and S29, the author simulated proton-depleted interfacial conditions. How was the "proton-free" interface explicitly defined in these AIMD simulations, through complete removal of all H3O+ ions or partial reduction of proton concentration?
2. In Lines 267–269, the description of oxygen doping effects feels inconsistent. While the strengthened *OOH binding at C8/C9 is clearly benchmarked against C7, the weakened binding at C5/C6 lacks a parallel reference (e.g., weaker than C4). Revising this to maintain a consistent comparative structure would improve clarity.
3. The presence of cations such as Mg2+ and Ca2+ in raw Yellow Sea seawater is expected to influence cathode scaling behavior. Measuring and reporting their concentrations is essential.
4. Although a commercial IrTaTi oxide anode was used for OER, its physicochemical properties (e.g., composition) and electrochemical performance relative to the Pt electrode should be characterized. Such data would help establish its contribution to lower the overall cell voltage.

Reviewer #2

(Remarks to the Author)
The authors report a strategy to enhance the electrochemical two-electron oxygen reduction reaction (2e⁻ ORR) for hydrogen peroxide (H_2O_2) production in natural seawater under acidic conditions. The core innovation is a “cationic modification strategy,” where oxidized carbon nanotubes (OCNTs) are modified with surfactants (e.g., CTAB) or ionomers. The authors attribute the performance enhancement to the modulation of the electrified interface by this cationic layer, which they claim prevents the over-stabilization of the *OOH intermediate at high negative potentials. The claims are supported by electrochemical data, spectroscopic characterization (ATR-SEIRAS, NMR), and Ab Initio Molecular Dynamics (AIMD) simulations. A techno-economic analysis (TEA) is also included to support the economic viability of the proposed method. The work addresses a significant challenge in electrosynthesis: achieving high current densities for H_2O_2 production in a complex, realistic electrolyte (seawater) while mitigating scaling and selectivity loss. However, I have several reservations

concerning the claimed industrial practicality, the long-term stability of the proposed interface, and the rigor of the mechanistic explanation. Consequently, I cannot recommend publication in its current form. Substantial revisions are required to address major concerns regarding the system's stability, the proposed mechanism, and its economic viability. My specific points are detailed below:

1. The proposed strategy of acidifying seawater to pH ~1 to prevent scaling is problematic. Given seawater's high buffering capacity, this approach would necessitate substantial acid input. The assertion that the acid cost is negligible (<2% of OpEx) is highly optimistic and lacks sufficient justification. The authors must provide a rigorous calculation of the acid volume required per unit of seawater, factoring in its natural alkalinity. Furthermore, the environmental unfeasibility of discharging highly acidic (pH 1) effluent is a critical flaw. The omission of wastewater neutralization costs from the TEA renders the analysis incomplete and misleading regarding the process's true economic viability.

2. The manuscript attributes the enhanced performance to the cationic headgroup's modulation of *OOH binding, distinct from simple hydrophobic effects. However, the use of CTAB, which possesses a long hydrophobic alkyl chain, convolutes the proposed electronic effect with mass transport limitations, particularly at high current densities. To deconvolve these effects and substantiate the claim regarding the electronic role of the cation, the authors should conduct control experiments with a short-chain cationic surfactant (e.g., tetramethylammonium bromide). If the hypothesis is correct, this molecule should still confer a performance benefit despite its significantly lower hydrophobicity. Additionally, electrochemical double-layer capacitance (Cdl) measurements should be performed to quantify any changes in the electrochemically active surface area (ECSA) and to probe the interfacial structure.

3. The stability test reveals a critical flaw. The authors attribute a performance drop to "electrode flooding," which required manual drying to remedy. The need for intermittent manual intervention represents an unacceptable failure mode for any practical or industrial process and severely undermines the reported stability claims. The authors must provide a transparent investigation into this failure mechanism. Is the performance loss due to degradation of the hydrophobic layer or leaching of the surfactant? Quantifying surfactant leaching via Total Organic Carbon (TOC) analysis of the effluent during the 200-hour stability test is essential to validate the long-term integrity of the electrode interface.

4. Natural seawater's high chloride concentration (~0.5 M) presents a significant, unaddressed challenge. At the operating conditions (pH ~1, high potentials), the Chlorine Evolution Reaction (CER) is a thermodynamically favorable parasitic reaction. Moreover, chloride ions are known to poison certain catalytic sites. The manuscript focuses on the competition between $2e^-$ and $4e^-$ ORR but largely overlooks the potential interference from chloride. The authors must demonstrate high selectivity against CER by testing for active chlorine species (e.g., Cl_2, HOCl) in the product stream. Furthermore, cyclic voltammetry studies in Cl^--containing and Cl^--free (e.g., with SO_4^{2-} as the non-interfering anion) electrolytes are needed to investigate whether Cl^- competitively adsorbs on active sites or alters the ORR onset potential.

5. The AIMD analysis (Figure 3), which suggests that cations prevent O-O cleavage at the electrified interface, requires clarification. The simulation setup is not sufficiently detailed. How was the surface coverage of cations determined, and was this density correlated with experimental loadings on the OCNTs? While the proposed "proton depletion" mechanism is intriguing, the link between the presented AIMD snapshots and the macroscopic electrochemical performance remains qualitative rather than quantitative.

6. The experiments utilize a membrane-separated cell (H-cell or flow cell), presumably with a cation exchange membrane (CEM/PEM) given the acidic anolyte (H_2SO_4). In such a system, cations (Na^+, Mg^{2+}, Ca^{2+}) from the seawater catholyte are expected to migrate across the membrane towards the anode. The authors should discuss the implications of this ion crossover on long-term anode stability, membrane integrity, and the purity of the produced H_2O_2.

7. In Tables S1/S2, the authors compare their performance metrics with literature values. For a fair and meaningful comparison, the authors must explicitly state the pH conditions for each cited work. Many studies on H_2O_2 production in "seawater" are conducted at neutral pH. Comparing performance across different pH regimes without providing proper context regarding overpotentials and thermodynamic potentials can be misleading.

8. The manuscript contains minor grammatical errors and colloquialisms. For instance, in the abstract/introduction, "ampere-level current densities" is informal; standard scientific phrasing such as "current densities on the order of A cm^{-2}" or simply stating the values is preferred. The text would benefit from careful proofreading.

9. The manuscript appears to self-cite a very recent publication by the same group (Ref. 4, Angew. Chem. Int. Ed.), which also investigates the role of cations in acidic electrosynthesis. The authors must explicitly and clearly delineate the novelty of the present work in relation to their previous publication. The distinction between the two studies must be sharpened to justify a separate publication.

Reviewer #3

(Remarks to the Author)
This study proposes an innovative strategy for dynamically modulating acidic electrified interfaces through cation modification of carbon-based materials, aiming to achieve efficient and highly selective electrosynthesis of hydrogen peroxide in natural seawater or high-salinity wastewater. The work demonstrates exceptional performance metrics (e.g., >90% Faradaic efficiency at 500 mA cm^{-2}). However, despite its promising prospects, the current manuscript contains a series of substantive deficiencies in the validation of core mechanisms, characterization of key physical properties, and

standardization of data presentation. These issues render the evidence chain supporting the central conclusions incomplete and insufficiently robust. Therefore, I recommend that this manuscript should not be accepted for publication until it undergoes Major Revision.

1. The authors attribute the efficiency loss at high currents to *OOH decomposition ($4e^-$ ORR) but completely fail to exclude the competitive contribution of the hydrogen evolution reaction (HER). This lack of empirical support undermines the explanation for the root cause of the FE loss.
2. Cationic modifiers (especially long-chain alkyl organics or polymers) are inherently insulators. The insulating organic modification layer may significantly alter electrode conductivity and mass transport structure, yet the authors have not performed any characterization of these effects.
3. Long-term electrolysis using acidified seawater necessitates the investigation of chlorine-related byproduct formation (e.g., hypochlorite) and electrode corrosion. The absence of such data renders the conclusions regarding stability and practical applicability unreliable.
4. K2SO4 is used as the supporting electrolyte in the study. Substantial literature indicates that alkali metal cations in the electrolyte (e.g., K+) can significantly influence the ORR pathway and suppress HER. The known effects of free K+ in the experimental system cannot be distinguished from the contribution of the covalently grafted cations. How can the unique mechanism of the proposed strategy be demonstrated?
5. The AIMD simulations merely illustrate phenomena; they lack in-depth analysis and quantitative evidence regarding the physicochemical nature of how cations promote desorption (e.g., electrostatic effects, solvation effects).
6. The manuscript inconsistently mixes the use of current and current density (e.g., Fig. 4a). It must be uniformly revised to use current density (mA cm⁻2) to ensure scientific rigor and readability.
7. For seawater electrolysis, the current cost calculation ($0.49–0.78/kg) is overly idealized. The economic analysis should be significantly enhanced in the discussion section. At a minimum, a qualitative discussion on the potential impact of energy costs for electrolyte recycling/recovery, and the lifespan/replacement costs of key components (e.g., proton exchange membrane, electrodes) on the total cost is required to provide a more realistic and informative cost range.

Version 1:

Reviewer comments:

Reviewer #1

(Remarks to the Author)
I think the authors have fully addressed my comments. The current manuscript is now suitable for publication in Nature Communications.

Reviewer #2

(Remarks to the Author)
I recommend publication of the manuscript.

Reviewer #3

(Remarks to the Author)
The manuscript has been well revised following the comments. It is recommended to be accepted.

Open Access This Peer Review File is licensed under a Creative Commons Attribution 4.0 International License, which permits use, sharing, adaptation, distribution and reproduction in any medium or format, as long as you give appropriate credit to the original author(s) and the source, provide a link to the Creative Commons license, and indicate if changes were made.
In cases where reviewers are anonymous, credit should be given to 'Anonymous Referee' and the source.
The images or other third party material in this Peer Review File are included in the article's Creative Commons license, unless indicated otherwise in a credit line to the material. If material is not included in the article's Creative Commons license and your intended use is not permitted by statutory regulation or exceeds the permitted use, you will need to obtain permission directly from the copyright holder.
To view a copy of this license, visit https://creativecommons.org/licenses/by/4.0/

Point-by-point response to the reviewers' comments

We would like to thank all the reviewers for the valuable comments. As described below, we have addressed all the comments in the revised manuscript.

Reviewer #1: This manuscript reports a combined experimental and computational investigation into electrocatalytic oxygen reduction for producing H_2O_2 at industrial-scale current densities under high-salinity conditions and in natural seawater. The content of the manuscript is well matched with the scope of *Nature Communications*, particularly in two key aspects: (1) The key experimental finding is that CTAB cations significantly enhance the catalytic performance of oxidized carbon, enabling acidic H2O2 electrosynthesis at industrial-level current densities (on the order of A cm^{-2}) in both high-salinity electrolytes and natural seawater. (2) DFT calculations reveal that cationic modifiers counteract the over-binding of *OOH intermediates caused by electrified acidic interfaces and intrinsic site adsorption characteristics, while also promoting proton-independent *OOH desorption. This mechanistic interpretation is both novel and convincing. The use of explicit water models and AIMD simulations to represent realistic interfacial environments represents a particular strength, compared with the simplified models commonly employed in the literature.

Response: We sincerely thank Reviewer 1 for their supportive comments and for highlighting the key strengths of our study, particularly the achievement of industrial-level current densities in complex electrolytes and the mechanistic insights gained from AIMD simulations with explicit solvation.

Question 1. In Supplementary Figs. 28 and 29**,** the author simulated proton-depleted interfacial conditions. How was the "proton-free" interface explicitly defined in these AIMD simulations, through complete removal of all H_3O^+ ions or partial reduction of proton concentration?

Response: Thank you for this important question. In our AIMD simulations, the "proton-free" or more accurately, the proton-depleted interface was explicitly defined neither by the complete removal of all H_3O^+ ions nor by a reduction of bulk proton concentration. This modeling choice is to estimate the effect of cationic modification in the H^+ concentration at the catalytic interface and its hydrogenation behavior toward reactants. Previous studies have indicated that under high-current-density cathodic electrolysis in acidic media, rapid consumption of H^+ leads to the formation of a proton-deficient region near the electrode interface, which has an important impact on H_2O_2 efficiency.

To realistically capture this specific microenvironment, we constructed a model where one proton (in the form of H_3O^+) was placed away from the catalyst-water interface. This setup explicitly creates a local interface that is transiently depleted of available protons for the reaction, which is a more physically accurate representation of the high-current-density operational condition.

Action: As suggested by the reviewer, we have clarified this approach by adding a detailed description to the Methods section (Lines 622-625) in the revised manuscript.

"To more realistically model the 2e⁻ ORR to H_2O_2 under high-current-density operation in acidic electrolyte, we constructed a locally proton-deficient catalyst–water interface, while positioning the solvated protons (H_3O^+) away from the interfacial region."

Question 2. In Lines 267-269, the description of oxygen doping effects feels inconsistent. While the strengthened *OOH binding at C8/C9 is clearly benchmarked against C7, the weakened binding at C5/C6 lacks a parallel reference (e.g., weaker than C4). Revising this to maintain a consistent comparative structure would improve clarity.

Response: Thank you for raising this point regarding comparative clarity. To address this, we have made the following revision. The strengthened *OOH binding at C8/C9 is clearly benchmarked against C7, the weakened binding at C5/C6 is in reference to C4.

Action: Accordingly, the description in Lines 288-290 has been revised as follows for clarity:

*"In multi-vacancy structures, oxygen doping modulated *OOH binding in contrasting ways: for C7-type carbon, binding strengthened at sites C8 and C9, whereas for C4-type carbon, it weakened at C5 and C6."*

(Previously: "*In multi-vacancy structures, oxygen doping exerted divergent effects: for C7-type carbon, it enhanced *OOH binding at C8 and C9 but weakened it at C4–C6.*")

Question 3. The presence of cations such as Mg^{2+} and Ca^{2+} in raw Yellow Sea seawater is expected to influence cathode scaling behavior. Measuring and reporting their concentrations is essential.

Response: Thank you for highlighting the importance of quantifying Mg^{2+} and Ca^{2+} concentrations. We measured the concentrations of Na^+, K^+, Mg^{2+}, and Ca^{2+} in raw Yellow Sea seawater sample collected from Dalian using an ion chromatography, which were determined to be 9854, 411, 1303 and 555 mg L^{-1}, respectively. Additionally, a seawater sample was collected from the Yellow Sea off the coast of Qingdao (36° N, 120° E). Its cation concentrations were found to be similar to those of a sample collected from Dalian.

Action: In response to the Reviewer's comment, we have added a detailed description of the analytical method to the Methods section. The measured concentrations are now reported in the revised manuscript and the Supporting Information.

Line 583-590 in Methods section

*"The concentrations of Na^+, K^+, Mg^{2+}, and Ca^{2+} in the samples were determined using an ion chromatograph (Thermo Scientific DIONEX AQUION RFIC) equipped with a Dionex IonPac CS16 5×150 mm separation column and a 30 mM aqueous methanesulfonic acid solution as the mobile phase. A mixed standard solution containing 50.0 mg L^{-1} of K^+, 250 mg L^{-1} of Na^+, 250 mg L^{-1} of Ca^{2+}, and 50.0 mg L^{-1} of Mg^{2+} was prepared (**Supplementary Fig. 65**; all R^2 > 0.99). It was then diluted into a series of standard samples with varying concentrations to establish a calibration curve."*

Line 420-422 the revised manuscript

*"The concentrations of Na^+, K^+, Mg^{2+}, and Ca^{2+} in the seawater sample were determined to be 9854, 411, 1303 and 555 mg L^{-1}, respectively, as presented in **Supplementary Fig. 65 and Supplementary Tables 2-3**."*

Figures in Supporting Information

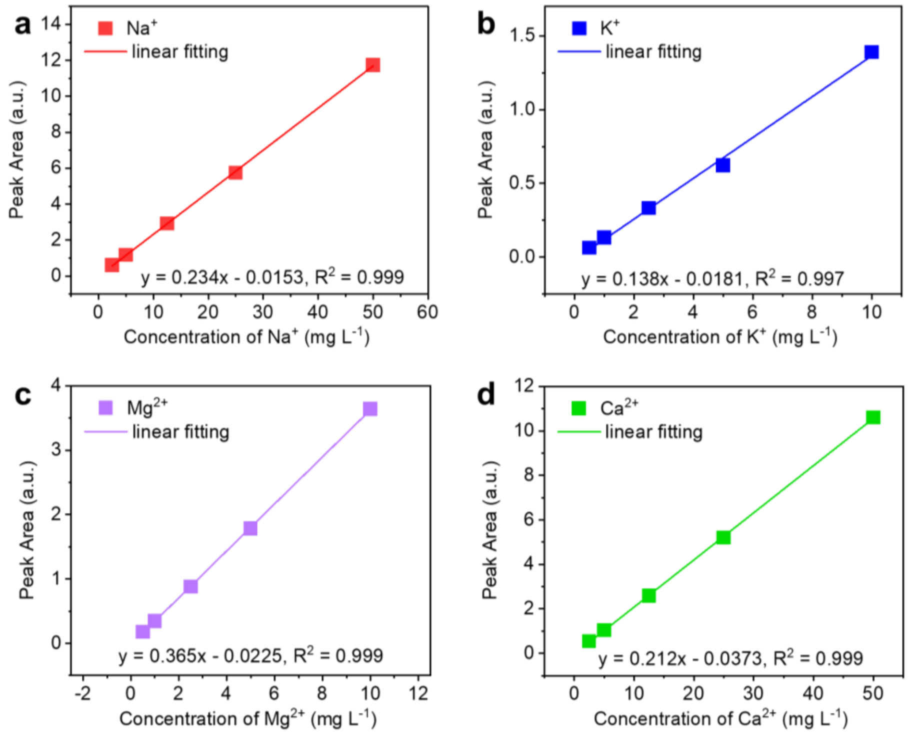

Supplementary Fig. 65 Established linear calibration curves (peak area vs. concentration) for Na^+, K^+, Mg^{2+}, and Ca^{2+}.

Supplementary Table 2 Concentrations of Na^+, K^+, Mg^{2+}, and Ca^{2+} in the raw Yellow Sea seawater sample collected from the Yellow Sea off the coast of Dalian city (39° N, 122° E).

Cations	Concentrations (mg L^{-1})	Concentrations (mM)
Na^+	9854	429
K^+	411	11
Mg^{2+}	1303	54
Ca^{2+}	555	14

Supplementary Table 3 Concentrations of Na^+, K^+, Mg^{2+}, and Ca^{2+} in the raw Yellow Sea seawater sample collected from the Yellow Sea off the coast of Qingdao city (36° N, 120° E) on 22 June 2025.

Cations	Concentrations (mg L^{-1})	Concentrations (mM)
Na^+	9735	423
K^+	396	10
Mg^{2+}	1290	53
Ca^{2+}	544	14

Question 4. Although a commercial IrTaTi oxide anode was used for OER, its

physicochemical properties (e.g., composition) and electrochemical performance relative to the Pt electrode should be characterized. Such data would help establish its contribution to lower the overall cell voltage.

Response: We examined the micro morphology and elemental composition of the IrTaTi oxide anode. SEM images revealed a nanoparticle-based coating of IrTa oxide on the Ti substrate. XPS analysis confirmed the presence of Ir, Ta, Ti, and O in the coating and identified their chemical states as IrO_2, Ta_2O_5, and TiO_2, respectively. The electrochemical performance of IrTaTi and Pt for the oxygen evolution reaction (OER) was evaluated in 0.5 M H_2SO_4 by linear sweep voltammetry and multi-step chronopotentiometry. Compared to a Pt electrode, the IrTaTi anode exhibited a lower onset potential and required 0.55–0.77 V lower overpotentials to achieve current densities of 100–350 mA cm^{-2}. This significantly higher OER activity of the IrTaTi anode contributes to a significantly lower overall cell voltage.

Action: In response to the Reviewer's comment, we have added new figures and discussion in the revised Supplementary Information, as follows:

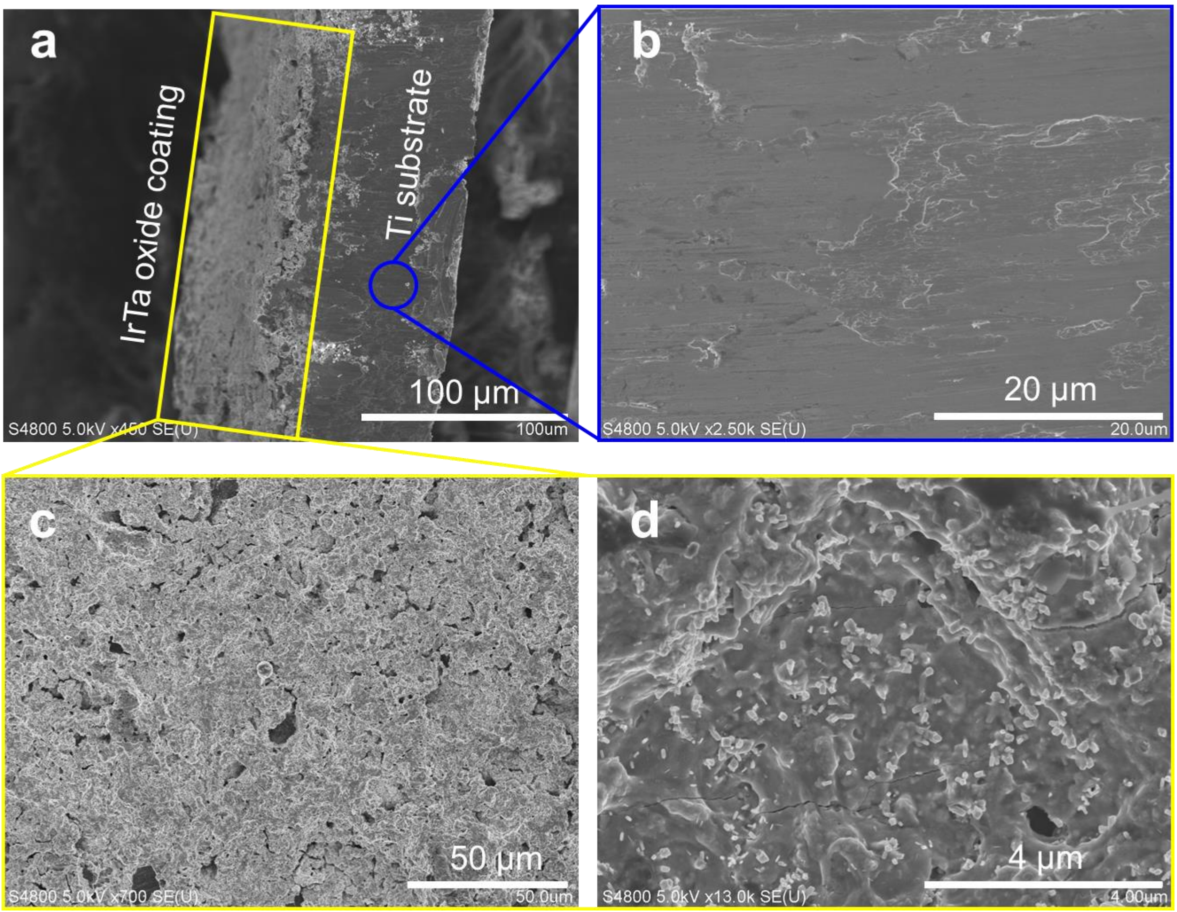

Supplementary Fig. 55 SEM images of IrTaTi electrode. a, Electrode cross-sectional view. b, High resolution image of the Ti substrate. c-d, High-resolution images of the IrTa oxide coating.

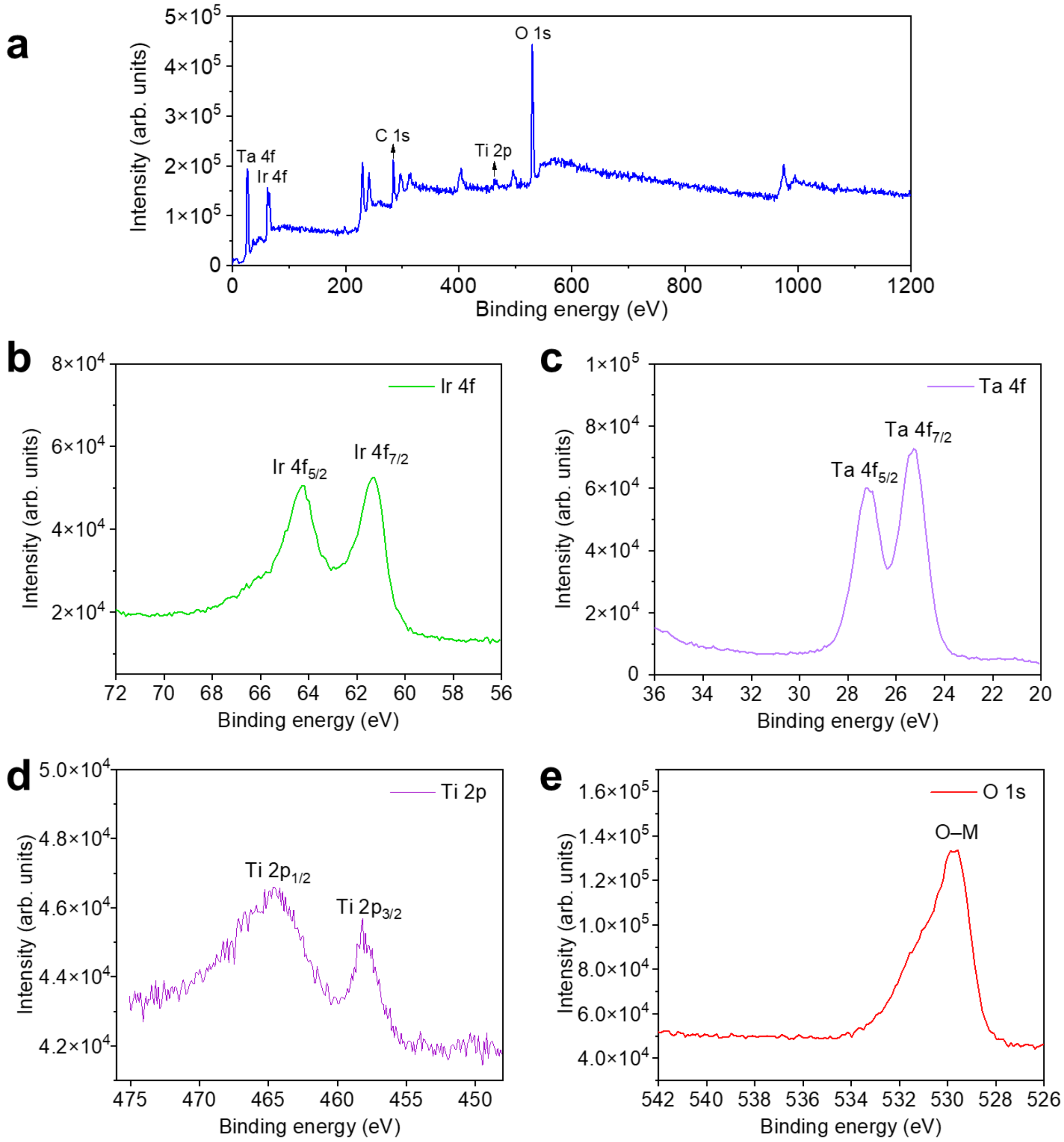

Supplementary Fig. 56 Elemental composition of the IrTaTi oxide anode measured by XPS spectra. a, XPS survey spectrum. b-d, high-resolution spectra of Ir, Ta, Ti and O.

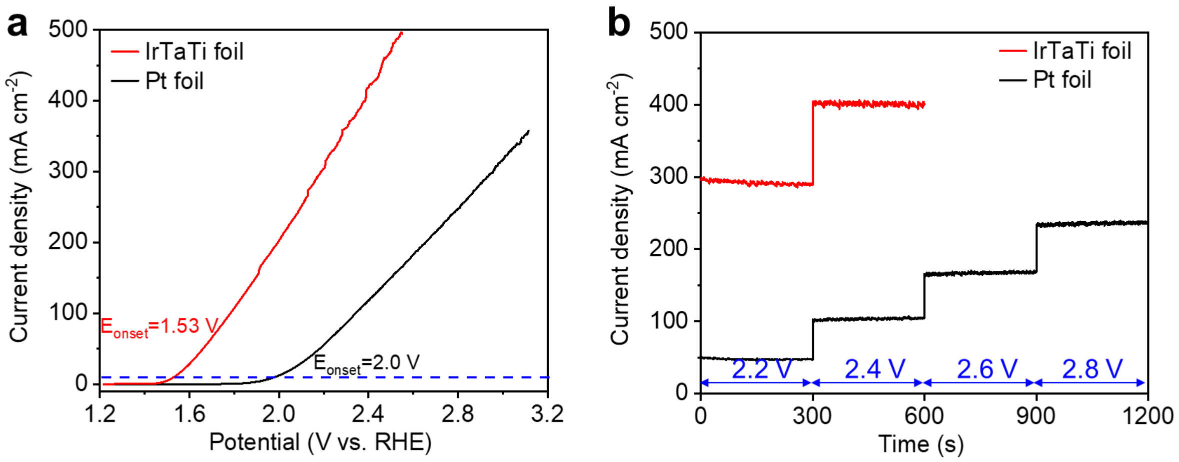

Supplementary Fig. 57 Electrochemical performance comparison of IrTaTi and Pt for the oxygen evolution reaction. a, Linear sweep voltammetry curves at a scan rate of 10 mV s^{-1} in 0.5 M H_2SO_4. b, Chronoamperometric responses obtained via multi-step chronopotentiometry

SEM images revealed a nanoparticle-based IrTa oxide coating on the Ti substrate, and XPS analysis confirmed its elemental composition, with atomic percentages of Ir (7.0%), Ta (14.4%), Ti (4.9%), and O (73.6%). In the survey spectrum, the primary peaks at 26, 62, 464, and 530 eV were assigned to Ta 4f, Ir 4f, Ti 2p, and O 1s core levels, respectively, while minor features at approximately 230 eV, 296 eV, and 313 eV were tentatively attributed to Mo 3d, Ir 4d, and a carbon-related signal. High-resolution spectral analysis further elucidated the chemical states: the Ir 4f doublet (61.4 and 64.3 eV) is characteristic of Ir^{4+} in IrO_2, the Ta 4f doublet (25.3 and 27.1 eV) corresponds to Ta^{5+} in Ta_2O_5, the Ti 2p doublet (458.2 and 464.5 eV) indicates Ti^{4+} in TiO_2, and the dominant O 1s peak at ~530 eV confirms the prevalence of metal-oxygen (O–M) bonding within the coating.

The electrochemical performance of IrTaTi and Pt for the oxygen evolution reaction (OER) was evaluated in 0.5 M H_2SO_4 by linear sweep voltammetry and multi-step chronopotentiometry. The IrTaTi anode exhibited a lower onset potential and required 0.55–0.77 V less overpotentials than that of the Pt electrode to deliver a current density of 100–350 mA cm^{-2}. This significantly higher current density at the same applied potential confirms the superior OER activity of IrTaTi, which contributes to a lower overall cell voltage.

Reviewer #2: The authors report a strategy to enhance the electrochemical two-electron oxygen reduction reaction ($2e^-$ ORR) for hydrogen peroxide (H_2O_2) production in natural seawater under acidic conditions. The core innovation is a "cationic modification strategy", where oxidized carbon nanotubes (OCNTs) are modified with surfactants (e.g., CTAB) or ionomers. The authors attribute the performance enhancement to the modulation of the electrified interface by this cationic layer, which they claim prevents the over-stabilization of the *OOH intermediate at high negative potentials. The claims are supported by electrochemical data, spectroscopic characterization (ATR-SEIRAS, NMR), and *Ab Initio* Molecular Dynamics (AIMD) simulations. A techno-economic analysis (TEA) is also included to support the economic viability of the proposed method. The work addresses a significant challenge in electrosynthesis: achieving high current densities for H_2O_2 production in a complex, realistic electrolyte (seawater) while mitigating scaling and selectivity loss. However, I have several reservations concerning the claimed industrial practicality, the long-term stability of the proposed interface, and the rigor of the mechanistic explanation. Consequently, I cannot recommend publication in its current form. Substantial revisions are required to address major concerns regarding the system's stability, the proposed mechanism, and its economic viability.

Response: Thank you for your constructive feedback. According to your suggestions, we have conducted additional experiments and simulations. New data on long-term stability, DFT calculations for the mechanistic insight, and an expanded techno-economic analysis have been incorporated into the revised manuscript. These additions directly address your key concerns regarding system stability, the proposed mechanism, and economic viability.

My specific points are detailed below:

Question 1. The proposed strategy of acidifying seawater to pH ~1 to prevent scaling is problematic. Given seawater's high buffering capacity, this approach would necessitate substantial acid input. The assertion that the acid cost is negligible (<2% of OpEx) is highly optimistic and lacks sufficient justification. The authors must provide a rigorous calculation of the acid volume required per unit of seawater, factoring in its natural alkalinity. Furthermore, the environmental unfeasibility of discharging highly acidic (pH 1) effluent is a critical flaw. The omission of wastewater neutralization costs from the TEA renders the analysis incomplete and misleading regarding the process's true economic viability.

Response: We appreciate the reviewer's important comment, which has allowed us to improve the study on H_2O_2 synthesis using seawater by addressing this issue. We measured the natural alkalinity of seawater, and the costs of acidification and neutralization of seawater were included in the total operating expenses.

(1) Seawater alkalinity, defined as the equivalent sum of all acid-neutralizing substances (primarily HCO_3^-, CO_3^{2-}, $B(OH)_4^-$, OH^-), according to the Chinese National Standard (GB/T 12763.4-2007). The method involved adding an excess of hydrochloric acid to acidify the sample to pH 1; the alkalinity was then calculated by subtracting the residual

acid from the total amount added. For a seawater sample collected from the Yellow Sea off Dalian (39° N, 122° E) with an initial pH of 8.0, this titration yielded a total alkalinity of 176 mmol/kg.

(2) We considered the cost of acidifying seawater using concentrated hydrochloric acid and concentrated sulfuric acid as acidic reagents this work, as well as the cost of neutralizing reagents using inexpensive hydrated lime (active ingredient is $Ca(OH)_2$). The amounts of 37% concentrated hydrochloric acid and 98% concentrated sulfuric acid required to adjust the pH of seawater (500 mL) to 1.6, 1.2, and 1.0 and the amount of $Ca(OH)_2$ consumed to re-neutralize seawater to a neutral pH (7.1-8.4) are shown as **Table R1** and **Table R2.**

Table R1 The amount of concentrated HCl or H_2SO_4 consumed to adjust the pH of seawater (500 mL) from an initial pH of about 8.0 to pH 1.6, 1.2 or 1.0 (these experiments were repeated 2-4 times, and the data represent the mean values).

HCl system	Consumed concentrated HCl (mL)	H_2SO_4 system	Consumed concentrated H_2SO_4 (mL)
pH 1.6	2.85	pH 1.6	0.53
pH 1.2	8.29	pH 1.2	1.25
pH 1.0	11.4	pH 1.0	5.87

Table R2 The amount of $Ca(OH)_2$ consumed to re-neutralize seawater (500 mL) to a near-neutral pH (7.1-8.4) (these experiments were repeated 2-4 times, and the data represent the mean values).

HCl system	Consumed $Ca(OH)_2$ (g)	H_2SO_4 system	Consumed $Ca(OH)_2$ (g)
pH 1.6	0.90	pH 1.6	0.69
pH 1.2	4.22	pH 1.2	1.90

(3) The cost of acidifying seawater and neutralization accounted for 7.9% of the total cost. In scenarios where K_2SO_4 and Na_2SO_4 were used as catholyte, we added the acid cost, which accounted for about 3–5% of the total cost, to the total operating expenses as shown below.

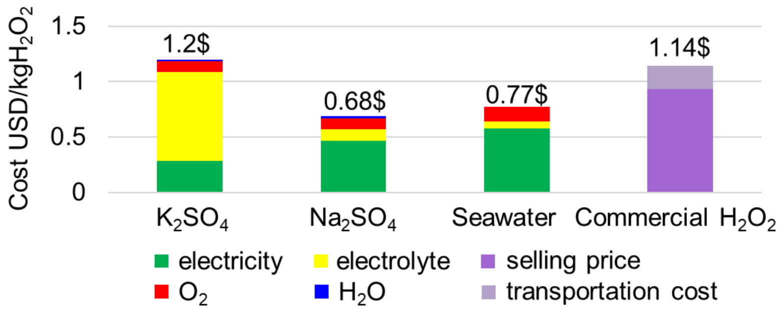

Figure R1 Operating cost comparison of acidic H_2O_2 electrosynthesis using acidified 0.3 M K_2SO_4, Na_2SO_4 or acidified natural seawater as the catholyte.

(4) We have performed Techno-economic assessments (TEA) calculations under two scenarios. In the first scenario, using a K_2SO_4 electrolyte for the electrosynthesis of H_2O_2, the energy costs for electrolyte recycling/recovery are taken into account. In the second scenario, using seawater for the electrosynthesis of H_2O_2, electrolyte recycling/recovery is not considered. Moreover, the capital equipment expenditure includes the electrolyzer stack (including key components of proton exchange membrane, electrodes) and its supporting facilities such as power supplies, pumps, compressors, separation membranes/columns; operational cost accounting on the total cost is also accounted for in order to provide a more realistic and informative cost range. The detailed calculations are presented below. In summary, the costs are \$0.64–0.98/kg and \$0.80–1.29/kg, respectively.

Action: We have added the calculation methods, data, and relevant discussions regarding the cost of acidifying seawater and neutralization to the Supporting Information. Accordingly, the operating costs for the electrosynthesis of H_2O_2 in the revised main text have been updated to reflect these considerations, as follows:

Line 434-453 in the revised main text

*"Cost analysis reveals that H_2O_2 electrosynthesis in high-salinity water can be optimized to achieve a production cost of \$0.80–\$4.78/kg, with acidified K_2SO_4 electrolyte consumption accounting for 49–85% of the total expense (**Supplementary Fig. 71**). When the Pt anode was replaced with a highly active IrTaTi electrode, the production cost can be reduced to \$0.69/kgH_2O_2. Substitution with less expensive Na_2SO_4 electrolyte reduces the operating cost to \$0.68/kgH_2O_2, where electrolyte constitutes only 16% of the total cost (**Supplementary Figs. 72-73**). When using natural seawater as the electrolyte, the cost of expensive electrolyte can be saved, resulting in an estimated production cost of \$0.77/kgH_2O_2. To evaluate the economic feasibility of H_2O_2 electrosynthesis, we conducted a techno-economic assessment (TEA) comparing two scenarios: using K_2SO_4 electrolyte and using seawater. The analysis encompassed operating costs, capital expenditures, and labor; detailed calculations are provided in the Supporting Information. For the K_2SO_4-based process, the cost assessment includes H_2O_2 separation and electrolyte recycling. Here, acidic H_2O_2, which remains stable up to 95 °C is suitable for distillation, unlike alkaline H_2O_2 that decomposes rapidly (**Supplementary Fig. 74**). For the seawater-based process, while electrolyte recycling was omitted, costs for acid pretreatment and neutralization of the acidic waste stream were included. The lowest achievable H_2O_2 production costs are estimated at \$0.64/kg and \$0.80/kg for the K_2SO_4 and seawater case, respectively. Collectively, our results demonstrate both the technical feasibility and economic competitiveness of this approach for potential industrial-scale H_2O_2 production."*

Page 72-79 Supporting Information

Other cost considerations

To assess the acid cost for H_2O_2 electrolysis in seawater, the required amount of concentrated hydrochloric acid (37 wt%, analytical grade) to acidify seawater to pH 1.2 and 1.6 was determined on a per-liter basis.

$$\mathbf{C_5} = \frac{V_{add} \times \eta \times \rho}{c \times M} \times P \tag{6}$$

where:

C_5: the cost of concentrated hydrochloric acid consumed in producing 1 kg H_2O_2 (on a 100% basis), with units of \$/kg H_2O_2.

V_{add}: the amounts of concentrated hydrochloric acid (37wt%) used for acidification, where 5.7 L and 16.6 L per ton of seawater with initial pH 8.0 were consumed to reach pH 1.6 and 1.2, respectively, as calculated from **Supplementary Table 6**.

η: the conversion factor from the amount of 37 wt% HCl to the equivalent amount of 31 wt%, with an approximate value of 1.2.

ρ: mass density of the concentrated hydrochloric acid (31 wt%: 1.152 kg L^{-1}; 37 wt%: 1.179 kg/L).

c: synthesized H_2O_2 concentration (mmol L^{-1}).

M: molecular weight (H_2O_2 = 34 g mol^{-1}).

P(concentrated hydrochloric acid): The price of concentrated hydrochloric acid is closely related to its purity grade. Although industrial-grade concentrated hydrochloric acid is available at a very low cost (\$1.43/t), its significant content of Fe^{3+} interferes with H_2O_2 synthesis, making it unsuitable for use in this system. A higher-purity grade, suitable for chemical synthesis (often at 31% concentration with heavy metals content < 0.005%), is priced around \$14.3/t. It is important to note that the market price of concentrated hydrochloric acid is highly volatile and closely tied to the dynamics of the chlor-alkali industry. This assessment uses the current Chinese market price (as of December 2025) as the evaluation baseline.

We adopted a similar approach to assess the cost of using concentrated sulfuric acid as the acid agent and the cost (**C6**) of neutralization using $Ca(OH)_2$.

$$\mathbf{C_6} = \frac{m_{add}}{c \times M} \times P \tag{7}$$

where:

C_6: the cost of $Ca(OH)_2$ consumed in producing 1 kg H_2O_2 (on a 100% basis), with units of \$/kgH_2O_2.

m_{add}: the amounts of $Ca(OH)_2$ used for neutralizing the acidified seawater, where 1.8 kg and 8.4 kg per ton seawater were consumed to reach pH 7.7-7.9 and 1.2, respectively.

c: synthesized H_2O_2 concentration (mmol L^{-1}).

P($Ca(OH)_2$): The price of industrial-grade $Ca(OH)_2$ (≥90% purity) is estimated to be \$57/t based on the current Chinese market price.

Supplementary Table 6 The amount of concentrated HCl or H_2SO_4 consumed to adjust the pH of seawater (500 mL) from an initial pH of about 8.0 to pH 1.6, 1.2 or 1.0 (these experiments were repeated 2-4 times, and the data represent the mean values).

HCl system	Consumed concentrated HCl (mL)	H_2SO_4 system	Consumed concentrated H_2SO_4 (mL)
pH 1.6	2.85	pH 1.6	0.53
pH 1.2	8.29	pH 1.2	1.25
pH 1.0	11.4	pH 1.0	5.87

Supplementary Table 7 The amount of $Ca(OH)_2$ consumed to re-neutralize seawater (500 mL) to a near-neutral pH (7.1-8.4) (these experiments were repeated 2-4 times, and the data represent the mean values).

HCl system	Consumed $Ca(OH)_2$ (g)	H_2SO_4 system	Consumed $Ca(OH)_2$ (g)
pH 1.6	0.90	pH 1.6	0.69
pH 1.2	4.22	pH 1.2	1.90

Discussion:

We calculated the cost of using concentrated acid to acidify seawater to pH 1.2 and 1.6, taking hydrochloric acid or concentrated sulfuric acid as examples. The minimum cost for producing per kg of H_2O_2 is $0.031 (for acidification to pH 1.6) and $0.076 (for acidification to pH 1.2). When using calcium hydroxide to neutralize the liquid at pH 1.6 and pH 1.2, the costs are $0.03 and $0.047, respectively. The overall costs are $0.06 and $0.186, respectively. When sulfuric acid is used as the acidifying agent, the costs are $0.093 and $0.193, respectively. Between these two options, the former is less expensive, but the latter offers higher operational safety due to the volatility of hydrochloric acid, whereas concentrated sulfuric acid is more stable.

Furthermore, one point needs to be raised it that: acidic H_2O_2 is more stable than alkaline H_2O_2, particularly in environments with temperatures exceeding 50°C. Additionally, acidic H_2O_2 can be directly utilized in the Fenton process for industrial wastewater treatment, which is currently the most efficient and economical method for industrial wastewater treatment. A key characteristic of this process is that the aqueous solution must be acidified, and iron ions must be neutralized and removed after the reaction. Therefore, if intended for such a process, the costs associated with acid addition for acidic H_2O_2 and subsequent neutralization should not be considered as additional expenses.

Techno-economic assessments (TEA)

Regarding the TEA analysis of the electrolysis technology, the U.S. DOE developed and used the H2A Production Model (H2A) framework for the techno-economic analysis of hydrogen production pathways. The model includes major inputs such as capital costs, operation and maintenance, different fuel types, and financial considerations such as dollar year, plant life, and internal rate of return. Therefore, our cost analysis for the electrosynthesis of H_2O_2 references the calculation principles of the H2A model.
The technical and economic factors for H_2O_2 electrosynthesis include: operating costs, capital equipment expenditure, and labor cost.

1. The analysis is conducted with the production cost per unit weight of H_2O_2 ($/kg$H_2O_2$) as the metric. H_2O_2 electrosynthesis at the cathode, at its most basic level, requires O_2, H_2O and electricity to produce H_2O_2 along with by-product O_2 at the anode which may serve as a supply of cathode. The system boundary encompasses raw materials (water, oxygen, electrolyte replenishment), and electrical energy consumption. There are 8000 hours of operation every year, and the rest is used for equipment maintenance.

Supplementary Table 8 Operating costs for H_2O_2 production using OCNT-CTAB as the cathode and IrTaTi as the anode with 4 cm^2 working area.

Current density mA cm^{-2}	Yield rate *10^{-3} kg/h	Electricity cost kwh/kgH_2O_2	O_2 cost $/kg$H_2O_2$	H_2O cost $/kg$H_2O_2$	Total operating cost $/kg$H_2O_2$
250	0.52859	0.40539	0.1287	0.05405	0.58814
500	1.01293	0.59234	0.06716	0.02821	0.68771
750	1.58307	0.71741	0.04297	0.01805	0.77843
1000	1.98707	0.92023	0.03423	0.01438	0.96885

Supplementary Table 9 Estimated operating costs for H_2O_2 production per year when working area was scaled up 500 m^2.

Current density mA cm^{-2}	Yield rate kg/h	Output ton/year	Total operating cost $/year
250	660	5285	3108849
500	1266	10129	6966020
750	1978	15830	12323091
1000	2483	19870	19251727

Note: the calculations of electricity cost, O_2 cost and H_2O cost are shown above.

2. Capital equipment expenditure includes the electrolyzer stack and its supporting facilities such as power supplies, pumps, compressors, separation membranes/columns, etc. The electrolyzer stack cost accounts for 65% of the total capital expenditure, with the balance of plant (BOP) comprising the remaining 35%. Their installation and infrastructure costs are typically a multiple of the equipment cost, e.g., 1.2 times. It also

includes product separation and purification costs, and the capital cost of membrane separation is estimated based on distillation.

$$\text{Electrolyzer cost} = 500\ \text{m}^2 \times \$1619\ \text{m}^{-2} \times 1.2 = \$971{,}400 \quad (8)$$

$$\text{BOP cost} = \text{Electrolyzer} \div 65\% \times 35\% = \$523{,}061 \quad (9)$$

$$\text{Separation cost} = \$10664000 \times \left(\frac{\text{production rate L min}^{-1}}{1000\ \text{L min}^{-1}}\right)^{0.7} \times 0.7 \times 0.1 = \$1{,}657{,}346 \quad (10)$$

$$\text{Equipment Cost} = \text{Base Cost} \times \left(\frac{\text{Throughput}}{\text{Base Throughput}}\right)^{\text{Scaling Factor}} \times \text{Process Cost Correction Factor} \quad (11)$$

This part is estimated referring “H2A Production Model Version 3 2018 User Guide Draft”. Process Cost Correction Factor: refers to the cost adjustment coefficient of a specific process (membrane separation) relative to the benchmark process (distillation). If the scaling factor exponent is 1.0, it means that the growth rate of equipment cost is exactly proportional to the growth rate of plant capacity. The scaling factor exponent is typically 1.0 or less. If the scaling factor exponent is 0.7, then the increase in equipment cost is proportional to the increase in plant capacity raised to the 0.7 power. Scaling factor reference (distillation column): The scaling factor for the distillation method typically ranges from 0.6 to 0.7.

$$\text{Depreciation cost} = \frac{(\text{Electrolyzer cost} + \text{BOP cost} + \text{Separation cost}) \times 3.25\%}{1 - \frac{1}{(1+3.25\%)^{20}}} = \$233{,}452/\text{year} \quad (12)$$

The maintenance cost is assumed 2.5% of capital cost per year (from H2A):

$$\text{Maintenance cost} = \text{Depreciation cost} \times 2.5\% = \$5{,}836/\text{year} \quad (13)$$

$$\text{Capital equipment expenditure} = \text{Depreciation cost} + \text{Maintenance cost} = \$239{,}288/\text{year} \quad (14)$$

3. Labor cost

According to 2025 data from the National Bureau of Statistics of China, the average annual income for manufacturing employees in urban non-private units in 2024 was 15,428$; while for manufacturing employees in urban private units, the average annual income was 10,209$. Therefore, the annual labor cost is estimated to be $50,000 based on an average of 3-5 personnel responsible for operating the project.

Supplementary Table 10 Estimated total costs for H_2O_2 production per year when working area was scaled up 500 m^2.

Current density mA cm^{-2}	Total operating cost $/year	Capital equipment expenditure $/year		Operational cost accounting $/year	Output ton/year	Total $/kg
		Depreciation cost $/year	Maintenance cost $/year			
250	3108849	233,452	5,836	50,000	5285	0.64298

500	6966020	233,452	5,836	50,000	10129	0.71629
750	12323091	233,452	5,836	50,000	15830	0.79674
1000	19251727	233,452	5,836	50,000	19870	0.98344

Notes: After the electrosynthesis of H_2O_2 in a salt solution, product separation and electrolyte recovery present a key challenge. Possible solution strategies include post-processing methods such as concentration and salting-out or integrated separation, which couples with the electrolyzer.

(1) Concentration and salting-out methods, such as vacuum distillation, concentrate the electrolyte to crystallize out the salt or obtain a more concentrated H_2O_2 solution based on solubility differences. The costs involve: (1) Capital investment for the vacuum system, evaporator, condenser, and heat pump. (2) High operational energy costs, dominated by thermal energy, which is typically high. (3) Maintenance costs for addressing equipment corrosion and scaling. Economic Profile: The initial investment might be lower than for complex membrane systems. However, the absolute value of operational energy consumption is very high, constituting the major cost component. This method is not suitable for alkaline H_2O_2 solutions, as it leads to rapid decomposition and a consequent decrease in effective product yield. The above cost assessment for acidic H_2O_2 product separation adopts this method.

(2) Integrated separation couples the electrolyzer with other separation processes (e.g., electrodialysis) to simultaneously produce H_2O_2 and separate or concentrate the salt. The cost structure involves: (1) Capital investment for specialized equipment, including the membrane stack, electrodes, and control system. (2) Operational energy costs, primarily the electrical power required to drive ion migration. (3) Additional expenses for membrane cleaning and replacement due to fouling or damage. Economic Profile: The initial investment may be relatively high. However, operational energy consumption is comparatively lower and more controllable. The system shares similarities with the H_2O_2 electrosynthesis setup, potentially reducing the need for an entirely set of additional equipment.

There is another scenario in which the recovery and reuse of electrolytes (e.g., seawater) are not considered when doing so is economically impractical. Instead, the cost of the electrolyte is included in the total production cost. In such cases, subsequent issues related to H_2O_2 application and waste stream disposal, such as the disposal of acidic solutions and the cost of neutralizing acidic waste, must also be addressed. Accordingly, the TEA factors for H_2O_2 electrosynthesis are calculated as follows.

1. The operating cost for H_2O_2 production includes the costs for O_2, electricity, and pre-treatment and post-treatment of electrolyte.

Supplementary Table 11 Operating costs for H_2O_2 production in seawater using OCNT-CTAB as the cathode and IrTaTi as the anode with 4 cm^2 working area.

Current density mA cm^{-2}	Yield rate $*10^{-3}$ kg/h	Electricity cost kwh/kgH_2O_2	O_2 cost \$/kgH_2O_2	Treatment cost \$/kgH_2O_2	Total operating cost \$/kgH_2O_2
50	0.11607	0.2954	0.58611	0.27762	1.15913

150	0.31304	0.4381	0.21731	0.10293	0.75835
250	0.52635	0.58353	0.12924	0.06122	0.774
350	0.74268	0.67323	0.0916	0.04339	0.80822
450	0.90684	0.8365	0.07502	0.03553	0.94705
500	1.008	0.86451	0.06749	0.03197	0.96397

Supplementary Table 12 Estimated operating costs for H_2O_2 production in seawater per year when working area was scaled up 500 m^2.

Current density mA cm^{-2}	Yield rate kg/h	Output ton/year	Total operating cost $/year
50	145	1160	1345402
150	391	3130	2373938
250	657	5263	4073949
350	928	7426	6002488
450	1133	9068	8588228
500	1260	10080	9716817

Note: the calculation of electricity cost, O_2 cost and H_2O cost is shown above.

2. Capital equipment expenditure

$$\text{Electrolyzer cost} = 500\ m^2 \times \$1619\ m^{-2} \times 1.2 = \$971{,}400 \quad (8)$$

$$\text{BOP cost} = \text{Electrolyzer} \div 65\% \times 35\% = \$523{,}061 \quad (9)$$

$$\text{Depreciation cost} = \frac{(\text{Electrolyzer cost}+\text{BOP cost}) \times 3.25\%}{1-\frac{1}{(1+3.25\%)^{20}}} = \$102{,}787/\text{year} \quad (15)$$

The maintenance cost is assumed 2.5% of capital cost per year (from H2A):

$$\text{Maintenance cost} = \text{Depreciation cost} \times 2.5\% = \$2{,}570/\text{year} \quad (16)$$

$$\text{Capital equipment expenditure} = \text{Depreciation cost} + \text{Maintenance cost} = \$105{,}357/\text{year} \quad (17)$$

3. Operational cost accounting

Consistent with the approach above, the cost here is calculated in the same manner. Therefore, the annual labor cost is estimated at $50,000 based on an average of 3 to 5 personnel required for project operation.

Supplementary Table 13 Estimated total costs for H_2O_2 production in seawater per year when working area was scaled up 500 m^2.

Current density $mA\ cm^{-2}$	Total operating cost \$/year	Capital equipment expenditure \$/year		Operational cost accounting \$/year	Output ton/year	Total \$/kg
		Depreciation cost \$/year	Maintenance cost \$/year			
50	1345402	102,787	2,570	50,000	1160	1.29376
150	2373938	102,787	2,570	50,000	3130	0.80808
250	4073949	102,787	2,570	50,000	5263	0.80359
350	6002488	102,787	2,570	50,000	7426	0.82923
450	8588228	102,787	2,570	50,000	9068	0.96422
500	9716817	102,787	2,570	50,000	10080	0.97938

Question 2. The manuscript attributes the enhanced performance to the cationic headgroup's modulation of *OOH binding, distinct from simple hydrophobic effects. However, the use of CTAB, which possesses a long hydrophobic alkyl chain, convolutes the proposed electronic effect with mass transport limitations, particularly at high current densities. To deconvolve these effects and substantiate the claim regarding the electronic role of the cation, the authors should conduct control experiments with a short-chain cationic surfactant (e.g., tetramethylammonium bromide). If the hypothesis is correct, this molecule should still confer a performance benefit despite its significantly lower hydrophobicity. Additionally, electrochemical double-layer capacitance (Cdl) measurements should be performed to quantify any changes in the electrochemically active surface area (ECSA) and to probe the interfacial structure.

Response: This suggestion is highly important, as it helps us further differentiate the contributions of electronic effects and mass transfer effects from cationic modifiers in the synthesis of H_2O_2. Therefore, following this advice, we synthesized a short-chain cationic surfactant (tetramethylammonium bromide, MTAB) modified OCNT-MTAB catalyst, which confirmed the important role of cations in the electrosynthesis of H_2O_2. Also, we conducted electrochemical double-layer capacitance (Cdl) measurements to evaluate the electrochemically active surface area (ECSA) and electrochemical impedance spectroscopy (EIS) to probe the interfacial structure.

(1) We synthesized a MTAB modified OCNT-MTAB catalyst. XPS high-resolution spectra of N 1s confirm the loading of MTAB on the OCNT (**Figure R2**). The electrode surface exhibited similar hydrophilicity to that of OCNT (**Figure R3**). Its performance in the electrosynthesis of H_2O_2 was lower than that of OCNT-CTAB, with a particularly significant Faradaic efficiency decrease at high current densities. However, it showed an improvement compared to OCNT. This indicates that cationic modification can indeed enhance performance of H_2O_2 electrosynthesis, but strong surface hydrophobicity is a necessary parameter for high Faradaic efficiency under high current conditions. This point has also been verified on OCNT electrodes with different hydrophilic/hydrophobic

properties.

(2) We conducted electrochemical double-layer capacitance (Cdl) measurements to evaluate the electrochemically active surface area (ECSA). The results indicate that after oxidation treatment, the capacitance of the SWCNT significantly increased from 3.0 $mF \cdot cm^{-2}$ to 4.2 $mF \cdot cm^{-2}$ (**Figure R4**). Oxygen-doped carbon serves as electrochemical active sites, and the oxygen content in the oxidized carbon nanotubes (OCNT) is significantly increased after oxidation, leading to the marked enhancement in capacitance, which is consistent with the increased number of active sites. When OCNT was modified with the short-chain cationic surfactant MTAB, its capacitance remained nearly the same as that of OCNT (**Figure R5**). However, when modified with other long-chain cationic surfactants, the capacitance decreased to varying degrees, ranging from 2.4 to 4.3 $mF \cdot cm^{-2}$. It is speculated that long-chain cationic surfactants can interact with oxygen in OCNT, possibly by inhibiting the electron storage process involved in capacitance. A similar phenomenon was observed under modification with Quaternary ammonia poly(N-methyl-piperidine-co-p-terphenyl (QAPPT) and Sustainion imidazolium (Sus) polymers, and the effect was even more pronounced (**Figure R6**). Similarly, we also observed that polymer modification substantially reduced the capacitance of MWOCNT, with its capacitance of 0.8 $mF \cdot cm^{-2}$ being reduced to 0.5 $mF \cdot cm^{-2}$ (**Figure R7**). Thus, electrochemically active surface area analysis did not support a significant role of cationic modifier for electronic effects in the activity enhancement.

(3) We conducted electrochemical impedance spectroscopy (EIS) to probe the interfacial structure. As shown in **Figure R8**, the Nyquist plots from EIS measurements reveal that a smaller semicircle radius in the high-frequency region indicates faster electron transfer kinetics, while a steeper slope in the low-frequency region represents faster mass transport. The plots show that oxidation treatment and cationic polymer modification hardly alter these rates, possibly because the modification involves molecular-level interactions with OCNT rather than physical mixing, which does not adversely affect conductivity. Interestingly, after modification with cationic polymers, the mass transport capability was significantly enhanced, but the difference in mass transport capacity among different chain lengths was relatively small. These results indicated that the factors of electron-transfer kinetics and mass-transport were also not the key factor for enhanced H_2O_2 electrosynthesis.

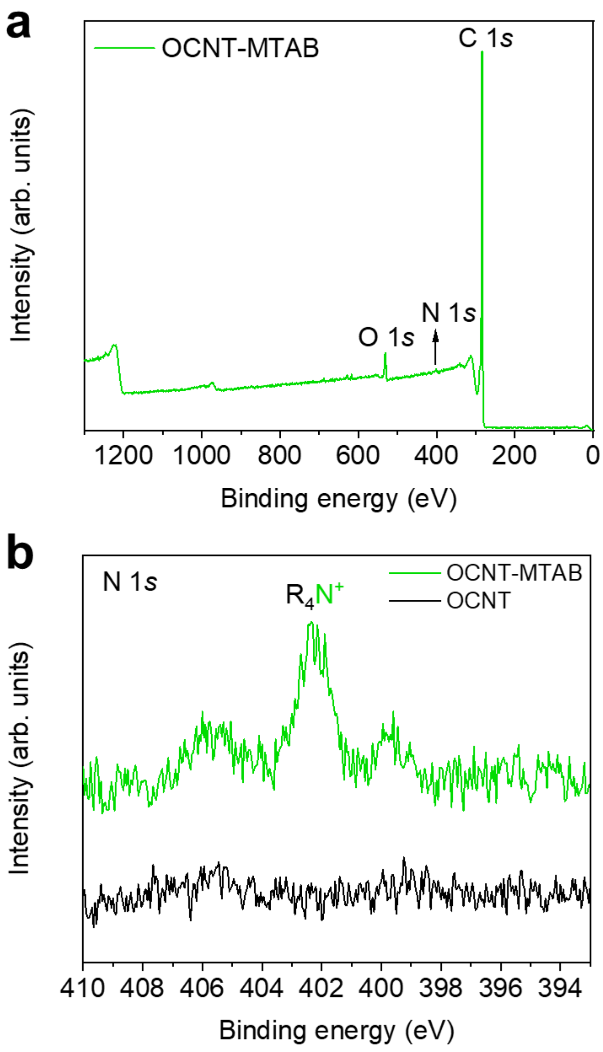

Figure R2 XPS survey spectra and high-resolution N 1*s* spectrum of OCNT-MTAB catalyst.

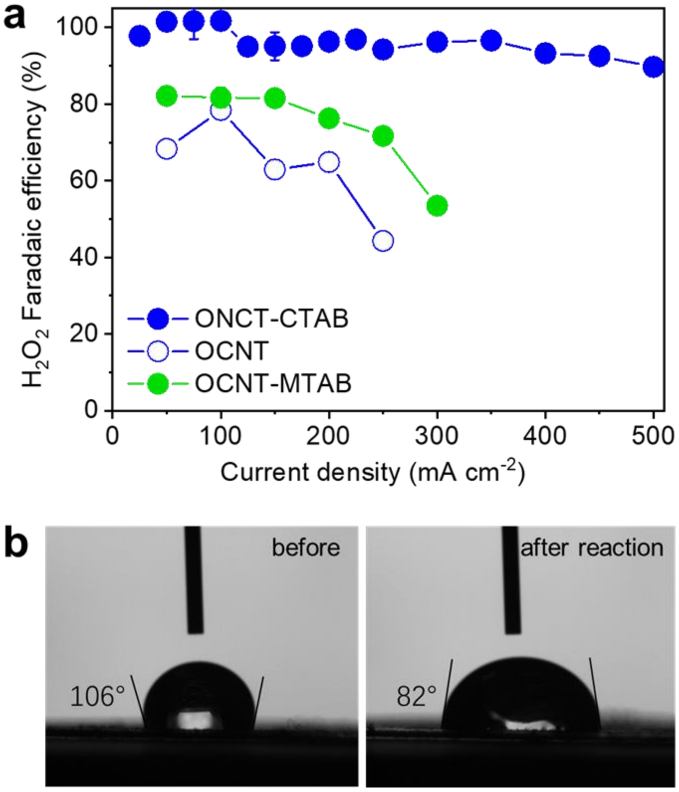

Figure R3 Performance evaluation of acidic H_2O_2 electrosynthesis using OCNT-MTAB electrode in 0.1 M H_2SO_4 with 0.3 M K_2SO_4 catholyte and 0.5 M H_2SO_4 anolyte. a, Faradaic efficiency for H_2O_2 production; b, water contact angles of the OCNT-MTAB electrode before and after reaction.

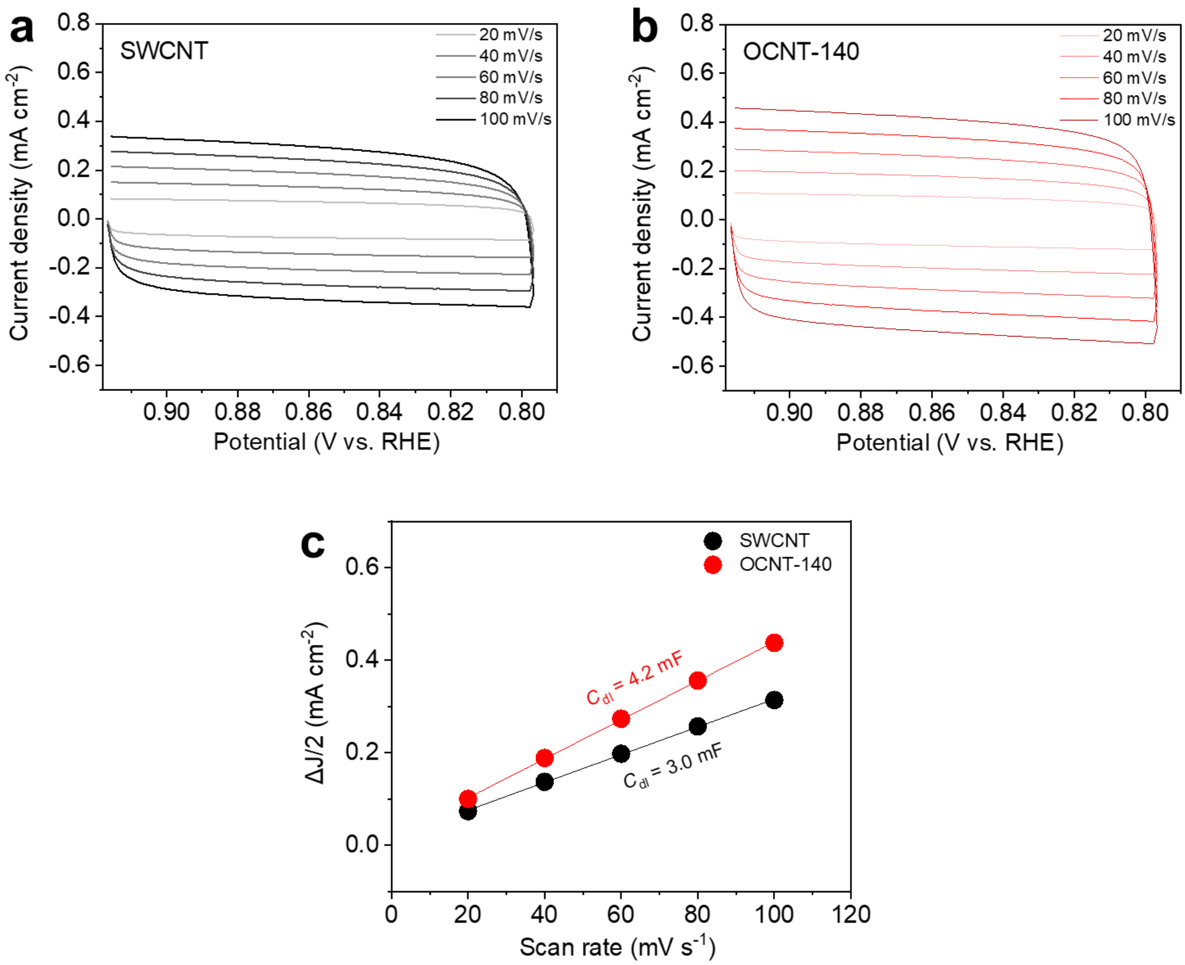

Figure R4 a-b, Cyclic voltammetry curves of SWCNT and OCNT-140 catalysts at scan rates of 20, 40, 60, 80, and 100 mV s^{-1}. c, Linear fitting of the current density at 0.86 V vs. RHE against scan rate for estimating the double-layer capacitance of the catalysts.

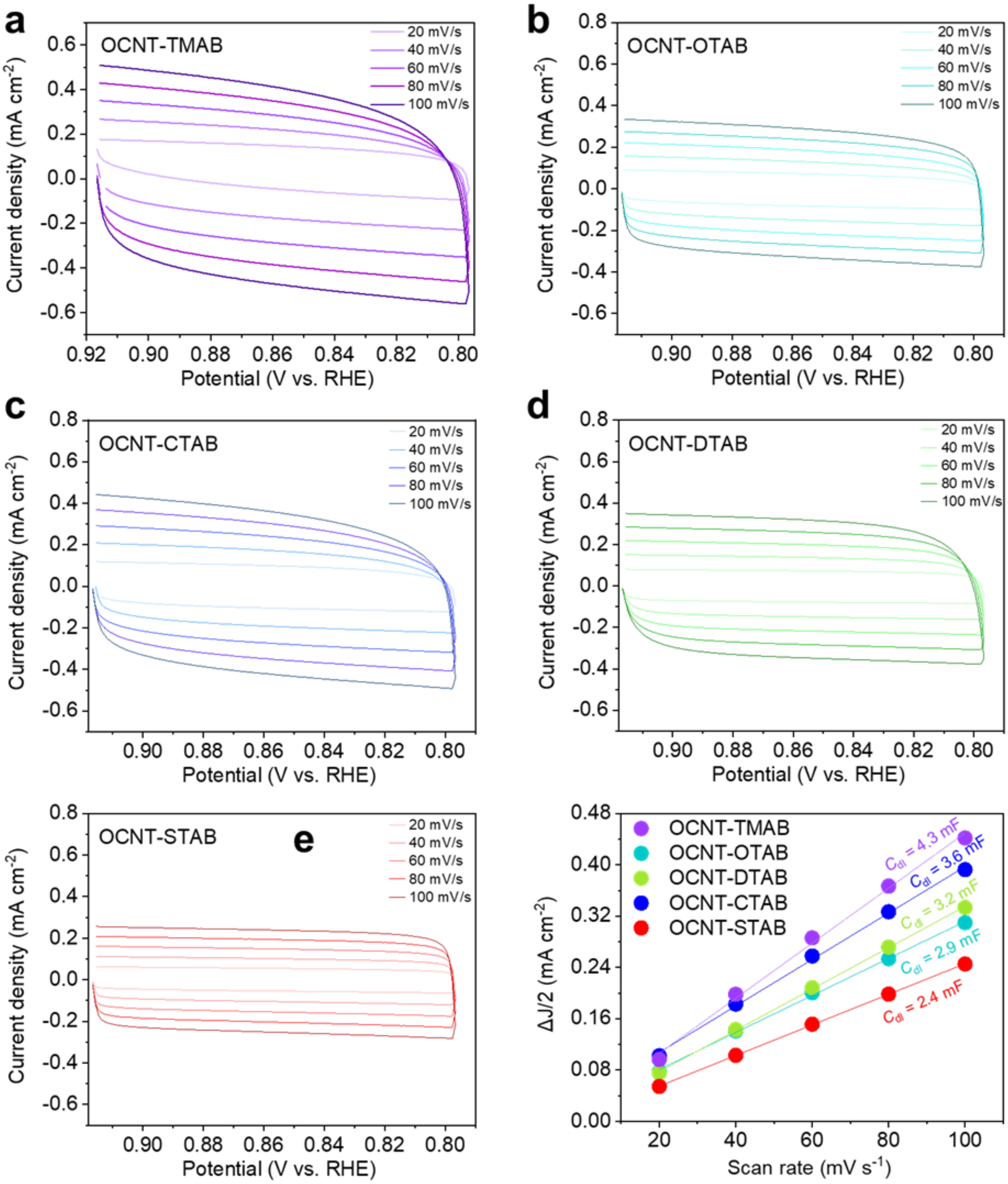

Figure R5 a-d, Cyclic voltammetry curves of OCNT-OTAB, OCNT-DTAB, OCNT-CTAB, and OCNT-STAB catalysts at scan rates of 20, 40, 60, 80, and 100 mV s^{-1}. e, Linear fitting of the current density at 0.86 V vs. RHE against scan rate for estimating the double-layer capacitance of the catalysts.

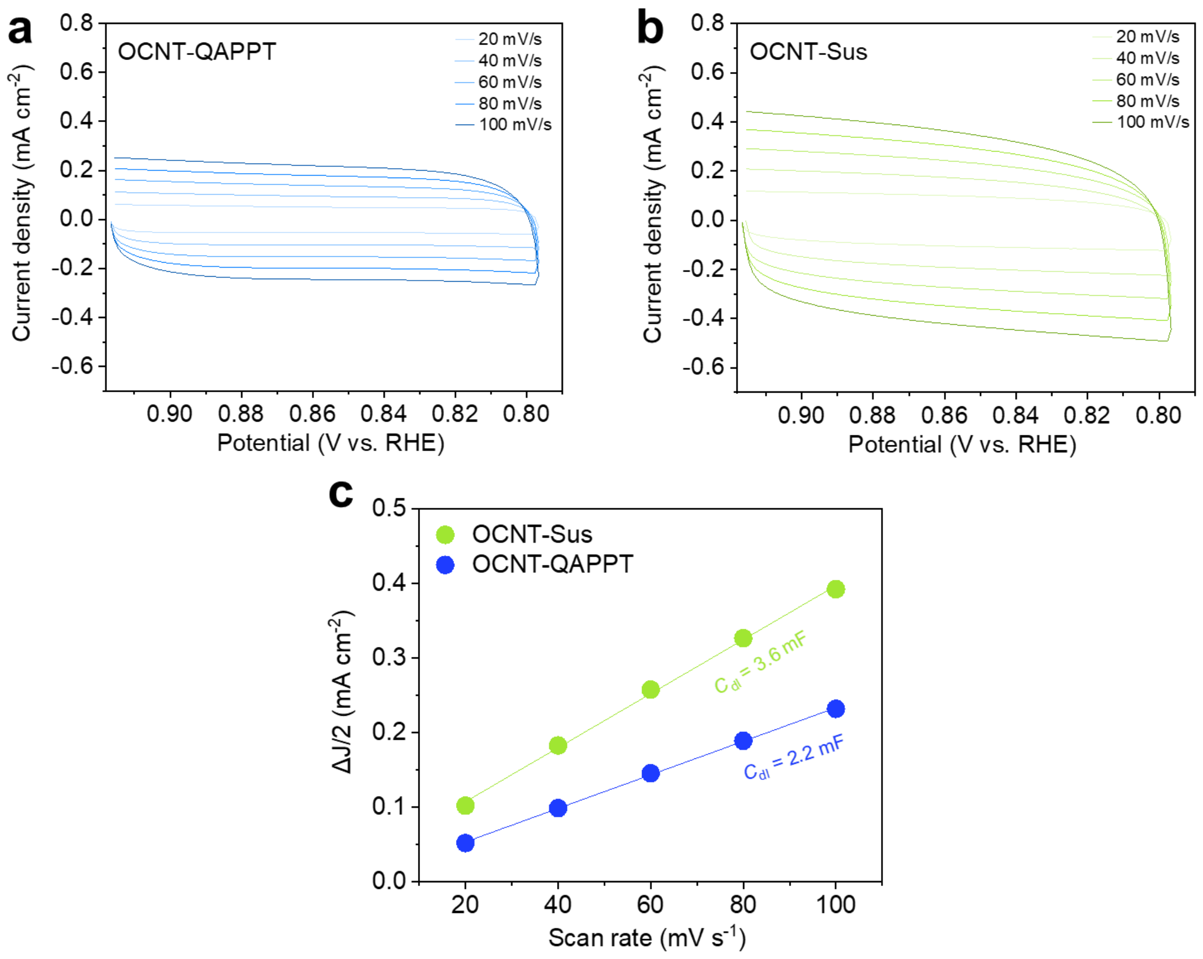

Figure R6 a-b, Cyclic voltammetry curves of OCNT-QAPPT and OCNT-Sus catalysts at scan rates of 20, 40, 60, 80, and 100 mV s^{-1}. c, Linear fitting of the current density at 0.86 V vs. RHE against scan rate for estimating the double-layer capacitance of the catalysts.

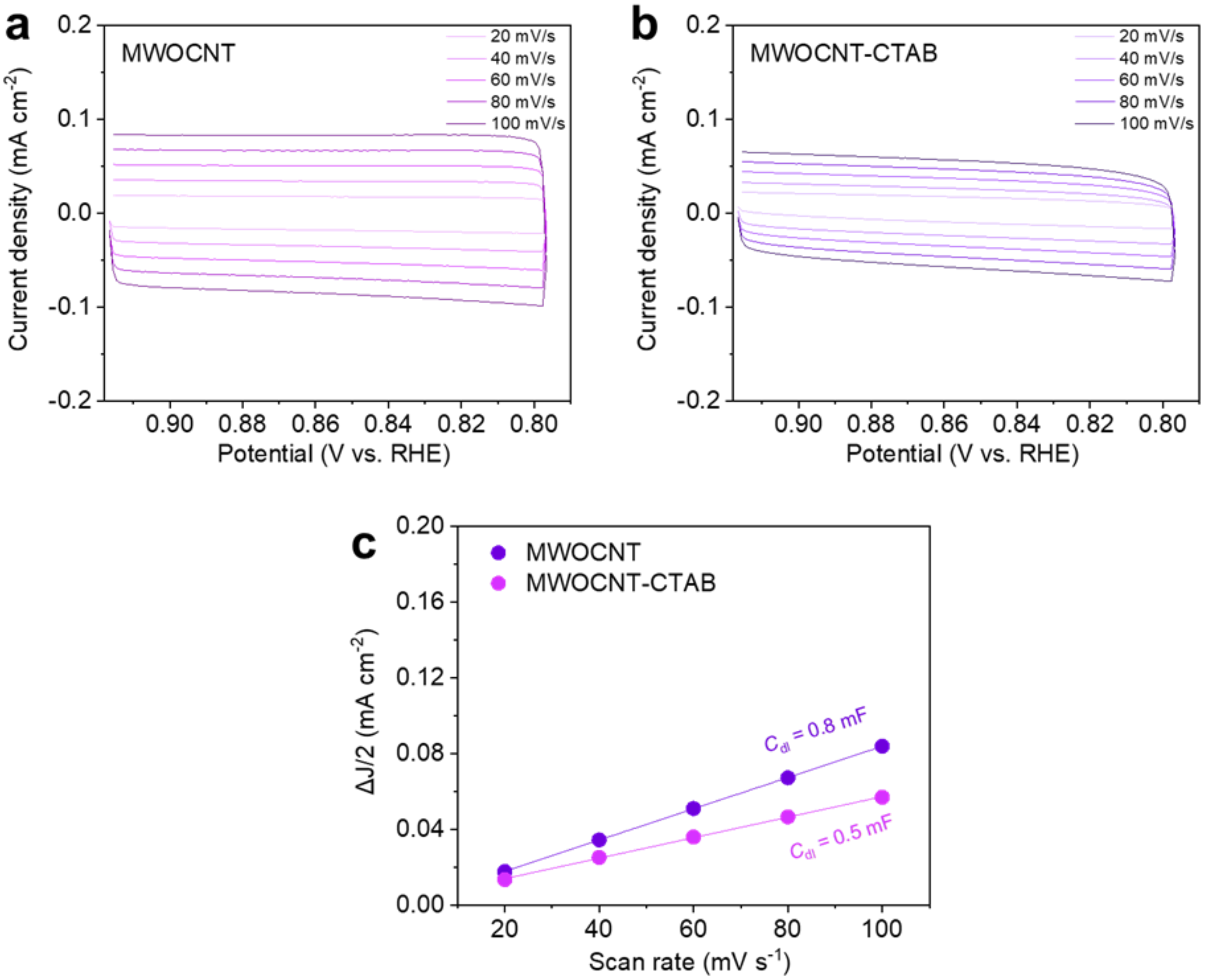

Figure R7 a-b, Cyclic voltammetry curves of MWOCNT and MWOCNT-CTAB catalysts at scan rates of 20, 40, 60, 80, and 100 mV s^{-1}. c, Linear fitting of the current density at 0.86 V vs. RHE against scan rate for estimating the double-layer capacitance of the catalysts.

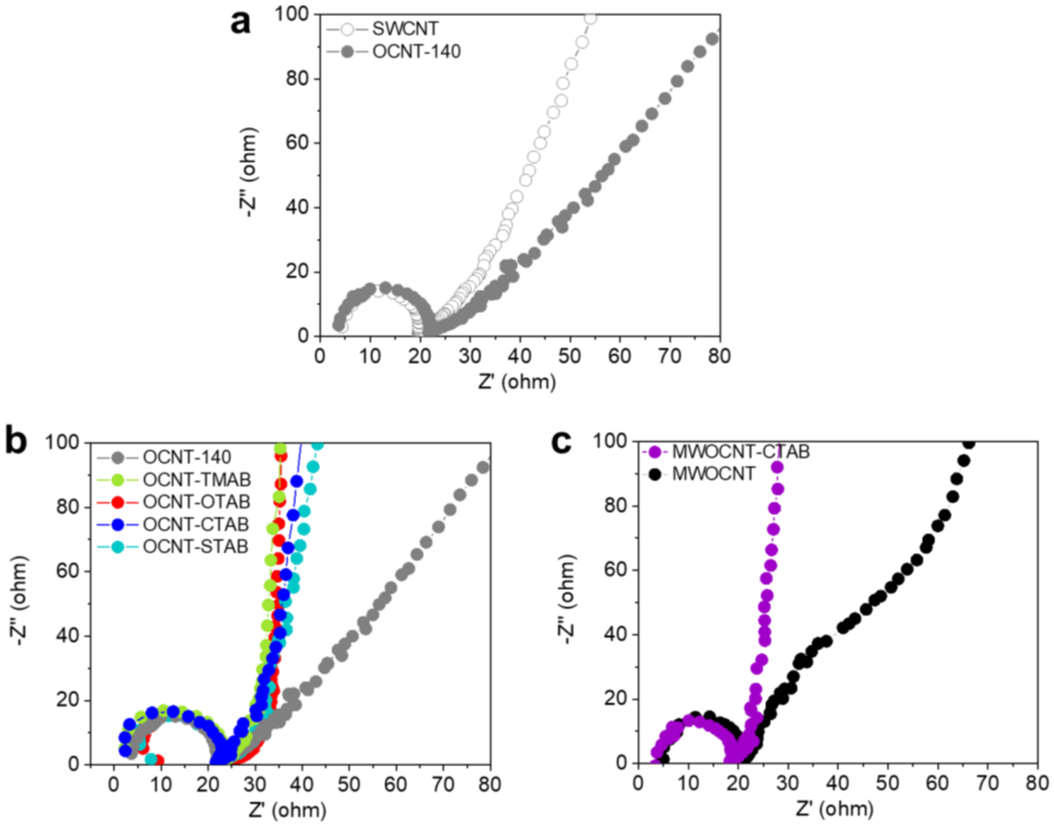

Figure R8 Electrochemical impedance spectroscopy (EIS) results. Nyquist plots of catalysts measured in 0.3 M K_2SO_4 containing 0.1 M H_2SO_4 at open-circuit voltage.

Action: We have added the supplementary figures in the Supporting Information, and the relevant discussion has been incorporated into the main text as shown below.

*"We prepared an MTAB-OCNT electrode by modifying OCNT with the short-chain cationic surfactant tetramethylammonium bromide (MTAB), which exhibited hydrophilicity comparable to that of pristine OCNT (**Supplementary Figs. 23-24**). Although its Faradaic efficiency for H_2O_2 electrosynthesis was obviously lower than that of OCNT-CTAB (especially at high current densities, **Supplementary Fig. 24a**), the catalyst still showed substantial improvement over unmodified OCNT. This demonstrates that cation modification can indeed enhance performance, but strong surface hydrophobicity remains crucial for achieving high Faradaic efficiency under high current densities.*

*The roles of electronic and mass-transfer effects from the cationic modifier were evaluated. Electrochemically active surface area analysis did not support a significant role of cationic modifier for electronic effects in the activity enhancement (**Supplementary Figs. 25-27**). Electrochemical impedance spectroscopy measurements showed that electron-transfer kinetics were largely unchanged, and all modifiers induced comparable mass-transport enhancements, indicating this was also not the key factor (**Supplementary Figs. 28**)."*

Question 3. The stability test reveals a critical flaw. The authors attribute a performance drop to “electrode flooding,” which required manual drying to remedy. The need for intermittent manual intervention represents an unacceptable failure mode for any practical or industrial process and severely undermines the reported stability claims. The authors must provide a transparent investigation into this failure mechanism. Is the performance loss due to degradation of the hydrophobic layer or leaching of the surfactant? Quantifying surfactant leaching via Total Organic Carbon (TOC) analysis of the effluent during the 200-hour stability test is essential to validate the long-term integrity of the electrode interface.

Response: We agree with this suggestion and have therefore repeated the stability test while monitoring the Total Organic Carbon (TOC) in the effluent H_2O_2 solution. The TOC value stabilized at ~0.5 mg L^{-1} after an initial period throughout the subsequent 20-hour operation, nearly equivalent to that of the fresh electrolyte (shown in **Figures R9**). The initially observed TOC may be attributed to the detachment of unstable carbon species from the OCNT in the early reaction stage. Based on contact angle (wettability) and XPS (surface composition) measurements, the electrode only exhibited a slight reduction in hydrophobicity after use, while the CTAB modifier was shown to remain effectively loaded without significant leaching (shown in **Figures R10-12**).

The observed fluctuations in Faradaic efficiency were primarily due to cathode flooding, where electrolyte penetration to the electrode backside limited oxygen diffusion. The performance decline was caused not by catalyst degradation or complete hydrophobicity loss, but by the gradual wetting of the gas diffusion layer, which transitioned from a superhydrophobic to a hydrophobic state with a contact angle of 127° during long-term operation. This mechanism was confirmed by the observed transition of the electrode backside from initially superhydrophobic to less hydrophobic after testing, which also explains why performance was restored after a simple drying step. Following a similar strategy to catalyst modification in subsequent research, an additional superhydrophobic treatment on the electrode backside should be effective in mitigating the flooding that results from the increased hydrophilicity of the gas diffusion layer.

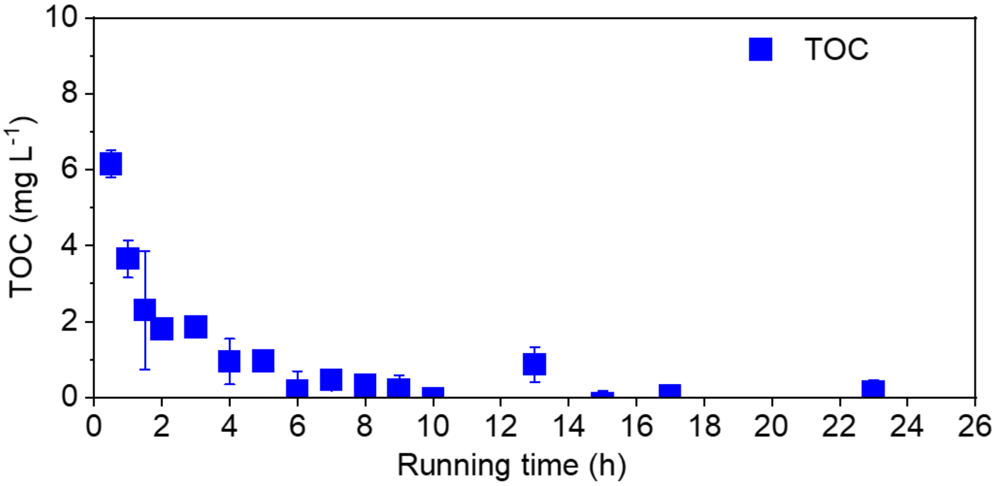

Figure R9 Total organic carbon (TOC) concentration in the effluent H_2O_2 solution during a

repeated 23-hour stability electrolysis test.

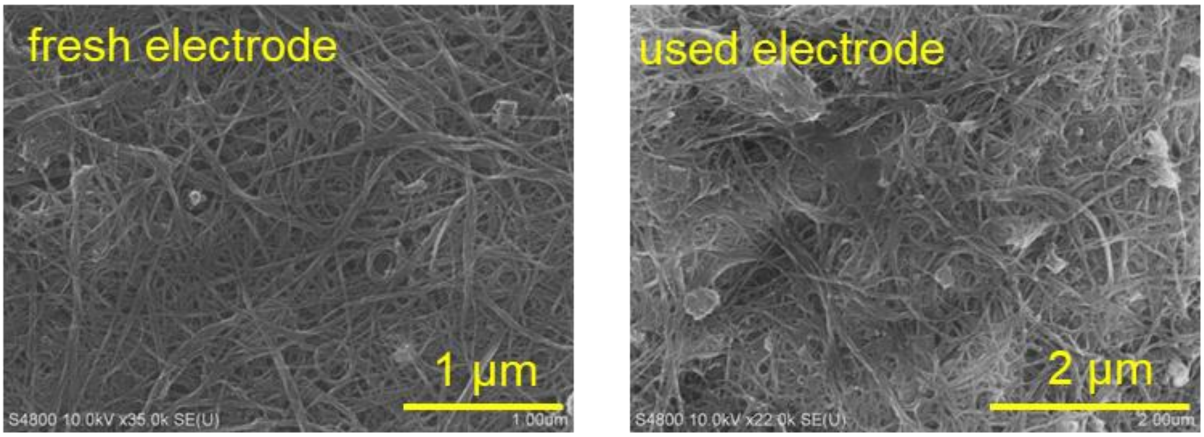

Figure R10 OCNT-CTAB electrode photographs before and after stability test conducted at 400 mA cm^{-2} for 200 hours.

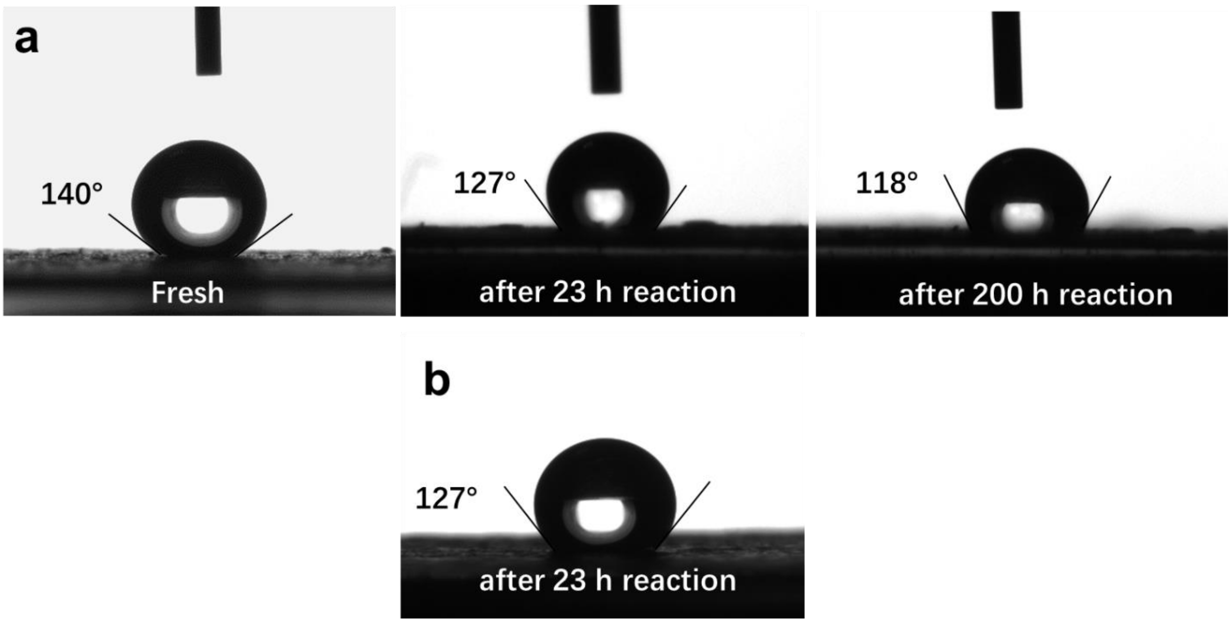

Figure R11 Water contact angles of the OCNT-CTAB electrode before and after reaction conducted at 300 mA cm^{-2} for 23 and 200 hours. a, catalytic layer; b, back side of electrode (gas diffusion layer).

Prior to reaction, the backside GDL surface was superhydrophobic, with a contact angle estimated at >150°. In this state, droplets beaded up and rolled off easily, making stable measurement unattainable.

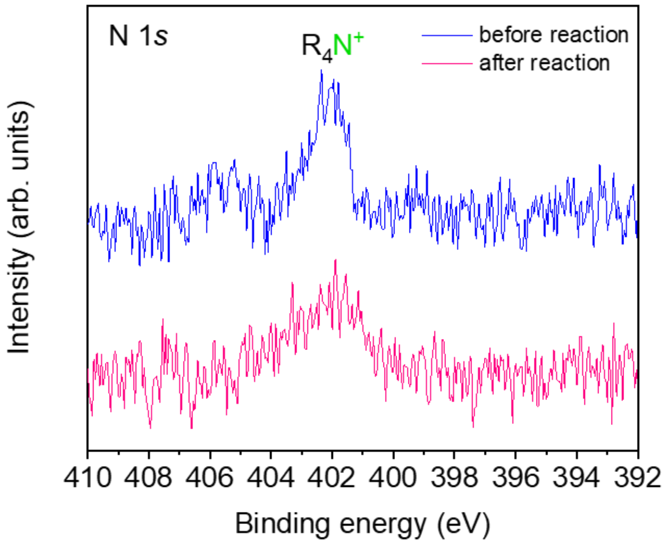

Figure R12 XPS N 1s high-resolution spectra of the OCNT-CTAB electrode after reaction conducted at 300 mA cm^{-2} for 200 hours.

Action: In response to the Reviewer's comment, we have incorporated the new figures (**Supplementary Figs. 50-52**) into the revised Supplementary Information and added the corresponding discussion in the main text (Lines 373-379), as detailed below.

*"Electrode stability was assessed by monitoring the effluent TOC and analyzing surface properties. The TOC value of the effluent stabilized at approximately 0.5 mg L^{-1} throughout the subsequent 20-hour operation, a level consistent with that of the fresh electrolyte (**Supplementary Fig. 49**). Furthermore, post-reaction characterization via contact angle (wettability) and XPS (surface composition) measurements, the electrode only exhibited a slight reduction in hydrophobicity after use, while the CTAB modifier was shown to remain effectively loaded without significant leaching (shown in **Supplementary Fig. 50-52**)."*

Question 4. Natural seawater's high chloride concentration (~0.5 M) presents a significant, unaddressed challenge. At the operating conditions (pH ~1, high potentials), the Chlorine Evolution Reaction (CER) is a thermodynamically favorable parasitic reaction. Moreover, chloride ions are known to poison certain catalytic sites. The manuscript focuses on the competition between $2e^-$ and $4e^-$ ORR but largely overlooks the potential interference from chloride. The authors must demonstrate high selectivity against CER by testing for active chlorine species (e.g., Cl_2, HOCl) in the product stream. Furthermore, cyclic voltammetry studies in Cl^--containing and Cl^--free (e.g., with SO_4^{2-} as the non-interfering anion) electrolytes are needed to investigate whether Cl^- competitively adsorbs on active sites or alters the ORR onset potential.

Response: We thank the reviewer for the important suggestions regarding the influence of Cl^- ions on the system. We have addressed the points raised in three main aspects.

1. The chlorine evolution reaction (CER) that produces Cl_2 or HClO via chloride oxidation

is an anodic reaction occurring at the anode. In our system, the chloride-containing seawater is used only in the catholyte and the anode employs 0.5 M H_2SO_4 electrolyte, which are separated by a proton exchange membrane. In principle, only cations migrate from the anolyte to the catholyte through the membrane during electrolysis, with no anions or cations migrating from the cathode to the anode. To confirm it, we monitored chloride and metal ions in the anolyte during H_2O_2 electrosynthesis, and no significant amounts of Cl^- ions or metal cations (Na^+, K^+, Ca^{2+}, Mg^{2+}) were detected (**Figure R13** below). Therefore, CER to generate Cl_2 or HClO should not occur in the current study.

2. We investigated the effect of Cl^- on the electrochemical synthesis of H_2O_2 at the cathode. First, according to the above suggestions, cyclic voltammetry (CV) tests were performed in three types of electrolytes K_2SO_4, K_2SO_4+KCl, and KCl solutions, all with the same total ion concentration, to evaluate ORR activity. OCNT and a series of cationic modified OCNT catalysts were evaluated. As shown in **Figure R14-15**, under an inert atmosphere (Ar), the CV curve shows no distinct peaks; the current is primarily attributed to non-Faradaic processes (double-layer charging). In an oxygen-saturated atmosphere (with O_2), a pronounced reduction current peak appears, corresponding to the reduction of O_2 on the catalyst surface, with the peak potential located at approximately 0.4 V. Notably, compared to the pure K_2SO_4 electrolyte, the reduction peak potential shifts positively in the Cl^--containing electrolyte. This positive shift indicates faster reaction kinetics, meaning that the ORR can proceed at a higher rate under a lower overpotential.

   We further employed the RRDE technique to assess ORR activity and $2e^-$ selectivity. As shown in **Figure R16**, the OCNT-140 catalyst exhibited an overpotential of ~0.2 V, referenced to a standard potential of 0.7 V for $2e^-$ ORR. While this overpotential remained virtually unchanged with or without Cl^- in the electrolyte, both the current density and H_2O_2 selectivity were significantly enhanced in the presence of Cl^-. This phenomenon was similarly observed for other catalysts.

3. These findings demonstrate that Cl^- plays a significant role in promoting the $2e^-$ ORR, and we further investigate the underlying reasons. We infer that the introduction of Cl^- is unlikely to significantly alter the electronic structure or the nature of the key active sites, which explains why the onset potential remains largely unchanged. Instead, Cl^- likely modulates the local environment or double-layer structure at the catalyst surface, enhancing the reaction rate (as reflected by the increased current). This is supported by the CV data obtained under Ar atmosphere: in the 0.8–1.0 V region, the presence of Cl^- promotes higher non-Faradaic current, confirming an enhancement in capacitive behavior.

   To further verify whether Cl^- influences the $2e^-$ ORR process by directly interacting with catalytic sites or by altering double-layer properties, DFT calculations were performed to investigate Cl^- adsorption behavior on various active sites. As shown in

Figure R17-18, Cl^- exhibits weak adsorption on most carbon sites evaluated in this work, making it difficult to compete with the *OOH intermediate for adsorption. Only the C7 site shows favorable Cl^- adsorption; however, this site is not an efficient ORR active site due to its weak interaction with *OOH.

These results collectively suggest that the enhanced H_2O_2 electrosynthesis in the presence of Cl^- likely stems from its modification of the double-layer properties, which in turn affects the environment and behavior of intermediates involved in the ORR process.

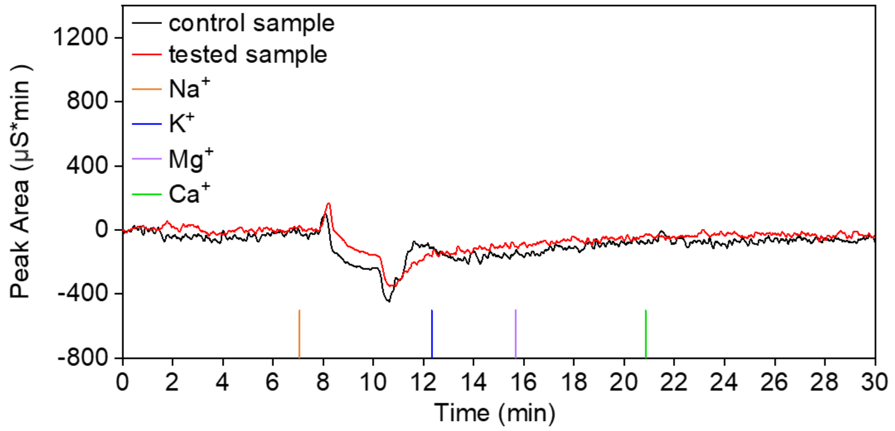

Figure R13 Ion chromatographic analysis of the sample. The signal peaks were compared with those of standard Na^+, K^+, Mg^{2+}, and Ca^{2+}. (The tested sample was the anode effluent from a system operated for 2 h at 500 mA cm^{-2}, with seawater as the cathode and 0.5 M H_2SO_4 as the anode. The control was the anode effluent from a system operated under the same duration and current, using 0.3 M K_2SO_4 + 0.1 M H_2SO_4 as the cathode and 0.5 M H_2SO_4 as the anode.)

No detectable Cl^- was found within the instrument's detection limit. Both Cl^- and metal ions (Na^+, K^+, Mg^{2+}, Ca^{2+}) weren't detected in the anode effluent above the instrument's detection limit during H_2O_2 electrosynthesis. The absence of these species indicates negligible ion crossover, thereby minimizing the risk of adverse side reactions at the anode.

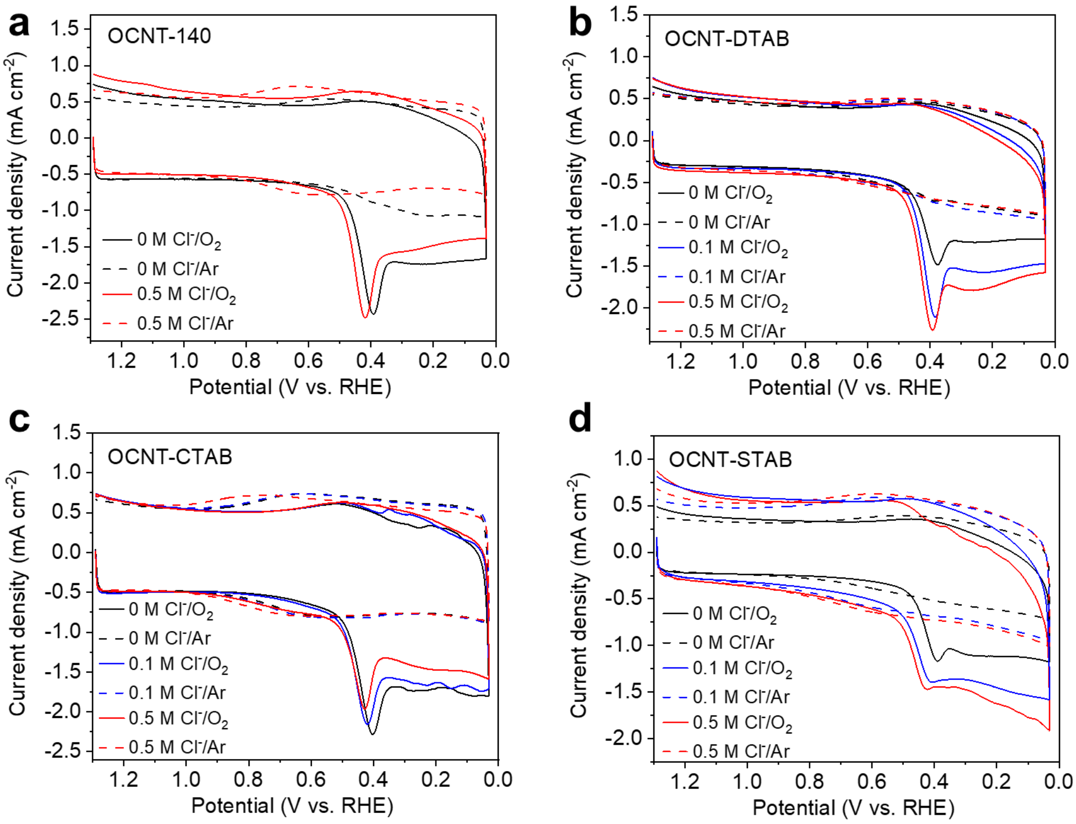

Figure R14 Assessment of the Cl^- effect on the ORR: Cyclic voltammetry curves of OCNT-140, OCNT-DTAB, OCNT-CTAB, and OCNT-STAB catalysts at a scan rate of 50 mV s^{-1} in electrolytes containing different Cl^- ratios; the corresponding ion composition of each solution is listed in **Table R1**.

Table R1 Ion compositions of solution used for ORR tests.

Solutions	K^+ concentrations (M)	Ion compositions (M)
0 M Cl^-	0.5 M	0.25 M K_2SO_4
0.1 M Cl^-	0.5 M	0.2 M K_2SO_4+0.1 M KCl
0.5 M Cl^-	0.5 M	0.5 M KCl

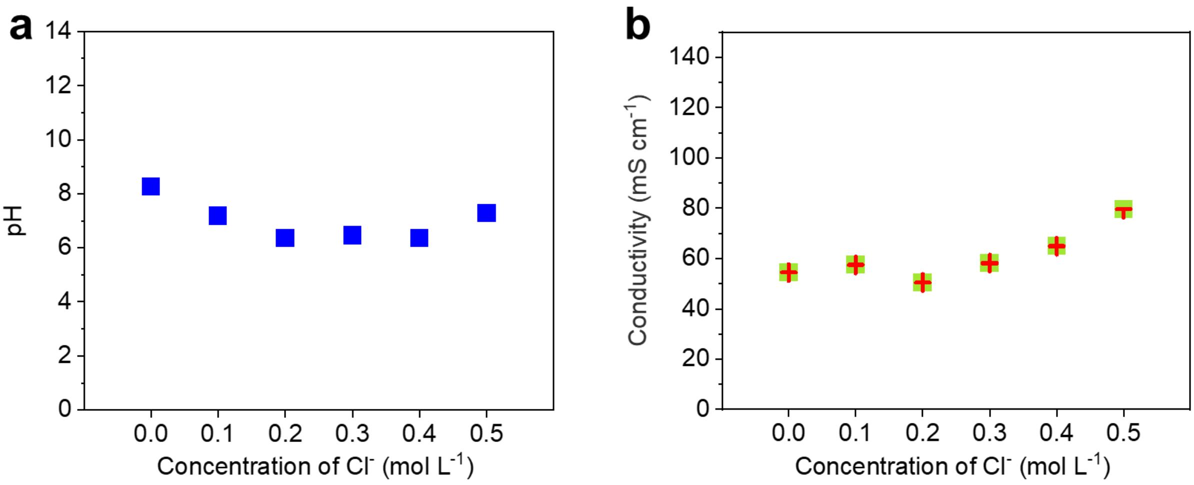

Figure R15 The pH and conductivity of electrolytes containing different Cl^- ratios.

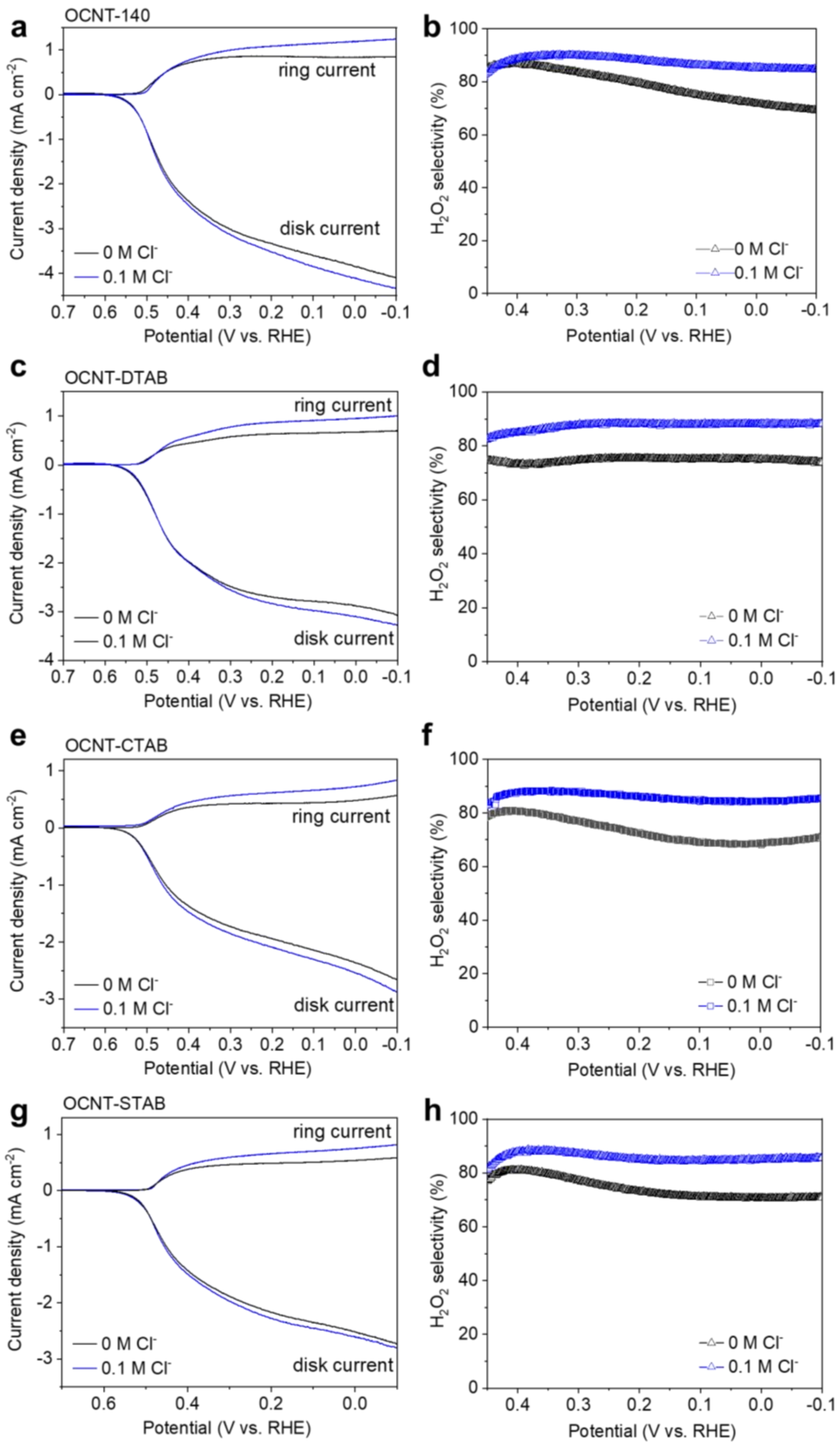

Figure R16 Assessment of the Cl^- effect on the ORR selectivity for H_2O_2 production. Rotating ring-disk electrode (RRDE) tests were performed on OCNT-140, OCNT-DTAB, OCNT-CTAB, and OCNT-STAB catalysts at a scan rate of 10 mV s^{-1} in electrolytes with and without 0.1 M Cl^-. For each catalyst, the left panel (a, c, e, g) shows the ORR polarization curve and the corresponding H_2O_2 oxidation current, while the right panel (b, d, f, h) shows the calculated H_2O_2 selectivity.

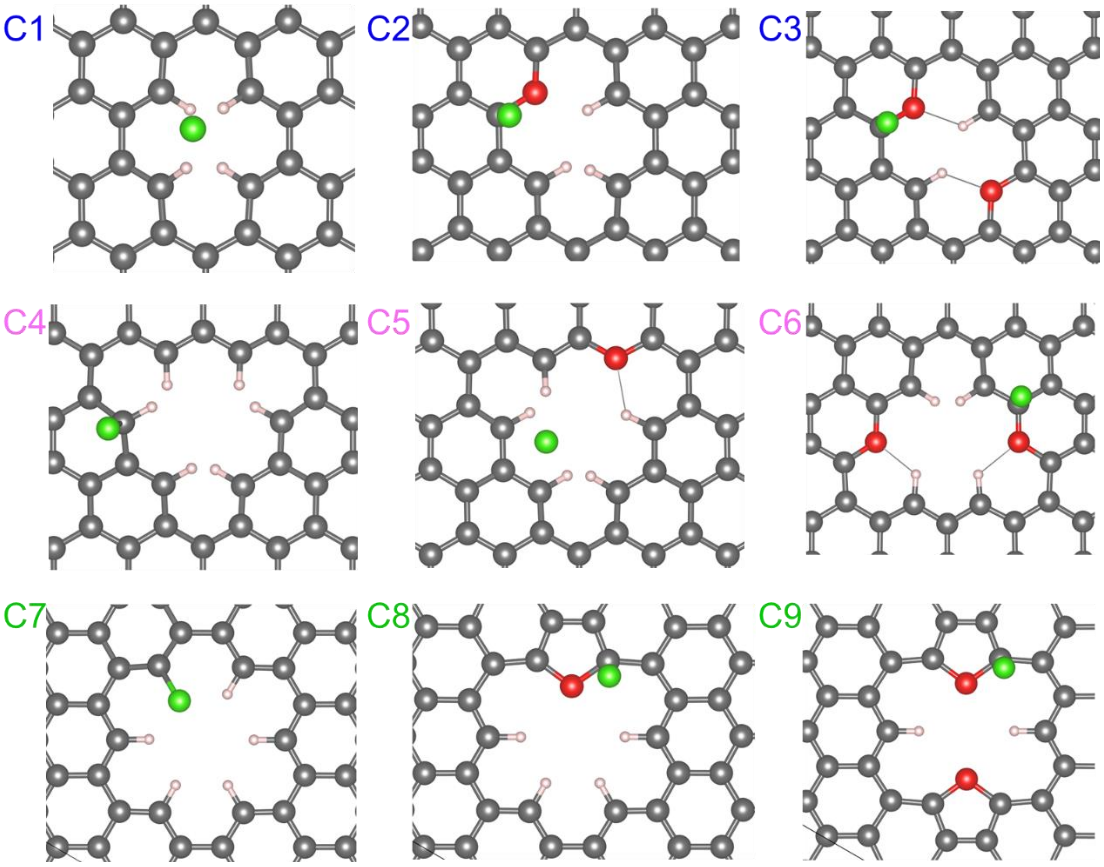

Figure. R17 Chloride ion adsorption on different defect carbon and oxygen-modified carbon (white, gray, red, and green spheres represent H, C, O, and Cl atoms, respectively).

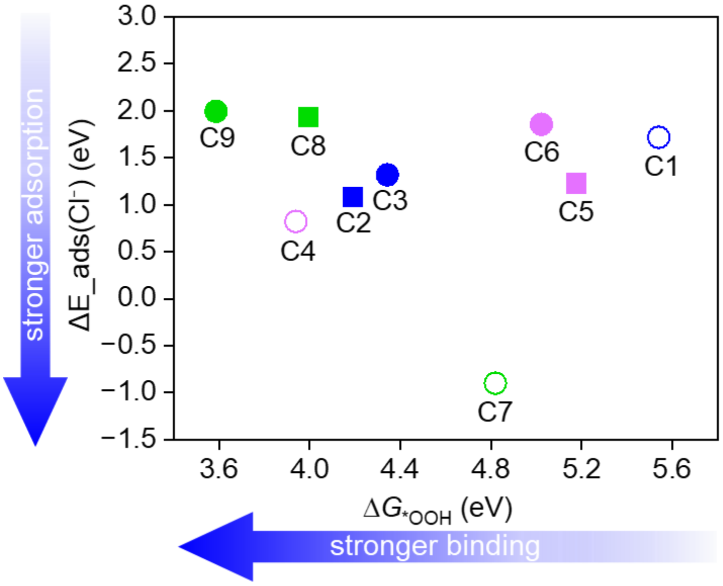

Figure R18 Relationship between Cl^- adsorption energies at 0 V and *OOH binding energies on defect-engineered and oxygen-modified carbon sites.

Action: In response to the Reviewer's comment, the new figures have been included as

Supplementary Figs. 58-62 in Supporting Information and corresponding discussion has been added to the main text (Lines 396-413). The updated text is provided below.

*"We evaluated the effect of Cl^- on ORR activity and $2e^-$ selectivity via CV and rotating ring-disk electrode (RRDE) measurements. CV results (**Supplementary Figs. 58-59**, **Table 1**) showed that the ORR peak potential shifted positively upon adding Cl^- to the K_2SO_4 electrolyte, suggesting faster reaction kinetics in the presence of Cl^-. RRDE results (**Supplementary Fig. 60**) showed that the OCNT-CTAB catalyst exhibited an overpotential of 0.2 V for the $2e^-$ ORR. Although this overpotential remained virtually unchanged with or without Cl^- in the electrolyte, both the current density and H_2O_2 selectivity were significantly enhanced in the presence of Cl^-. This phenomenon was similarly observed for all tested catalysts. To further verify whether Cl^- influences the $2e^-$ ORR process by directly affecting the activity of catalytic sites or by altering double-layer properties, DFT calculations were performed to investigate the adsorption behavior of Cl^- on the active sites. As shown in **Supplementary Figs. 61-62**, Cl^- exhibited weak adsorption on various carbon sites evaluated in this work, making it difficult to compete with the *OOH intermediate for adsorption. We thus infer that the introduction of Cl^- is unlikely to significantly alter the electronic structure or the nature of the key active sites, which explains why the onset potential remains largely unchanged. Instead, Cl^- likely modulates the local environment or double-layer structure at the catalyst surface, enhancing the H_2O_2 electrosynthesis."*

Question 5. The AIMD analysis (Fig. 3), which suggests that cations prevent O-O cleavage at the electrified interface, requires clarification. The simulation setup is not sufficiently detailed. How was the surface coverage of cations determined, and was this density correlated with experimental loadings on the OCNTs? While the proposed "proton depletion" mechanism is intriguing, the link between the presented AIMD snapshots and the macroscopic electrochemical performance remains qualitative rather than quantitative.

Response: Thank you for the reviewer's important suggestions regarding AIMD analysis. We have addressed the points raised in four main aspects.

1. In **Fig. 3e–f**, the changes in the O–O and C–O bond lengths were monitored to determine whether O–O bond cleavage occurs and whether the adsorbates remain adsorbed or undergo desorption at the electrified interface. We have added the following detailed description in the main text.

*"The transformations of *O_2 and *OOH take place on the carbon sites of the catalyst; therefore, we monitored the O–O and C–O bond lengths to examine whether O–O bond cleavage occurs and whether these adsorbates remain adsorbed or undergo desorption. As shown in **Figure 3e** and **Fig. S33**, during the hydrogenation of *O_2 on the bare C2 site, both the O–O and C–O bond lengths remained nearly unchanged, indicating that the O–O bond scission did not occur, *OOH was successfully formed, and it remained stably adsorbed on the carbon site. In contrast, during the subsequent hydrogenation of *OOH, the O–O bond length increased abruptly from 1.5 Å to over 5 Å, signaling O–O bond cleavage, while the C–O bond shortened slightly from 1.56 Å to 1.23 Å. The simulation results revealed that at the C2-H_2O interface, *OOH formed readily from O_2 protonation but underwent rapid decomposition, which sharply contrasted with the moderate *OOH binding strength predicted for vacuum conditions."*

*Previously, "At the C2-H_2O interface, *OOH formed readily but underwent decomposition (**Figure 3e, Fig. S25**), contrasting with the stable binding predicted in vacuum calculations. This suggests that the electrified acidic environment over-stabilizes *OOH beyond the optimal range defined by the volcano relationship, thereby suppressing H_2O_2 formation."*

2. We added the description on the simulation setup in the revised manuscript, as follows, Line 296-298 in the revised manuscript:

"*We further employed AIMD to simulate the $2e^-$ ORR pathway toward H_2O_2 via sequential hydrogenation of *O_2 to *OOH and *OOH to *H_2O_2 at cationic-modified carbon interfaces within H_2O/H_3O^+ electrolyte. To more realistically model the $2e^-$ ORR to H_2O_2 under practical electrified acidic interface, we introduced an H_3O^+ ion into the water layer (pH 0) above the catalyst slab (**Supplementary Fig. 32**).*"

Description followed by Supplementary Fig. 32:

"*This addition of H_3O^+ triggers spontaneous charge compensation at the electrocatalytic interface, injecting an equivalent number of electrons into the catalyst surface to maintain electroneutrality and modulate the electrode potential. As a result, the electrode potentials are established at −0.94 V vs. RHE for C2-H_2O-H^+ and −1.12 V vs. RHE for C2-OTAB-H_2O-*

*H^+. Although these potentials are sufficiently negative to drive O_2 protonation in principle, they likely concurrently exceed the theoretical threshold for cleaving the O–O bond in the *OOH intermediate, which promotes H_2O formation and leads to low H_2O_2 selectivity.*"

3. The surface coverage of cations was correlated with the experimental loadings on the OCNTs. XPS measurements of the cation-modified OCNT catalysts showed an N/C atomic ratio of approximately 1/98–1.5/98, which aligns well with the N/C ratio of 1/68 used in our model.

4. Here, the "proton depletion" condition at the charged interface is considered as a possible scenario to investigate the effect of cationic modifiers on ORR properties. Previous studies have indicated that under high-current-density cathodic electrolysis in acidic media, rapid consumption of H^+ leads to the formation of a proton-deficient region near the electrode interface, even though the bulk electrolyte remained acidic. (*Nat. Catal.* 2022, 5, 564-570; *Science* 2020, 367*; Angew Chem Int Edit* 2024, 63), which can enhance H_2O_2 efficiency.

   To qualitatively link the presented AIMD snapshots with macroscopic electrochemical performance, we have supplemented the study with electrochemical testing and in-depth analysis of the influence of H^+ with cationic modifier on ORR selectivity. We conducted H_2O_2 performance tests at pH 0.8 and pH 1.5 (**Figure R19**). The results indicated that H_2O_2 efficiency under pH 0.8 and pH 1.5 remains closely comparable to that at pH 1.0 under high current densities. At low current densities, unlike earlier work which showed a strong dependence of efficiency on pH, the efficiency at pH 0.8 is only slightly lower than that at pH 1.0 and 1.5. This may be attributed to electrostatic repulsion between the cationic modifiers and H^+, which helps maintain H_2O_2 efficiency even under high proton concentrations.

   We further investigated the effect of cationic modifiers on H^+ distribution at the electrode surface and its hydrogenation behavior toward reactants. OTAB modification was found to repel H^+. This repulsive effect was proven to be independent of the specific catalytic sites, as it occurred consistently on carbon with different catalytic sites. Regarding the hydrogenation behavior of H^+ towards H_2O_2, at the bare C2 site, our evolution trajectory indicated that H_3O^+ transfers to the $*O_2$/*OOH via the hydrogen bond network for hydrogenation whether it approaches or moves away from the catalytic site initially. However, when H^+ is close to the interface, the proximity of H_3O^+ to the catalytic site leads to excessively strong interactions with *OOH, resulting in its decomposition due to the induced electric field effects and stronger hydrogen-bonding interactions. In contrast, the presence of the OTAB modifier at the C2 site repels H_3O^+, which suppresses the direct hydrogenation of $*O_2$/*OOH by shielding it from H_3O^+. Charge analysis of *OOH adsorbed at the catalytic site indicates that electron is transferred from the C site to the bonding O atom (**Figures R20-21**), a process accompanied by O–O bond elongation on the CTAB-modified surface. This weaker interaction on the CTAB-modified carbon contributes to the preferential desorption of *OOH instead of bond cleavage. These results underscore that

*OOH formation is feasible under both proton-rich and proton-poor conditions at highly negative potentials, the reaction selectivity is ultimately governed by cation-modulated *OOH desorption.

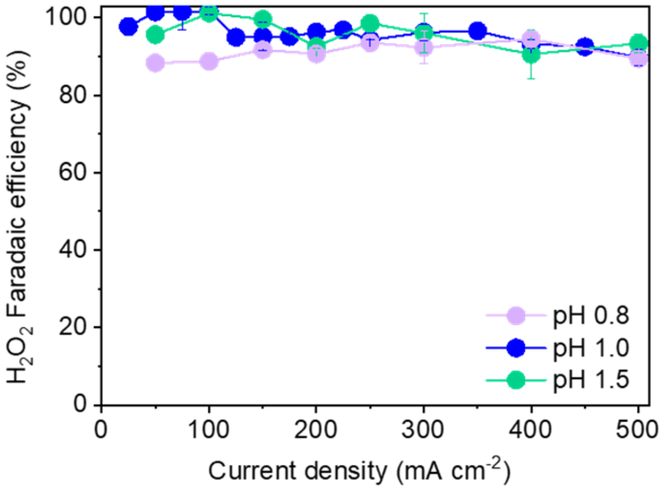

Figure R19 Performance evaluation of the effect of catholyte pH on acidic H_2O_2 electrosynthesis using OCNT-CTAB electrode.

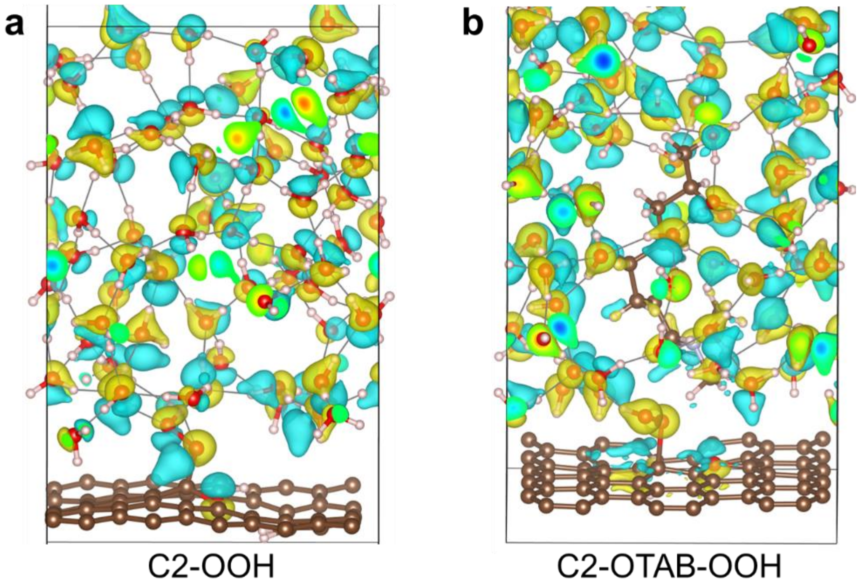

Figure 20 Differential charge density distributions of adsorbed *OOH on the C2-H_2O and C2-OTAB-H_2O surfaces. In the differential charge density map, blue region indicates electron density depletion (loss), and yellow indicates accumulation (gain).

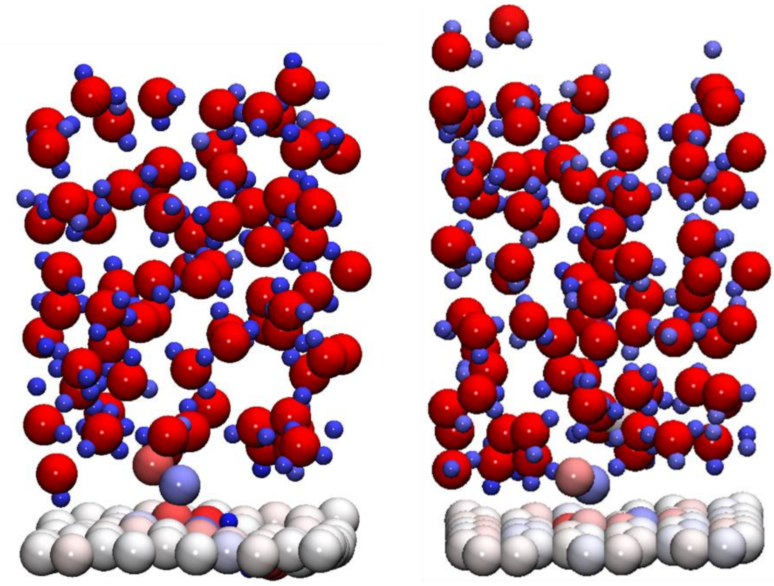

Figure R21 Atom charge distribution of adsorbed *OOH on the C2-H_2O and C2-OTAB-H_2O surfaces. Red and blue indicate electron loss and gain, respectively. The results show charge depletion at the C atom and charge accumulation at the O atom of adsorbed *OOH.

Action: We have included the newly added experiments as **Supplementary Figs. 14, 40 and 42** in the Supporting Information, and added the updated text into the revised manuscript (Line 319-340).

*"Cationic modifiers primarily function by electrostatically regulating the surface potential, we examined their effect on modulating H^+ aggregation and the ensuing hydrogenation behavior at the catalytic interface[56-58]. Previous studies indicated that during high-current-density electrolysis in acid, rapid H^+ consumption creates a proton-deficient interfacial region that can enhance H_2O_2 efficiency [22, 59, 60]. We investigated the effect of cationic modifiers on H^+ distribution at the electrode surface. Results indicated that OTAB modification can repel H^+, which occurred consistently across different carbon sites (C2, C3, C8) (**Supplementary Figs. 35-37**). This supports the notion that modifiers can influence the local proton activity during high-current-density operation. Regarding the hydrogenation behavior of H^+ towards H_2O_2, the evolution trajectory revealed that at the bare C2 site, the proximity of H^+ (as H_3O^+) to the interface induces strong interactions with *OOH, leading to its decomposition. In contrast, keeping H^+ away from the interface prevents this cleavage but was insufficient to promote efficient *OOH desorption (**Supplementary Figs. 33, 38**). By contrast, the OTAB modifier repelled H_3O^+ by shielding *O_2/*OOH, thereby suppressing their direct hydrogenation and promoting the alternative proton-transfer pathway from H_2O (**Supplementary Figs. 34** and **39**). Charge analysis reveals electron transfer from the C site to the bonding O atom in adsorbed *OOH, accompanied by O–O bond elongation on the CTAB-modified surface (**Supplementary Figs. 40-41**). The resulting weaker interaction*

*promoted *OOH desorption rather than O–O cleavage. These results indicate that while *OOH formation is feasible under both proton-rich and -deficient conditions at highly negative potentials, the reaction selectivity is ultimately determined by the cation-modulated desorption of *OOH."*

Previously: *"Previous studies have shown that high-current-density cathodic electrolysis induces a proton-depleted electrode interface due to rapid H^+ consumption[22, 56, 57]. We hypothesized that such depletion could influence *OOH desorption. Simulations of proton-free C2-H_2O interface revealed that although H^+ depletion weakened *OOH adsorption as expected, this weakening alone was insufficient to trigger significant *OOH desorption or HO_2^- release (**Fig. S28**). In contrast, cation-modified interface maintained efficient *O_2 hydrogenation and *OOH desorption even under proton-deficient conditions (**Fig. S29**). These results imply that *OOH formation remains feasible at highly negative potentials regardless of interfacial proton availability, provided that cationic modifiers maintain near-optimal binding strength. The critical determinant of selectivity is thus *OOH desorption, which is facilitated by cation-induced electronic modulation."*

Question 6. The experiments utilize a membrane-separated cell (H-cell or flow cell), presumably with a cation exchange membrane (CEM/PEM) given the acidic anolyte (H_2SO_4). In such a system, cations (Na^+, Mg^{2+}, Ca^{2+}) from the seawater catholyte are expected to migrate across the membrane towards the anode. The authors should discuss the implications of this ion crossover on long-term anode stability, membrane integrity, and the purity of the produced H_2O_2.

Response: Thank you for the important suggestion regarding the possible crossover of cations from the seawater catholyte to the anolyte in this system. We have addressed this point by monitoring metal ions in the anolyte during H_2O_2 electrosynthesis, and no significant amounts of metal cations (Na^+, K^+, Ca^{2+}, Mg^{2+}) were detected. Therefore, the influence of such ion crossover on anode stability and membrane integrity should be minimal. Furthermore, since H_2O_2 is produced and remains in the seawater catholyte compartment, the purity of the generated H_2O_2 is not directly affected by any potential minor cation crossover.

Action: We have included the added test and discussion as **Supplementary Fig. 68** in the Supporting Information, as follows.

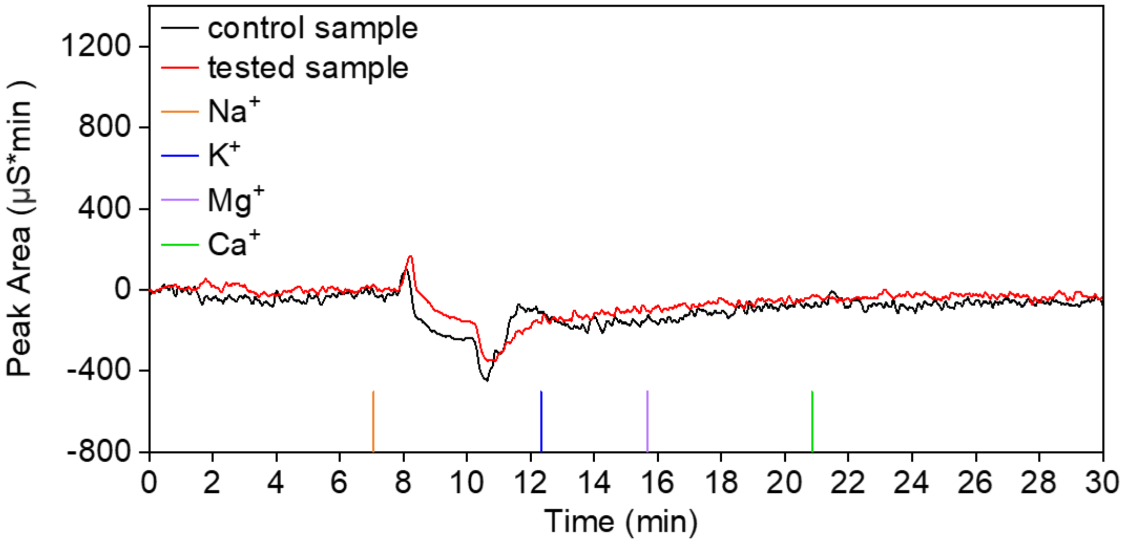

Supplementary Fig. 68 Ion chromatographic analysis of the sample. The signal peaks were compared with those of standard Na^{+}, K^{+}, Mg^{2+}, and Ca^{2+}. (The tested sample was the anode effluent from a system operated for 2 h at 500 mA cm^{-2}, with seawater as the cathode and 0.5 M H_2SO_4 as the anode. The control was the anode effluent from a system operated under the same duration and current, using 0.3 M K_2SO_4 + 0.1 M H_2SO_4 as the cathode and 0.5 M H_2SO_4 as the anode.)

No detectable Cl^{-} was found within the instrument's detection limit. Both Cl^{-} and metal ions (Na^{+}, K^{+}, Mg^{2+}, Ca^{2+}) weren't detected in the anode effluent above the instrument's detection limit during H_2O_2 electrosynthesis. The absence of these species indicates negligible ion crossover, thereby minimizing the risk of adverse side reactions at the anode.

Question 7. In Tables S1/S2, the authors compare their performance metrics with literature values. For a fair and meaningful comparison, the authors must explicitly state the pH conditions for each cited work. Many studies on H_2O_2 production in "seawater" are conducted at neutral pH. Comparing performance across different pH regimes without providing proper context regarding overpotentials and thermodynamic potentials can be misleading.

Response: In the original supporting information, we specified that the cited studies primarily used 0.1 M or 0.5 M $HClO_4$ or H_2SO_4 as the acidic catholyte, with an estimated pH range of 0.5–1.0. We have now added the reported overpotentials and H_2O_2 selectivity (from RRDE measurements) for the cited seawater H_2O_2 catalysts in the table below (Supplementary Table 5 of Supporting Information).

Supplementary Table 5 Recently reported high-performance electrocatalysts and their current densities and Faradaic efficiencies for electrochemical 2e$^-$ ORR to produce H_2O_2 in simulated or natural seawater.

Catalysts	Onset potential (V vs RHE)/ H_2O_2 selectivity	Currents density (mA cm^{-2})	FEs (%)	Production rate	Catholytes	References
Co-N-C	0.55 V/93.7% (0.5 M NaCl)	50	95.6%	4.5 mol g_{cat}^{-1} h^{-1}	0.5 M NaCl	13
$NiPS_3$	0.564 V/82% (0.5 M NaCl)	60	84%	/	seawater	14
Nanocarbon	0.57 V/95% (0.5 M NaCl)	~50	80 %	1200 mg L^{-1} h^{-1}	simulated seawater	15
$Ni(OH)_2$/CNT	~0.45 V/~90% (3.6 wt.% NaCl)	100	~80 %	1420 μmol cm^{-2} h^{-1}	natural seawater	16
OCNT-CTAB	~0.5 V/90% (0.1 M KCl+0.3 M K_2SO_4)	500	~89%	~8400 μmol cm^{-2} h^{-1}	acidified seawater	This work

Question 8. The manuscript contains minor grammatical errors and colloquialisms. For instance, in the abstract/introduction, “ampere-level current densities” is informal; standard scientific phrasing such as “current densities on the order of A cm^{-2}” or simply stating the values is preferred. The text would benefit from careful proofreading.

Response: Thank you for the helpful feedback on reviewing the content. We have replaced all instances of "ampere-level" with the standard scientific phrasing "current densities on the order of A cm^{-2}." Additionally, we have gone through the entire manuscript to correct minor grammatical errors and colloquial expressions.

Question 9. The manuscript appears to self-cite a very recent publication by the same group (Ref. 4, Angew. Chem. Int. Ed.), which also investigates the role of cations in acidic electrosynthesis. The authors must explicitly and clearly delineate the novelty of the present work in relation to their previous publication. The distinction between the two studies must be sharpened to justify a separate publication.

Response: While our previous study (*Angew. Chem. Int. Ed.*) showed that K^+ ions in the electrolyte can enhance H_2O_2 electrosynthesis via the $2e^-$ ORR, this approach still faces significant practical limitations. However, the promoting effect of K^+ can be severely compromised under demanding conditions such as high current density and low pH, as it hinges on a synergy with the catalytic material and other reaction parameters (e.g., applied potential and electrolyte composition). Thus, these systems still fail to reach current densities on the order of A cm^{-2} in acidic systems. Moreover, the need for continuous metal-salt addition raises operating costs and complicates waste management.

This work shifts the focus from electrolyte additives to the catalyst material itself. We investigate a strategy of cationic modification of the carbon catalyst to unlock stable, high-performance H_2O_2 production under demanding conditions—specifically, at high current densities in acidic seawater.

(1) Through extensive comparative studies in both experimental and theoretical calculations, this work highlights the crucial role of cationic modification. Comparisons between pristine OCNT and its cationic-modified counterparts in the same acidified K_2SO_4 electrolyte reveal a significant performance enhancement, with the markedly distinct H_2O_2 efficiencies at high current densities particularly at high current densities clearly demonstrating the key role of modification. We have conducted a series of experiments to verify the crucial contribution of cationic modification to performance enhancement. This includes investigating the effects of different cationic modifiers, extending this promoting effect with cationic polymers, examining the influence of this molecular-level modification method, and comparing the results with the adverse effect induced by anionic modifiers. Cation-modified carbon catalysts maintain >90% efficiency at 500 mA cm^{-2} in natural seawater for H_2O_2 electrosynthesis, and achieve current densities up to 1.125 A cm^{-2} in high-salinity electrolytes, which far outperform the performance promoted by K^+ ions reported in our previous study.

(2) Furthermore, we performed AIMD simulations to compare the impact of OCNT and its cation-modified counterparts on the 2e$^-$ ORR reaction pathway. This study found that the electrified acidic environment over-strengthens the *OOH intermediate, which make the *OOH intermediate deviate from the optimized adsorption energy and consequently suppress H_2O_2 formation. Cationic modification counteracted this effect by weakening *OOH binding and enabling H_2O_2 generation. This cationic modification strategy effectively cooperates with diverse carbon active sites, irrespective of whether their intrinsic *OOH binding is too strong or too weak, by dynamically tuning the acidic electrified interface to promote *OOH formation and facilitate its desorption for selective 2e$^-$ ORR. In summary, the introduced cationic modifiers critically mediate the timely desorption of *OOH into solution-phase H_2O_2/ HO_2^-, thereby mitigating both the inherent suboptimal binding energy on unmodified catalysts and the potential-induced over-binding at the electrified interface.

(3) We conducted evaluations using seawater as the electrolyte, including the electrosynthesis of H_2O_2 at high current densities, an investigation of the influence of Cl^- on the 2e$^-$ ORR properties combined with theoretical calculations, as well as long-term performance and electrode stability tests, techno-economic analysis (TEA), and comparative assessments, to validate the feasibility of this technology.

Reviewer #3: This study proposes an innovative strategy for dynamically modulating acidic electrified interfaces through cation modification of carbon-based materials, aiming to achieve efficient and highly selective electrosynthesis of hydrogen peroxide in natural seawater or high-salinity wastewater. The work demonstrates exceptional performance metrics (e.g., >90% Faradaic efficiency at 500 mA cm^{-2}). However, despite its promising prospects, the current manuscript contains a series of substantive deficiencies in the validation of core mechanisms, characterization of key physical properties, and standardization of data presentation. These issues render the evidence chain supporting the central conclusions incomplete and insufficiently robust. Therefore, I recommend that this manuscript should not be accepted for publication until it undergoes Major Revision.

Response: We thank the reviewer for their positive evaluation and constructive suggestions. To address the comments regarding the validation of core mechanisms, characterization of key physical properties, and standardization of data presentation, we have supplemented the study with electronic structure analysis, additional experiments, further electrochemical characterization, and a Techno-Economic Analysis. These comprehensive revisions have been integrated to provide a more complete and robust evidential chain supporting the conclusions of the manuscript.

Question 1. The authors attribute the efficiency loss at high currents to *OOH decomposition ($4e^-$ ORR) but completely fail to exclude the competitive contribution of the hydrogen evolution reaction (HER). This lack of empirical support undermines the explanation for the root cause of the FE loss.

Response: Thank you for the reviewer's consideration regarding the hydrogen evolution side reaction. We have supplemented experimental evaluations of the H_2 production efficiency for both OCNT and OCNT-CTAB catalysts under 50-500 mA cm^{-2}. As shown in the **Figure R22**, the H_2 production efficiency for the OCNT catalyst remained below 2% up to 350 mA cm^{-2}, but increased sharply to 16% when the current was further raised to 400 and 500 mA cm^{-2}. Meanwhile, the H_2O_2 efficiency of OCNT was 63%-78% at 50–200 mA cm^{-2} and declined to 44% at 250 mA cm^{-2}. Hence, within the 50–250 mA cm^{-2} range, the $4e^-$ ORR is considered the dominant competing side reaction. At higher currents, hydrogen evolution becomes more significant. In contrast, the OCNT-CTAB catalyst maintained H_2 production below 3% across the entire 50–500 mA cm^{-2} range, demonstrating its consistently high H_2O_2 selectivity even at high current densities.

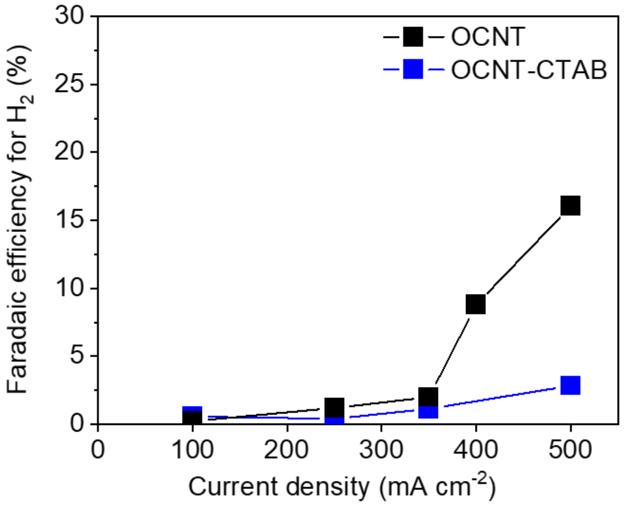

Figure R22 H_2 production efficiency for the OCNT and OCNT-CTAB catalysts at 50–500 mA cm^{-2} mA.

Action: Based on the Reviewer's comment, we have added the new figure as Supplementary Fig. 11 in the revised Supplementary Information and discussion below in the revised main text.

Line 173-178

*"The H_2O_2 efficiency of OCNT was 63-78% at 50-200 mA cm^{-2}, but declined to 44% at 250 mA cm^{-2}. This decrease was mainly attributed to $4e^-$ ORR, because H_2 production efficiency for the OCNT catalyst was detected to be less than 2% up to 350 mA cm^{-2}, and it rose sharply to 16% when further increasing current to 400 and 500 mA cm^{-2} (**Supplementary Fig. 11**). In contrast, the OCNT-CTAB catalyst maintained H_2 production below 3% at 50-500 mA cm^{-2}, demonstrating its consistently high H_2O_2 selectivity even at high current densities."*

Line 69-70 in Introduction Part

*"Sufficient acidification of the electrolyte can maintain high interfacial H^+ concentration and prevents precipitation at high current densities, however, it accelerates the competing $4e^-$ ORR pathway by influencing the adsorption of the key *OOH intermediate, and cause hydrogen evolution reaction."*

Question 2. Cationic modifiers (especially long-chain alkyl organics or polymers) are inherently insulators. The insulating organic modification layer may significantly alter electrode conductivity and mass transport structure, yet the authors have not performed any characterization of these effects.

Response: This suggestion is highly important, as it helps us further differentiate the contributions of electronic effects and mass transfer effects from cationic modifiers in the synthesis of H_2O_2. Therefore, following this advice, we conducted electrochemical impedance spectroscopy (EIS) to evaluate interfacial electron transfer and mass transport. Also, we

conducted electrochemical double-layer capacitance (Cdl) measurements to evaluate the effect of cationic modifiers on electrochemically active surface area (ECSA).

(1) We conducted electrochemical impedance spectroscopy (EIS) to probe the interfacial structure. As shown in **Figure R23**, the Nyquist plots from EIS measurements reveal that a smaller semicircle radius in the high-frequency region indicates faster electron transfer kinetics, while a steeper slope in the low-frequency region represents faster mass transport. The plots show that oxidation treatment and cationic polymer modification hardly alter these rates, possibly because the modification involves molecular-level interactions with OCNT rather than physical mixing, which does not adversely affect conductivity. Interestingly, after modification with cationic polymers, the mass transport capability was significantly enhanced, but the difference in mass transport capacity among different chain lengths was relatively small.

(2) We conducted electrochemical double-layer capacitance (Cdl) measurements to evaluate the electrochemically active surface area (ECSA). The results indicate that after oxidation treatment, the capacitance of the SWCNT significantly increased from 3.0 $mF \cdot cm^{-2}$ to 4.2 $mF \cdot cm^{-2}$ (**Figure R24**). Oxygen-doped carbon serves as electrochemical active sites, and the oxygen content in the oxidized carbon nanotubes (OCNT) is significantly increased after oxidation, leading to the marked enhancement in capacitance, which is consistent with the increased number of active sites. When OCNT was modified with the short-chain cationic surfactant MTAB, its capacitance remained nearly the same as that of OCNT (**Figure R25**). However, when modified with other long-chain cationic surfactants, the capacitance decreased to varying degrees, ranging from 2.4 to 4.3 $mF \cdot cm^{-2}$. It is speculated that long-chain cationic surfactants can interact with oxygen in OCNT, possibly by inhibiting the electron storage process involved in capacitance. A similar phenomenon was observed under modification with Quaternary ammonia poly(N-methyl-piperidine-co-p-terphenyl (QAPPT) and Sustainion imidazolium (Sus) polymers, and the effect was even more pronounced (**Figure R26**). Similarly, we also observed that polymer modification substantially reduced the capacitance of MWOCNT, with its capacitance of 0.8 $mF \cdot cm^{-2}$ being reduced to 0.5 $mF \cdot cm^{-2}$ (**Figure R27**). Thus, electrochemically active surface area analysis did not support a significant role of cationic modifier for electronic effects in the activity enhancement.

These results indicated that the factors of electron-transfer kinetics and mass-transport were also not the key factor for enhanced H_2O_2 electrosynthesis.

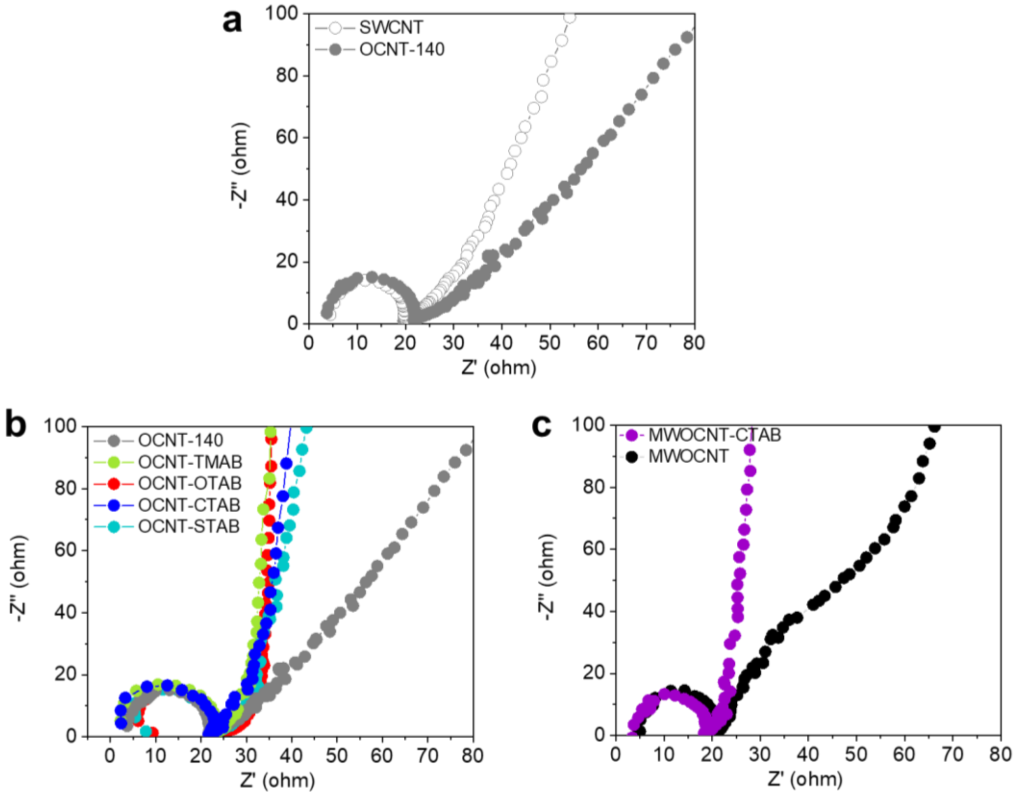

Figure R23 Electrochemical impedance spectroscopy (EIS) results. Nyquist plots of catalysts measured in 0.3 M K_2SO_4 containing 0.1 M H_2SO_4 at open-circuit voltage.

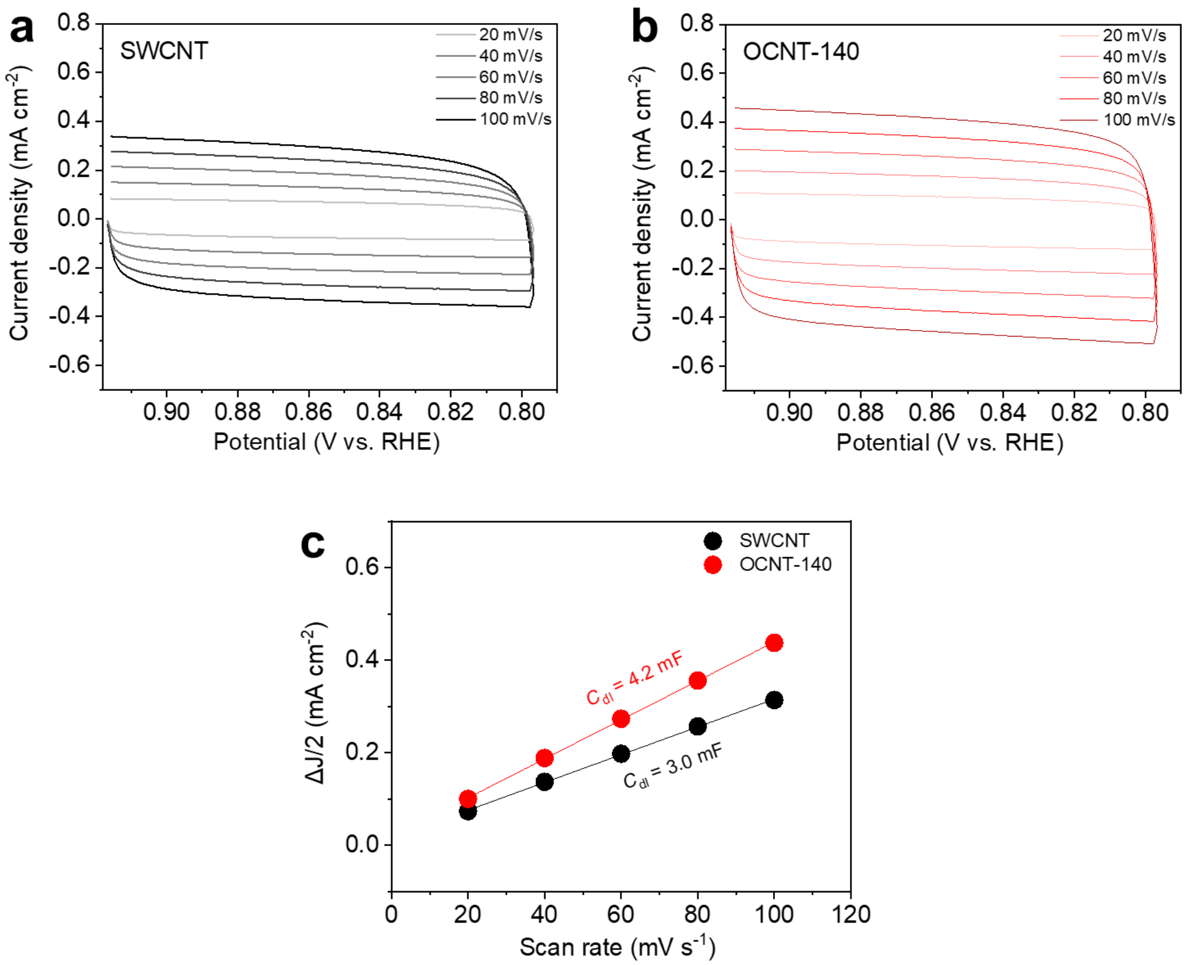

Figure R24 a-b, Cyclic voltammetry curves of SWCNT and OCNT-140 catalysts at scan rates of 20, 40, 60, 80, and 100 mV s^{-1}. c, Linear fitting of the current density at 0.86 V vs. RHE against scan rate for estimating the double-layer capacitance of the catalysts.

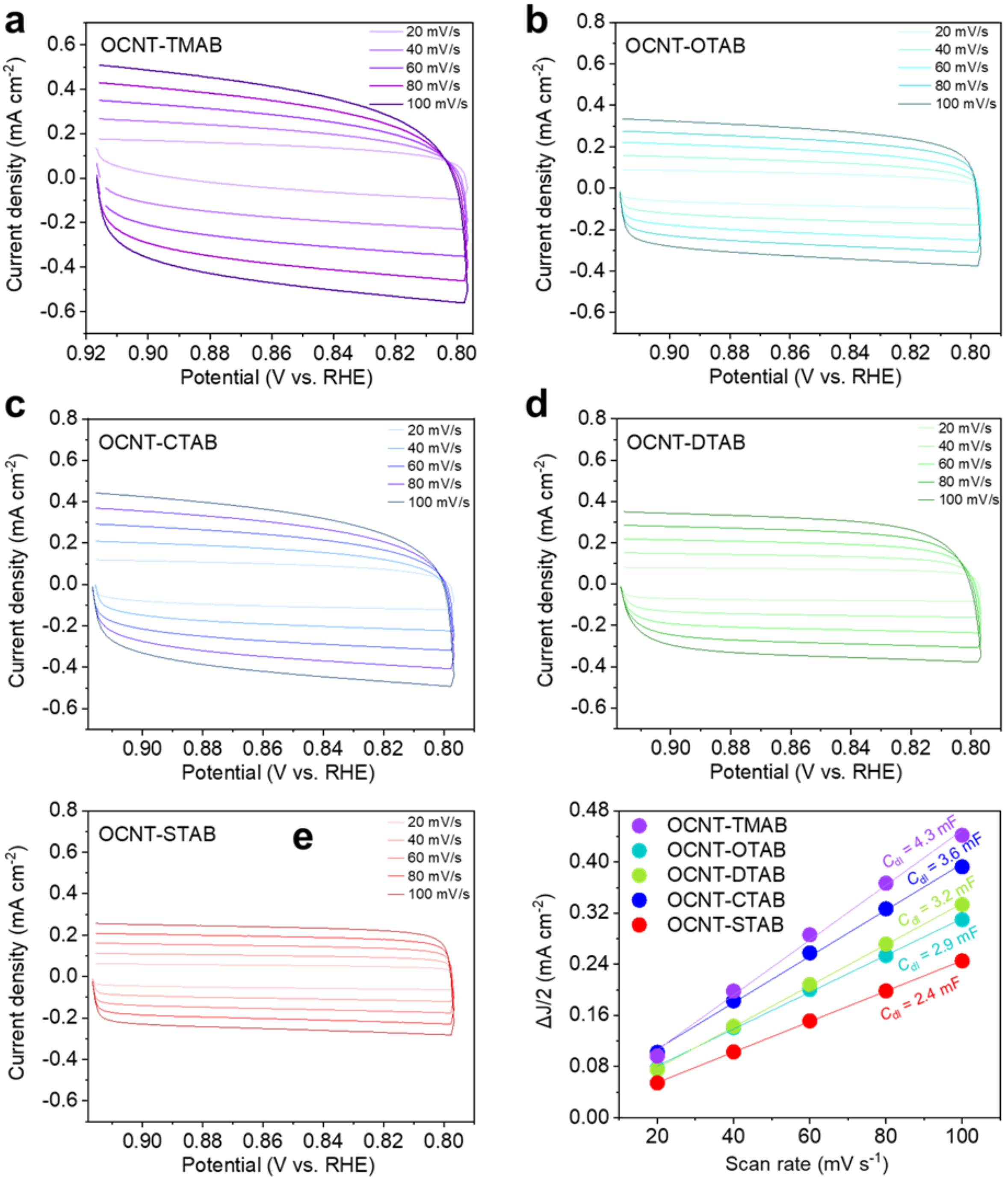

Figure R25 a-d, Cyclic voltammetry curves of OCNT-OTAB, OCNT-DTAB, OCNT-CTAB, and OCNT-STAB catalysts at scan rates of 20, 40, 60, 80, and 100 mV s^{-1}. e, Linear fitting of the current density at 0.86 V vs. RHE against scan rate for estimating the double-layer capacitance of the catalysts.

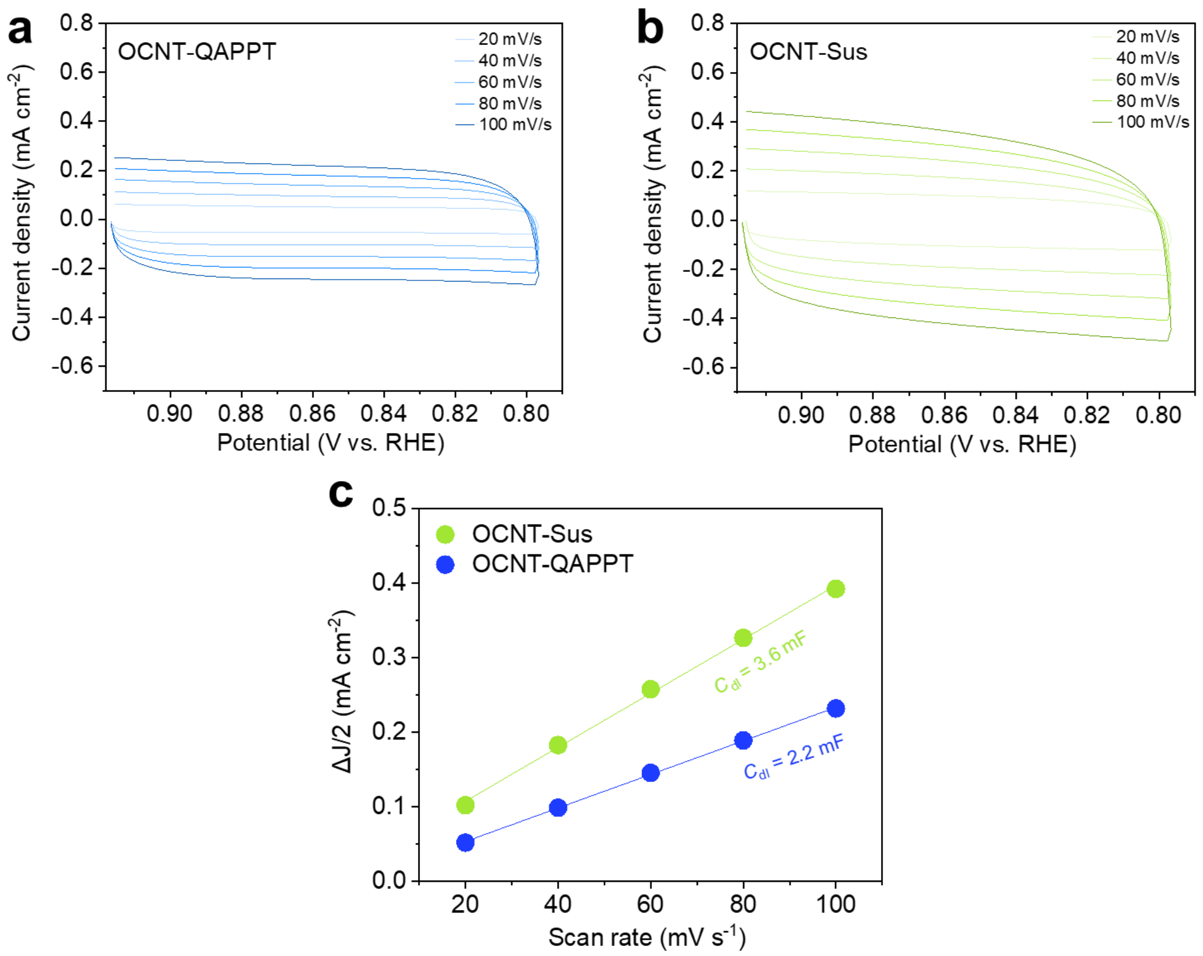

Figure R26 a-b, Cyclic voltammetry curves of OCNT-QAPPT and OCNT-Sus catalysts at scan rates of 20, 40, 60, 80, and 100 mV s^{-1}. c, Linear fitting of the current density at 0.86 V vs. RHE against scan rate for estimating the double-layer capacitance of the catalysts.

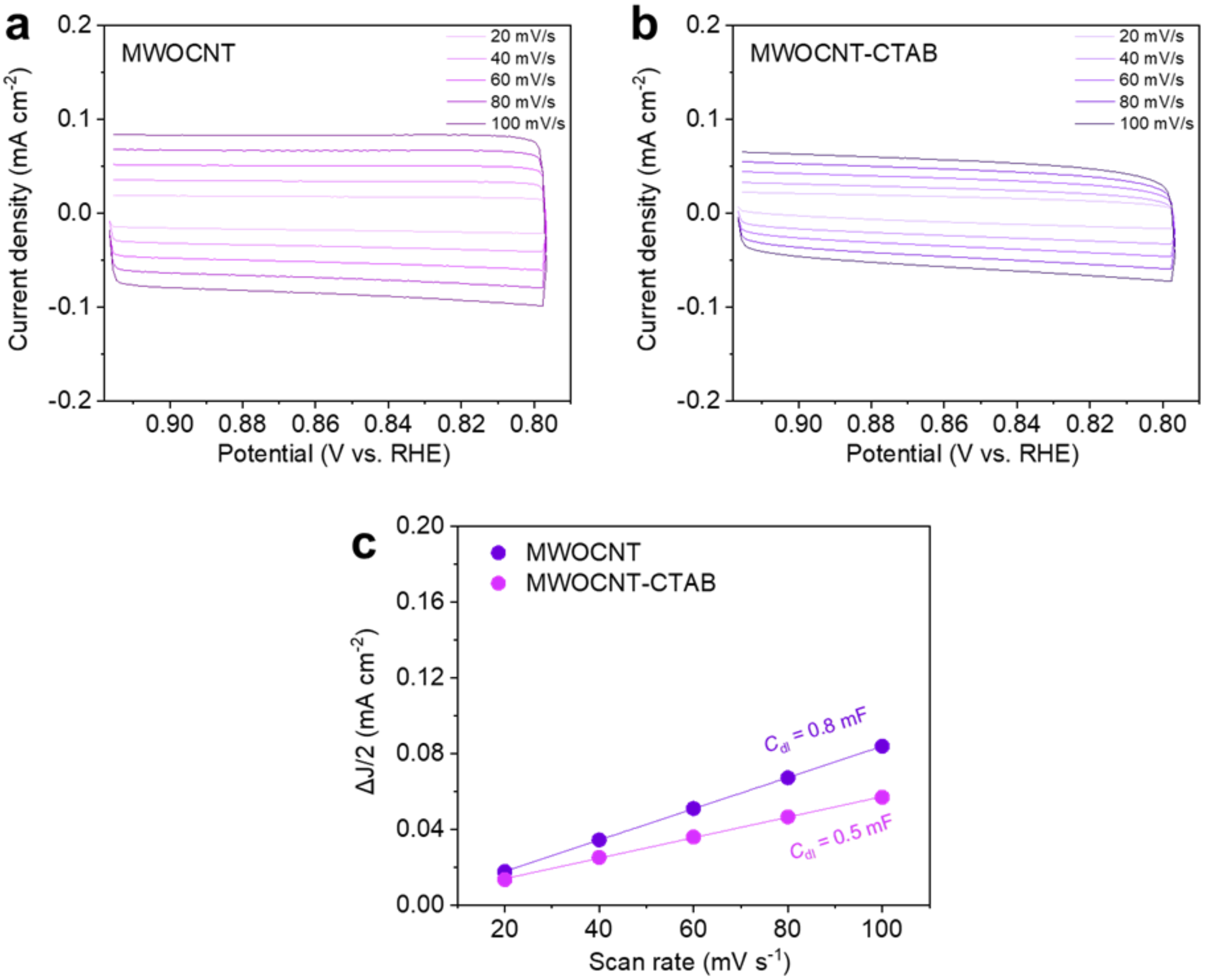

Figure R27 a-b, Cyclic voltammetry curves of MWOCNT and MWOCNT-CTAB catalysts at scan rates of 20, 40, 60, 80, and 100 mV s^{-1}. c, Linear fitting of the current density at 0.86 V vs. RHE against scan rate for estimating the double-layer capacitance of the catalysts.

Action: We have added the supplementary figures as Supplementary Figs. 25-28 in the Supporting Information, and the relevant discussion has been incorporated into the main text as shown below.

*"The roles of electronic and mass-transfer effects from the cationic modifier were evaluated. Electrochemically active surface area analysis did not support a significant role of cationic modifier for electronic effects in the activity enhancement (**Supplementary Figs. 25-27**). Electrochemical impedance spectroscopy measurements showed that electron-transfer kinetics were largely unchanged, and all modifiers induced comparable mass-transport enhancements, indicating this was also not the key factor (**Supplementary Figs. 28**)."*

Question 3. Long-term electrolysis using acidified seawater necessitates the investigation of chlorine-related byproduct formation (e.g., hypochlorite) and electrode corrosion. The absence of such data renders the conclusions regarding stability and practical applicability

unreliable.

Response: Thank you for the important suggestions regarding the influence of chlorine-related byproduct formation on the system.

The formation of chlorine-related byproducts (e.g., hypochlorite) occurs via the oxidation of Cl^-. The first possible pathway involves the reaction of Cl^- with H_2O_2, which requires a catalyst such as Fe^{2+} to promote the generation of highly oxidative hydroxyl radicals (•OH) from H_2O_2. If this were the case, the produced H_2O_2 would undergo rapid chain-reaction decomposition, and the generated •OH would oxidize the carbon-based catalyst. However, the efficient synthesis of H_2O_2 and the detection of only minimal organic carbon in the effluent (shown in **Figure R28**) suggest that the likelihood of this oxidation pathway being significantly present is relatively low. The second pathway involves Cl^- possibly crossing over to the anode, where it could be oxidized to hypochlorous acid or Cl_2. We detected no significant change in Cl^- concentration in the anode effluent under a 300mA cm^{-2} condition, indicating that the second pathway is also unlikely to be prominent.

Under acidic conditions, Cl^- ions can cause corrosion of the anode, which is a highly destructive electrochemical process by damaging the protective oxide layer/passivation film on the anode and preventing the formation and repair of the protective passive layer. In our system, chloride-containing seawater is used only in the catholyte, while the anode employs a 0.5 M H_2SO_4 electrolyte, with the two compartments separated by a proton exchange membrane. In principle, during electrolysis, only cations migrate from the anolyte to the catholyte through the membrane, with no anions or cations migrating from the cathode to the anode. No significant amounts of Cl^- ions or metal cations (Na^+, K^+, Ca^{2+}, Mg^{2+}) were detected in the anolyte, suggests that crossover effects are minimal or negligible. Therefore, anode corrosion is unlikely to be a critical factor affecting the stability and practical applicability of the system.

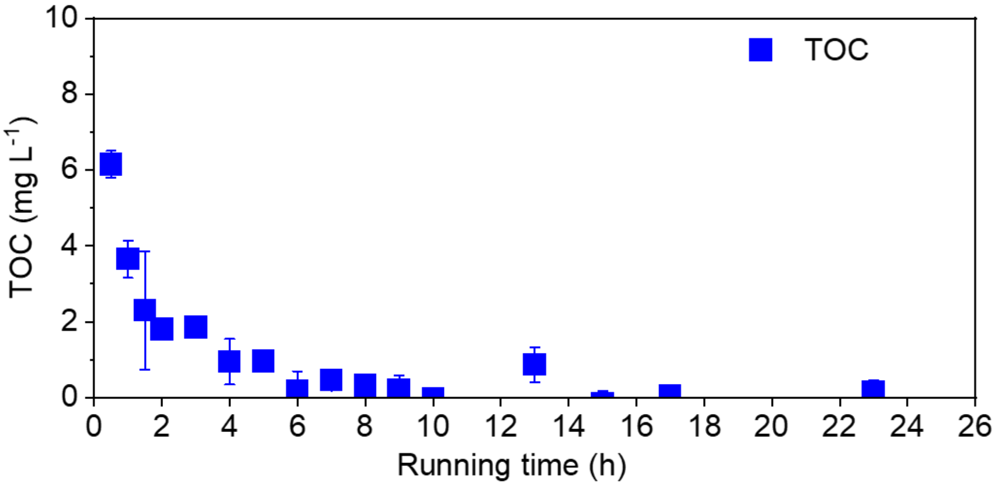

Figure R28. Total organic carbon (TOC) concentration in the effluent H_2O_2 solution during a repeated 23-hour stability electrolysis test.

Action: In response to the Reviewer's comment, we have incorporated the new figures (**Supplementary Figs 49**) into the revised Supplementary Information and added the corresponding discussion in the main text (Lines 373-379), as detailed below.

*"Electrode stability was assessed by monitoring the effluent TOC and analyzing surface properties. The TOC value of the effluent stabilized at approximately 0.5 mg L^{-1} throughout the subsequent 20-hour operation, a level consistent with that of the fresh electrolyte (**Supplementary Fig. 49**). Furthermore, post-reaction characterization via contact angle (wettability) and XPS (surface composition) measurements, the electrode only exhibited a slight reduction in hydrophobicity after use, while the CTAB modifier was shown to remain effectively loaded without significant leaching (shown in **Supplementary Fig. 50-52**)."*

Question 4. K_2SO_4 is used as the supporting electrolyte in the study. Substantial literature indicates that alkali metal cations in the electrolyte (e.g., K^+) can significantly influence the ORR pathway and suppress HER. The known effects of free K^+ in the experimental system cannot be distinguished from the contribution of the covalently grafted cations. How can the unique mechanism of the proposed strategy be demonstrated?

Response: We fully agree with the reviewer that it has been established in previous studies that K^+ ions can promote the $2e^-$ ORR while simultaneously suppressing the HER. However, the promoting effect of K^+ can be severely compromised under demanding conditions such as high current density and low pH, as it hinges on a synergy with the catalytic material and other reaction parameters (e.g., applied potential and electrolyte composition). Therefore, this work focuses on investigating the mechanisms responsible for the enhanced catalytic performance of cation-modified catalytic materials under high current densities and in acidic environments.

Through extensive comparative studies in both experimental and theoretical calculations, this work highlights the crucial role of cationic modification. Comparisons between pristine OCNT and its cationic-modified counterparts in the same acidified K_2SO_4 electrolyte reveal a significant performance enhancement, with the markedly distinct H_2O_2 efficiencies at high current densities demonstrating the key role of modification. We have conducted a series of experiments to verify the crucial contribution of cationic modification to performance enhancement. This includes investigating the effects of different cationic modifiers, extending this promoting effect with cationic polymers, examining the influence of this molecular-level modification method, and comparing the results with the adverse effect induced by anionic modifiers.

Furthermore, we performed AIMD simulations to compare the impact of OCNT and its cation-modified counterparts on the $2e^-$ ORR reaction pathway. This study found that the electrified acidic environment over-strengthens the *OOH intermediate, which make the *OOH intermediate deviate from the optimized adsorption energy and consequently suppress H_2O_2 formation. Cationic modification counteracted this effect by weakening *OOH binding and enabling H_2O_2 generation. This cationic modification strategy effectively cooperates with

diverse carbon active sites, irrespective of whether their intrinsic *OOH binding is too strong or too weak, by dynamically tuning the acidic electrified interface to promote *OOH formation and facilitate its desorption for selective 2e⁻ ORR. In summary, the introduced cationic modifiers critically mediate the timely desorption of *OOH into solution-phase H_2O_2/ HO_2^-, thereby mitigating both the inherent suboptimal binding energy on unmodified catalysts and the potential-induced over-binding at the electrified interface.

Question 5. The AIMD simulations merely illustrate phenomena; they lack in-depth analysis and quantitative evidence regarding the physicochemical nature of how cations promote desorption (e.g., electrostatic effects, solvation effects).

Response: We appreciate the reviewer's insightful comment. We agree that our initial AIMD simulations primarily illustrated the phenomenological contrast: *OOH cleavage on the bare carbon versus desorption on the OTAB-modified surface. To delve deeper into the underlying physicochemical mechanisms, particularly the role of H^+ in facilitating *OOH desorption, we have conducted in-depth trajectory analysis and electronic structure calculations, and have supplemented the following quantitative evidence and discussion.

Action: We have included the newly added experiments as **Supplementary Figs. 40-41** in the Supporting Information, and added the updated text into the revised manuscript (Line 319-340).

*"Cationic modifiers primarily function by electrostatically regulating the surface potential, we examined their effect on modulating H^+ aggregation and the ensuing hydrogenation behavior at the catalytic interface[56-58]. Previous studies indicated that during high-current-density electrolysis in acid, rapid H^+ consumption creates a proton-deficient interfacial region that can enhance H_2O_2 efficiency [22, 59, 60]. We investigated the effect of cationic modifiers on H^+ distribution at the electrode surface. Results indicated that OTAB modification can repel H^+, which occurred consistently across different carbon sites (C2, C3, C8) (**Supplementary Figs. 35-37**). This supports the notion that modifiers can influence the local proton activity during high-current-density operation. Regarding the hydrogenation behavior of H^+ towards H_2O_2, the evolution trajectory revealed that at the bare C2 site, the proximity of H^+ (as H_3O^+) to the interface induces strong interactions with *OOH, leading to its decomposition. In contrast, keeping H^+ away from the interface prevents this cleavage but was insufficient to promote efficient *OOH desorption (**Supplementary Figs. 33, 38**). By contrast, the OTAB modifier repelled H_3O^+ by shielding *O_2/*OOH, thereby suppressing their direct hydrogenation and promoting the alternative proton-transfer pathway from H_2O (**Supplementary Figs. 34** and **39**). Charge analysis reveals electron transfer from the C site to the bonding O atom in adsorbed *OOH, accompanied by O–O bond elongation on the CTAB-modified surface (**Supplementary Figs. 40-41**). The resulting weaker interaction promoted *OOH desorption rather than O–O cleavage. These results indicate that while *OOH formation is feasible under both proton-rich and -deficient conditions at highly negative*

*potentials, the reaction selectivity is ultimately determined by the cation-modulated desorption of *OOH."*

Previously: *"Previous studies have shown that high-current-density cathodic electrolysis induces a proton-depleted electrode interface due to rapid H^+ consumption[22, 56, 57]. We hypothesized that such depletion could influence *OOH desorption. Simulations of proton-free C2-H_2O interface revealed that although H^+ depletion weakened *OOH adsorption as expected, this weakening alone was insufficient to trigger significant *OOH desorption or HO_2^- release (**Fig. S28**). In contrast, cation-modified interface maintained efficient *O_2 hydrogenation and *OOH desorption even under proton-deficient conditions (**Fig. S29**). These results imply that *OOH formation remains feasible at highly negative potentials regardless of interfacial proton availability, provided that cationic modifiers maintain near-optimal binding strength. The critical determinant of selectivity is thus *OOH desorption, which is facilitated by cation-induced electronic modulation."*

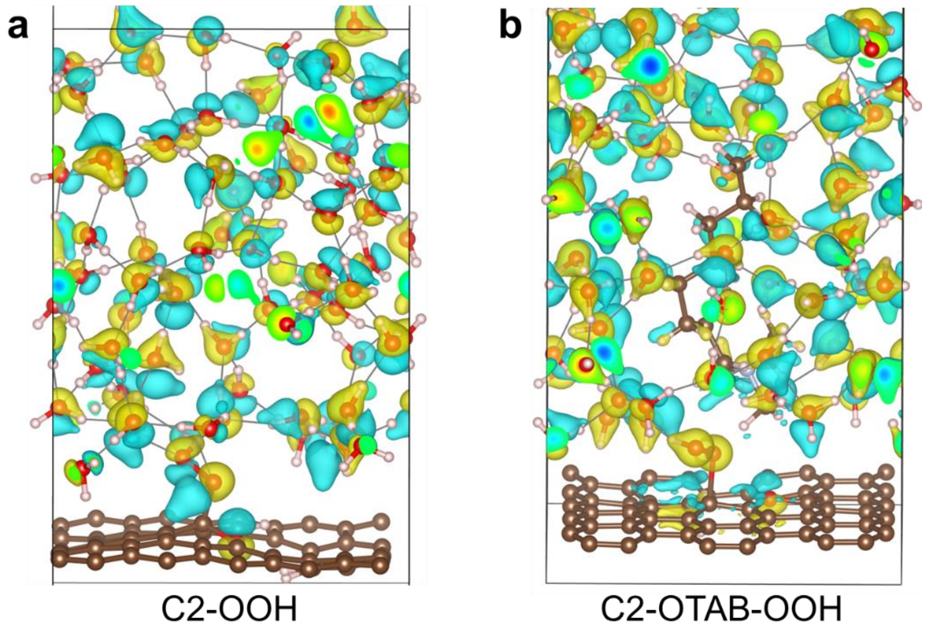

Supplementary Fig. 40 Differential charge density distributions of adsorbed *OOH on the C2-H_2O and C2-OTAB-H_2O surfaces.

In the differential charge density map, blue region indicates electron density depletion (loss), and yellow indicates accumulation (gain). The pattern indicates that upon *OOH adsorption, electron density is transferred from the catalytic C site to the interacting O atom of OOH.

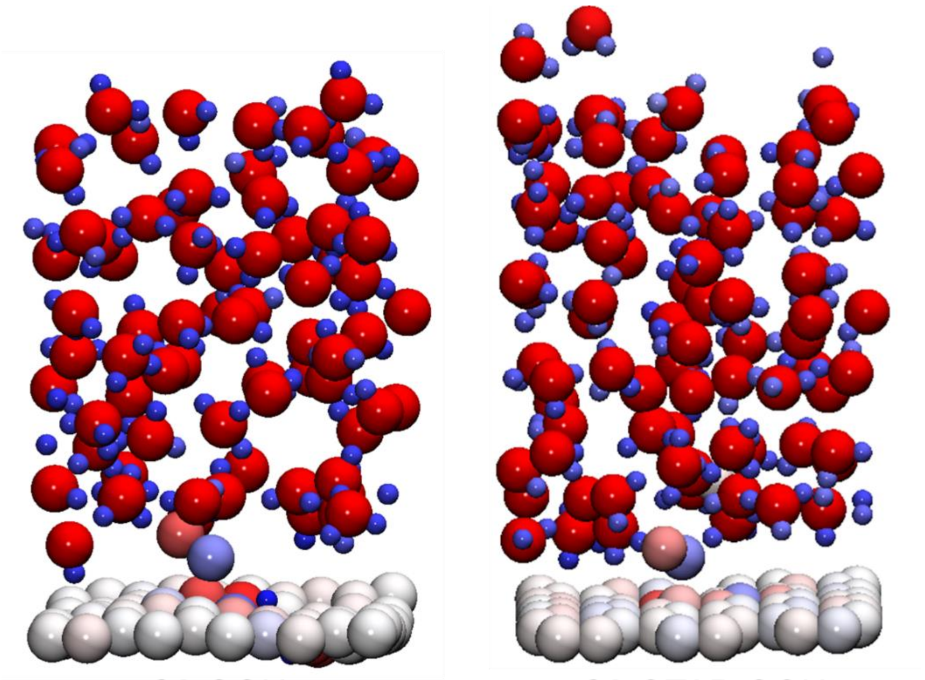

Supplementary Fig. 41 Atom charge distribution of adsorbed *OOH on the C2-H_2O and C2-OTAB-H_2O surfaces.

Red and blue indicate electron loss and gain, respectively. The results show charge depletion at the C atom and charge accumulation at the O atom of adsorbed *OOH.

Question 6. The manuscript inconsistently mixes the use of current and current density (e.g., Fig. 4a). It must be uniformly revised to use current density (mA cm^{-2}) to ensure scientific rigor and readability.

Response: Following this recommendation, we have converted all current values from mA to current density (mA cm^{-2}) and revised all current-related descriptions in the main text and Supporting Information accordingly.

Question 7. For seawater electrolysis, the current cost calculation ($0.49–0.78/kg) is overly idealized. The economic analysis should be significantly enhanced in the discussion section. At a minimum, a qualitative discussion on the potential impact of energy costs for electrolyte recycling/recovery, and the lifespan/replacement costs of key components (e.g., proton exchange membrane, electrodes) on the total cost is required to provide a more realistic and informative cost range.

Response: We appreciate the reviewer's suggestions. We have performed Techno-economic assessments (TEA) calculations under two scenarios. In the first scenario, using a K_2SO_4 electrolyte for the electrosynthesis of H_2O_2, the energy costs for electrolyte recycling/recovery are taken into account. In the second scenario, using seawater for the electrosynthesis of H_2O_2, electrolyte recycling/recovery is not considered. Moreover, the capital equipment expenditure includes the electrolyzer stack (including key components of

proton exchange membrane, electrodes) and its supporting facilities such as power supplies, pumps, compressors, separation membranes/columns, labor cost on the total cost is accounted for in order to provide a more realistic and informative cost range. The detailed calculations are presented below. In summary, the costs are \$0.64–0.98/kg and \$0.80–1.29/kg, respectively.

We have added the discussion in the revised main text () and the content below in the revised Supplementary Information.

*"To evaluate the economic feasibility of H_2O_2 electrosynthesis, we conducted a techno-economic assessment (TEA) comparing two scenarios: using K_2SO_4 electrolyte and using seawater. The analysis encompassed operating costs, capital expenditures, and labor; detailed calculations are provided in the Supporting Information. For the K_2SO_4-based process, the cost assessment includes H_2O_2 separation and electrolyte recycling. Here, acidic H_2O_2, which remains stable up to 95 °C is suitable for distillation, unlike alkaline H_2O_2 that decomposes rapidly (**Supplementary Fig. 74**). For the seawater-based process, while electrolyte recycling was omitted, costs for acid pretreatment and neutralization of the acidic waste stream were included. The lowest achievable H_2O_2 production costs are estimated at \$0.64/kg and \$0.80/kg for the K_2SO_4 and seawater case, respectively. Collectively, our results demonstrate both the technical feasibility and economic competitiveness of this approach for potential industrial-scale H_2O_2 production."*

Techno-economic assessments (TEA)

Regarding the TEA analysis of the electrolysis technology, the U.S. DOE developed and used the H2A Production Model (H2A) framework for the techno-economic analysis of hydrogen production pathways. The model includes major inputs such as capital costs, operation and maintenance, different fuel types, and financial considerations such as dollar year, plant life, and internal rate of return. Therefore, our cost analysis for the electrosynthesis of H_2O_2 references the calculation principles of the H2A model.

The technical and economic factors for H_2O_2 electrosynthesis include: operating costs, capital equipment expenditure, and labor cost.

4. The analysis is conducted with the production cost per unit weight of H_2O_2 (\$/kgH_2O_2) as the metric. H_2O_2 electrosynthesis at the cathode, at its most basic level, requires O_2, H_2O and electricity to produce H_2O_2 along with by-product O_2 at the anode which may serve as a supply of cathode. The system boundary encompasses raw materials (water, oxygen, electrolyte replenishment), and electrical energy consumption. There are 8000 hours of operation every year, and the rest is used for equipment maintenance.

Supplementary Table 8 Operating costs for H_2O_2 production using OCNT-CTAB as the cathode and IrTaTi as the anode with 4 cm^2 working area.

Current density mA cm^{-2}	Yield rate $*10^{-3}$ kg/h	Electricity cost kwh/kgH_2O_2	O_2 cost \$/kgH_2O_2	H_2O cost \$/kgH_2O_2	Total operating cost \$/kgH_2O_2
250	0.52859	0.40539	0.1287	0.05405	0.58814

500	1.01293	0.59234	0.06716	0.02821	0.68771
750	1.58307	0.71741	0.04297	0.01805	0.77843
1000	1.98707	0.92023	0.03423	0.01438	0.96885

Supplementary Table 9 Estimated operating costs for H_2O_2 production per year when working area was scaled up 500 m^2.

Current density mA cm^{-2}	Yield rate kg/h	Output ton/year	Total operating cost $/year
250	660	5285	3108849
500	1266	10129	6966020
750	1978	15830	12323091
1000	2483	19870	19251727

Note: the calculations of electricity cost, O_2 cost and H_2O cost are shown above.

5. Capital equipment expenditure includes the electrolyzer stack and its supporting facilities such as power supplies, pumps, compressors, separation membranes/columns, etc. The electrolyzer stack cost accounts for 65% of the total capital expenditure, with the balance of plant (BOP) comprising the remaining 35%. Their installation and infrastructure costs are typically a multiple of the equipment cost, e.g., 1.2 times. It also includes product separation and purification costs, and the capital cost of membrane separation is estimated based on distillation.

$$\text{Electrolyzer cost} = 500\ m^2 \times \$1619\ m^{-2} \times 1.2 = \$971{,}400 \quad (8)$$

$$\text{BOP cost} = \text{Electrolyzer} \div 65\% \times 35\% = \$523{,}061 \quad (9)$$

$$\text{Separation cost} = \$10664000 \times \left(\frac{\text{production rate L min}^{-1}}{1000\ \text{L min}^{-1}}\right)^{0.7} \times 0.7 \times 0.1 = \$1{,}657{,}346 \quad (10)$$

$$\text{Equipment Cost} = \text{Base Cost} \times \left(\frac{\text{Throughput}}{\text{Base Throughput}}\right)^{\text{Scaling Factor}} \times \text{Process Cost Correction Factor} \quad (11)$$

This part is estimated referring "H2A Production Model Version 3 2018 User Guide Draft". Process Cost Correction Factor: refers to the cost adjustment coefficient of a specific process (membrane separation) relative to the benchmark process (distillation). If the scaling factor exponent is 1.0, it means that the growth rate of equipment cost is exactly proportional to the growth rate of plant capacity. The scaling factor exponent is typically 1.0 or less. If the scaling factor exponent is 0.7, then the increase in equipment cost is proportional to the increase in plant capacity raised to the 0.7 power. Scaling factor reference (distillation column): The scaling factor for the distillation method typically ranges from 0.6 to 0.7.

$$\text{Depreciation cost}=\frac{(\text{Electrolyzer cost + BOP cost+ Separation cost})\times 3.25\%}{1-\frac{1}{(1+3.25\%)^{20}}}$$

$$=\$233{,}452/\text{year} \quad (12)$$

The maintenance cost is assumed 2.5% of capital cost per year (from H2A):

$$\text{Maintenance cost = Depreciation cost} \times 2.5\% = \$5{,}836/\text{year} \quad (13)$$

$$\text{Capital equipment expenditure = Depreciation cost+ Maintenance cost}$$
$$=\$239{,}288/\text{year} \quad (14)$$

6. Labor cost

According to 2025 data from the National Bureau of Statistics of China, the average annual income for manufacturing employees in urban non-private units in 2024 was 15,428$; while for manufacturing employees in urban private units, the average annual income was 10,209$. Therefore, the annual labor cost is estimated to be $50,000 based on an average of 3-5 personnel responsible for operating the project.

Supplementary Table 10 Estimated total costs for H_2O_2 production per year when working area was scaled up 500 m^2.

Current density mA cm^{-2}	Total operating cost $/year	Capital equipment expenditure $/year		Operational cost accounting $/year	Output ton/year	Total $/kg
		Depreciation cost $/year	Maintenance cost $/year			
250	3108849	233,452	5,836	50,000	5285	0.64298
500	6966020	233,452	5,836	50,000	10129	0.71629
750	12323091	233,452	5,836	50,000	15830	0.79674
1000	19251727	233,452	5,836	50,000	19870	0.98344

Notes: After the electrosynthesis of H_2O_2 in a salt solution, product separation and electrolyte recovery present a key challenge. Possible solution strategies include post-processing methods such as concentration and salting-out or integrated separation, which couples with the electrolyzer.

(1) Concentration and salting-out methods, such as vacuum distillation, concentrate the electrolyte to crystallize out the salt or obtain a more concentrated H_2O_2 solution based on solubility differences. The costs involve: (1) Capital investment for the vacuum system, evaporator, condenser, and heat pump. (2) High operational energy costs, dominated by thermal energy, which is typically high. (3) Maintenance costs for addressing equipment corrosion and scaling. Economic Profile: The initial investment might be lower than for complex membrane systems. However, the absolute value of operational energy consumption is very high, constituting the major cost component. This method is not suitable for alkaline H_2O_2 solutions, as it leads to rapid decomposition and a consequent decrease in effective product yield. The above cost assessment for acidic H_2O_2 product separation adopts this method.

(2) Integrated separation couples the electrolyzer with other separation processes (e.g., electrodialysis) to simultaneously produce H_2O_2 and separate or concentrate the salt. The cost structure involves: (1) Capital investment for specialized equipment, including the membrane stack, electrodes, and control system. (2) Operational energy costs, primarily the electrical power required to drive ion migration. (3) Additional expenses for membrane cleaning and replacement due to fouling or damage. Economic Profile: The initial investment may be relatively high. However, operational energy consumption is comparatively lower and more controllable. The system shares similarities with the H_2O_2 electrosynthesis setup, potentially reducing the need for an entirely set of additional equipment.

There is another scenario in which the recovery and reuse of electrolytes (e.g., seawater) are not considered when doing so is economically impractical. Instead, the cost of the electrolyte is included in the total production cost. In such cases, subsequent issues related to H_2O_2 application and waste stream disposal, such as the disposal of acidic solutions and the cost of neutralizing acidic waste, must also be addressed. Accordingly, the TEA factors for H_2O_2 electrosynthesis are calculated as follows.

4. The operating cost for H_2O_2 production includes the costs for O_2, electricity, and pre-treatment and post-treatment of electrolyte.

Supplementary Table 11 Operating costs for H_2O_2 production in seawater using OCNT-CTAB as the cathode and IrTaTi as the anode with 4 cm^2 working area.

Current density mA cm^{-2}	Yield rate $*10^{-3}$ kg/h	Electricity cost kwh/kgH_2O_2	O_2 cost \$/kgH_2O_2	Treatment cost \$/kgH_2O_2	Total operating cost \$/kgH_2O_2
50	0.11607	0.2954	0.58611	0.27762	1.15913
150	0.31304	0.4381	0.21731	0.10293	0.75835
250	0.52635	0.58353	0.12924	0.06122	0.774
350	0.74268	0.67323	0.0916	0.04339	0.80822
450	0.90684	0.8365	0.07502	0.03553	0.94705
500	1.008	0.86451	0.06749	0.03197	0.96397

Supplementary Table 12 Estimated operating costs for H_2O_2 production in seawater per year when working area was scaled up 500 m^2.

Current density mA cm^{-2}	Yield rate kg/h	Output ton/year	Total operating cost \$/year
50	145	1160	1345402
150	391	3130	2373938
250	657	5263	4073949
350	928	7426	6002488
450	1133	9068	8588228
500	1260	10080	9716817

Note: the calculation of electricity cost, O_2 cost and H_2O cost is shown above.

5. Capital equipment expenditure

$$\text{Electrolyzer cost} = 500\ \text{m}^2 \times \$1619\ \text{m}^{-2} \times 1.2 = \$971{,}400 \quad (8)$$

$$\text{BOP cost} = \text{Electrolyzer} \div 65\% \times 35\% = \$523{,}061 \quad (9)$$

$$\text{Depreciation cost} = \frac{(\text{Electrolyzer cost} + \text{BOP cost}) \times 3.25\%}{1 - \frac{1}{(1+3.25\%)^{20}}} = \$102{,}787/\text{year} \quad (15)$$

The maintenance cost is assumed 2.5% of capital cost per year (from H2A):

$$\text{Maintenance cost} = \text{Depreciation cost} \times 2.5\% = \$2{,}570/\text{year} \quad (16)$$

$$\text{Capital equipment expenditure} = \text{Depreciation cost} + \text{Maintenance cost} = \$105{,}357/\text{year} \quad (17)$$

6. Operational cost accounting

Consistent with the approach above, the cost here is calculated in the same manner. Therefore, the annual labor cost is estimated at $50,000 based on an average of 3 to 5 personnel required for project operation.

Supplementary Table 13 Estimated total costs for H_2O_2 production in seawater per year when working area was scaled up 500 m^2.

Current density mA cm^{-2}	Total operating cost $/year	Capital equipment expenditure $/year		Operational cost accounting $/year	Output ton/year	Total $/kg
		Depreciation cost $/year	Maintenance cost $/year			
50	1345402	102,787	2,570	50,000	1160	1.29376
150	2373938	102,787	2,570	50,000	3130	0.80808
250	4073949	102,787	2,570	50,000	5263	0.80359
350	6002488	102,787	2,570	50,000	7426	0.82923
450	8588228	102,787	2,570	50,000	9068	0.96422
500	9716817	102,787	2,570	50,000	10080	0.97938

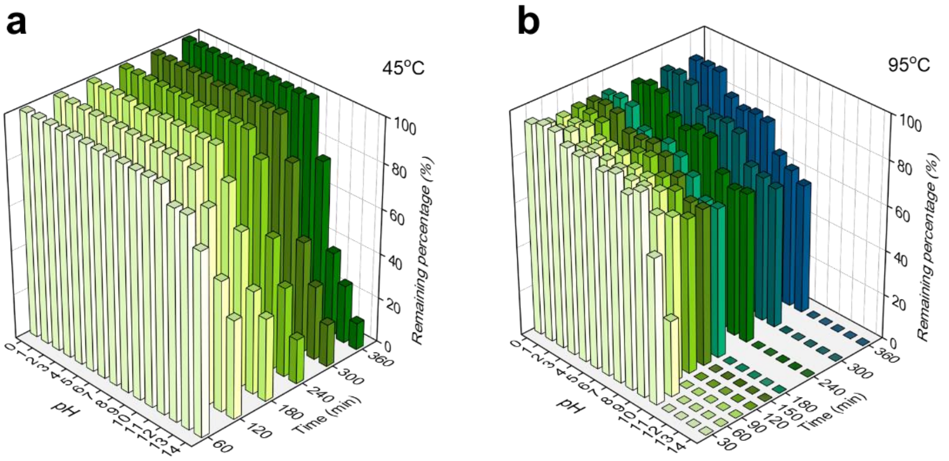

Supplementary Fig. 74 The retention of 1 wt% H_2O_2 at 45°C and 95°C as a function of solution pH.
